# β C–H di-halogenation *via* iterative hydrogen atom transfer†
†Electronic supplementary information (ESI) available. CCDC 1581032. For ESI and crystallographic data in CIF or other electronic format see DOI: 10.1039/c8sc01214h


**DOI:** 10.1039/c8sc01214h

**Published:** 2018-04-30

**Authors:** Ethan A. Wappes, Avassaya Vanitcha, David A. Nagib

**Affiliations:** a The Ohio State University , Department of Chemistry and Biochemistry , Columbus , OH 43210 , USA . Email: nagib.1@osu.edu

## Abstract

A radical relay strategy for mono- and di-halogenation (iodination, bromination, and chlorination) of sp^3^ C–H bonds has been developed. This first example of β C–H di-halogenation is achieved through sequential C–H abstraction by iterative, hydrogen atom transfer (HAT). A double C–H functionalization is enabled by *in situ* generated imidate radicals, which facilitate selective N˙ to C˙ radical translocation and tunable C–X termination. The versatile, geminal di-iodide products are further elaborated to β ketones and vinyl iodides. Mechanistic experiments explain the unique di-functionalization selectivity of this iterative HAT pathway, wherein the second C–H iodination is twice as fast as the first.

## Abstract 2

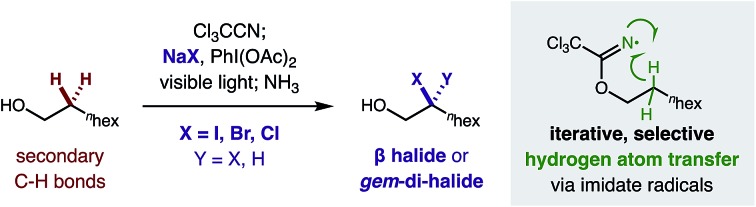
A radical relay strategy for mono- and di-halogenation (iodination, bromination, and chlorination) of sp^3^ C–H bonds has been developed. This first example of double, geminal C–H functionalization is enabled *via* iterative, hydrogen atom transfer (HAT) by *in situ* generated imidate radicals.

## Introduction

**Fig. 1:**
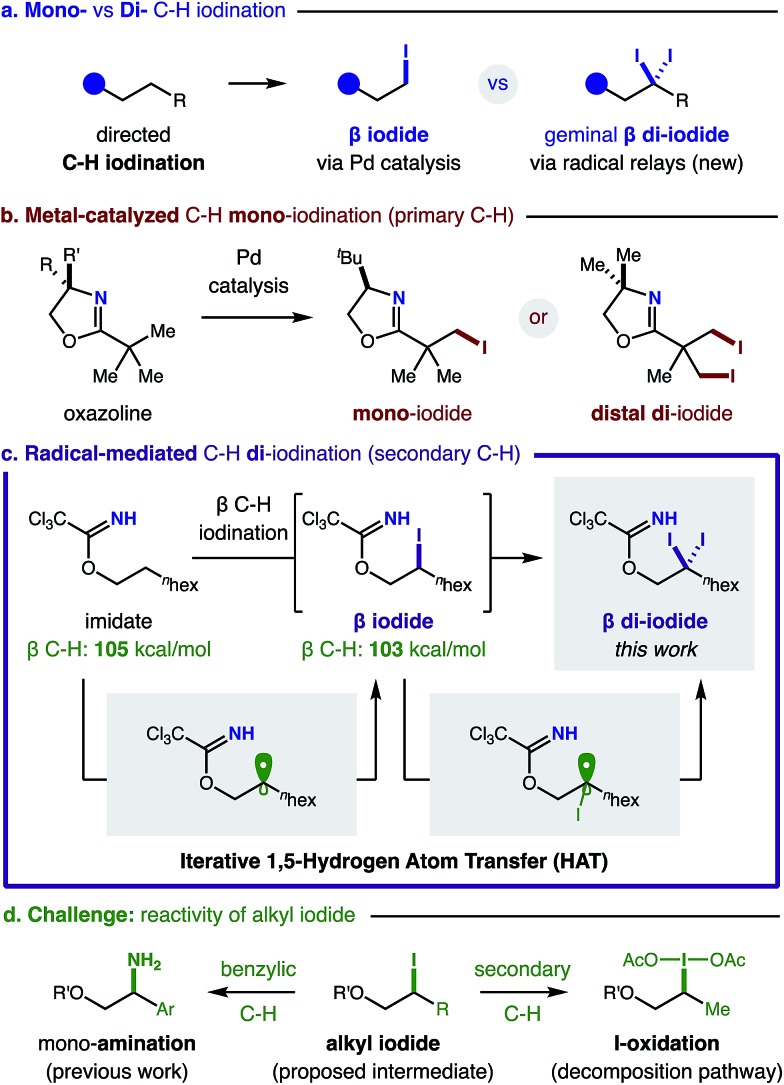
Directed, mono- and di-iodination of sp^3^ C–H bonds.

The halogenation of an sp^3^ C–H bond^1^ enables direct conversion of an inert motif into a versatile synthetic handle that permits broad reactivity *via* cross-coupling and substitution.^2^ Generally, C–H halogenation occurs by radical-mediated^3^ or organometallic^4^ mechanisms. Each approach exhibits complementary reactivity and selectivity – especially for incorporation of the most versatile halide: an iodide (Fig. 1a). In the realm of metal-mediated sp^3^ C–H iodination, there are just a few methods that can install this reactive handle; they are stoichiometric^5^ or catalytic^6^ in Pd. In the latter cases, only Yu and Rao have reported directed sp^3^ C–H iodination – employing oxazolines, amides, or oximes as directing groups (Fig. 1b).^6^ These Pd-catalyzed methods exclusively effect primary C–H conversion to a terminal mono-iodide, which is deactivated to further reactivity. In this mechanism, a second iodination at a distal, primary C–H affords a 1,3-di-iodide.^7^

Alternatively, radical mechanisms can promote efficient iodination of various types of sp^3^ C–H bonds *via* hydrogen atom transfer (HAT).^8^ Moreover, intramolecular HAT provides unique, δ selective C–H functionalizations.^9^ Yet, non-directed methods^10^ surpass the few, pioneering examples of δ (or γ) C–H halogenation.^11^ Notably, a directed C–H iodination has yet to be developed, despite the key intermediacy of a distal iodide in several δ C–H aminations (or etherification) mediated by 1,5-HAT.^12^ Due to the penchant for iodide displacement, intercepting this alkyl iodide intermediate is challenging. As an alternate strategy, we proposed a cascade mechanism – involving abstraction of the adjacent, α-iodo C–H – might enable geminal C–H di-iodination (Fig. 1c).

We noted that Suárez observed a minor di-iodide byproduct upon intramolecular δ amination of 8-membered lactams.^13^ Benzylic tri-iodination mechanisms have also been proposed,^14^ but no method yet exists to isolate them.

Given the limited synthetic accessibility (and potential pharmacological value^15^) of *gem*-di-iodides – an important, versatile motif (previously only accessible from hydrazones or vinyl iodides)^16^ – we sought to design a strategy to harness a directed, iterative HAT mechanism to introduce geminal di-halides at remote carbons. Notably, this new type of double C–H iodination at a single carbon atom is complementary to Pd-catalyzed methods and uniquely possible *via* a radical mechanism (Fig. 1).

To develop a versatile β C–H di-iodination *via* iterative, intramolecular HAT and sequential iodination, we chose to employ imidates as readily accessible, radical relay precursors (Fig. 1c). In our proposed di-iodination mechanism, we envisioned that *in situ* formation of a weak imidate sp^2^ N–I bond would enable its rapid homolysis by visible light. Selective translocation of the ensuing N-centered radical to a β C˙ can occur *via* thermodynamically favored 1,5-HAT. Finally, either radical recombination with I˙ (derived from the initial N–I homolysis), or homolytic substitution by I_2_ (or N–I), can afford a reactive β iodide. However, we were cognizant of two major challenges (Fig. 1d) for trapping the δ iodide intermediate of HAT mechanisms, including its reactivity: (1) as a leaving group, and (2) towards further oxidative decomposition.

Whereas, we previously observed weak C–H bonds (*e.g.* benzyl, allyl) provide activated iodides that are rapidly displaced (in a formal C–H amination),^17^ secondary (2°) C–H bonds yield complete decomposition. Given our knowledge that I_3_^–^ efficiently mediates HAT of 2° C–H bonds,^18^ we hypothesized a β iodide intermediate is formed, yet is prone to further I-oxidation. In this case, decomposition may ensue from the resulting sp^3^ hypervalent iodide, which is an excellent nucleofuge for elimination or cyclization.^19^ Instead, to enable access to *gem*-di-iodides, we proposed an alternate N-selective oxidation may promote a second HAT of the slightly weaker β C–H (103 *vs.* 105 kcal mol^–1^).^20^ Importantly, however, this iterative HAT mechanism for directed, di-functionalization is only possible if N-oxidation is more rapid than the previously observed, I-oxidation pathway.

## Results and discussion

To our delight, adaptation of our radical relay strategy allowed us to intercept the 2° β iodide intermediate for the first time to access both mono- and di-β C–H iodides. The key factors that enabled discovery of these new reactions included judicious choice of oxidant, increased reaction concentration, and shorter reaction duration – all essential to limit product decomposition. Notably, NIS oxidant was found to favor β mono-iodide **1** formation, while a combination of NaI and PhI(OAc)_2_ provides desired β di-iodide **2–17**. For the latter, a strong solvent effect was also observed, wherein greater solubility of NaI (in HFIP or CH_2_Cl_2_) affords less product (**3**, <30%), while more polar, but less solubilizing MeCN affords a higher yield of β di-iodide **3** (58%). Ultimately, a 3 : 1 mixture of CH_2_Cl_2_ : MeCN was found to provide the *gem*-di-iodide most efficiently (**3**, 88%, 83% isolated yield) (see ESI† for full details of optimization).

Having developed the first method for β C–H di-iodination, we next investigated the generality of this radical-mediated transformation with a variety of imidates – derived from base-induced addition of alcohols into Cl_3_C–CN. In all cases, we observed efficient formation of β di-iodides with greater than 20 : 1 regioselectivity (Table 1).

**Table 1:** β C–H mono- and di-iodination of imidates *via* a radical relay strategy

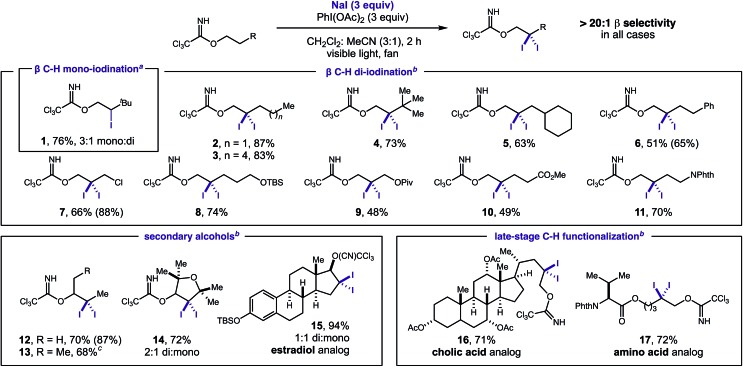

^*a*^Conditions: C–H mono-iodination: NIS (1 equiv.), MeCN, visible light (26 W CFL).

^*b*^Conditions: C–H di-iodination: NaI (3 equiv.), PhI(OAc)_2_ (3 equiv.), 3 : 1 CH_2_Cl_2_ : MeCN, visible light (26 W CFL).

^*c*^Conditions: 2 equiv. NaI and PhI(OAc)_2_; <10% distal di-iodide. Isolated yields. ^1^H NMR yields in parenthesis.

Except for the NIS-based conditions that afford mono-iodide **1**, di-iodide is always the major product, typically isolated in high yields (**2–3**). Interestingly, this reaction is tolerant of steric congestion (**4–5**) and remains β selective even in the presence of weaker C–H bonds adjacent to arenes, halides, ethers, esters, and amides at the γ or δ positions (**6–11**). Secondary alcohols are also amenable to this di-iodination with selectivity observed for secondary over primary C–H bonds (**12**) – in contrast to Pd-mediated pathways.^6^ While acyclic 2° alcohols efficiently yield di-iodide (**13**), cyclic alcohols afford a 2 : 1 mixture of di- and mono-iodide (**14**) – illustrating conformational constraints for the HAT mechanism. Similarly, an estradiol-derived imidate affords a 1 : 1 mixture of mono- and di-iodide (**15**). Imidates derived from cholic acid and amino acid, valine, yield *gem*-di-iodides (**16–17**) efficiently.

Cognizant of the synthetic utility of *gem*-di-halides, we sought to extend this unique di-iodination mechanism to other halides. To this end, we found that the use of NaBr or NaCl (instead of NaI) affords analogous β halogenation (Table 2). These new transformations require slight deviation from standard reaction conditions since NaBr and NaCl are less soluble. In these cases, increased halide concentration *via* phase transfer catalysts (Bu_4_N^+^X^–^) and a more solubilizing solvent mixture (3 : 1 HFIP : CH_2_Cl_2_) are the key factors that enable these new reactions.

**Table 2:** β C–H bromination and chlorination

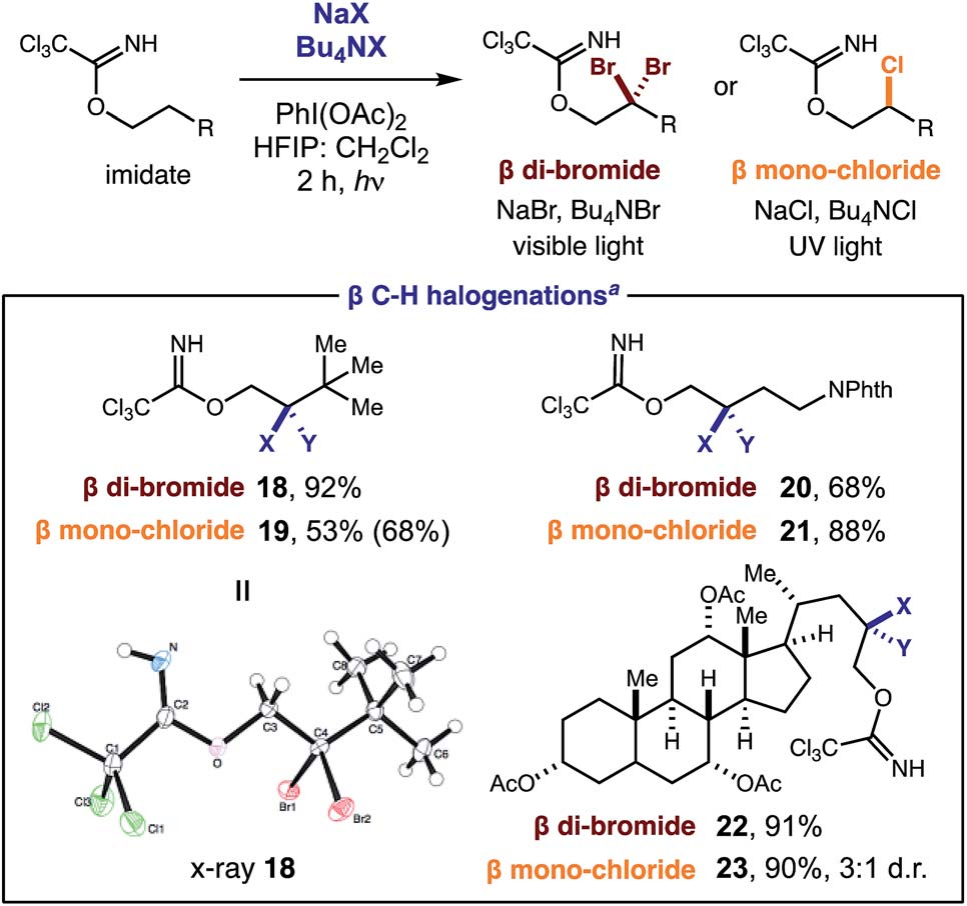

^*a*^C–H di-bromination: NaBr (3 equiv.), Bu_4_NBr (1 equiv.), PhI(OAc)_2_ (3 equiv.), 3 : 1 HFIP : CH_2_Cl_2_, visible light (26 W CFL). C–H mono-chlorination: NaCl (3 equiv.), Bu_4_NCl (1 equiv.), PhI(OAc)_2_ (3 equiv.), 3 : 1 HFIP : CH_2_Cl_2_, UV light (300 nm). Isolated yields. ^1^H NMR yields in parenthesis.

Notably, a stronger N–Cl intermediate requires UV light (300 nm) for initiation of the radical relay. It is also noteworthy that C–H chlorination ceases after the first halogenation despite a relative similarity in the α-Cl and α-Br C–H bond strengths (±1 kcal).^21^ The scope is as general as the iodination, with three representative examples shown for each halide (**18–23**). X-ray crystallographic analysis of di-bromide **18** confirms the structure of these distal geminal halides.

**Fig. 2:**
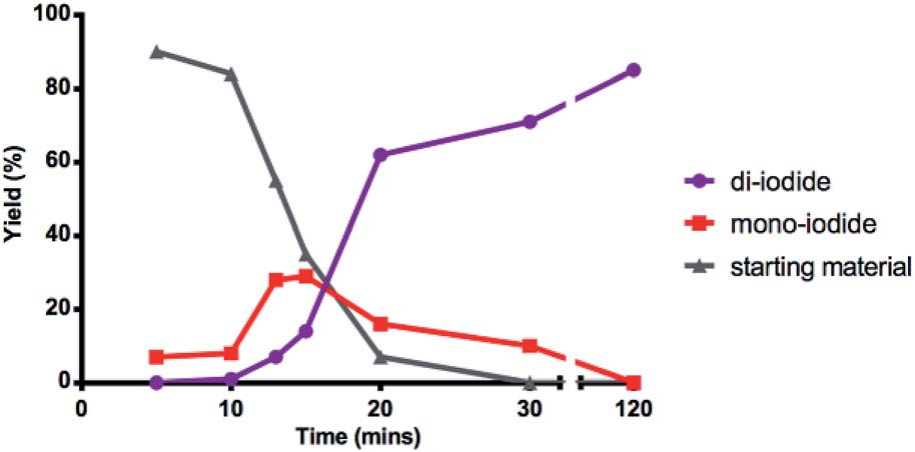
Kinetics of mono and di C–H iodination.

Interested in further understanding this exceptionally efficient sequential di-iodination (which provides orthogonal reactivity and selectivity to Pd catalysis), we sought to explore our hypothesis that the weaker α-iodo C–H bond enables this transformation. First, a kinetic study by ^1^H NMR illustrates a rapid conversion of the mono-iodide intermediate to the di-iodide product (Fig. 2). After an initial induction period (*ca.* 10 min), mono-iodide **24** is formed in ∼30% yield, before rapid conversion to di-iodide **2**.

**Fig. 3:**
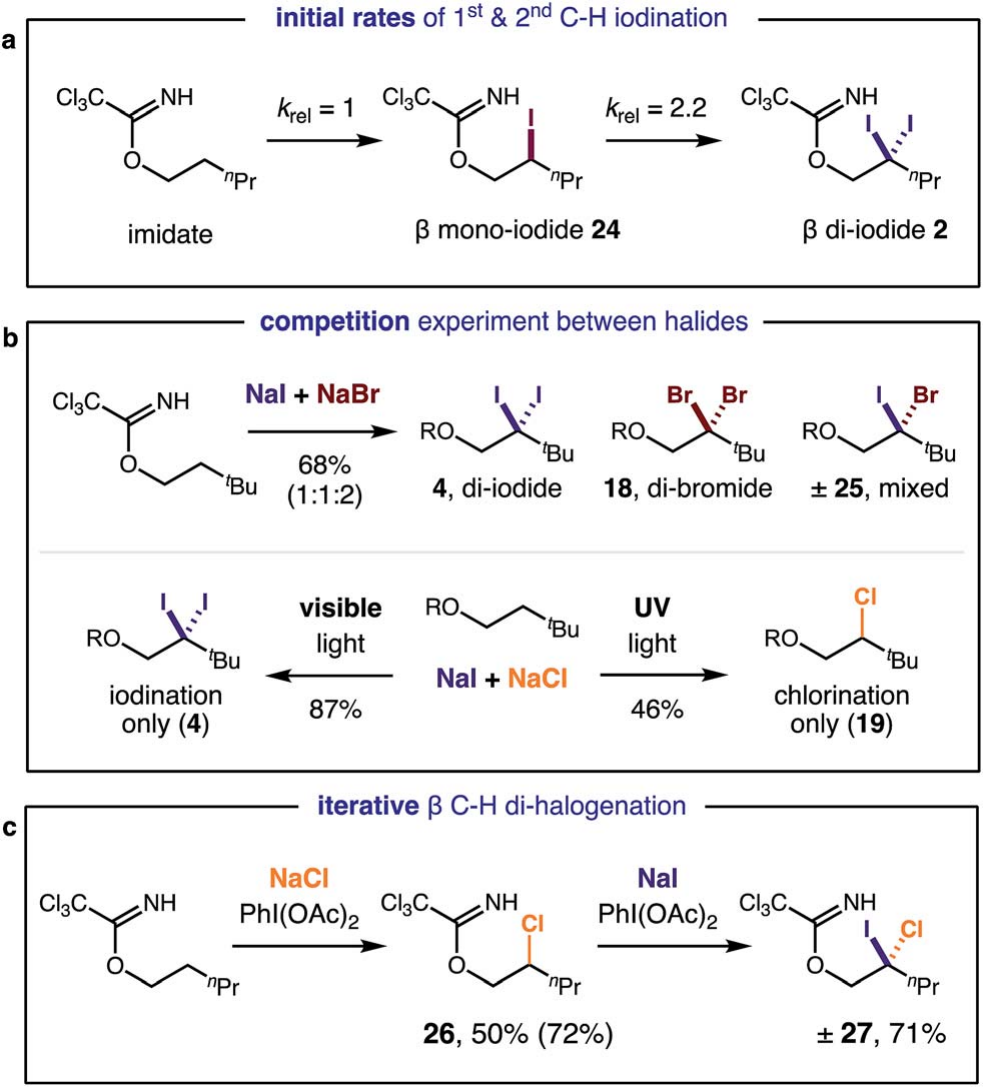
Mechanistic experiments: (a) initial rates of mono *vs.* di C–H iodination; (b) competitive and (c) iterative C–H halogenation.

In separate experiments, initial rates of formation of mono-iodide **24** and di-iodide **2** were independently measured from their respective starting materials (Fig. 3a), using 1 equiv. of oxidant, for more accurate measurements. A relative rate of 2.2 was observed in the second iodination, supporting the expectation it is more rapid than the first due to a weaker C–H bond. In the course of our studies, we were also interested in comparing the relative rates of reactivity among the various halides. To this end, we performed competition experiments between NaI & NaBr/NaCl (Fig. 3b). In the I/Br competition, a statistical mixture of products is formed (1 : 1 : 2 di-iodide **4** : di-bromide **18** : mixed **25**) – suggesting both reaction rates are comparable. On the other hand, an I/Cl competition provides greater selectivity. Only mono- and di-iodide products (**4**) are observed with visible light irradiation (since chlorination requires UV light); yet UV irradiation (which unproductively consumes iodinated species) exclusively affords chlorination (**19**). Lastly, we exploited the difference in halide reactivity to enable a synthetically useful, iterative C–H halogenation (Fig. 3c). In the sequence, mono C–H chlorination (**26**) and subsequent C–H iodination affords β geminal halide **27** that contains two different halides (Cl, I).

**Fig. 4:**
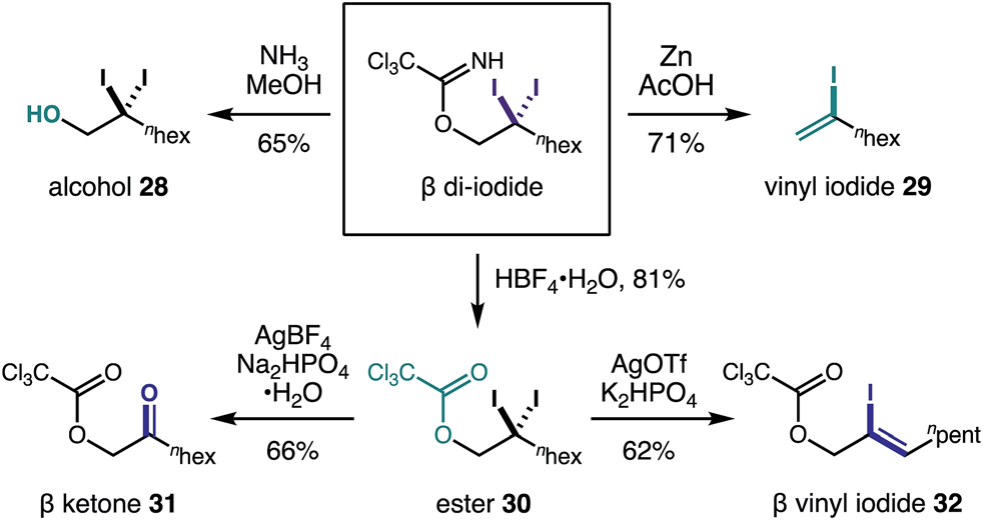
Synthetic versatility of the geminal β di-iodides.

Equipped with the first method to access β *gem*-di-halides *via* C–H functionalization, we sought to elucidate the synthetic utility of these versatile handles. Fig. 4 illustrates five post-synthetic transformations we investigated to further elaborate the β di-iodide imidates. First, aminolysis with NH_3_ affords β di-iodo-alcohol **28**. Alternatively, reduction of one of the iodides by Zn in AcOH affords vinyl iodide **29***via* imidate elimination. Otherwise, imidate hydrolysis to ester **30** occurs under acidic conditions (HBF_4_·H_2_O), leaving the di-iodide intact. From the β di-iodo-ester, hydrolysis to α-oxy ketone **31** is possible (AgBF_4_, Na_2_HPO_4_·H_2_O); or conversion to allyl alcohol **32**, bearing a vinyl iodide, is realized *via* addition of AgOTf and K_2_HPO_4_.

## Conclusions

In summary, a radical relay strategy has enabled the one-step conversion of imidates to mono- or di-halides *via* iterative β C–H halogenation. In particular, synthetic access to the versatile, geminal di-halides is uniquely facilitated by an imidate radical-based 1,5-HAT mechanism. By developing a new strategy to bypass oxidative decomposition pathways, reactive alkyl halide intermediates of a radical relay reaction mechanism were intercepted. Along with new methods for mono- and di-C–H halogenation (X = I, Br, Cl), competitive rates and kinetic profiles have also been investigated. Finally, the versatility of the β di-iodides is showcased in the synthesis of functionally rich molecules – uniquely enabled by an HAT-based β C–H functionalization mechanism.

## Conflicts of interest

There are no conflicts to declare.

## Supplementary Material

Supplementary information

Supplementary information

Crystal structure data

## References

[cit1] Liu W., Groves J. T. (2015). Acc. Chem. Res..

[cit2] The Chemistry of Halides Pseudo-Halides and Azides, ed. S. Patai and Z. Rappoport, John Wiley & Sons, Ltd., Chichester, UK, 2010.

[cit3] Breslow R. (1980). Acc. Chem. Res..

[cit4] Lyons T. W., Sanford M. S. (2010). Chem. Rev..

[cit5] Carr K., Sutherland J. K. (1984). J. Chem. Soc., Chem. Commun..

[cit6] Giri R., Chen X., Yu J.-Q. (2005). Angew. Chem., Int. Ed..

[cit7] Giri R., Wasa M., Breazzano S. P., Yu J.-Q. (2006). Org. Lett..

[cit8] Tanner D. D., Gidley G. C. (1968). J. Am. Chem. Soc..

[cit9] Wang Y.-F., Chen H., Zhu X., Chiba S. (2012). J. Am. Chem. Soc..

[cit10] Liu W., Groves J. T. (2010). J. Am. Chem. Soc..

[cit11] Walling C., Padwa A. (1963). J. Am. Chem. Soc..

[cit12] Majetich G., Wheless K. (1995). Tetrahedron.

[cit13] Dorta R. L., Francisco C. G., Suárez E. (1989). J. Chem. Soc., Chem. Commun..

[cit14] Katohgi M., Togo H., Yamaguchi K., Yokoyama M. (1999). Tetrahedron.

[cit15] Tozer M. J., Herpin T. F. (1996). Tetrahedron.

[cit16] Barton D. H. R., O'Brien R. E., Sternhell S. (1962). J. Chem. Soc..

[cit17] Wappes E. A., Nakafuku K. M., Nagib D. A. (2017). J. Am. Chem. Soc..

[cit18] Wappes E. A., Fosu S. C., Chopko T. C., Nagib D. A. (2016). Angew. Chem., Int. Ed..

[cit19] Martínez C., Muńiz K. (2015). Angew. Chem., Int. Ed..

[cit20] Luo Y. R., Comprehensive Handbook of Chemical Bond Energies, Taylor & Francis, Boca Raton, FL, 2010.

[cit21] The increased electrophilicity of Cl (*vs.* I or Br) may polarize the C–H to such an extent that it is no longer polarity-matched with the imidate radical. For example, see: Roberts B. P., Chem. Soc. Rev., 1999, 28 , 25 .

[cit22] CCDC 1581032 contains the supplementary crystallographic data for this paper.

