# Supplementary figures and images for: β C–H di-halogenation via iterative hydrogen atom transfer
Source: Chem Sci. 2018 Apr 30;9(19):4500–4. doi: 10.1039/c8sc01214h (PMC5958344; doi:10.1039/c8sc01214h)

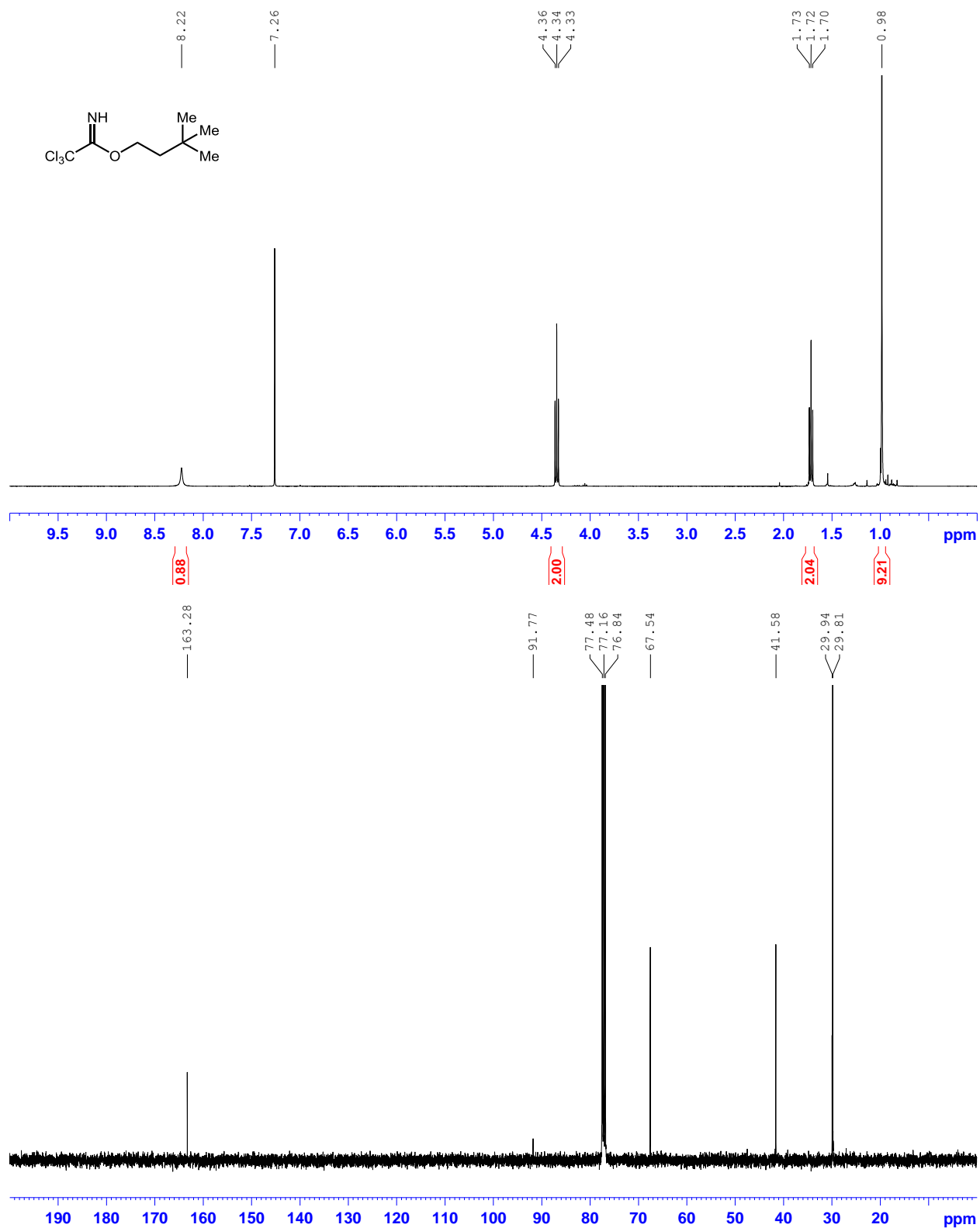

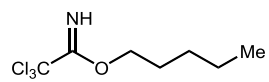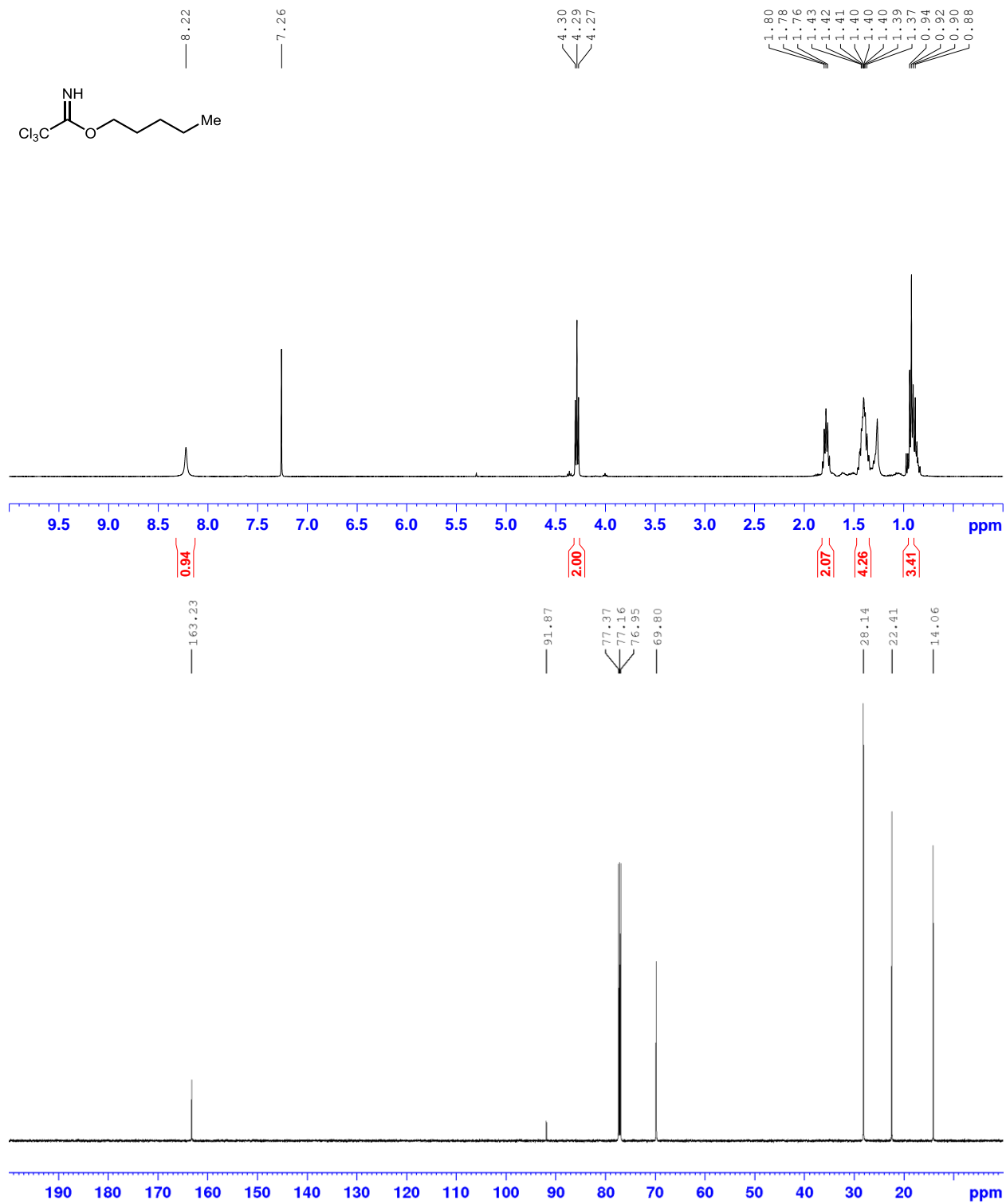

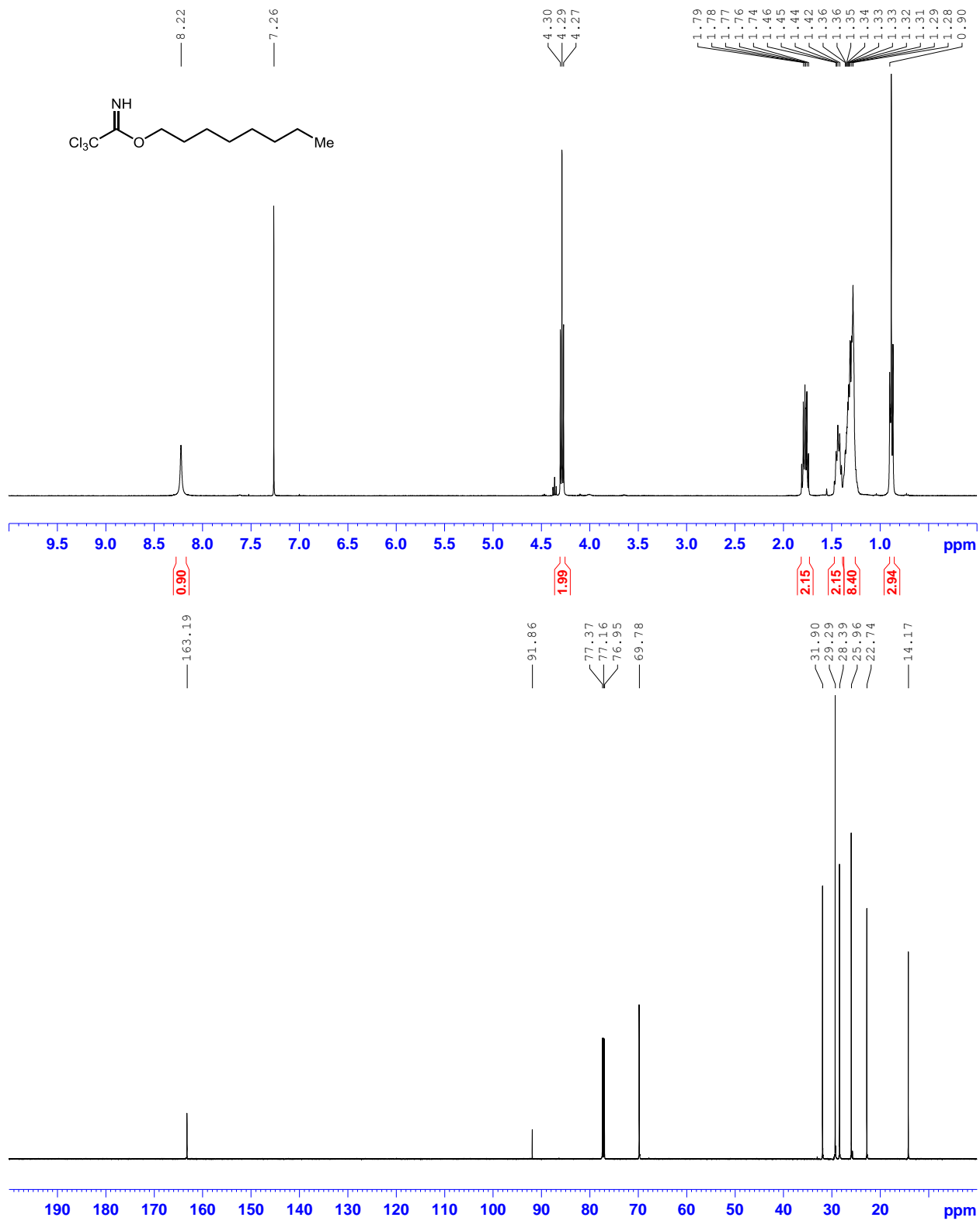

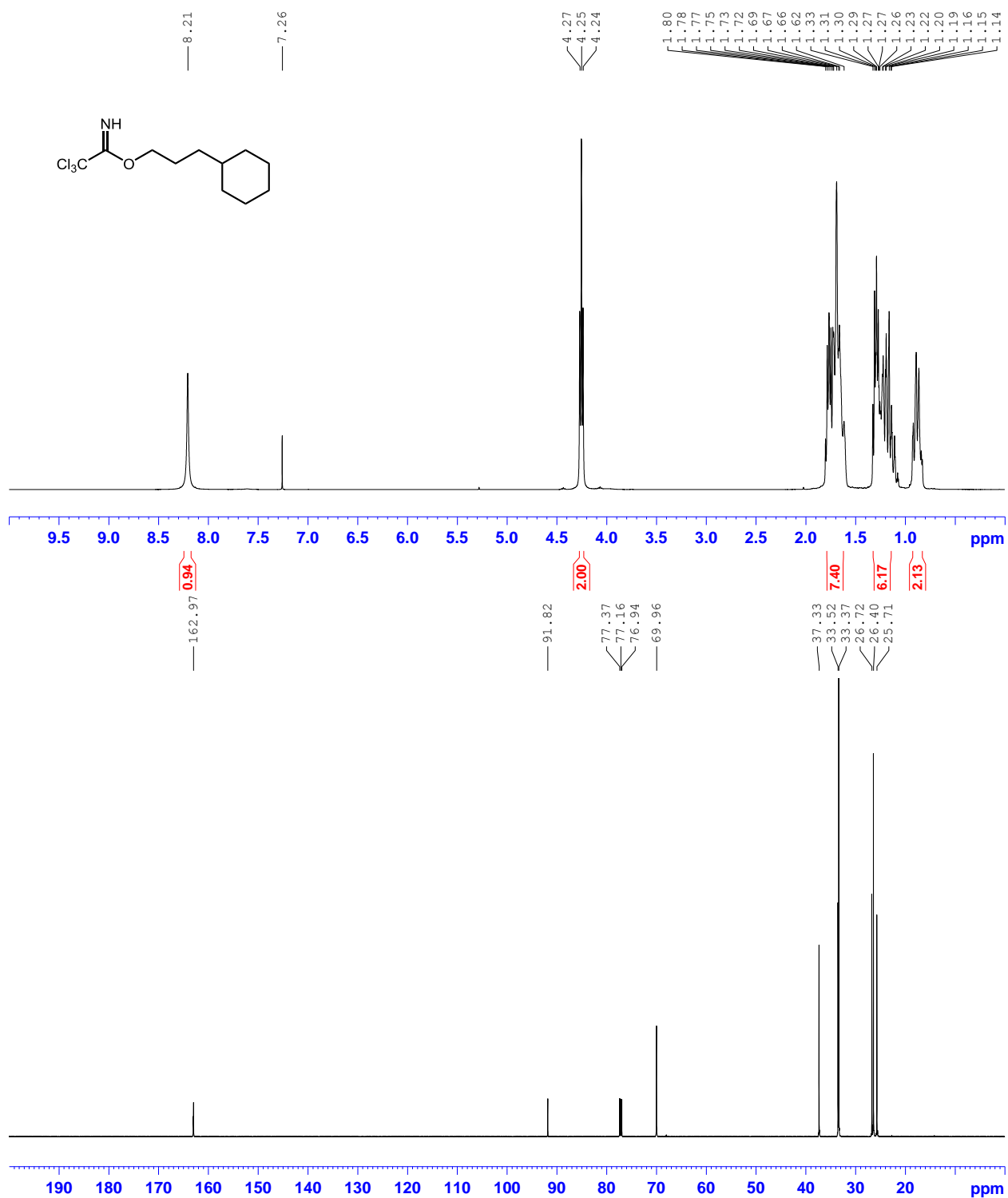

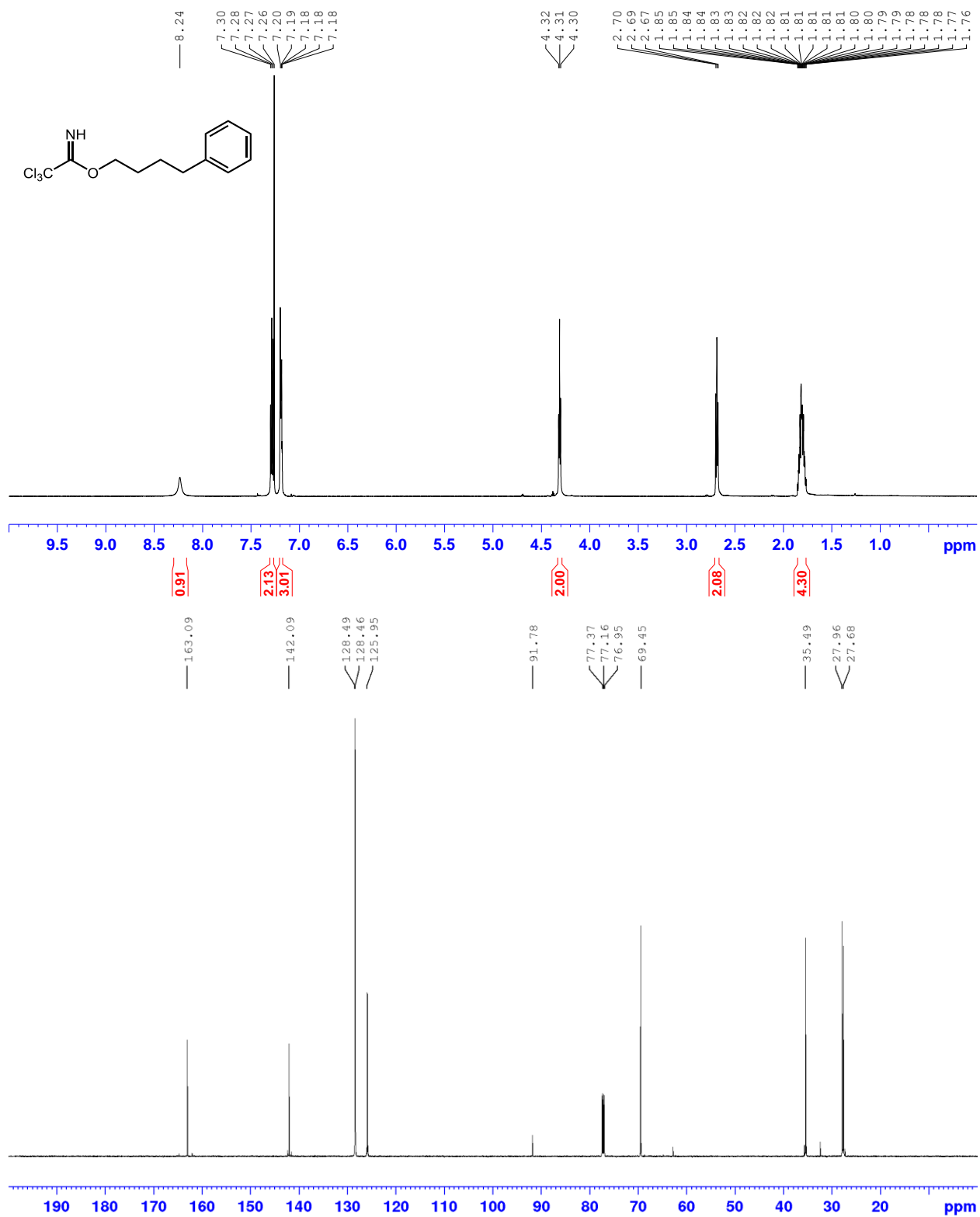

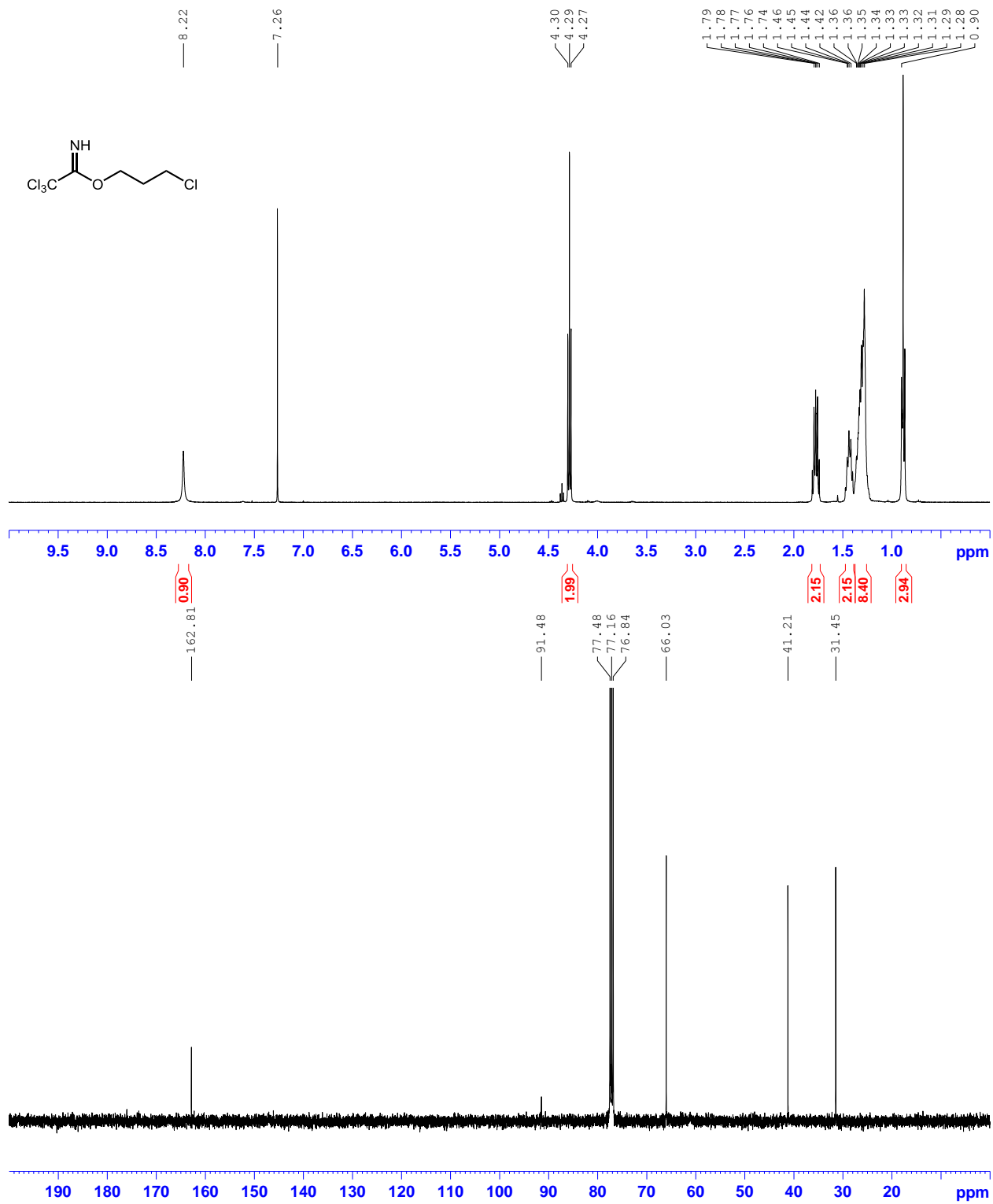

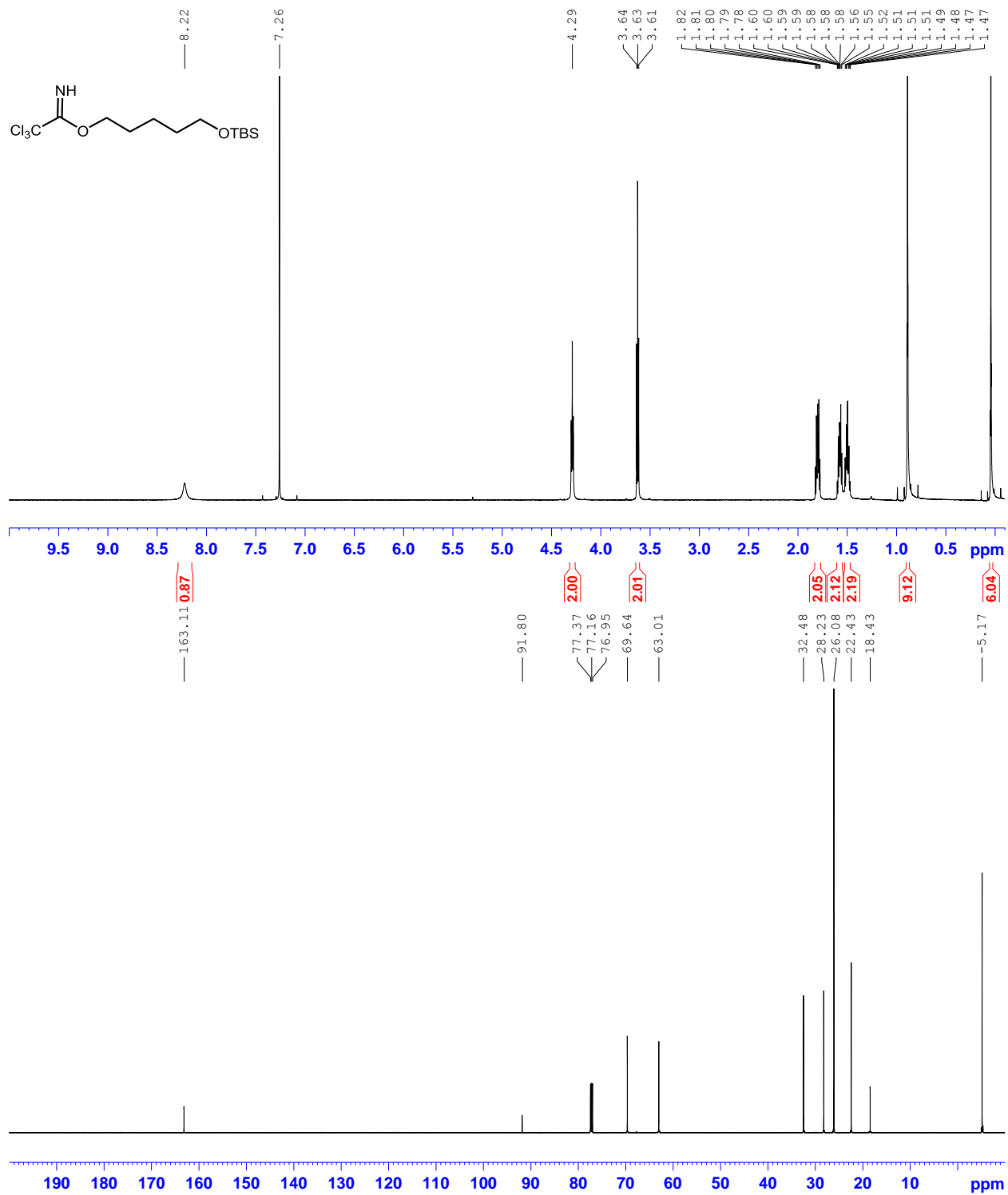

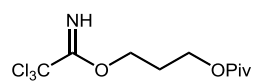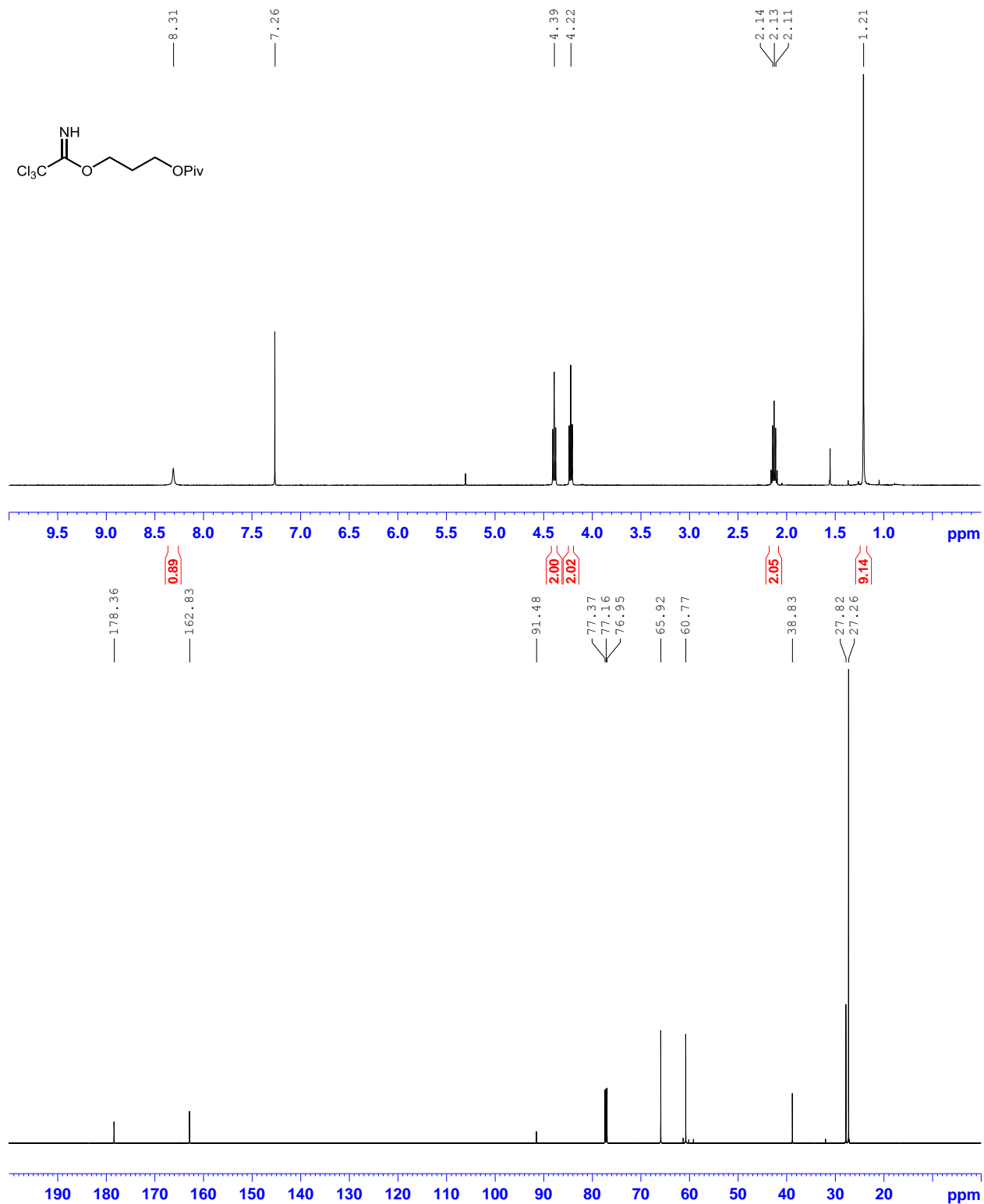

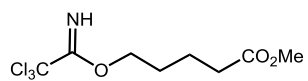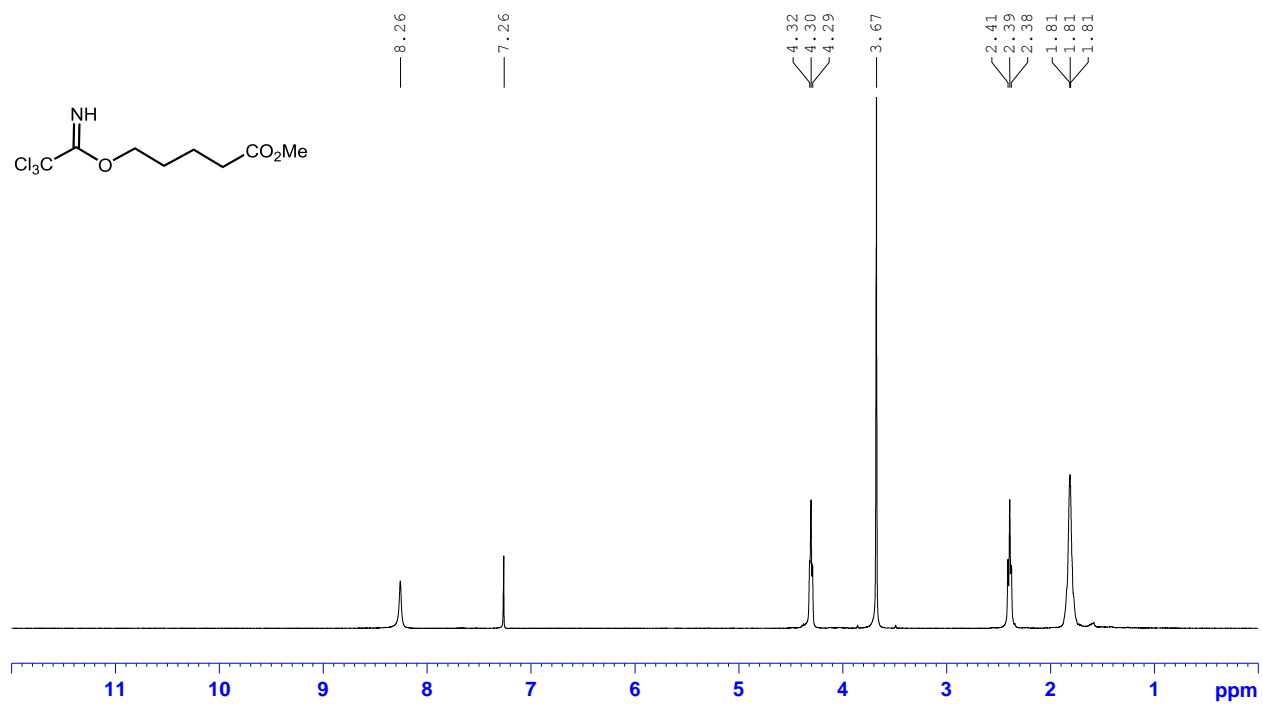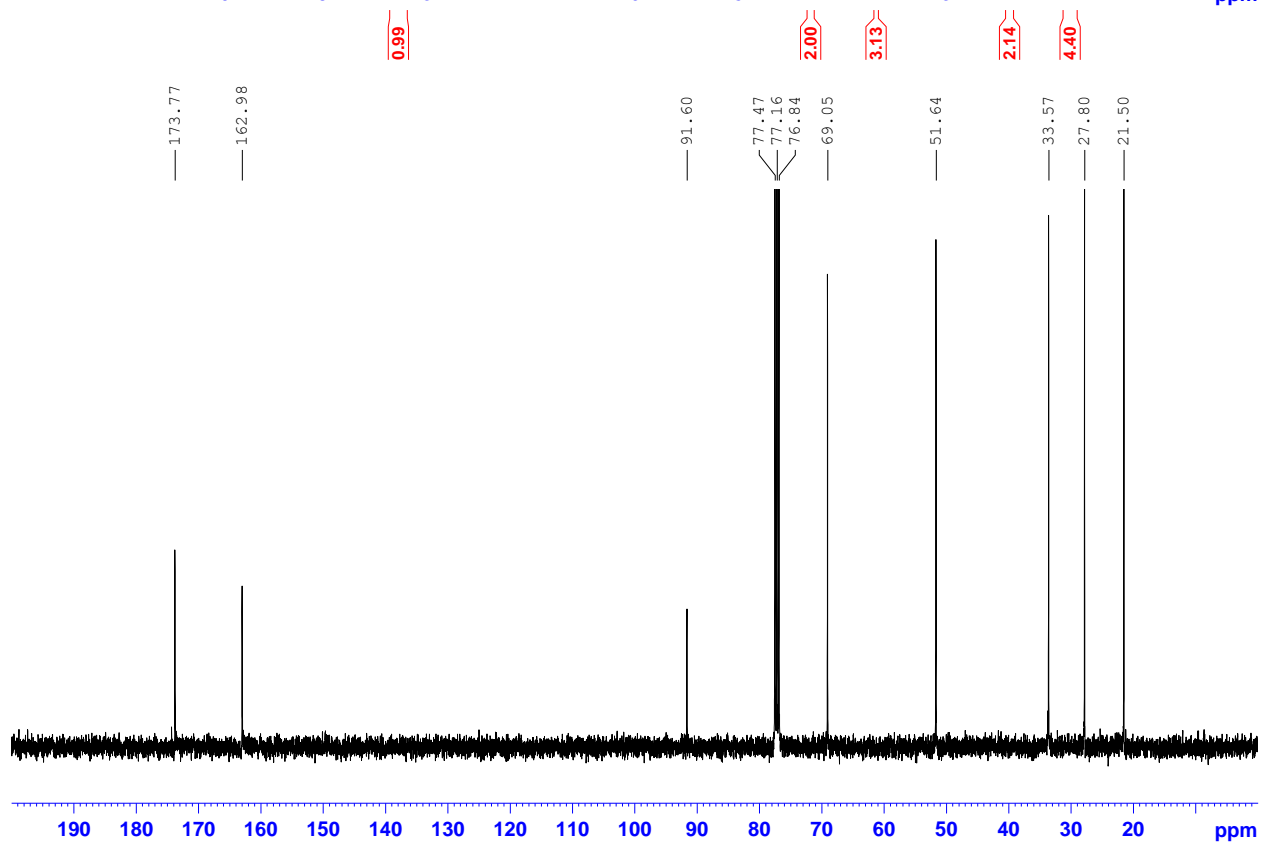

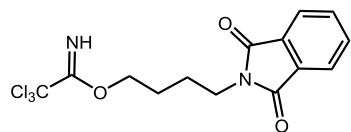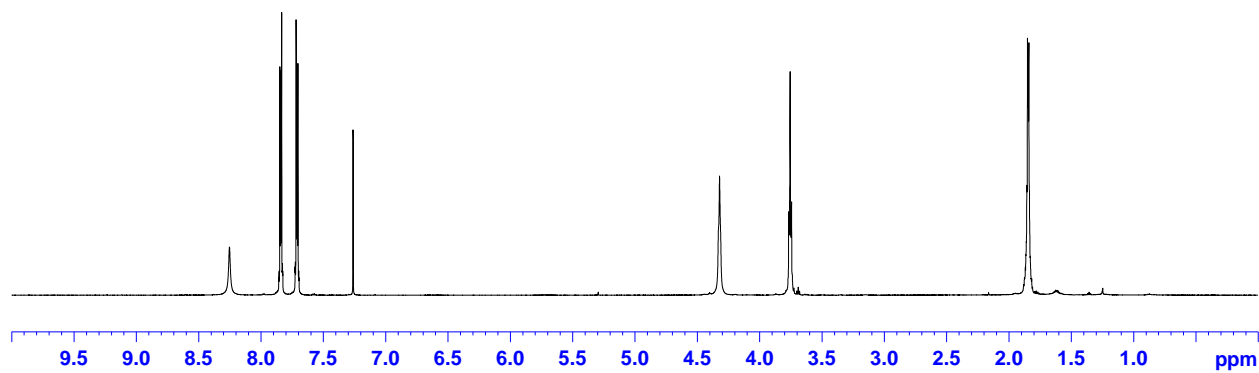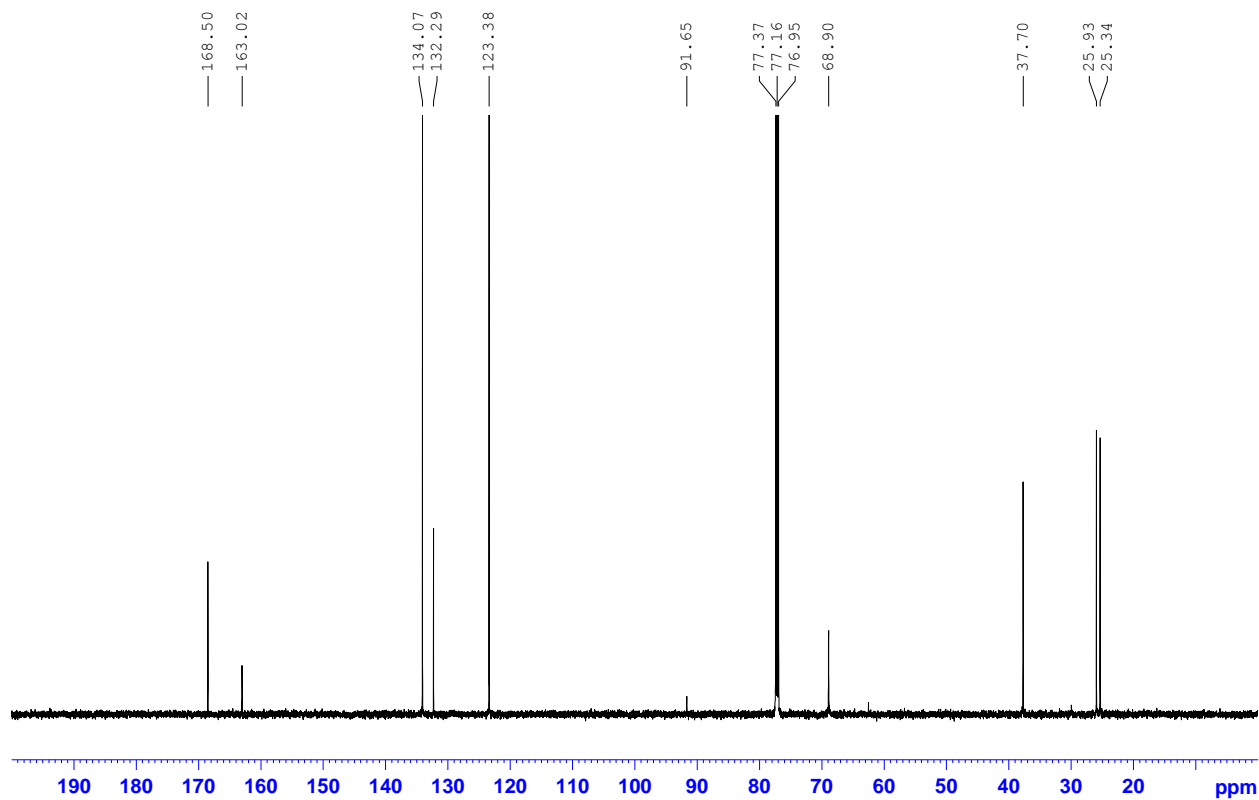

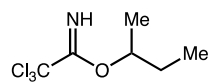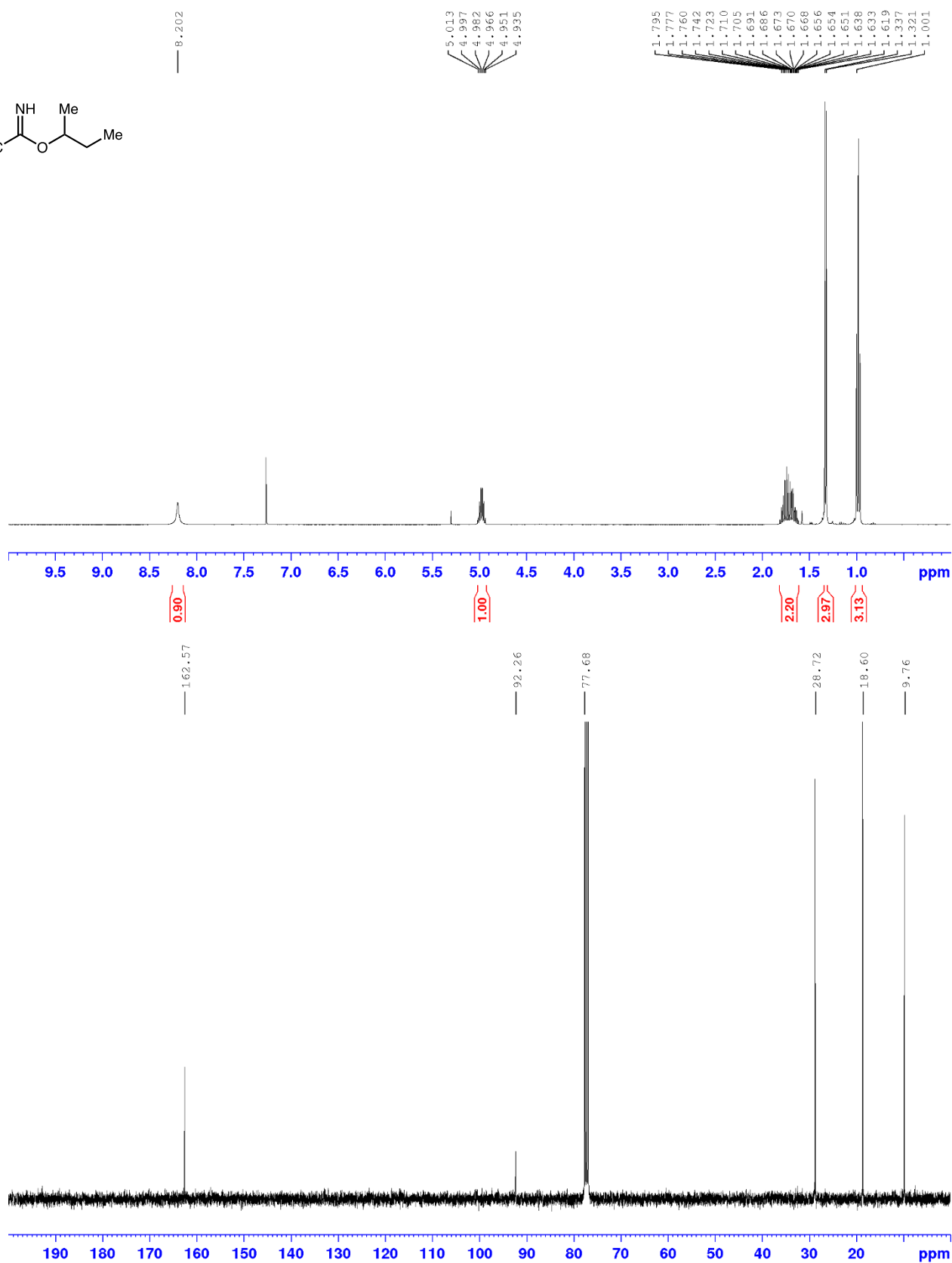

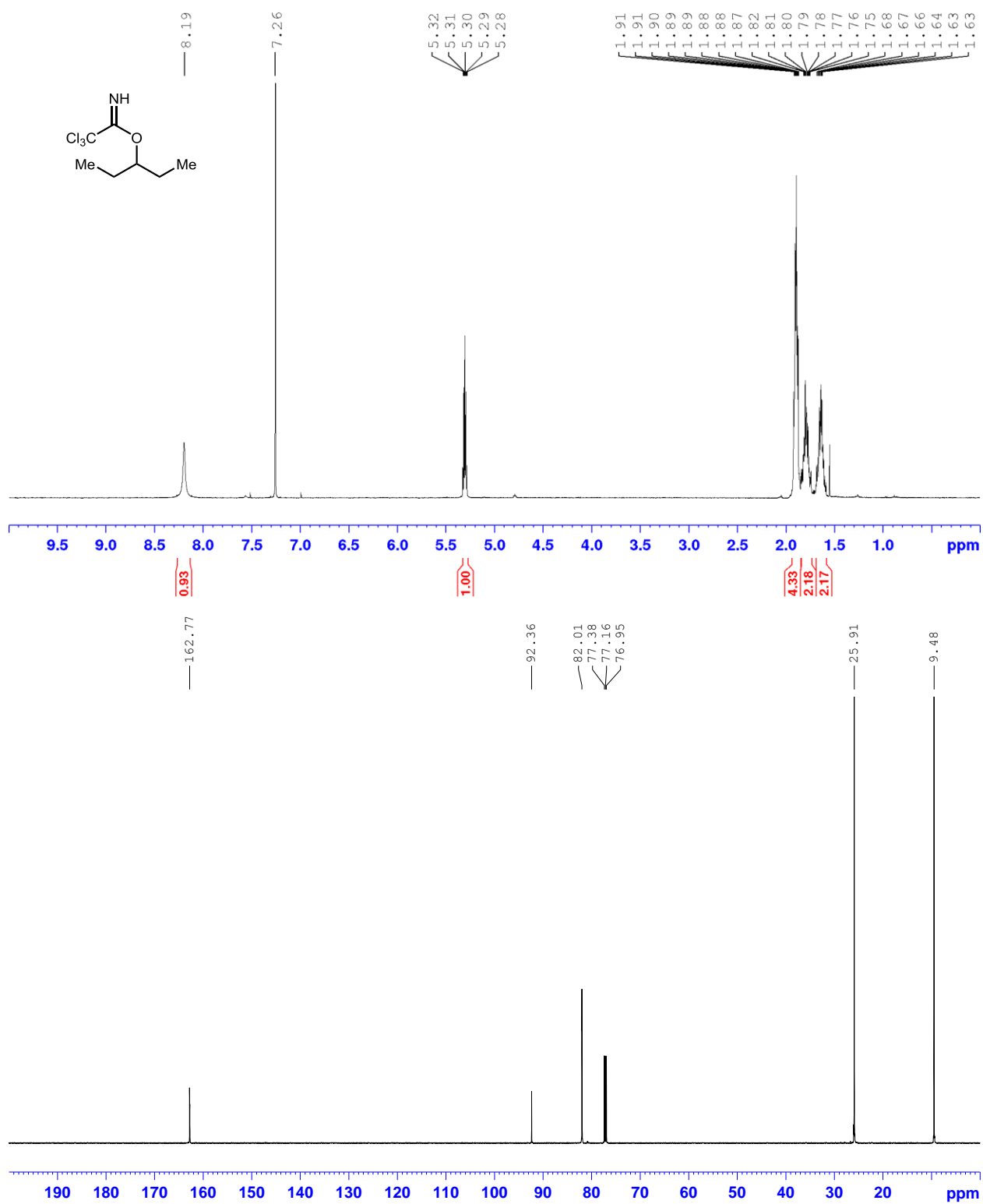

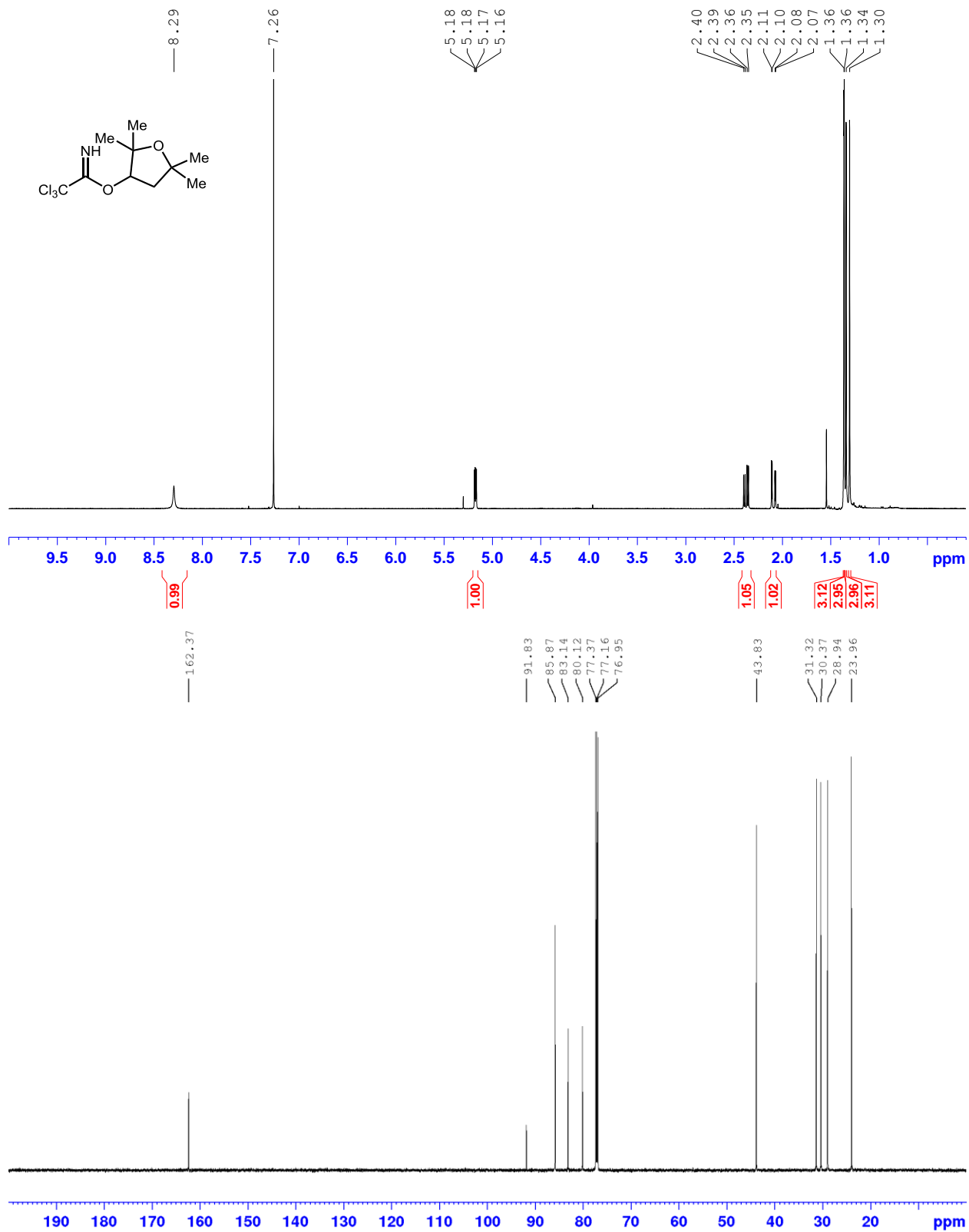

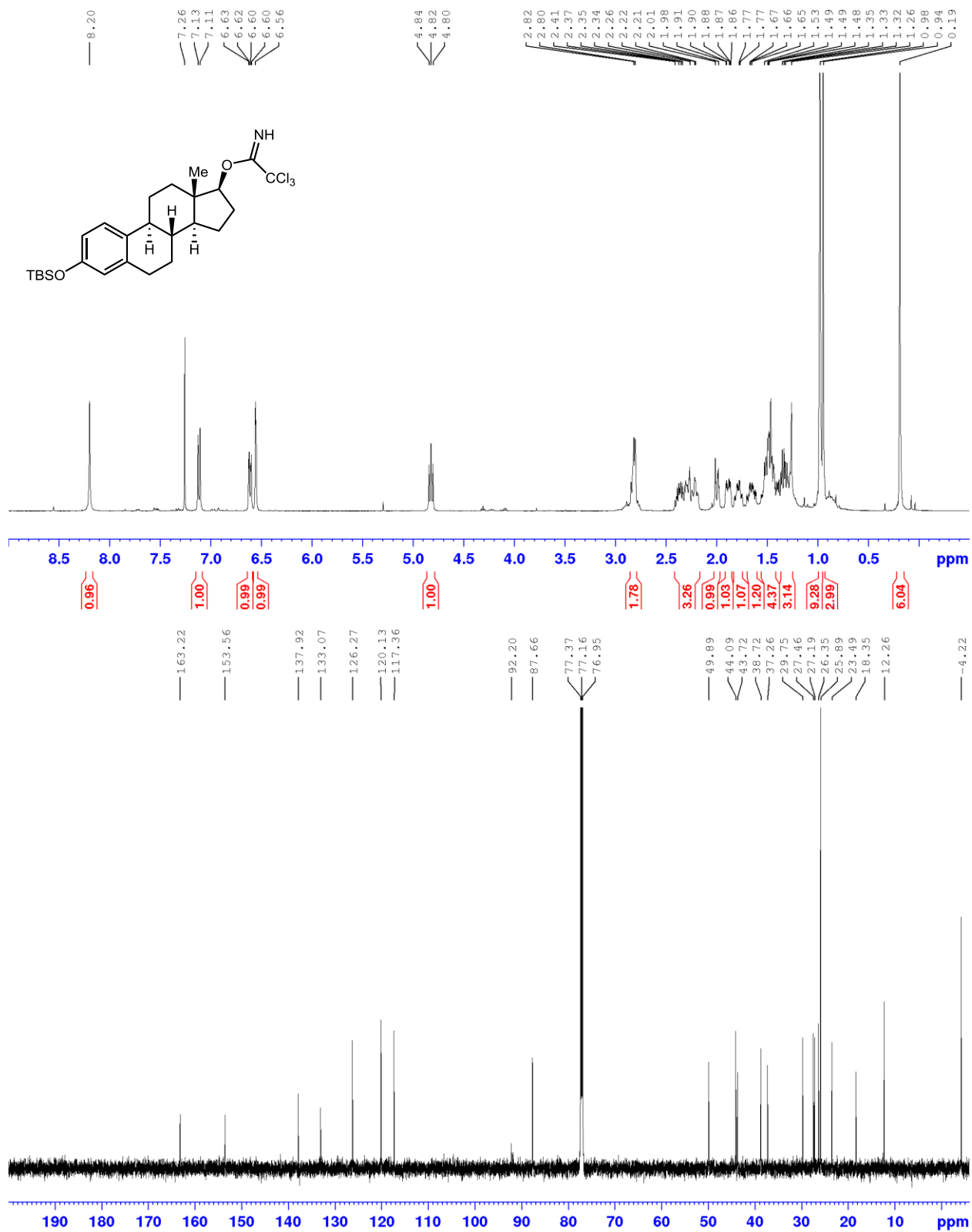

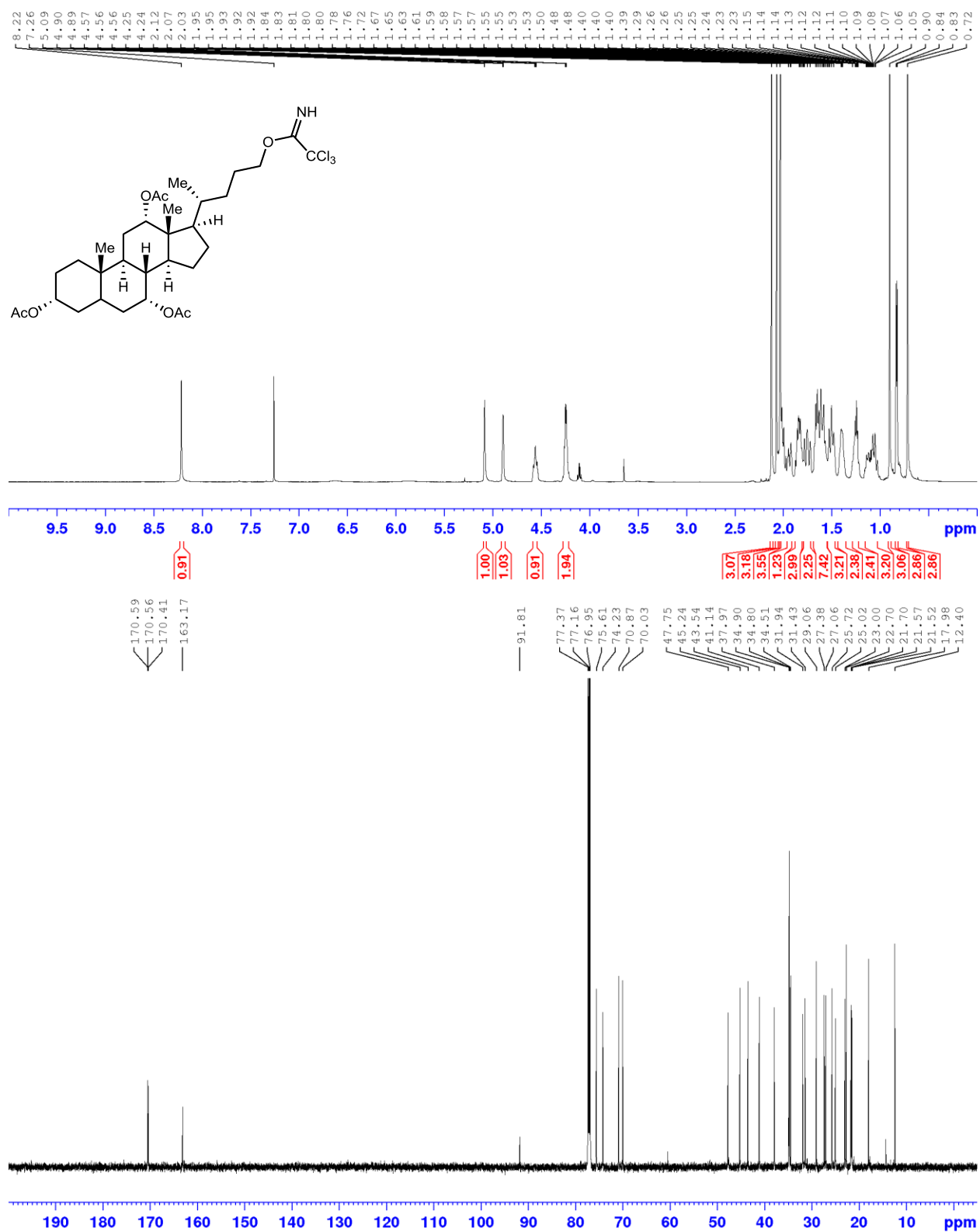

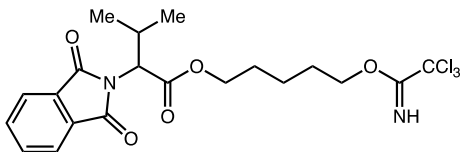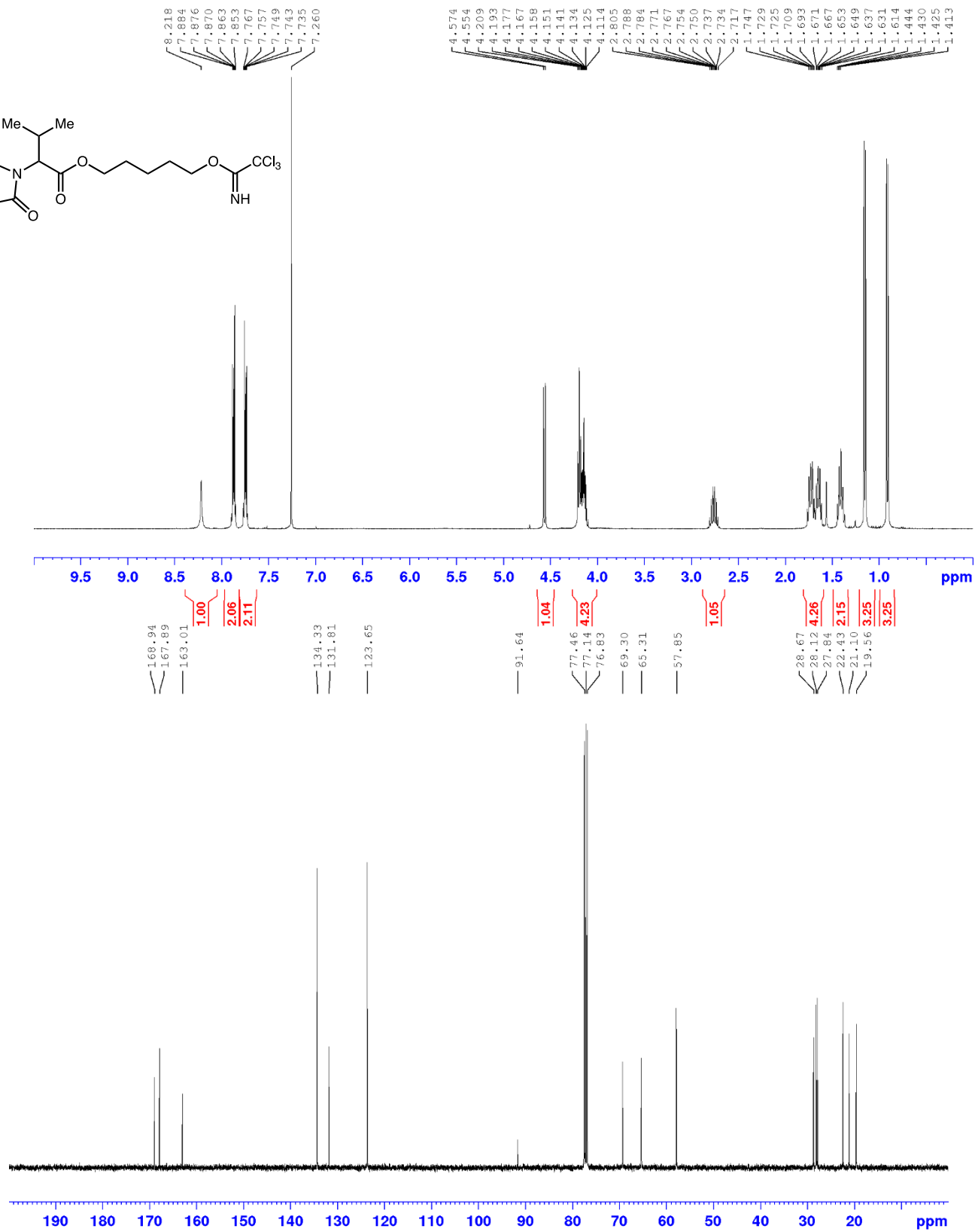

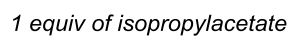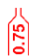

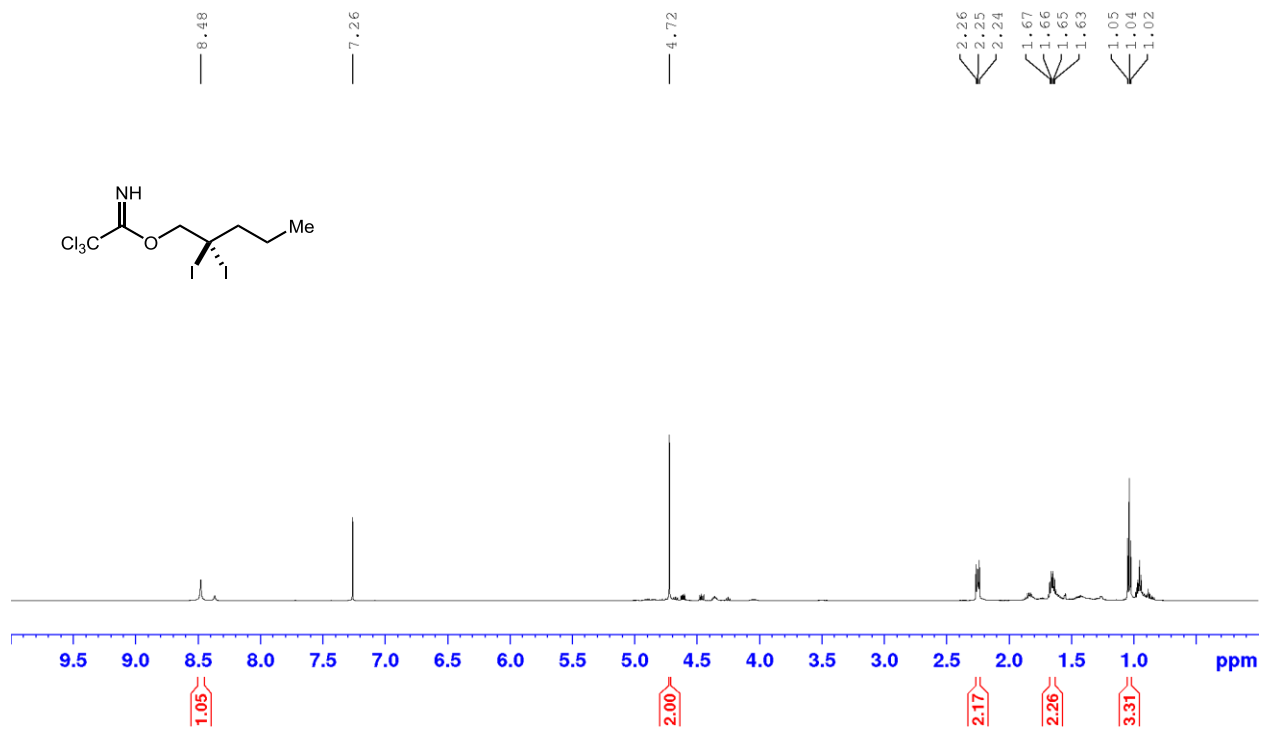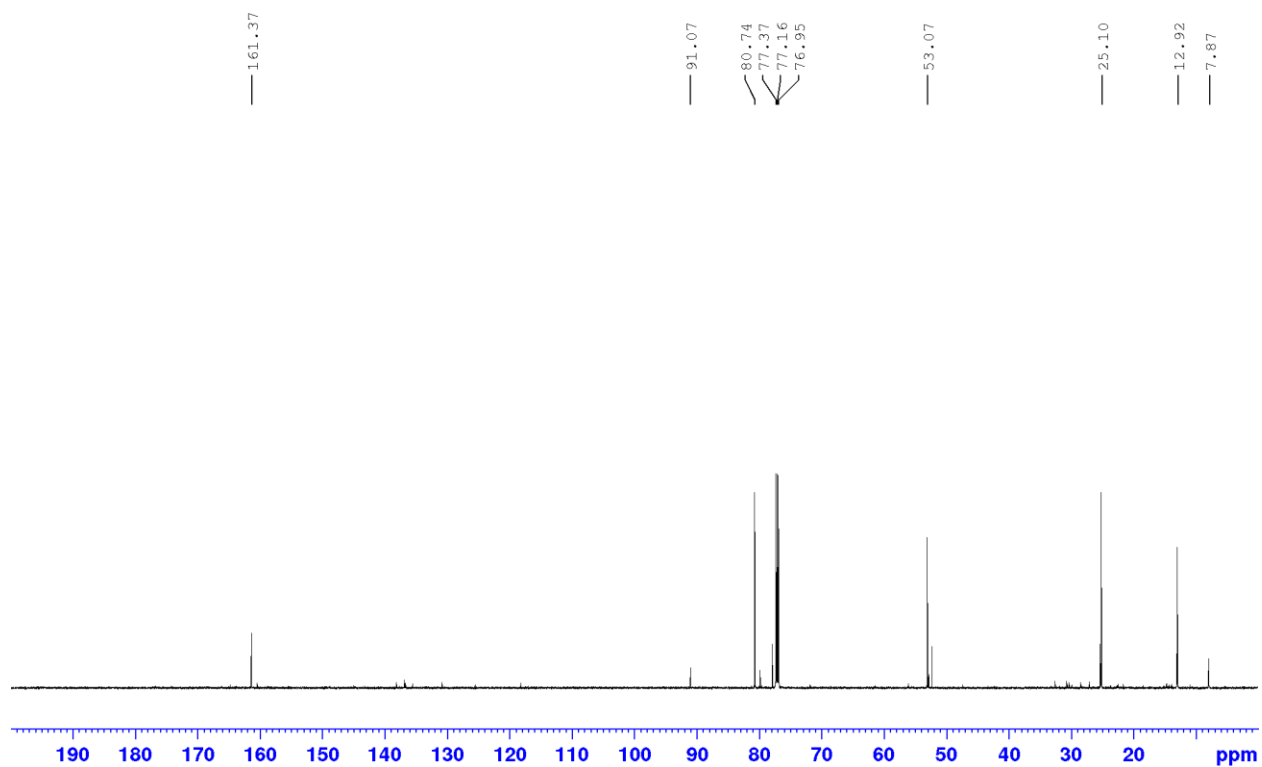

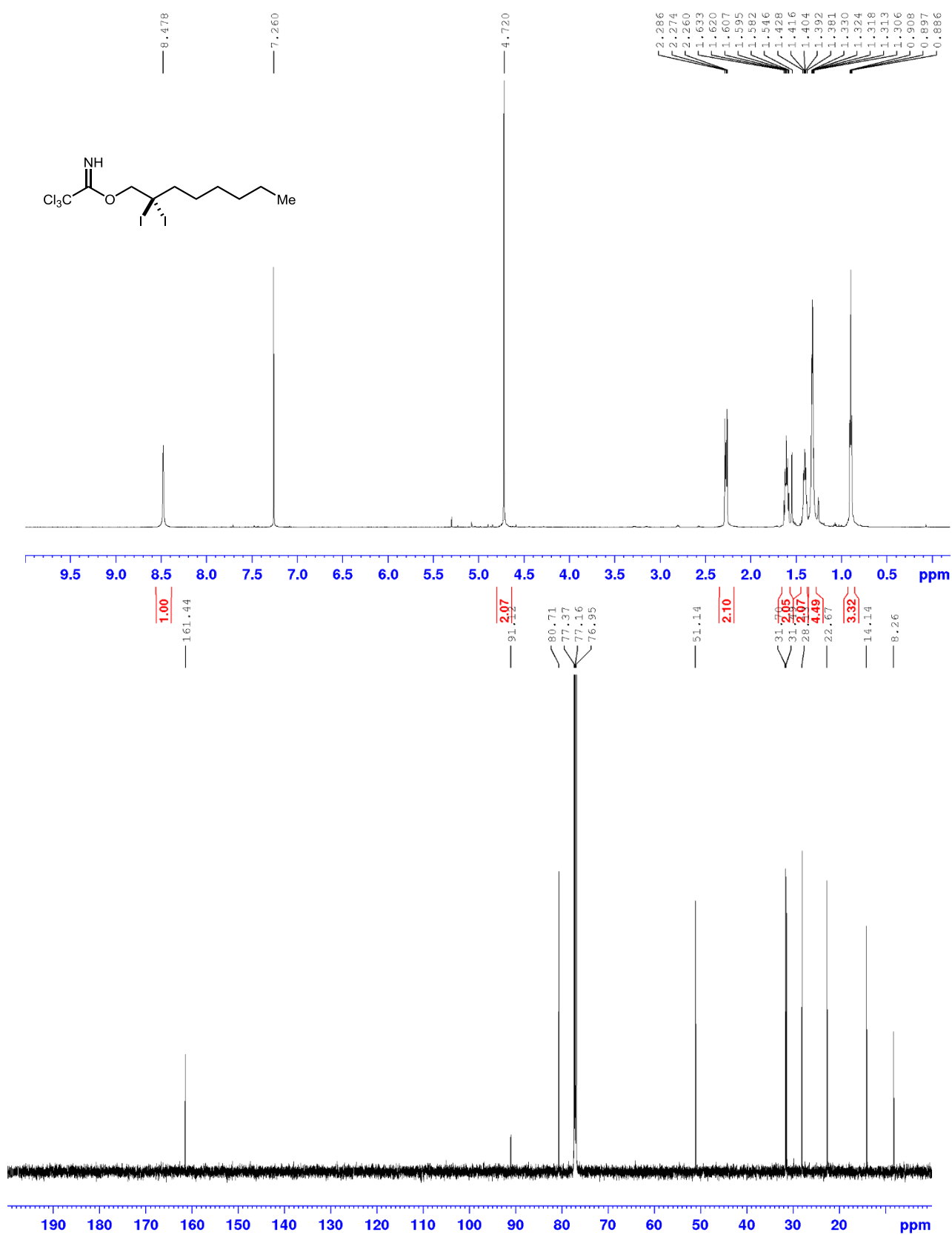

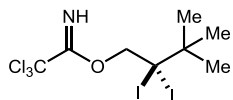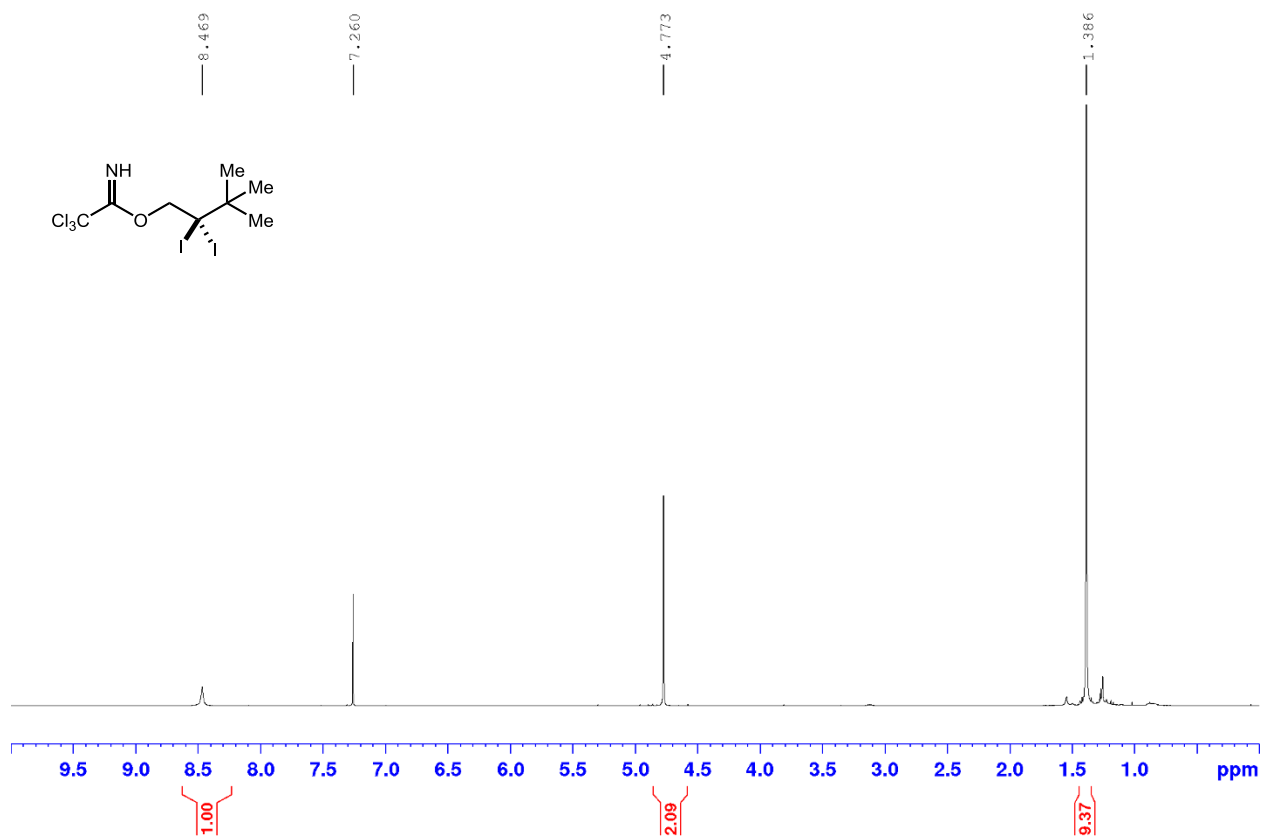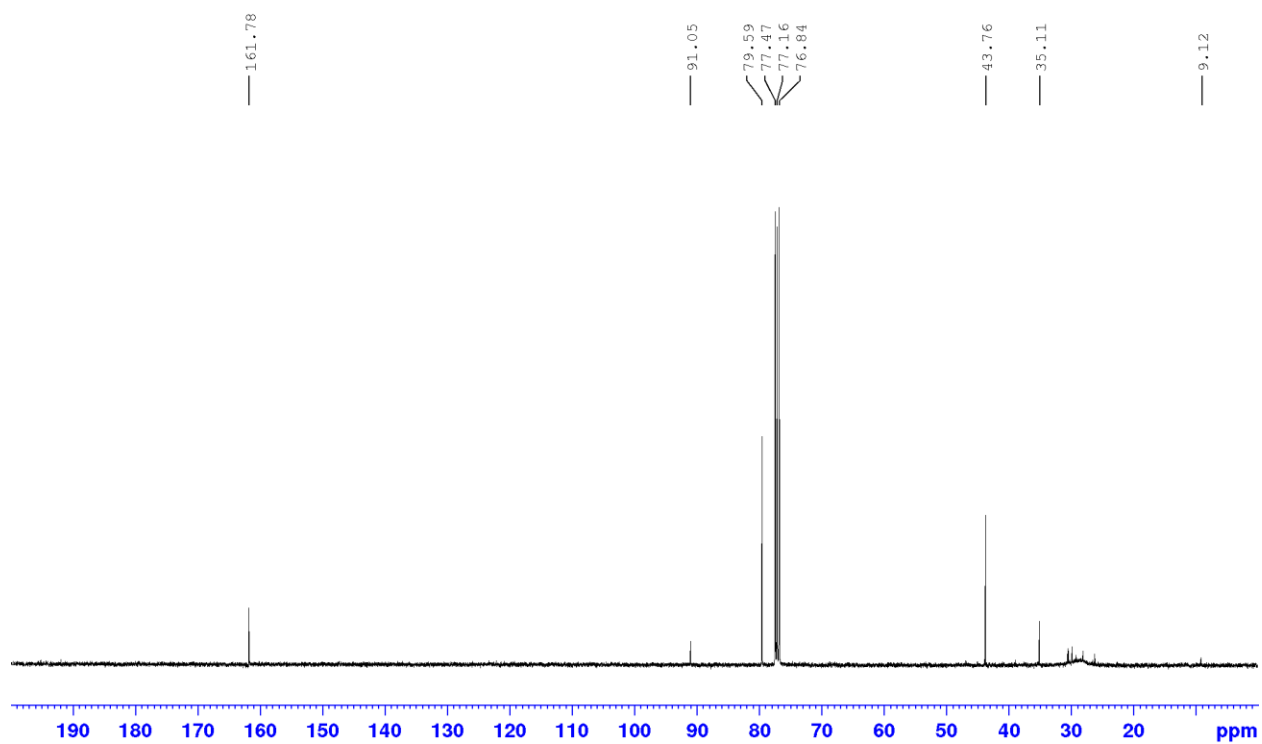

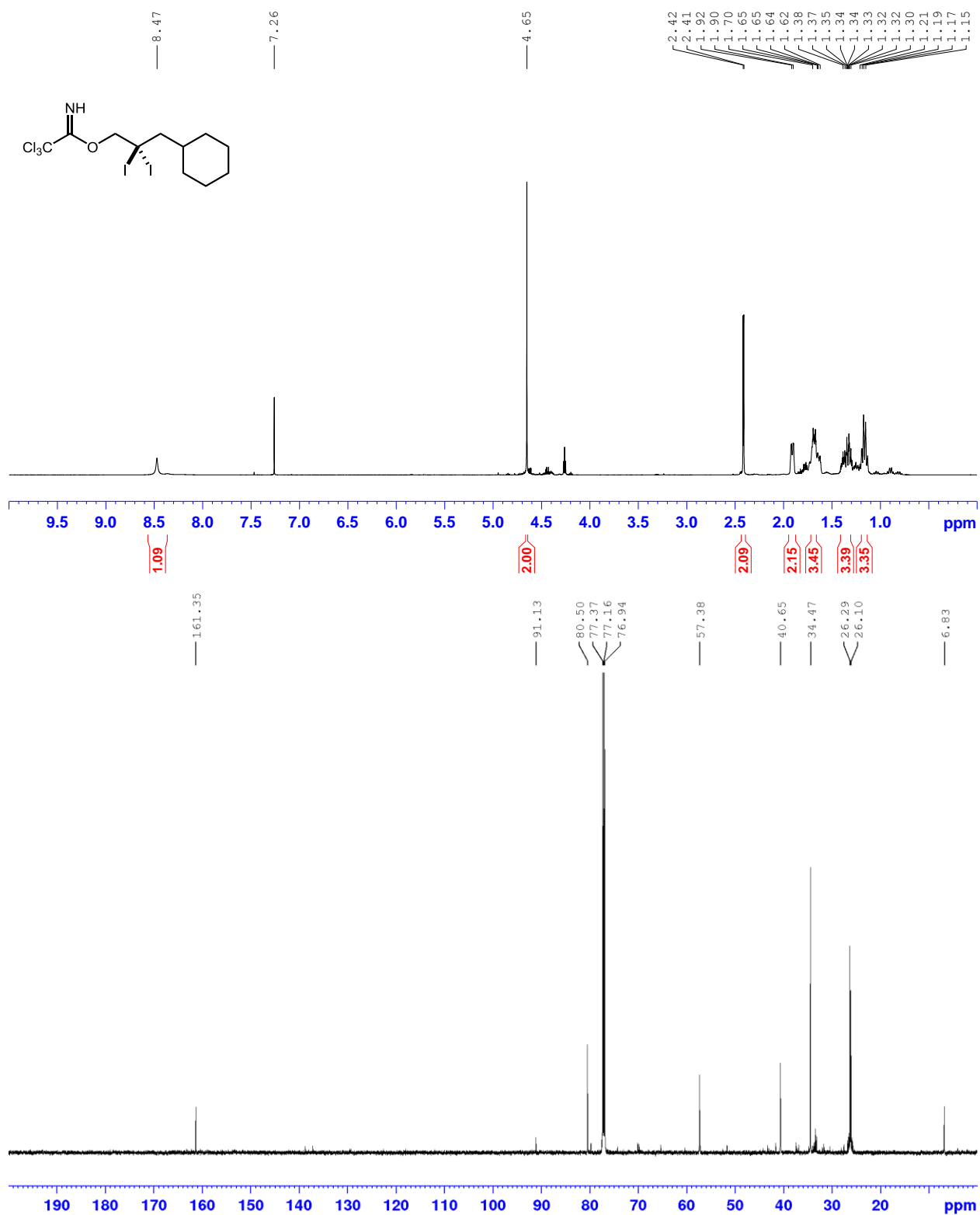

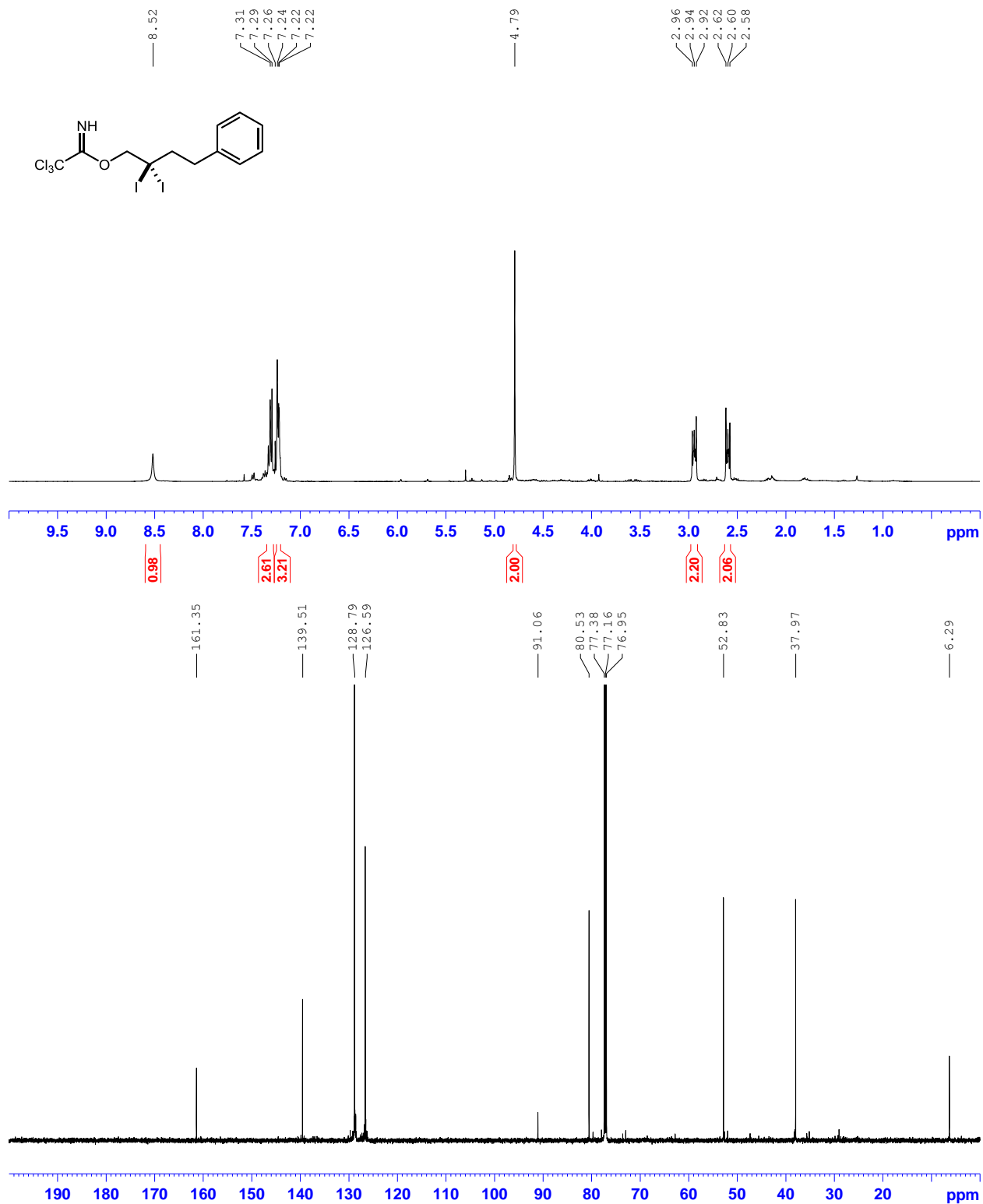

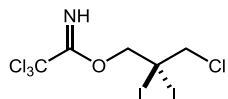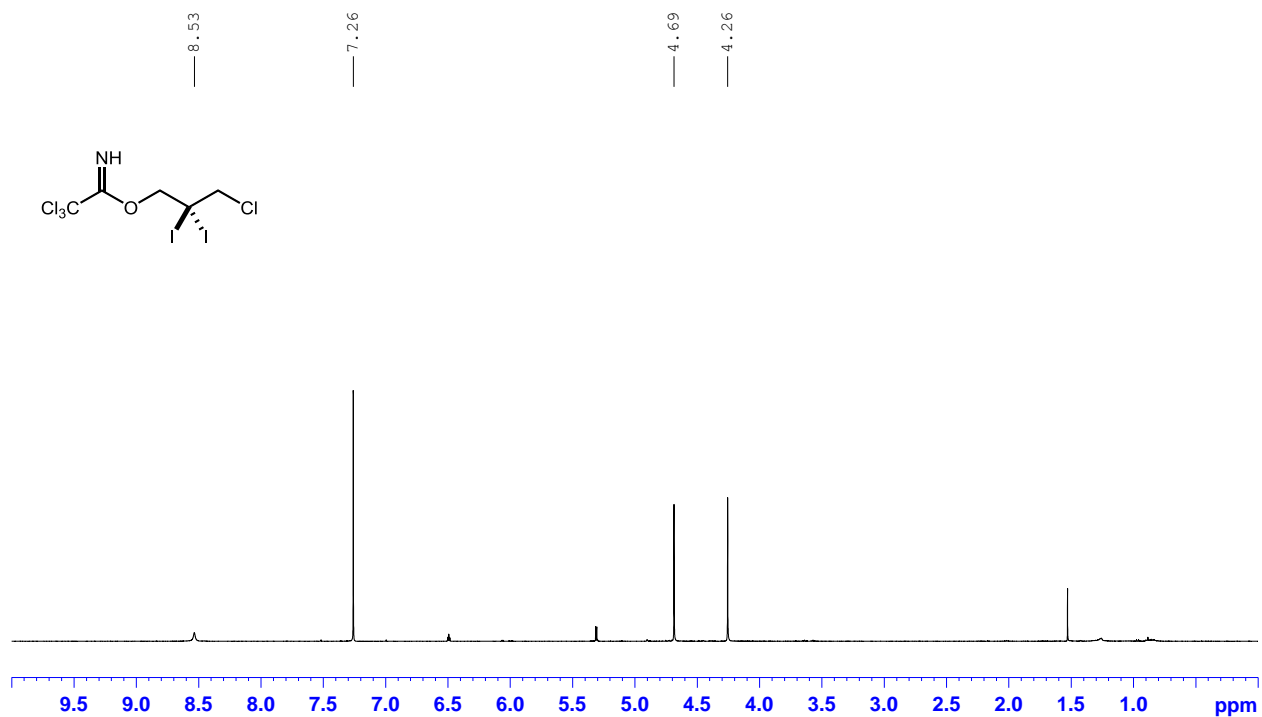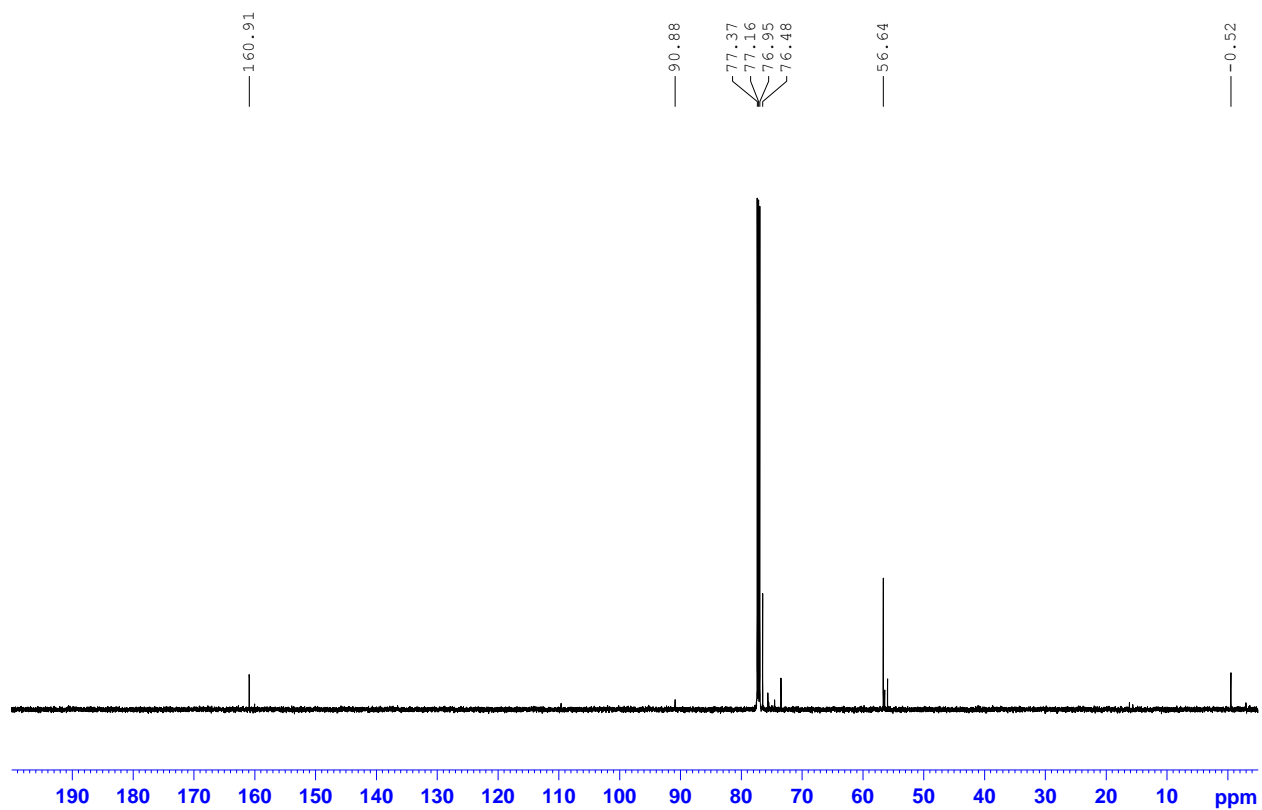

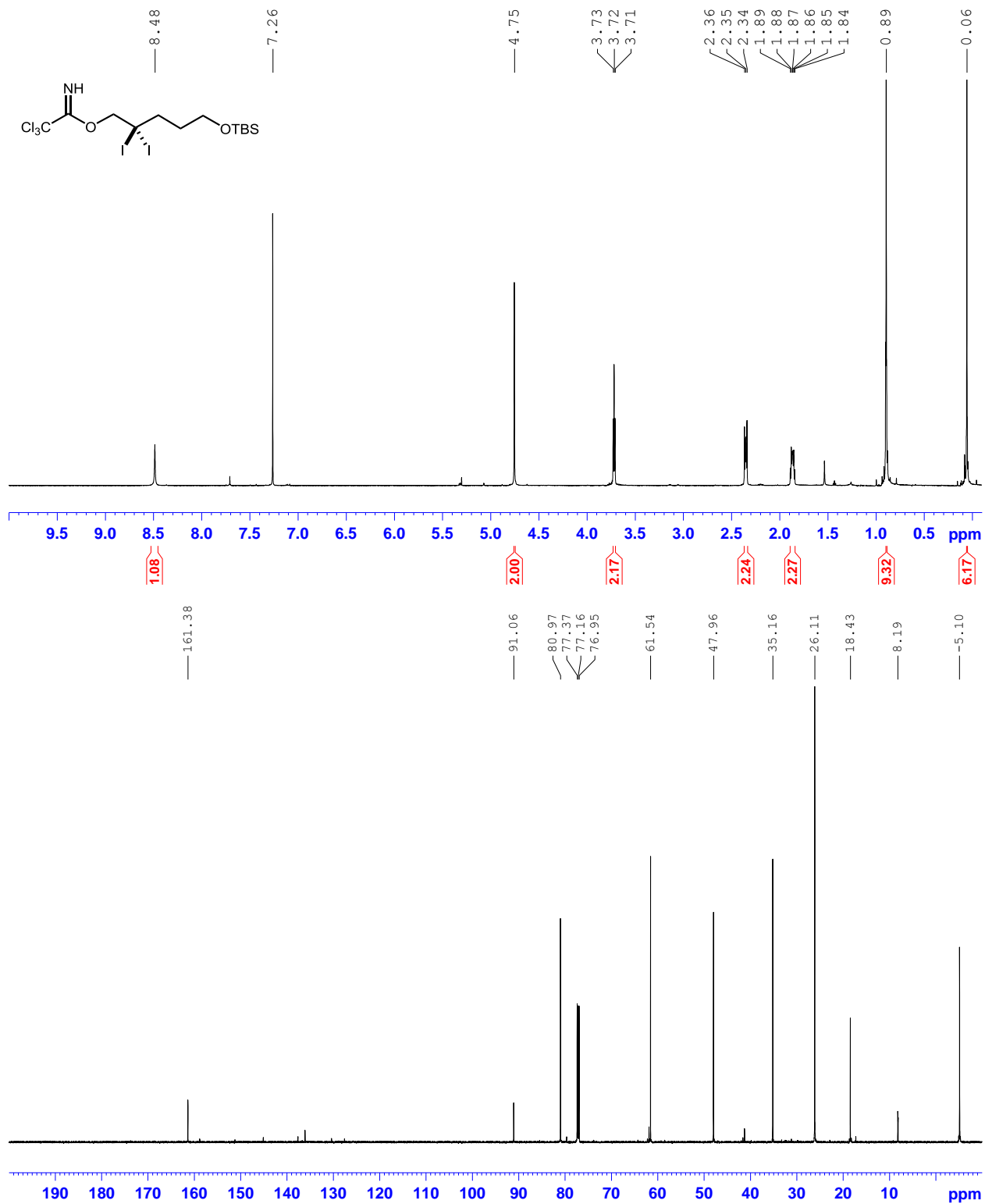

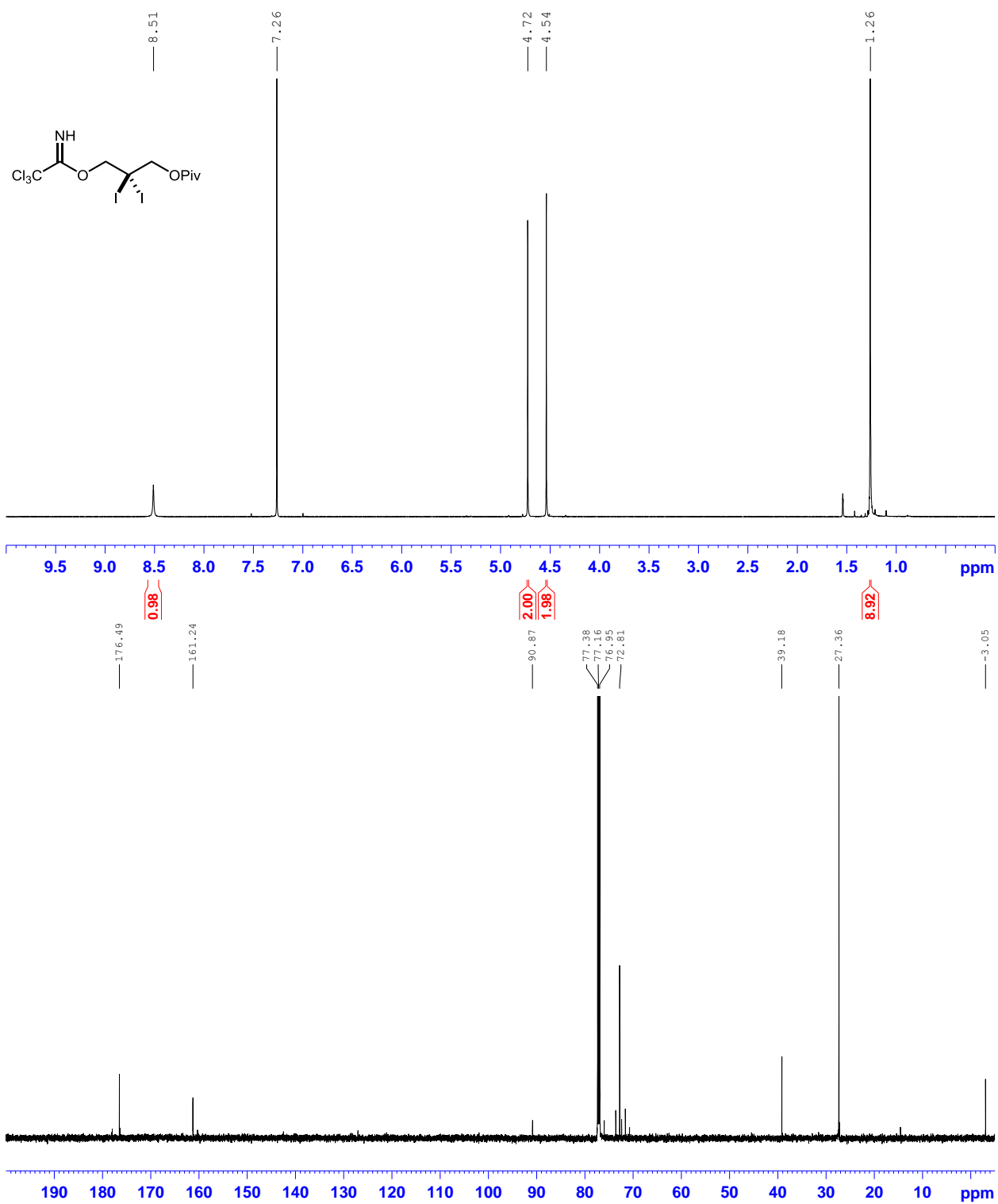

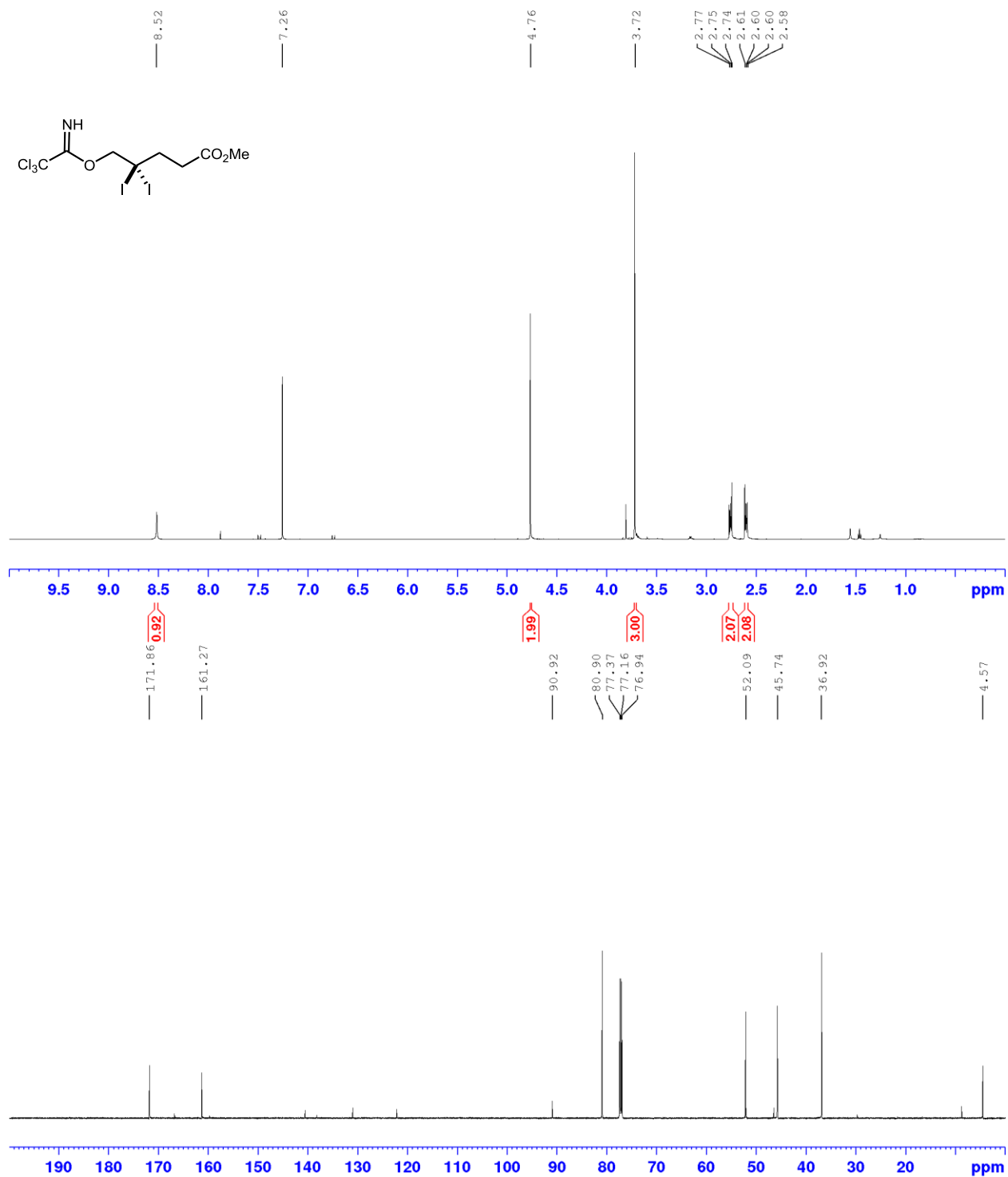

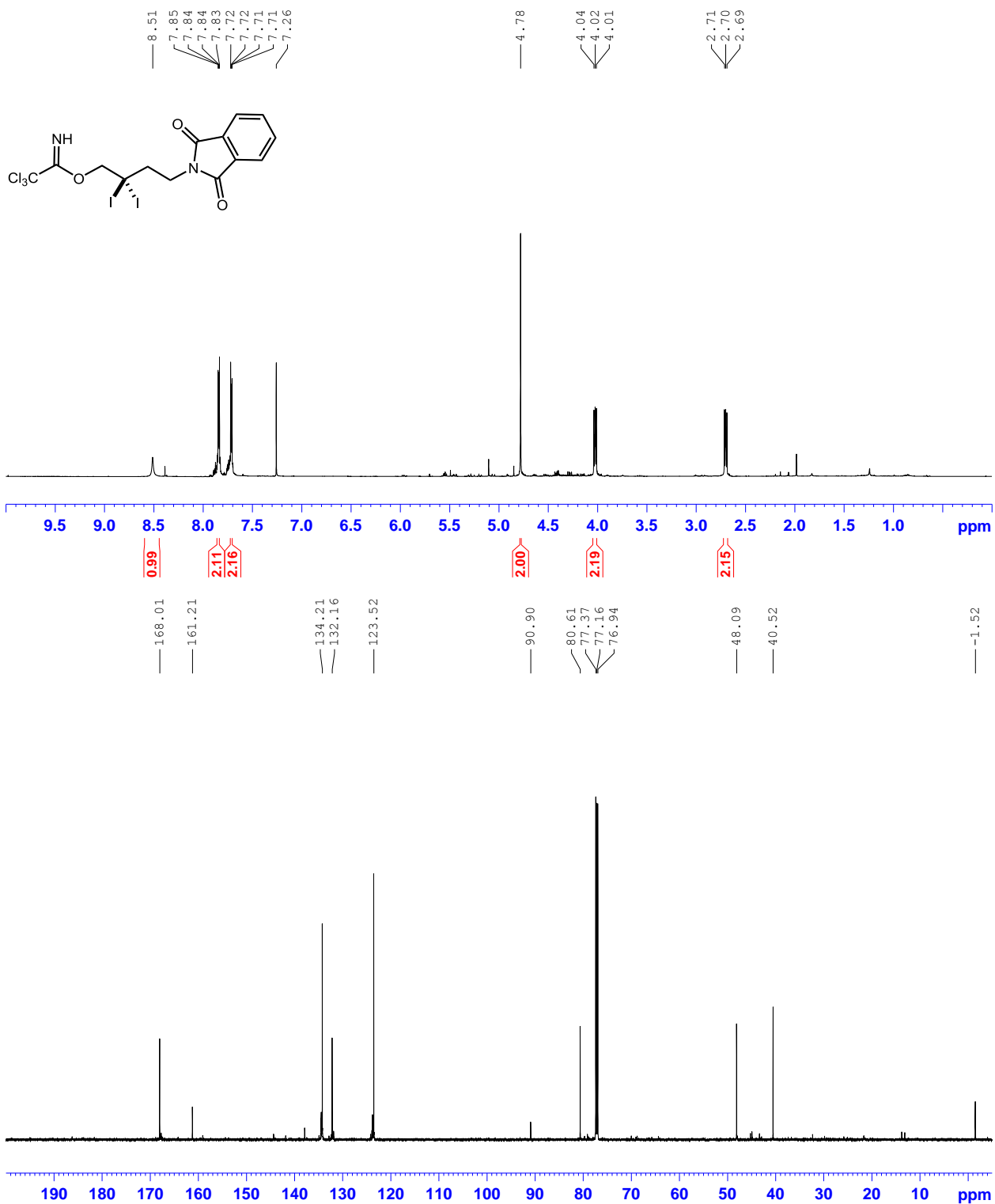

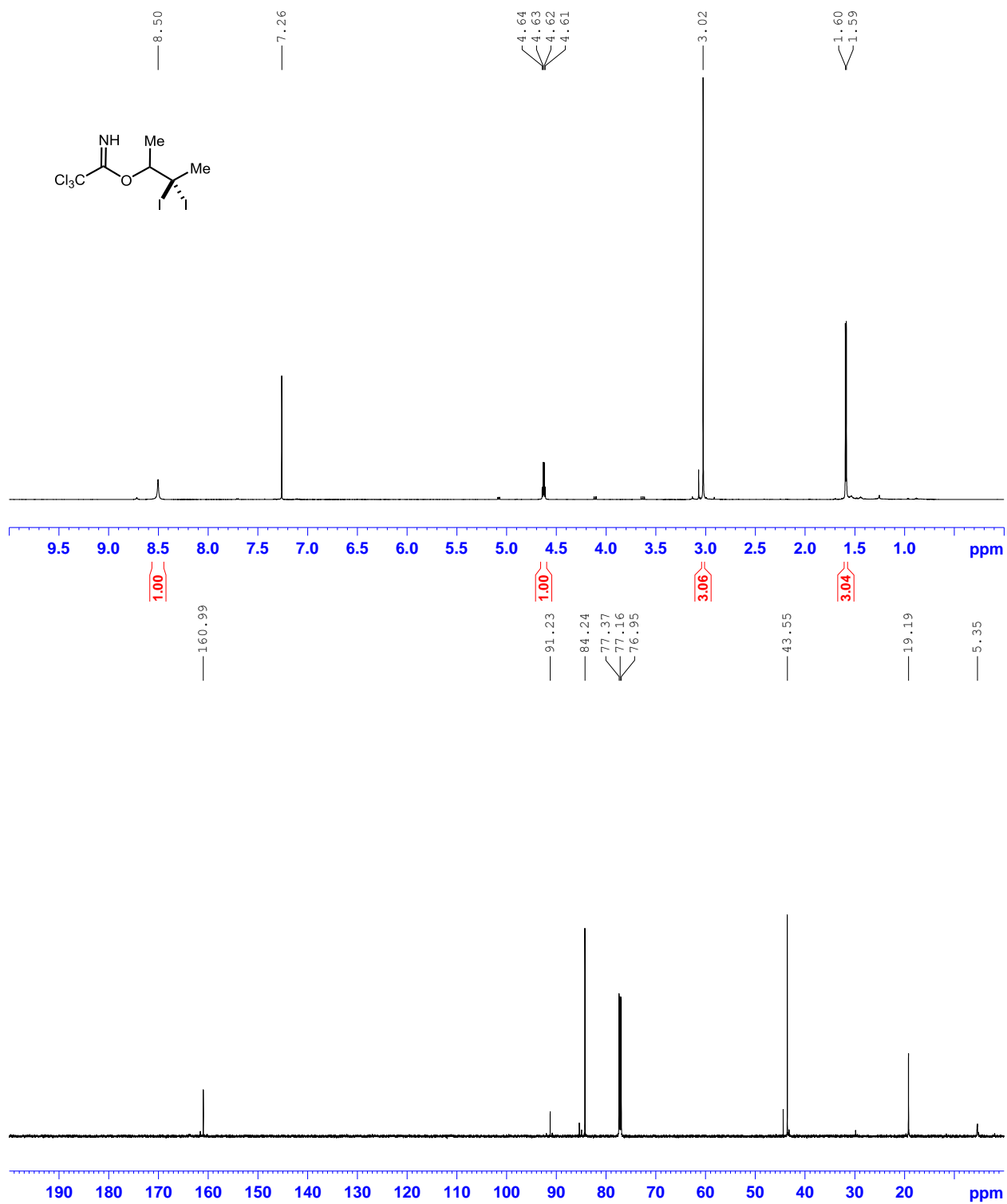

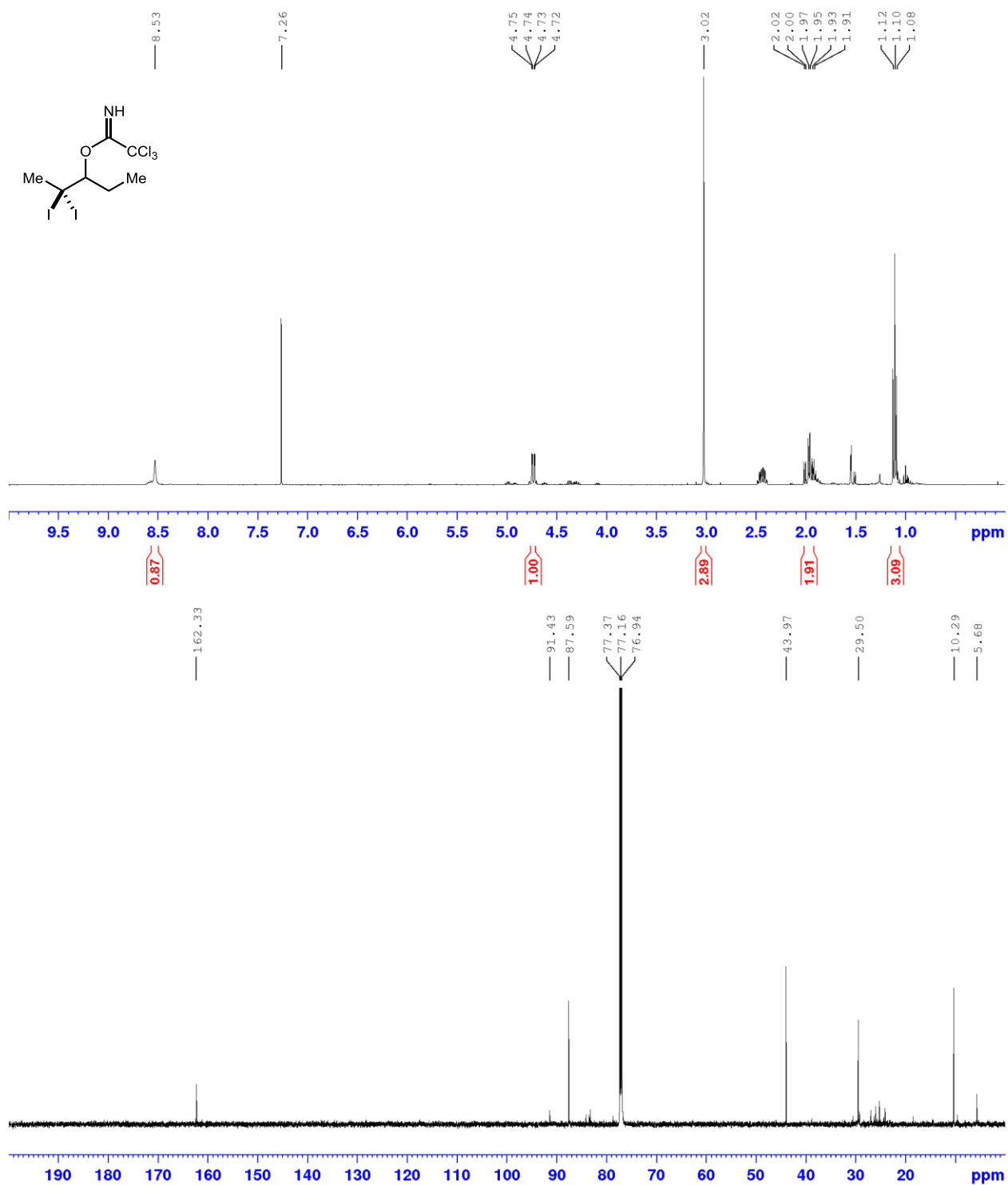

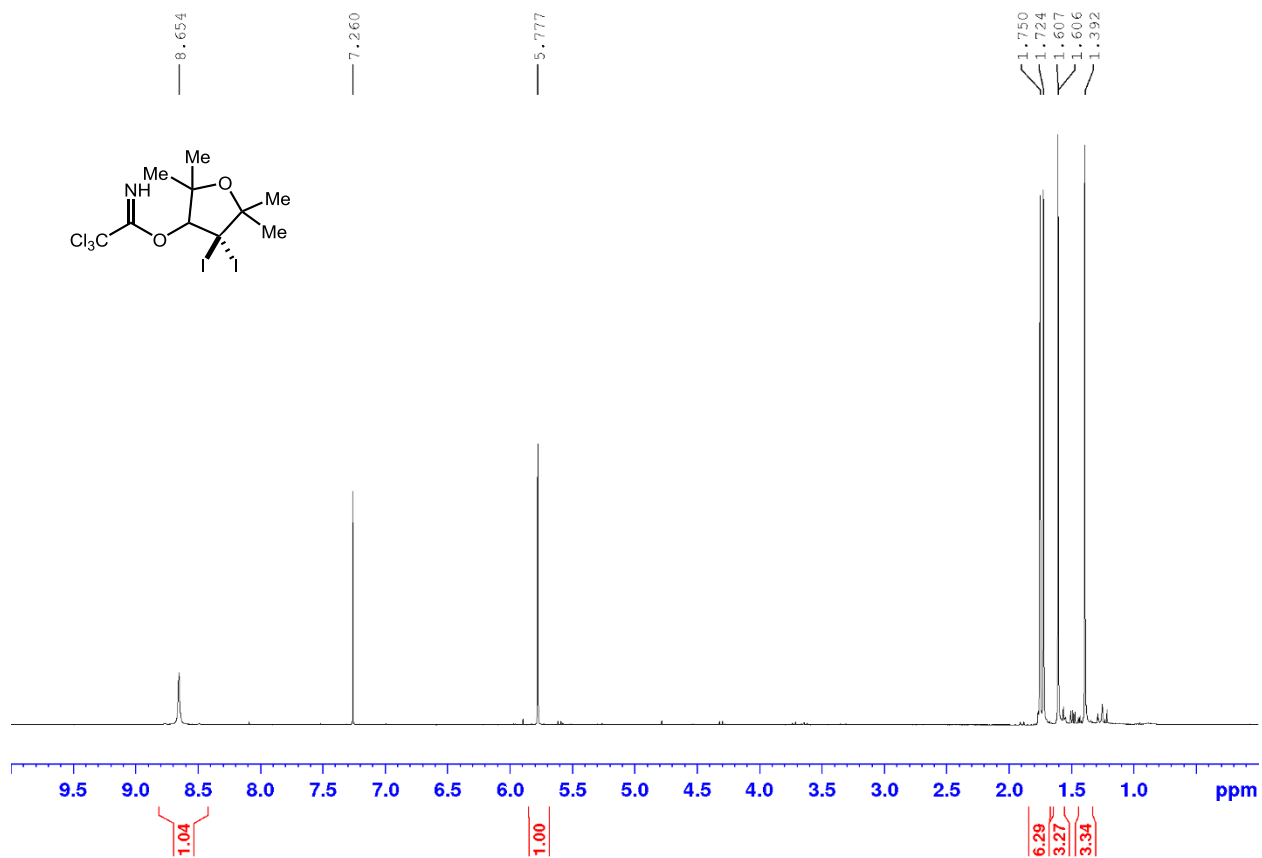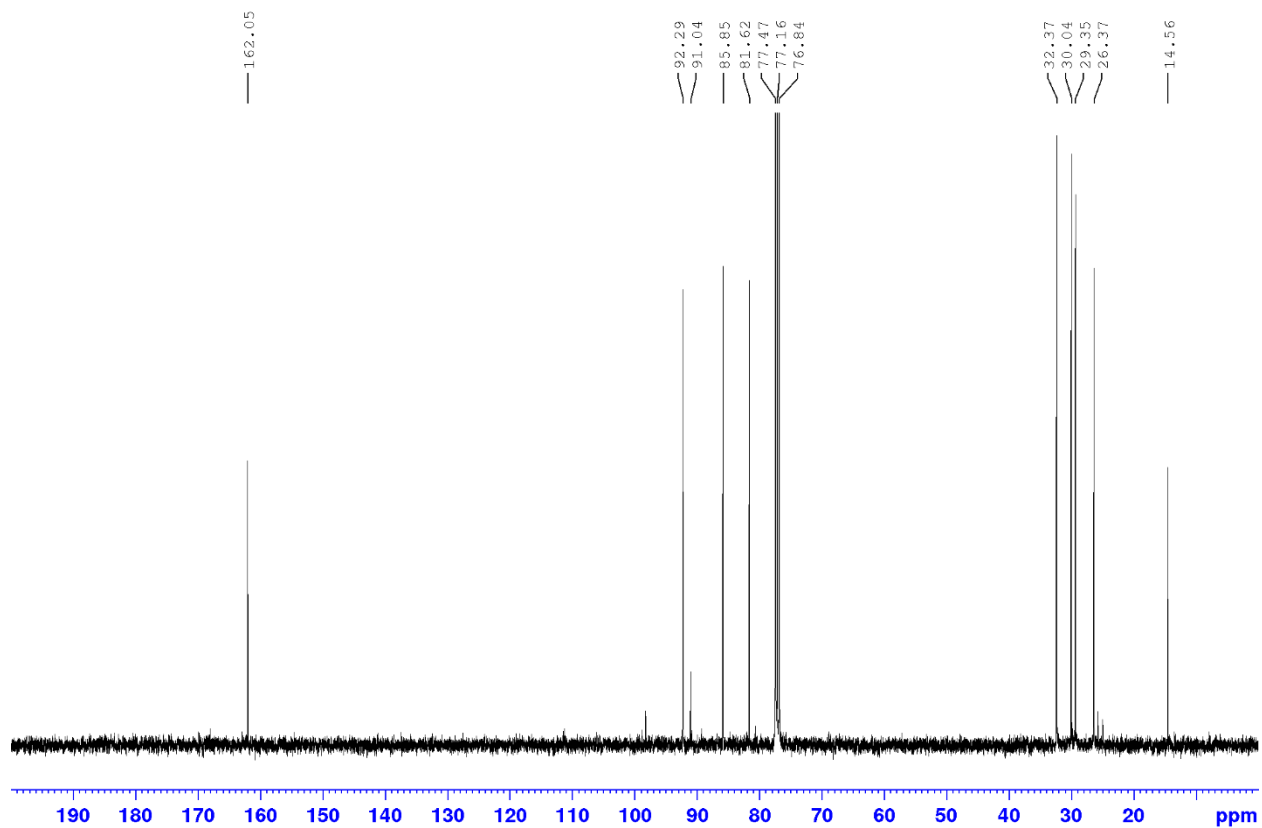

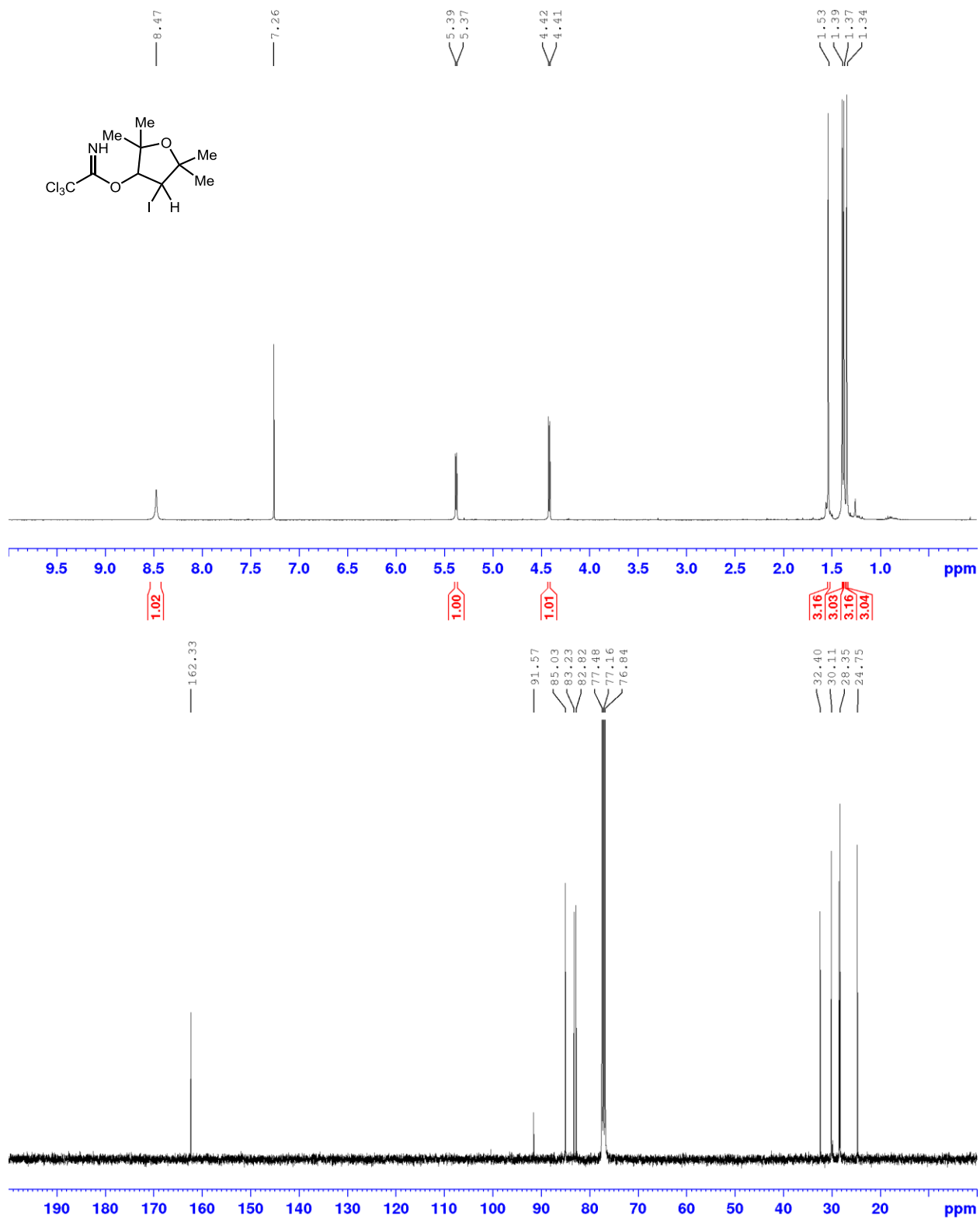

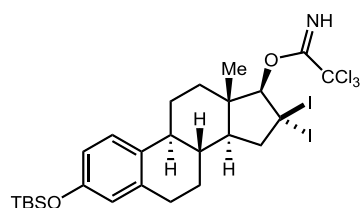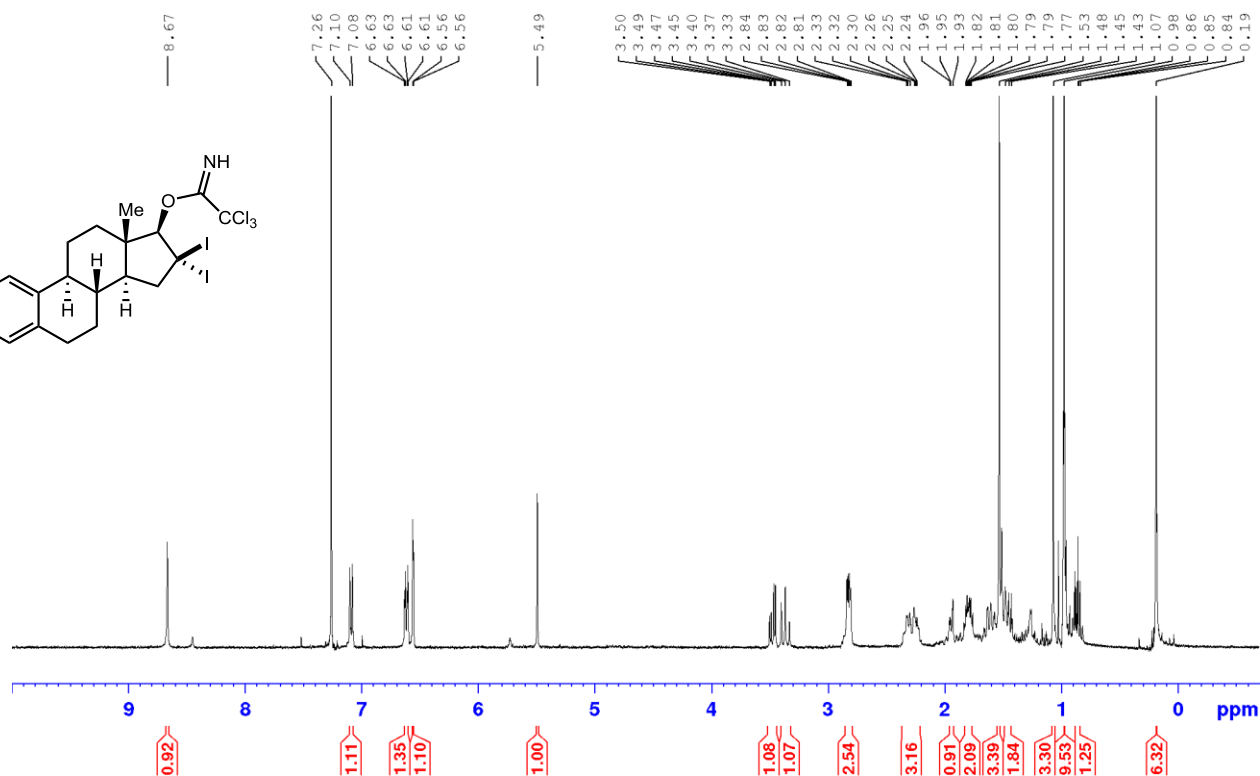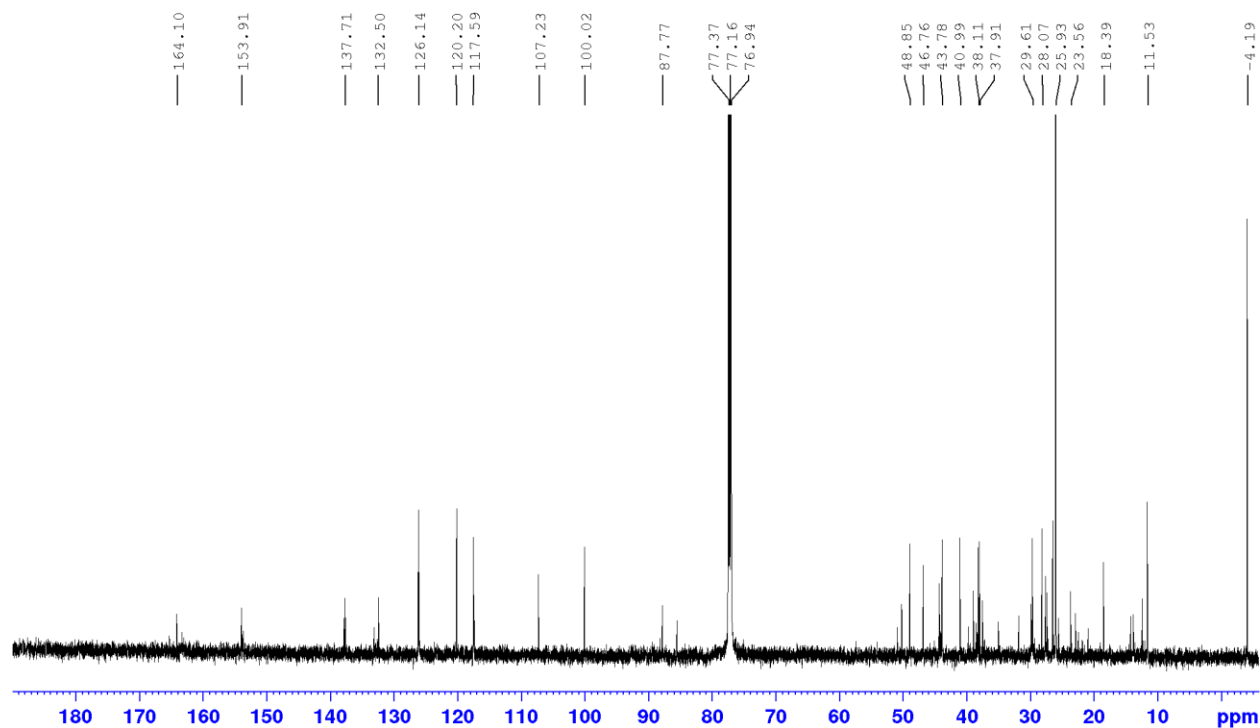

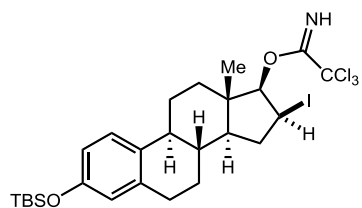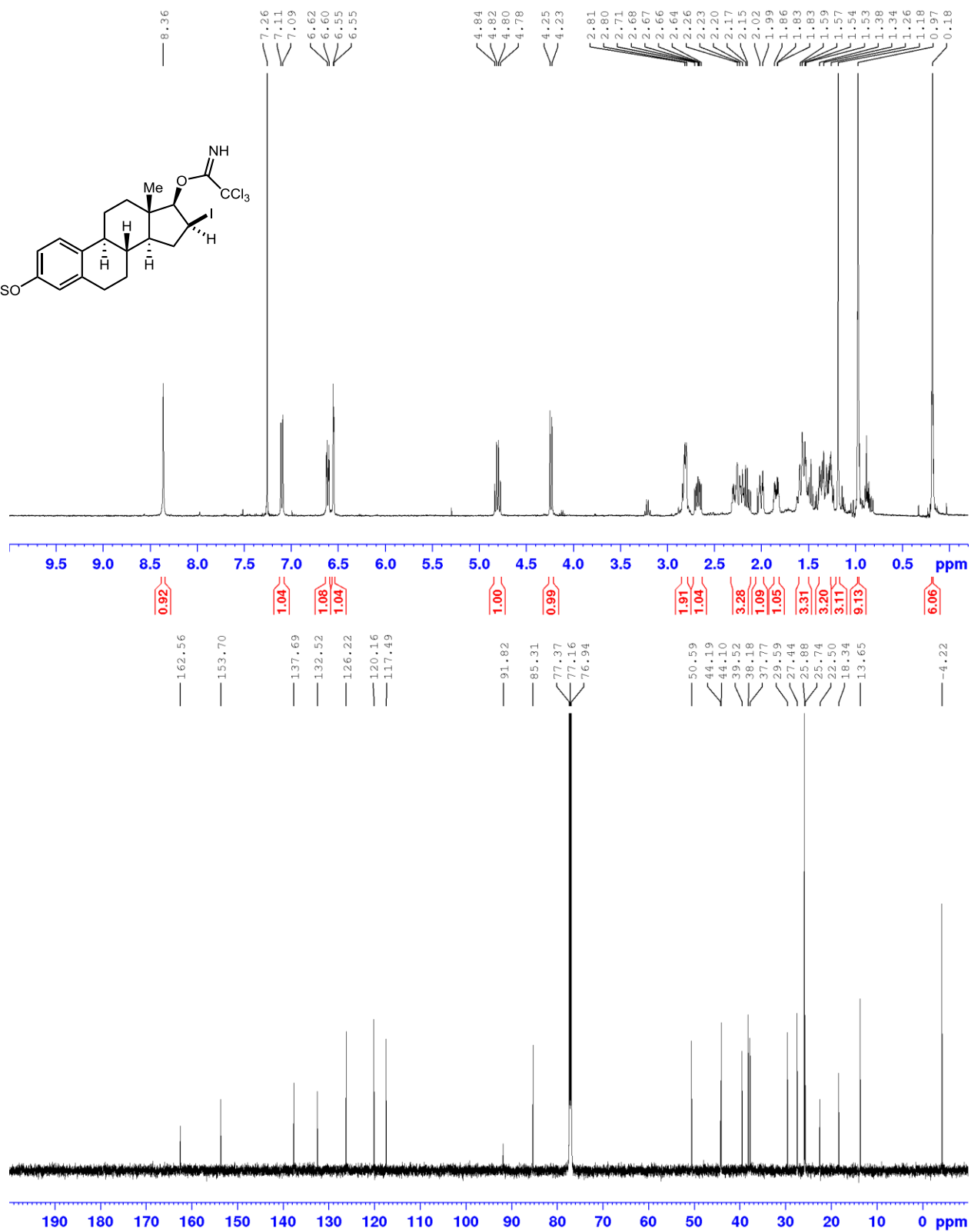

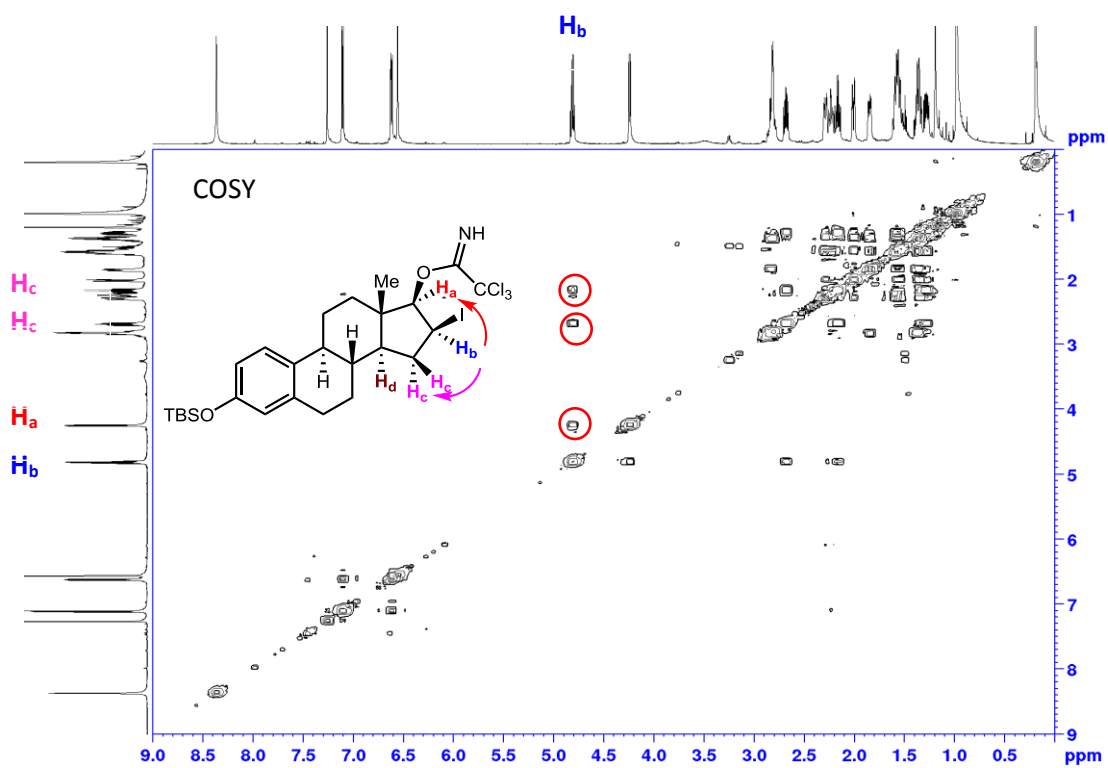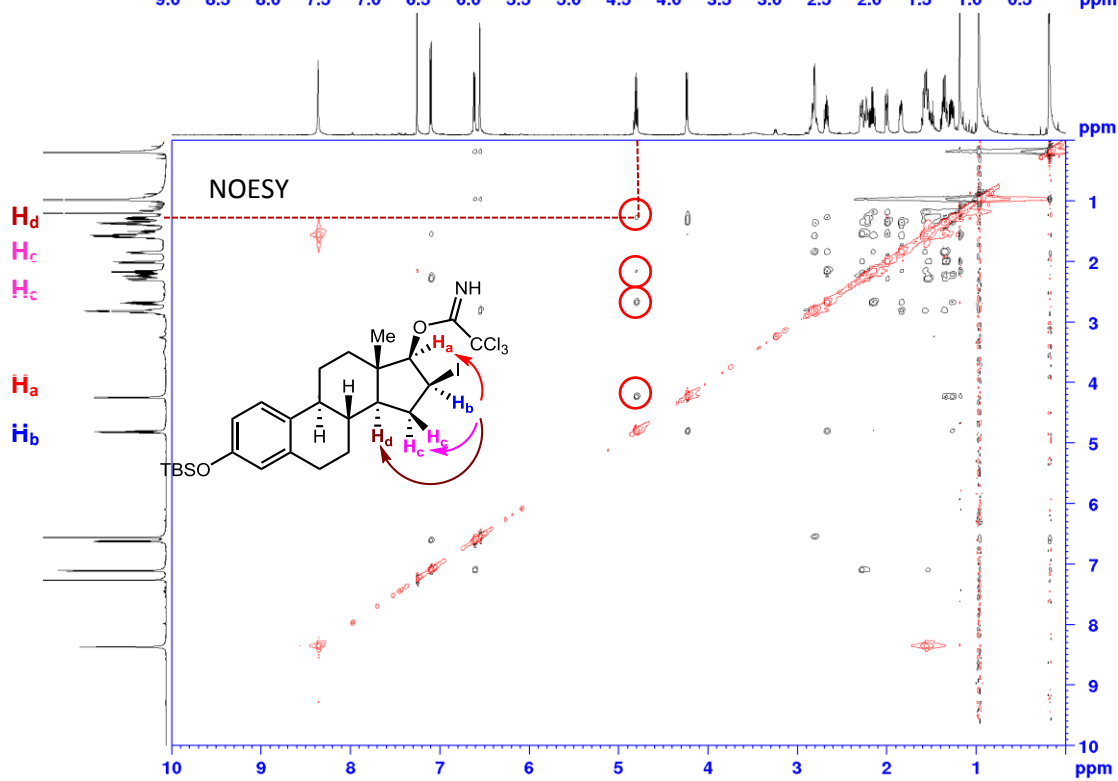

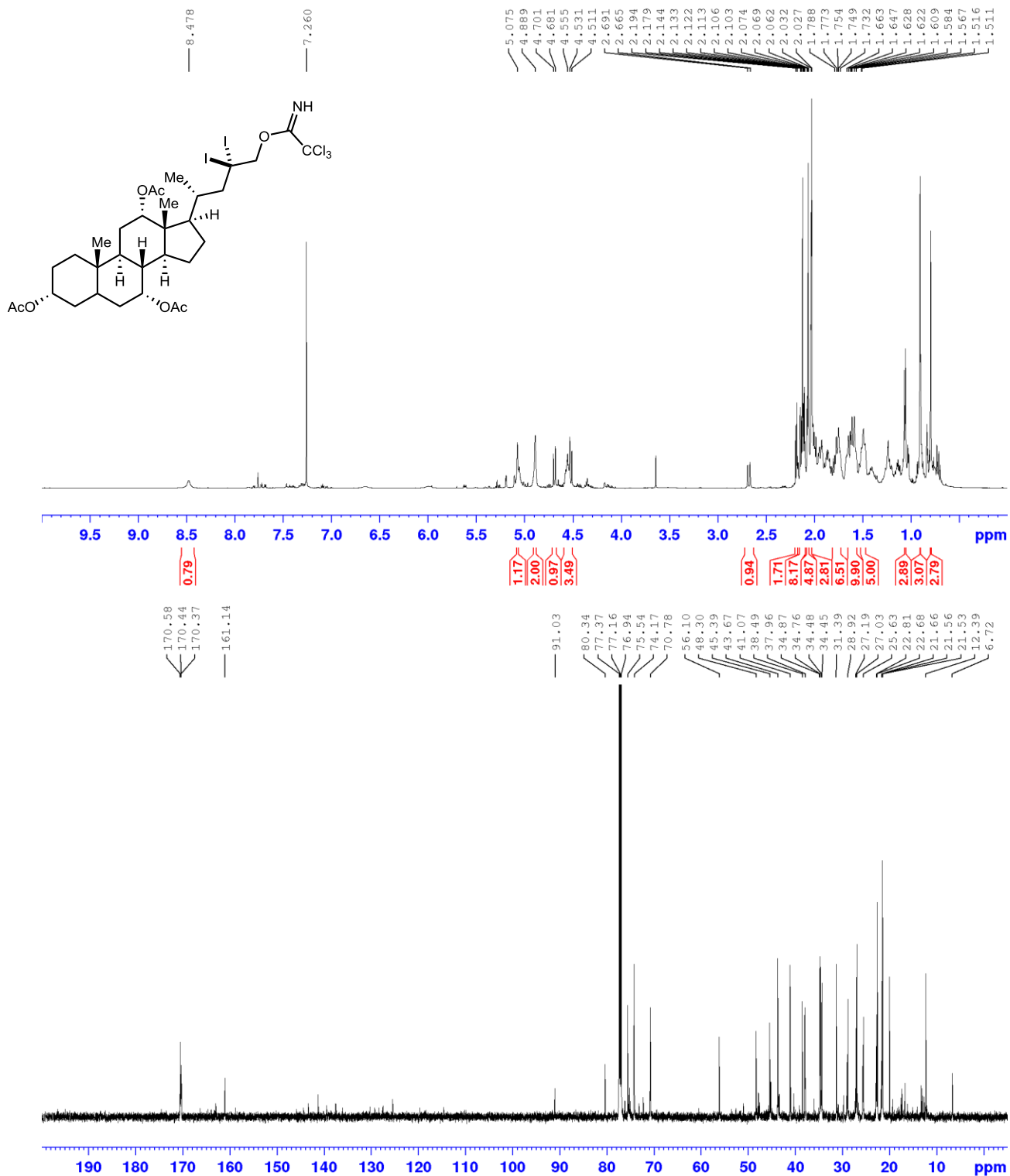

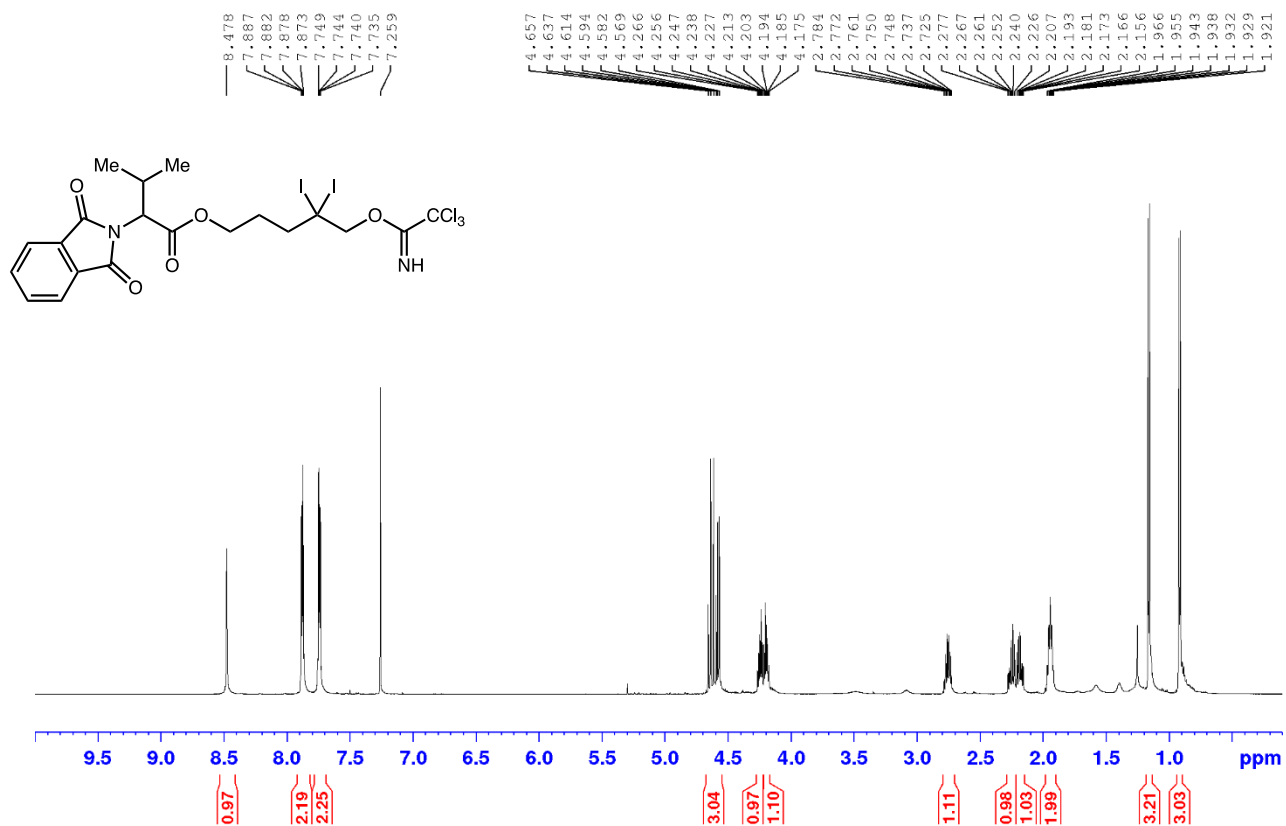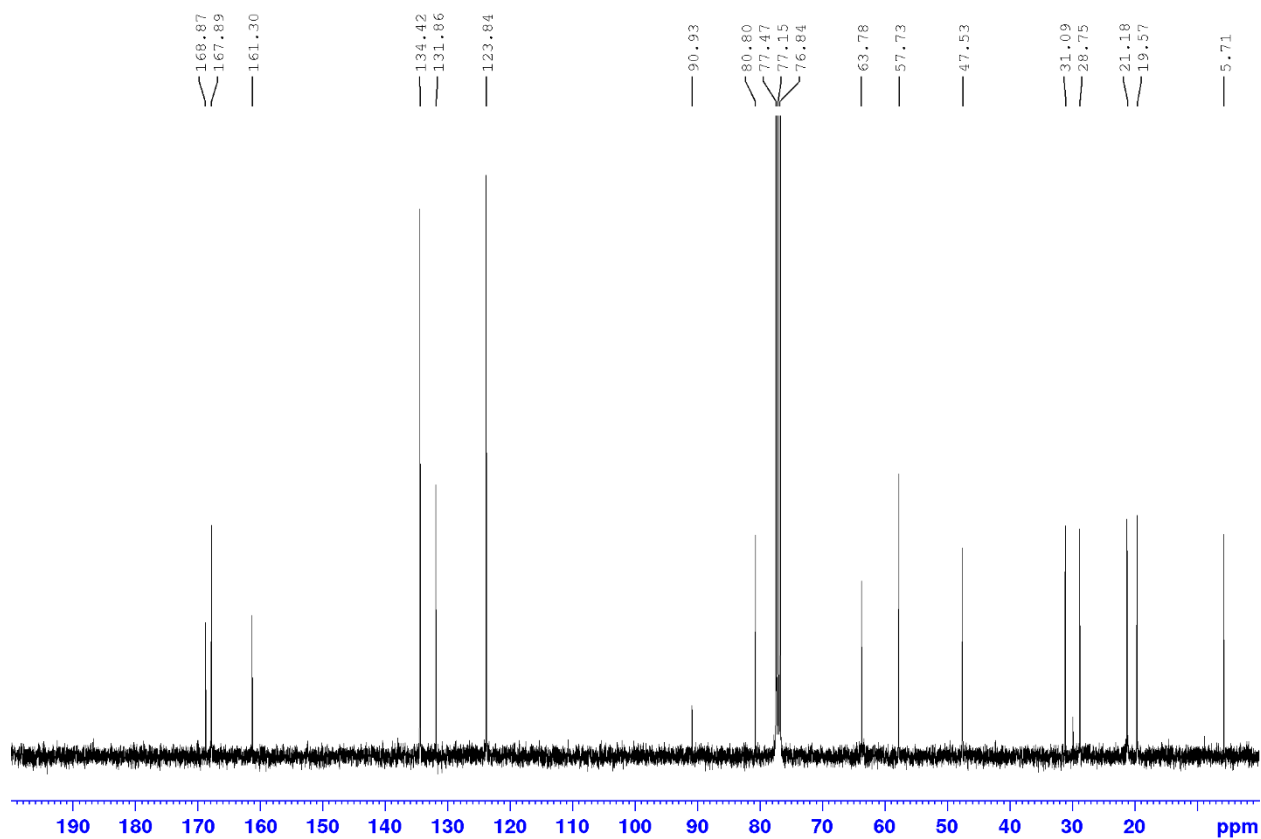

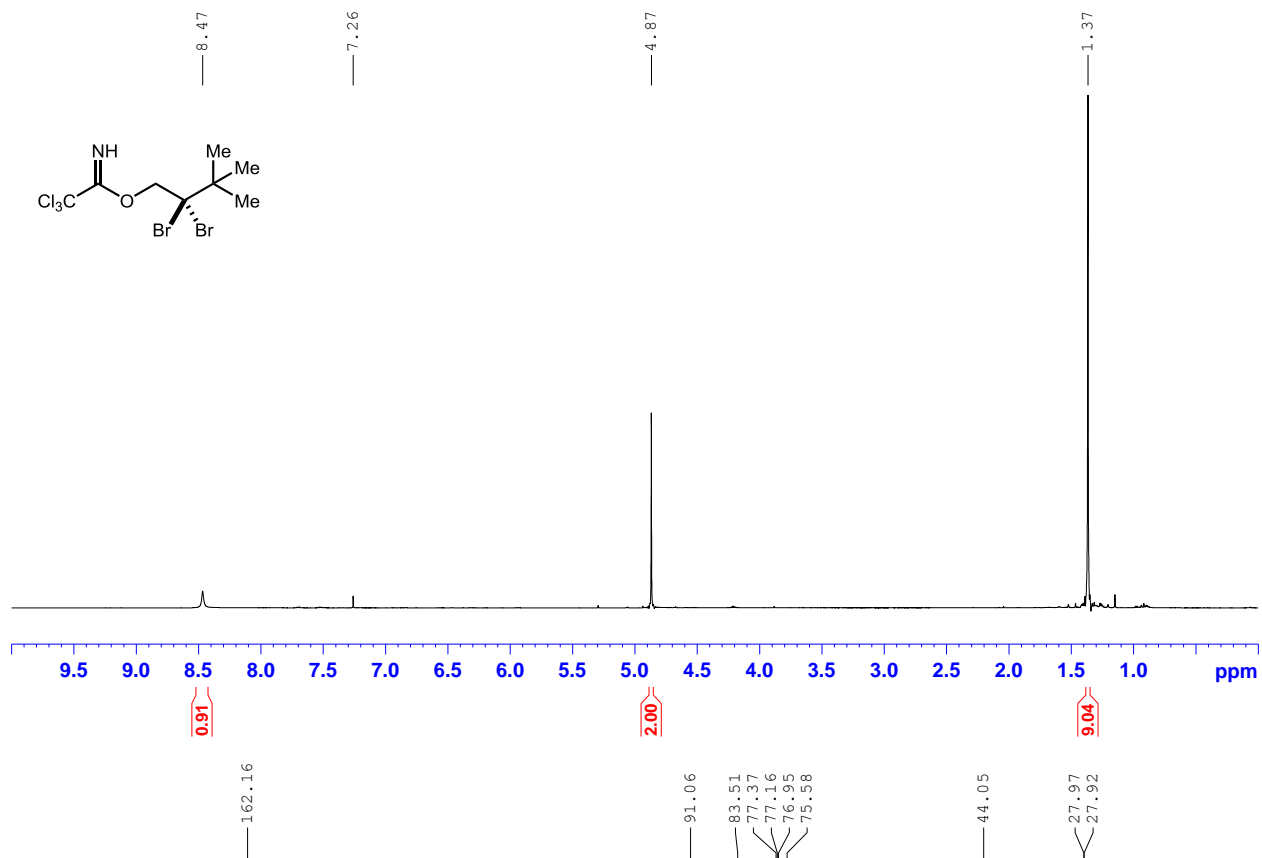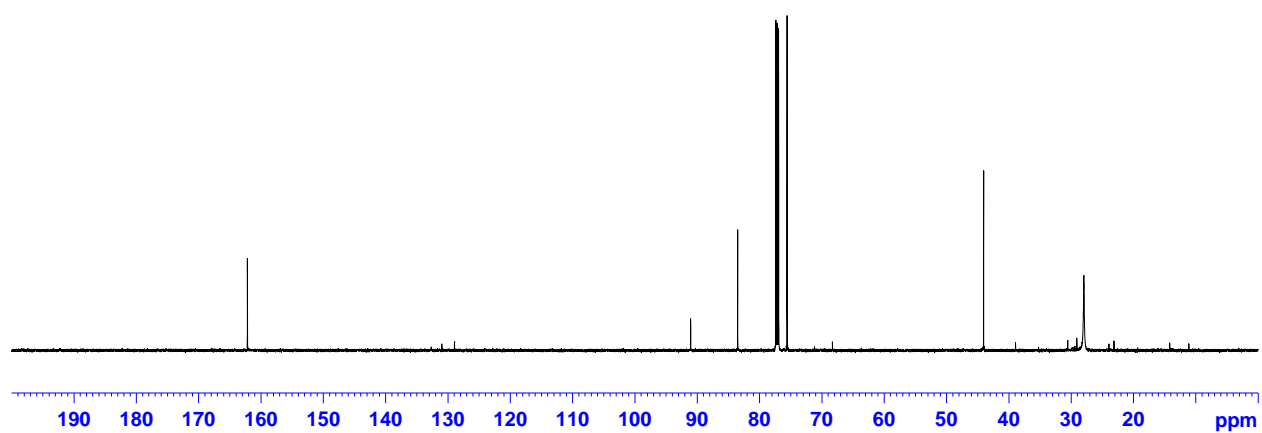

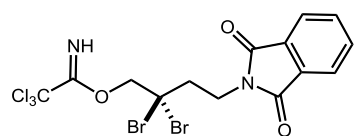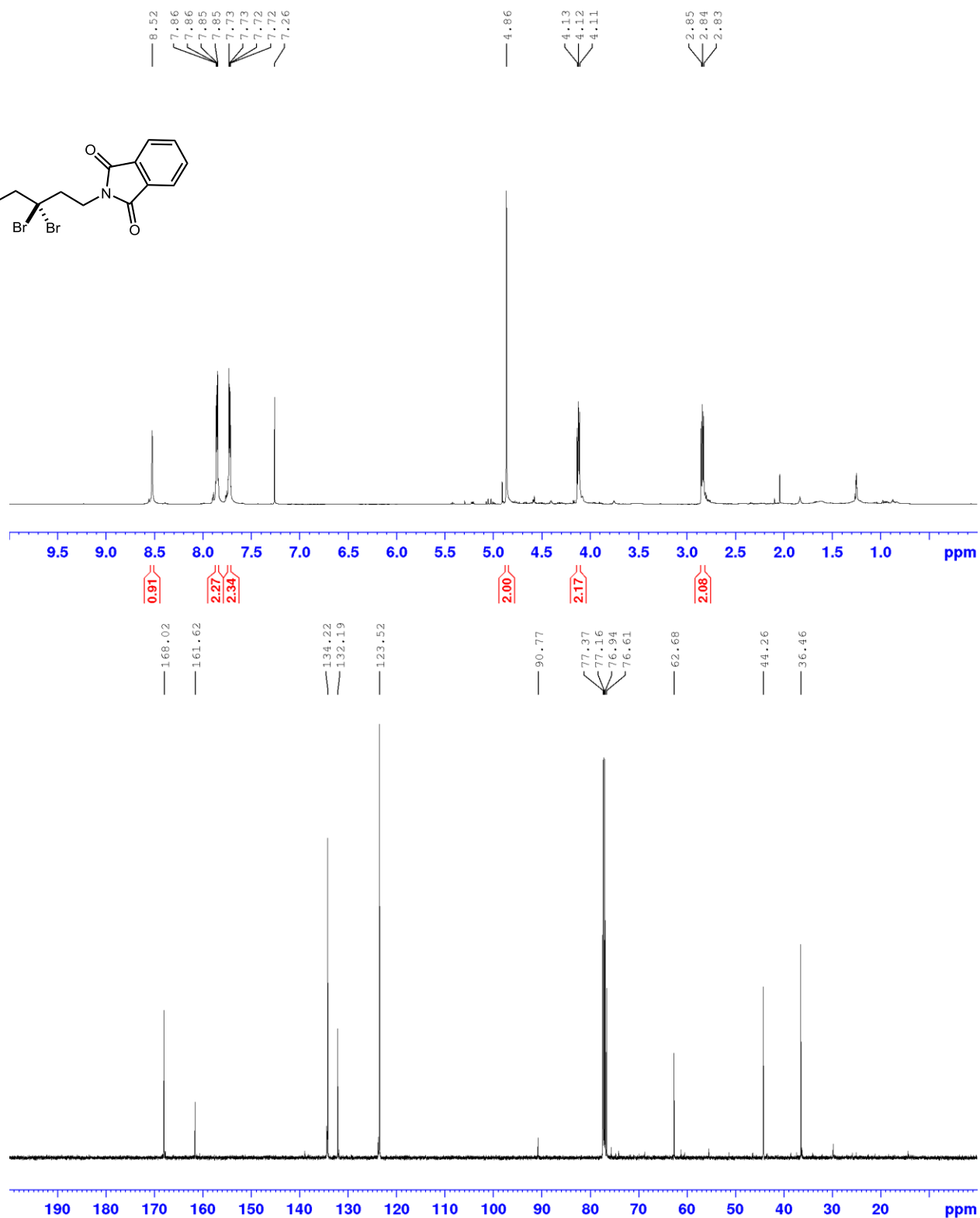

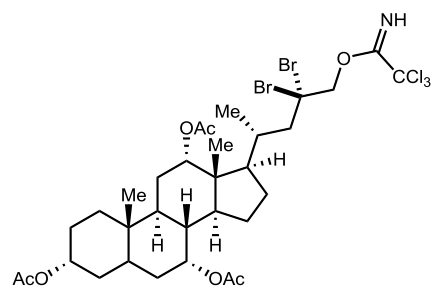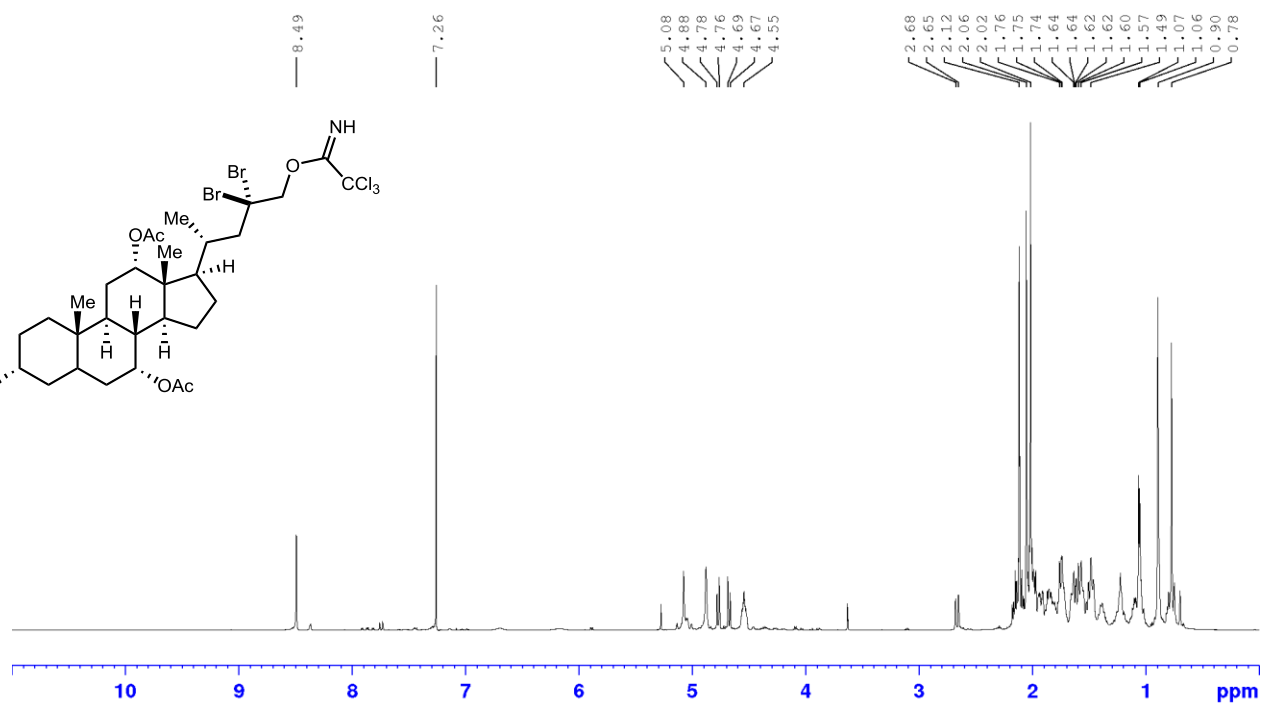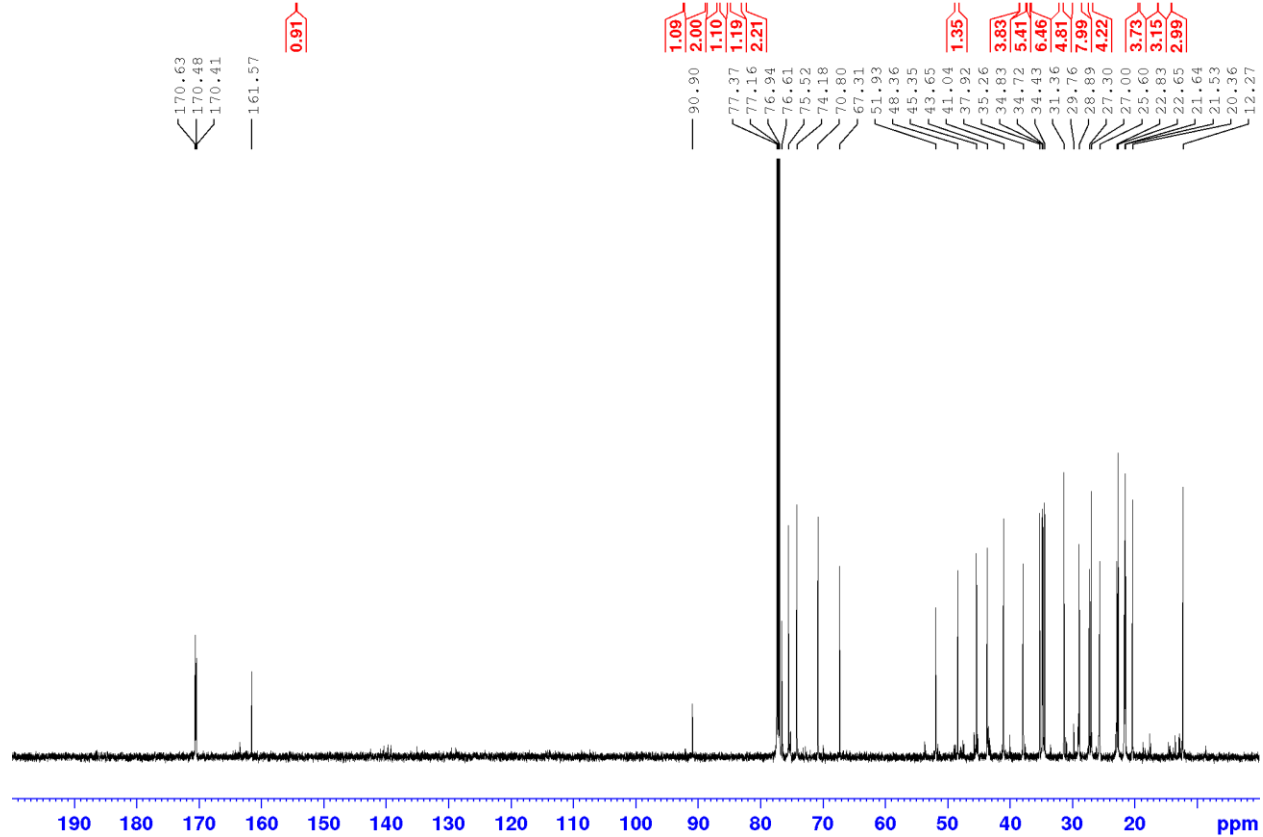

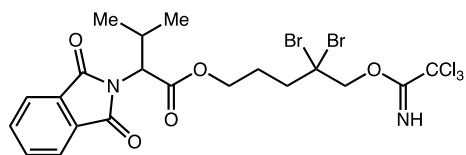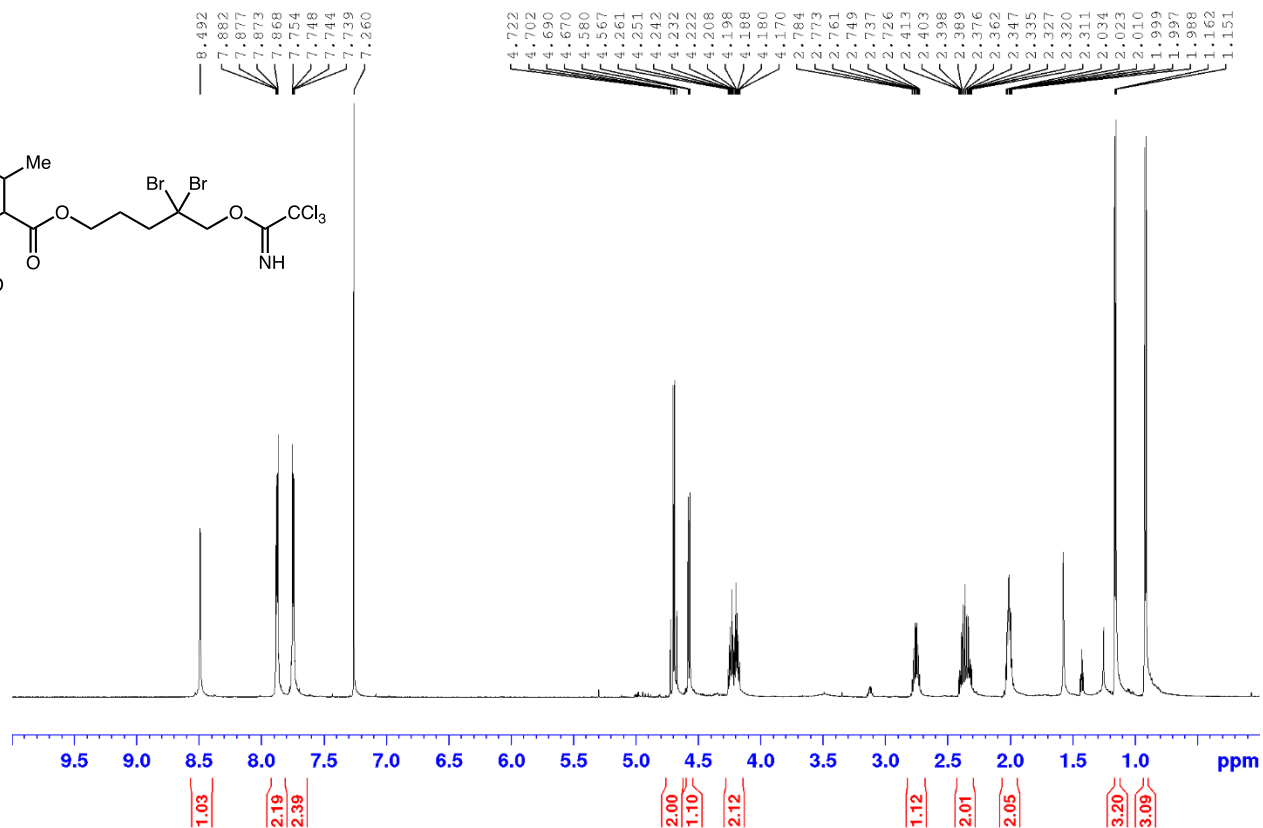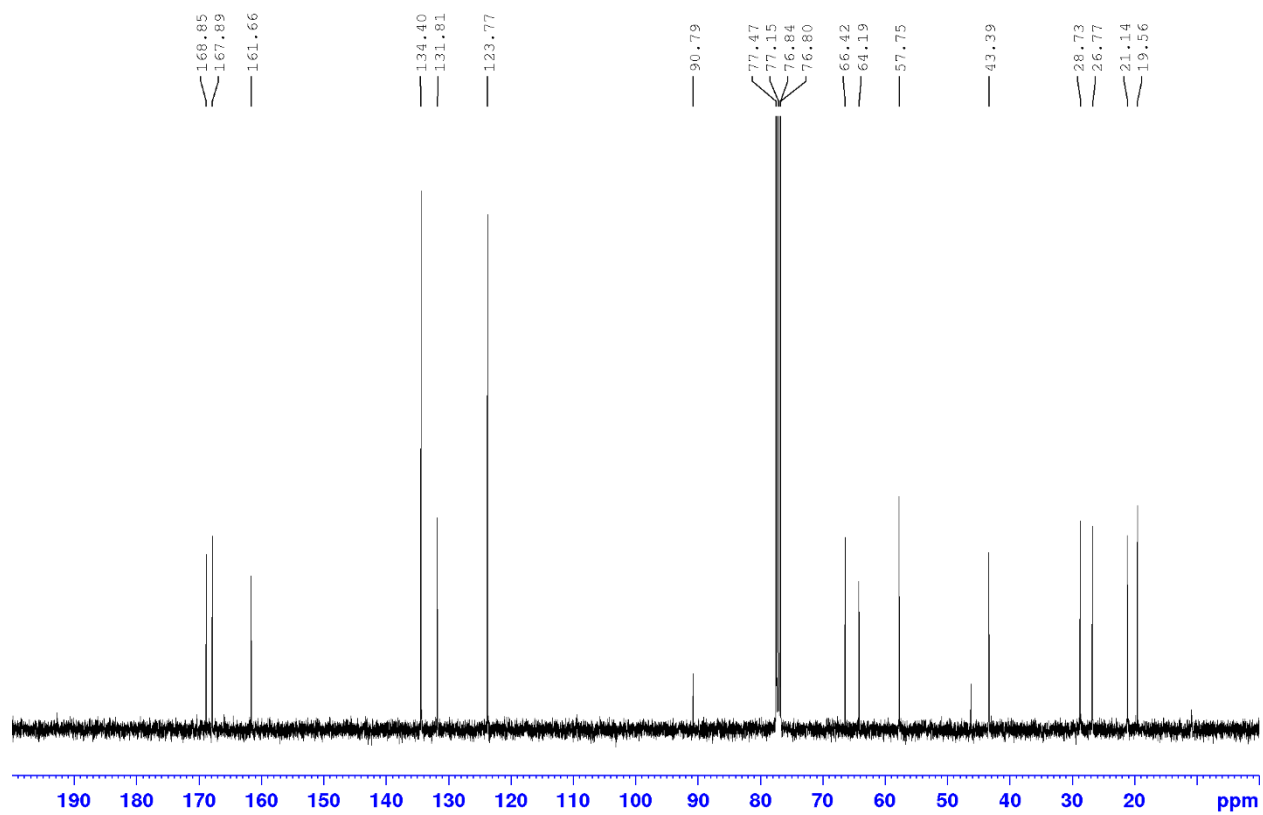

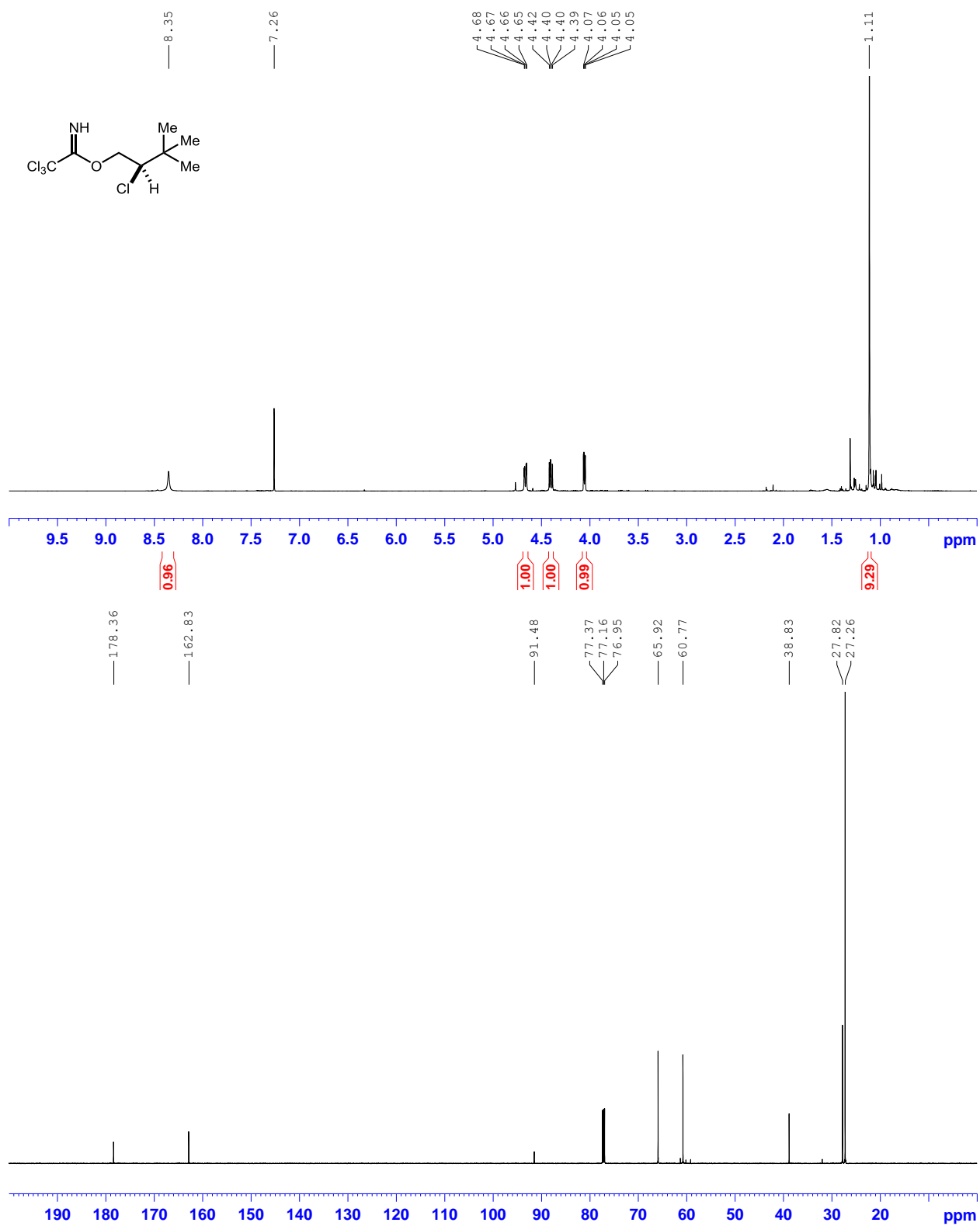

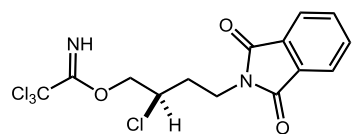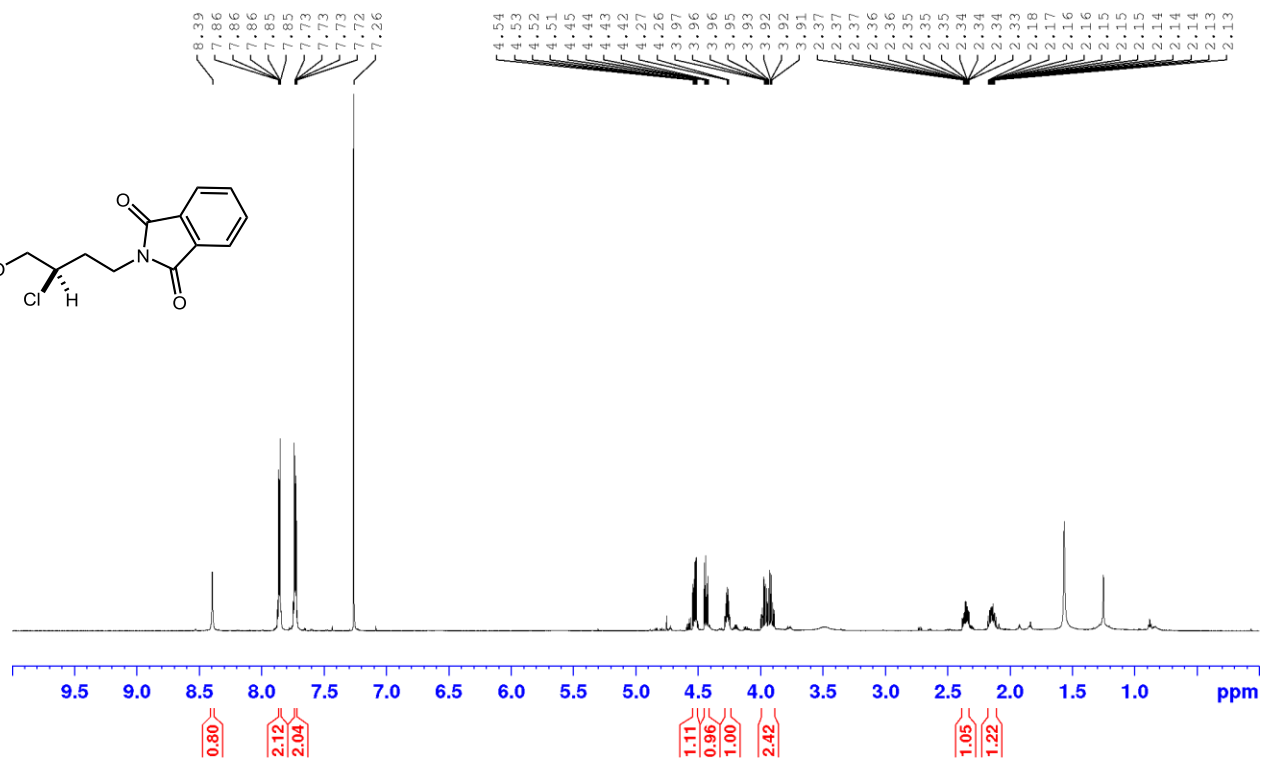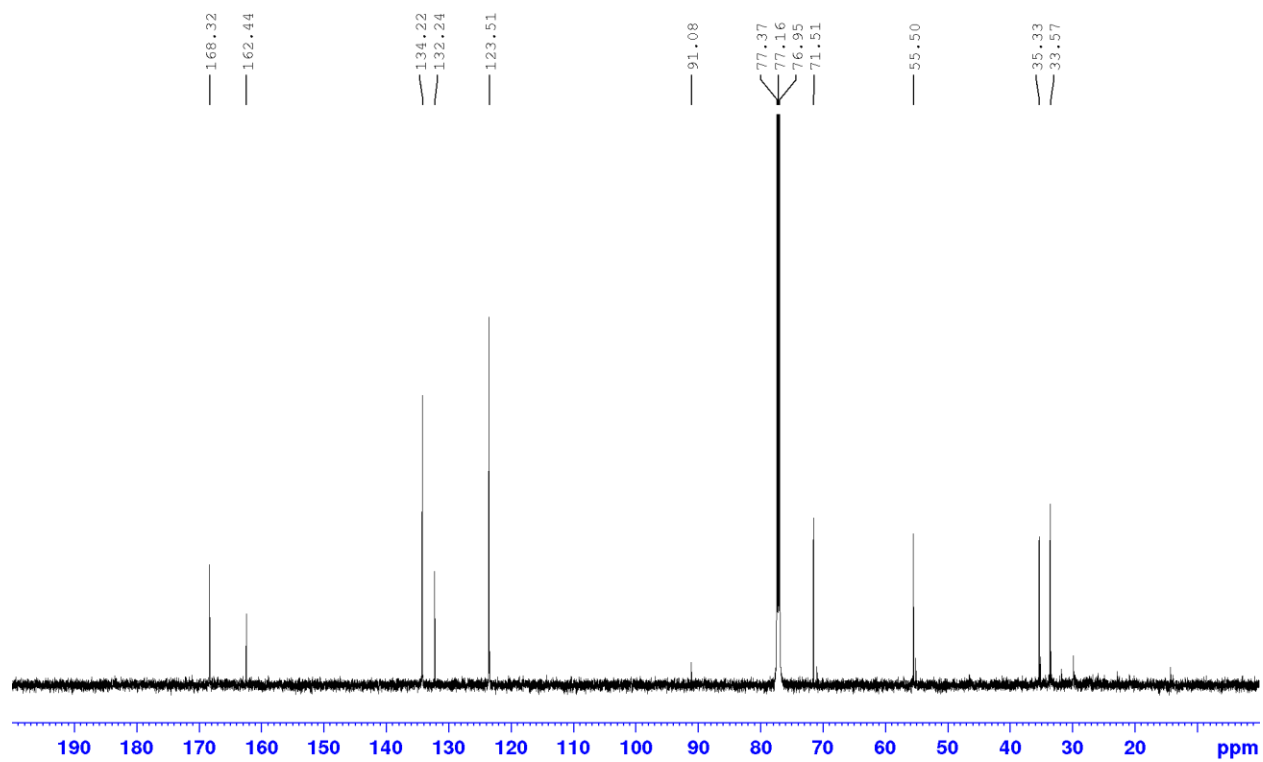

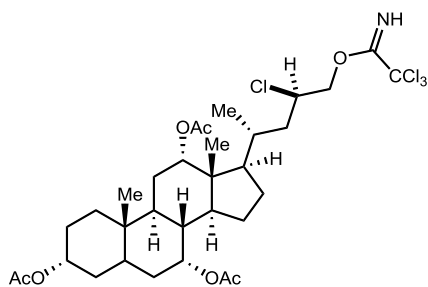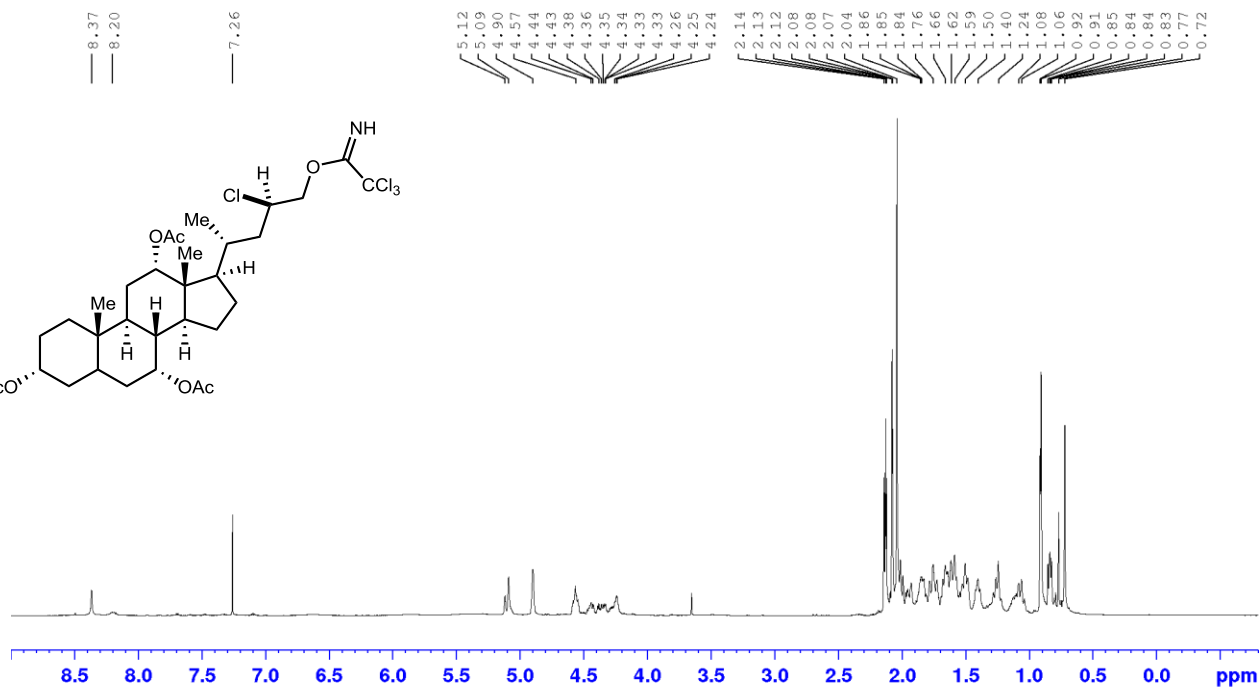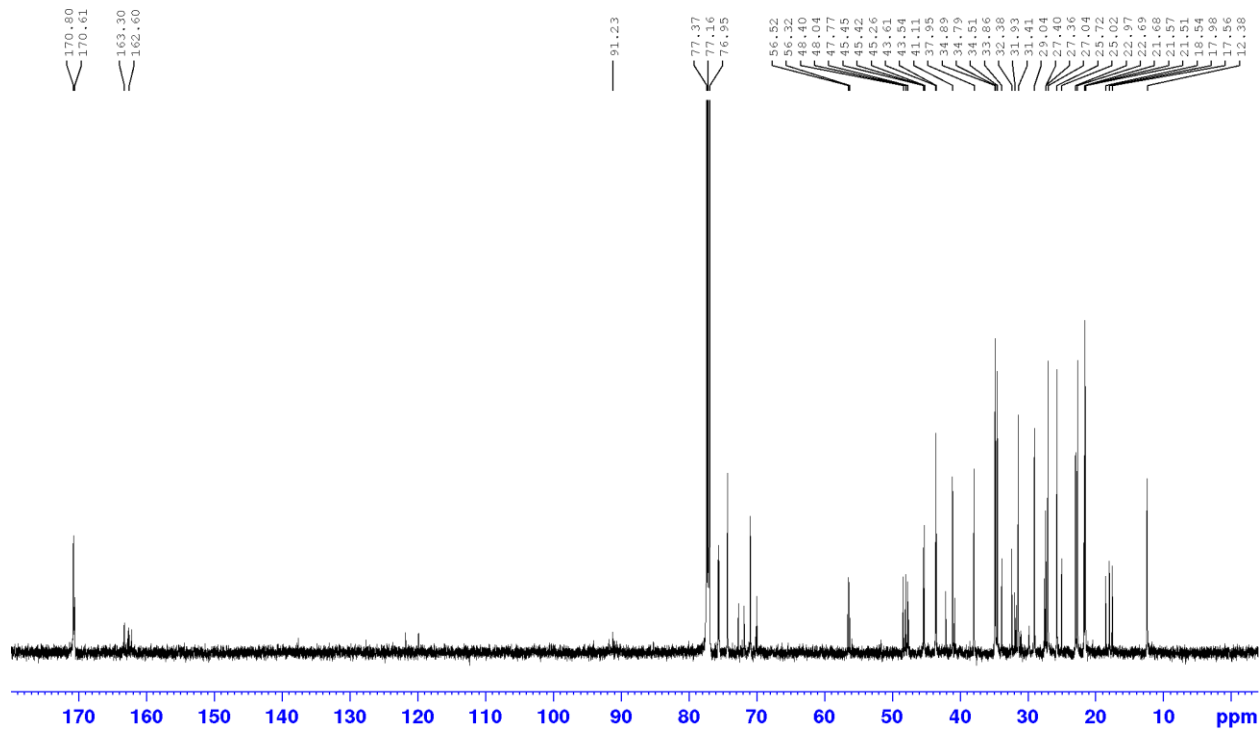

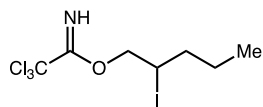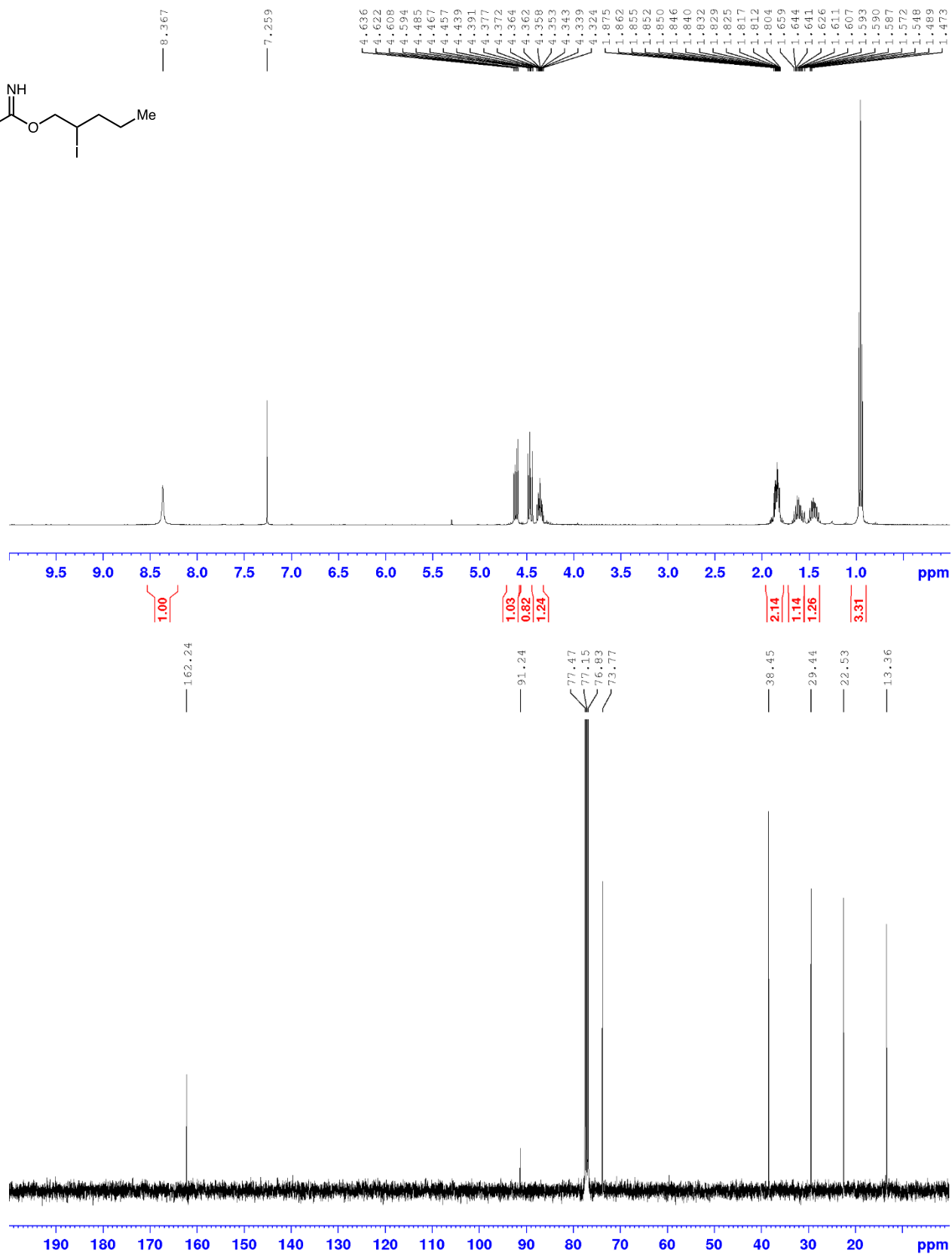

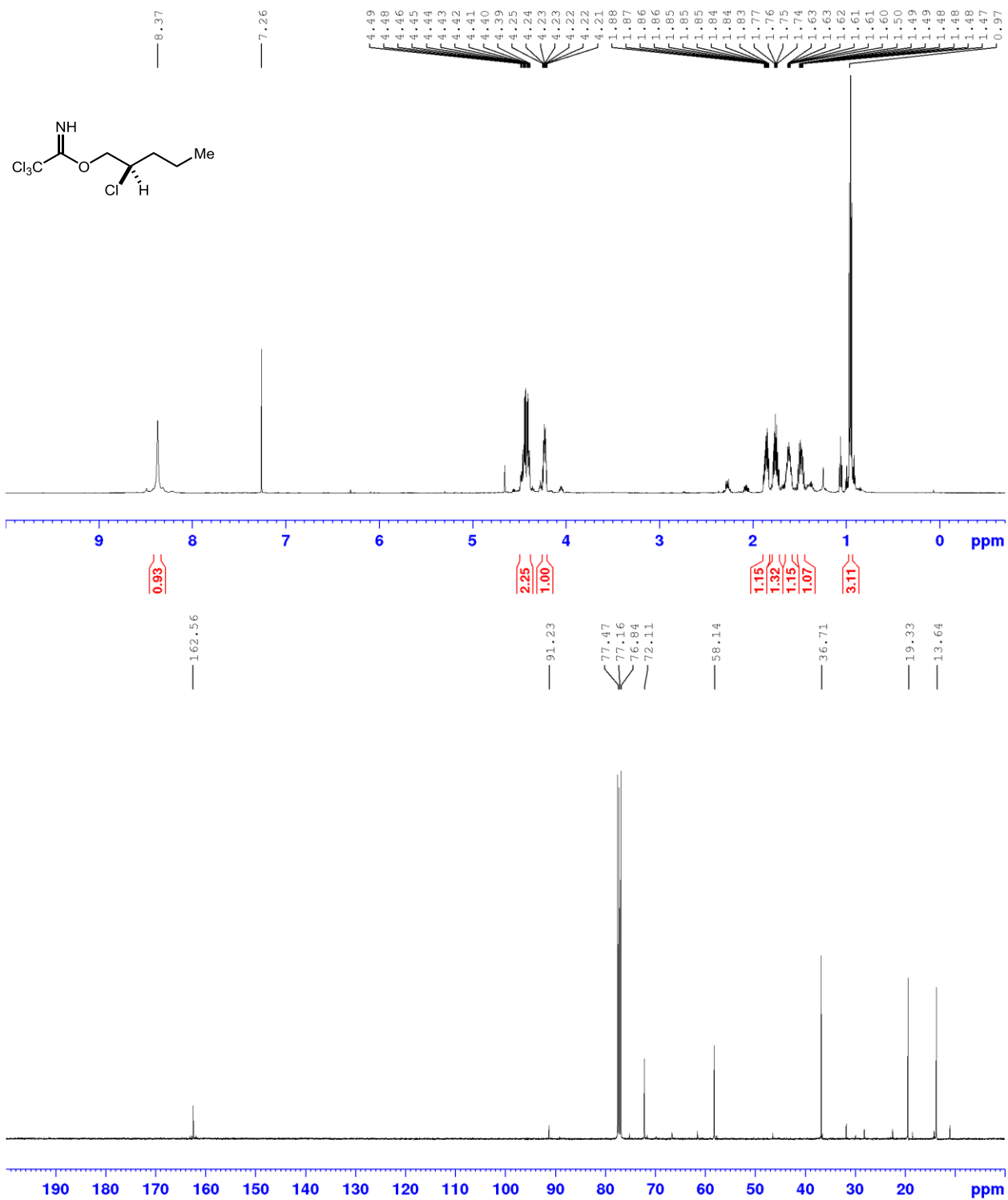

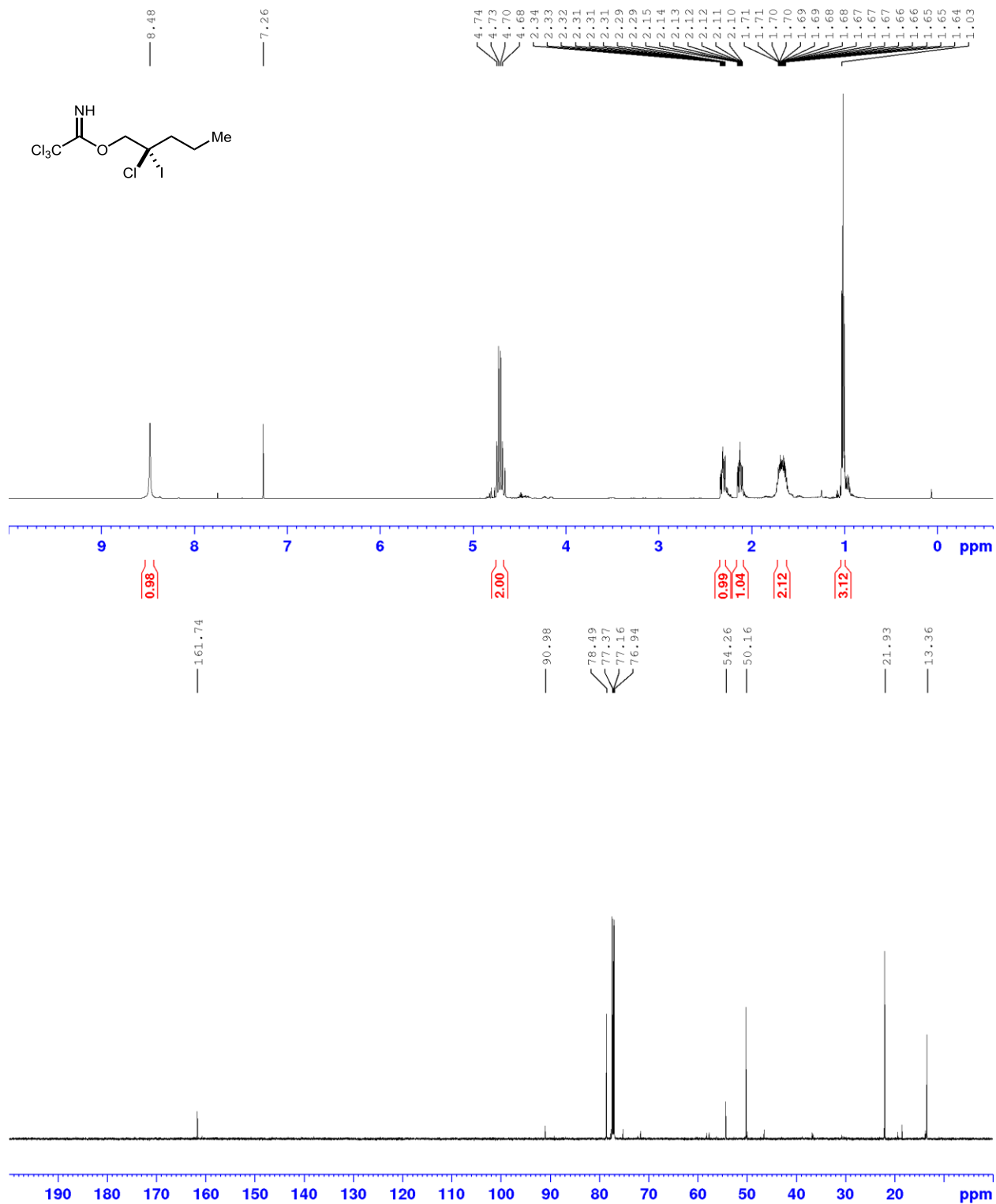

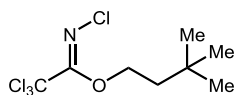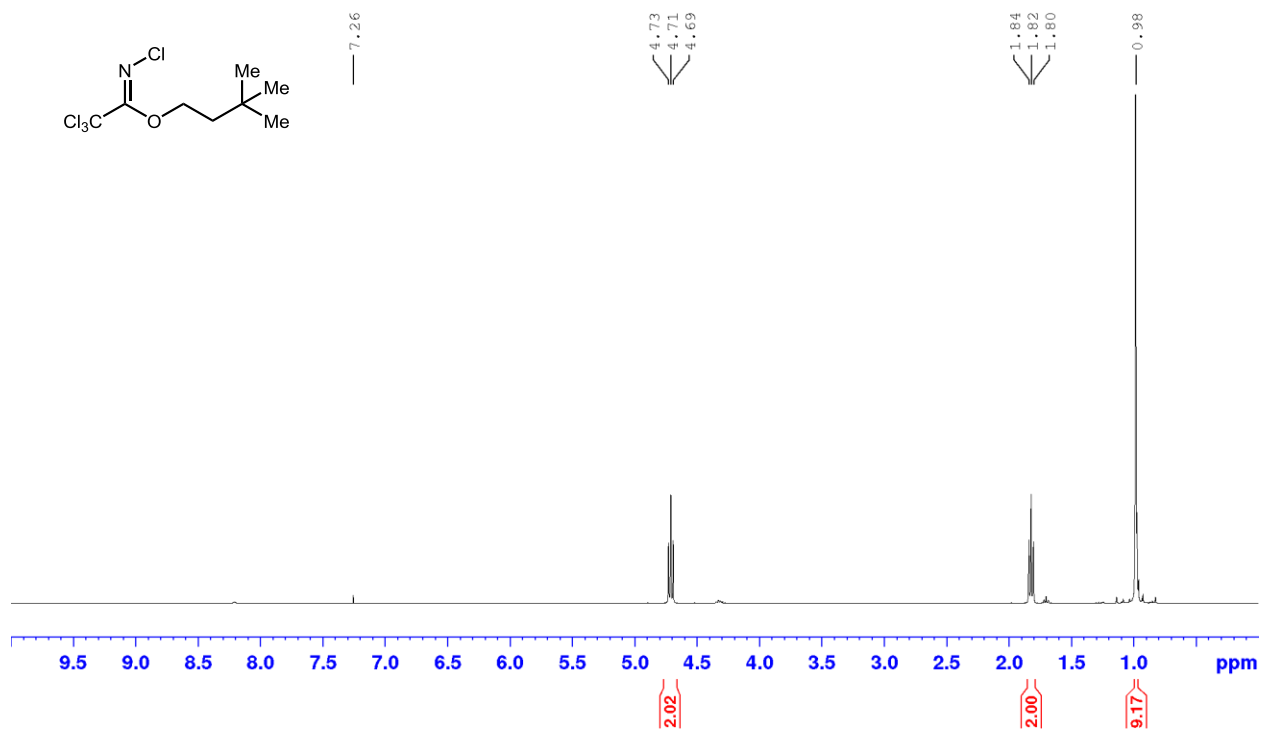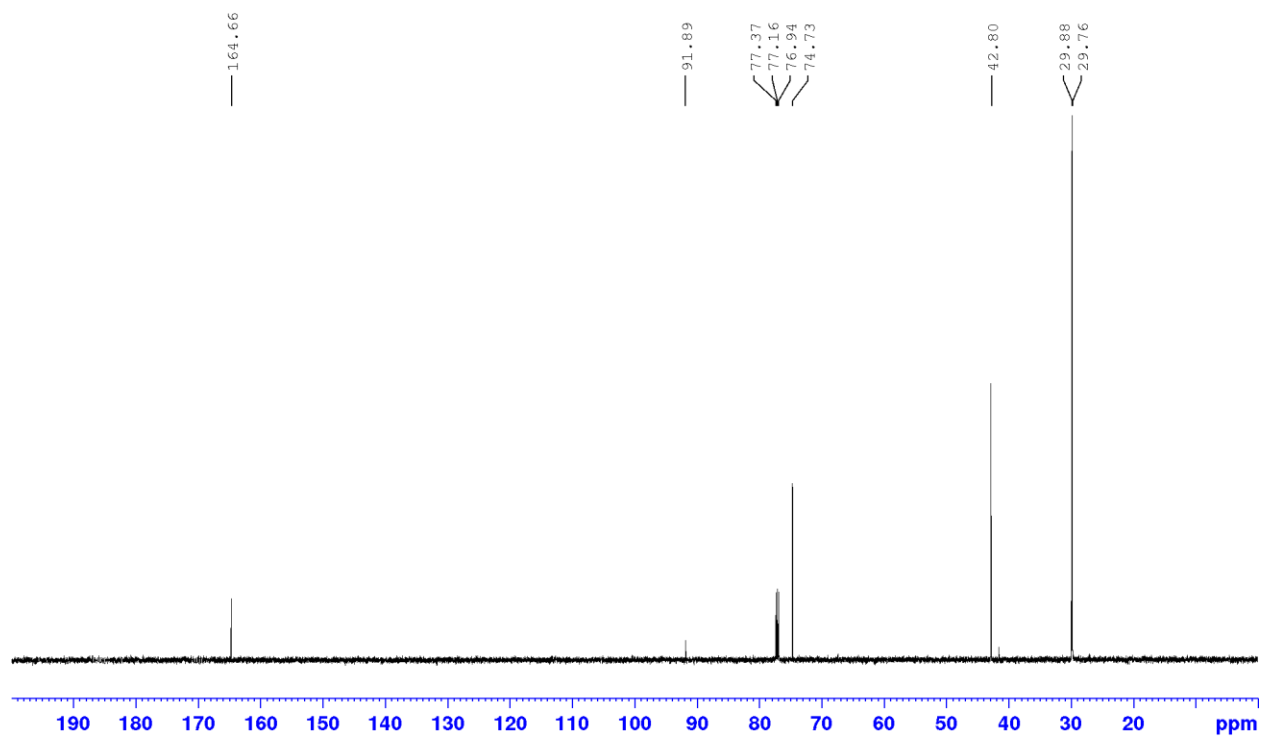

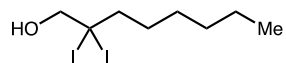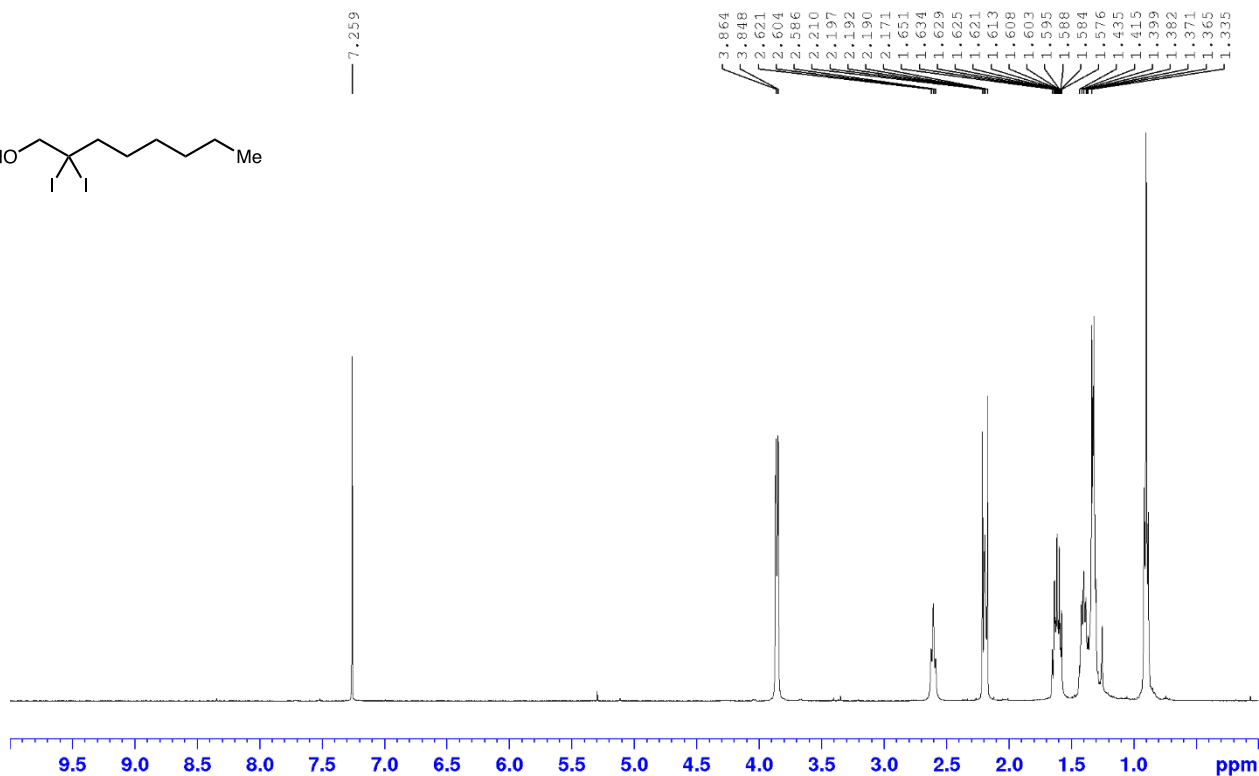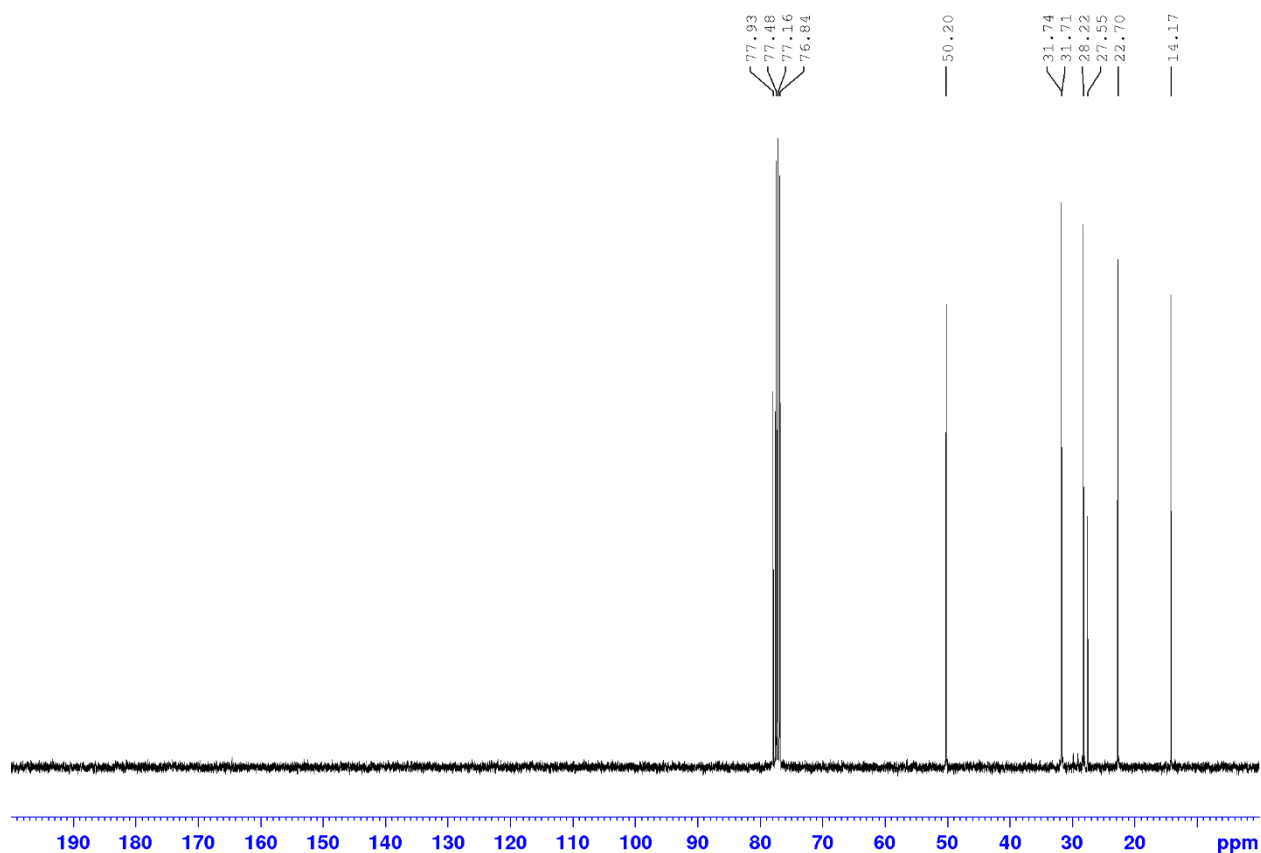

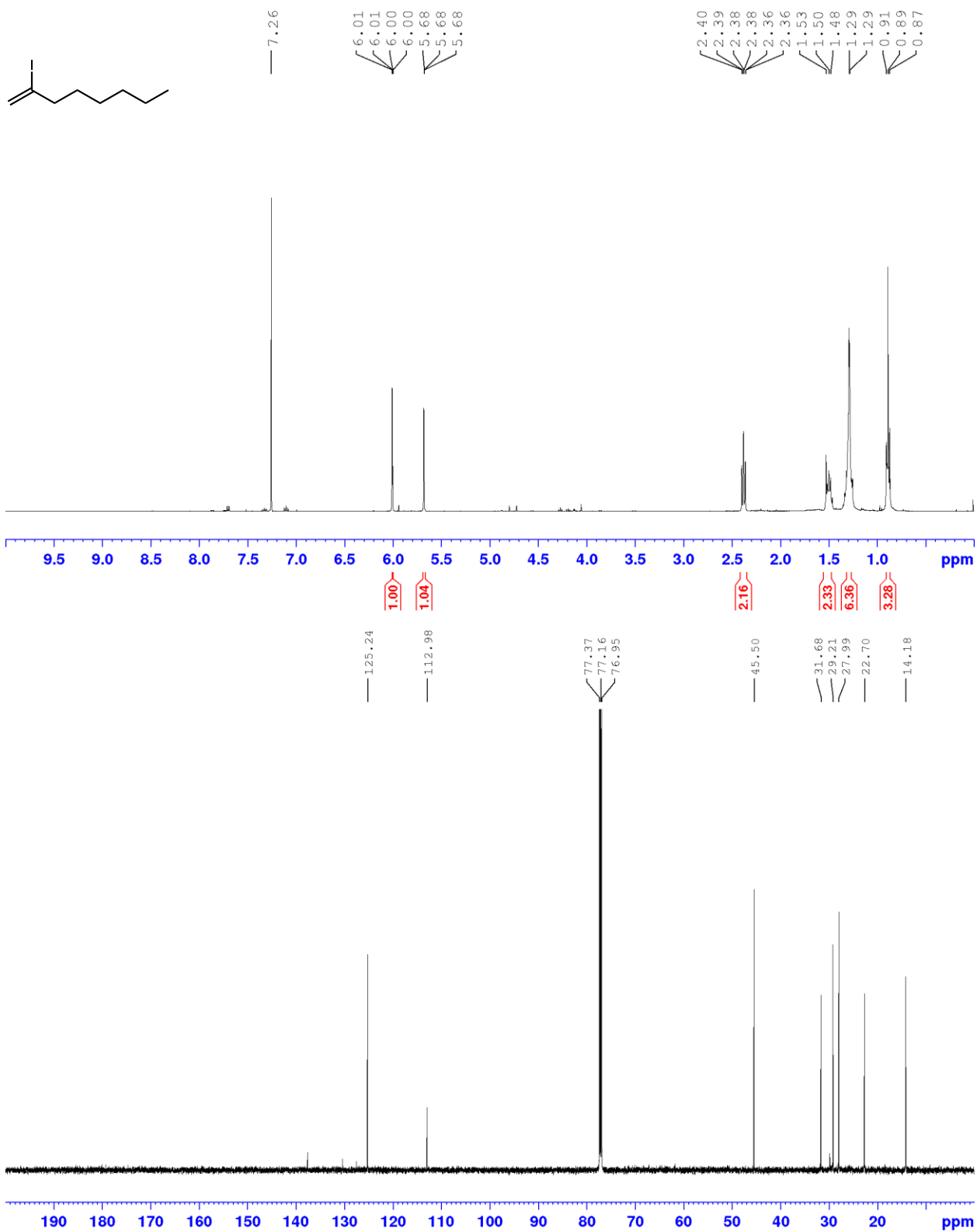

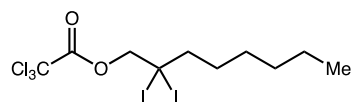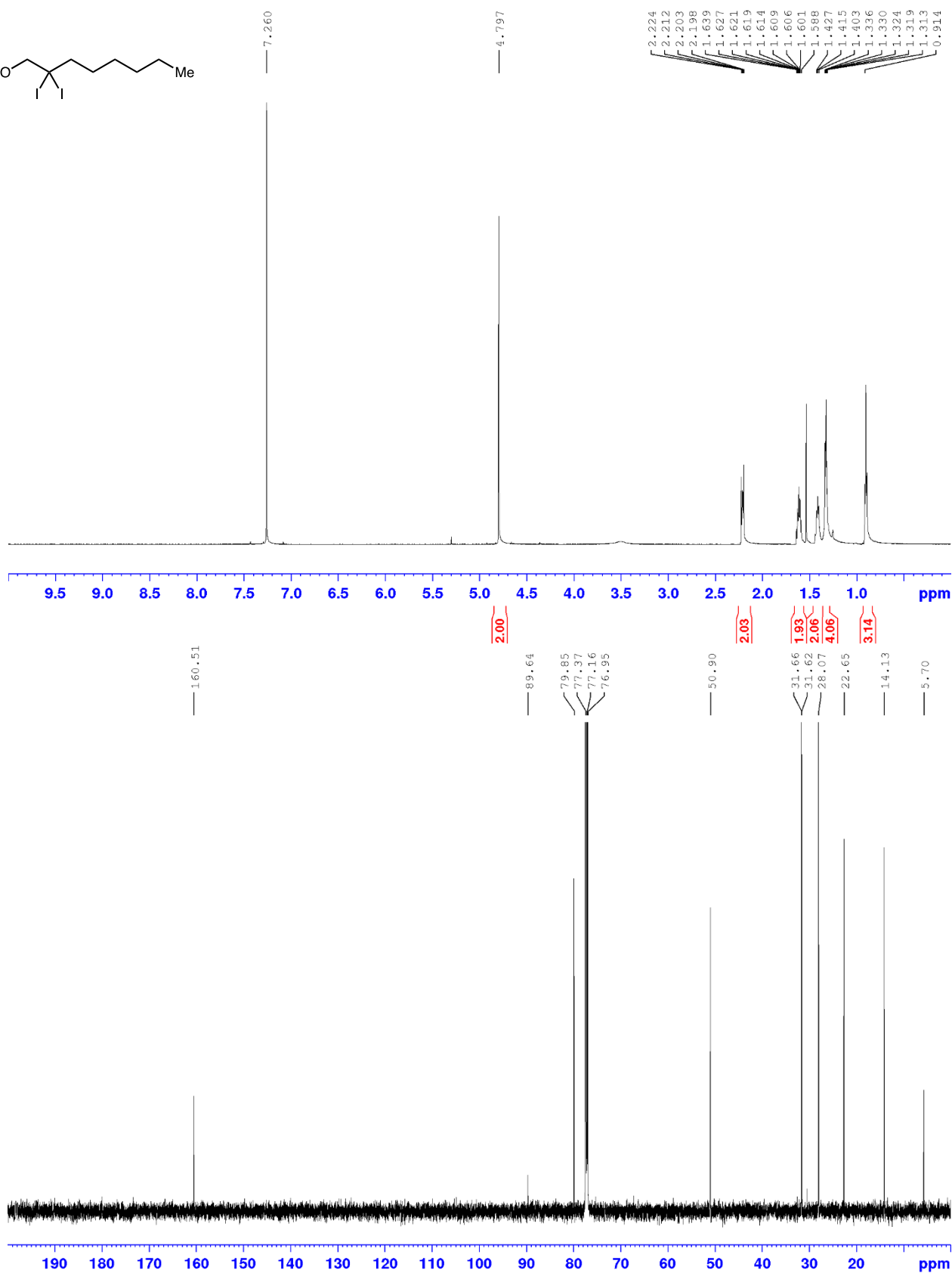

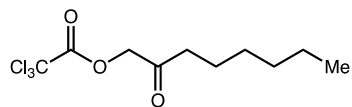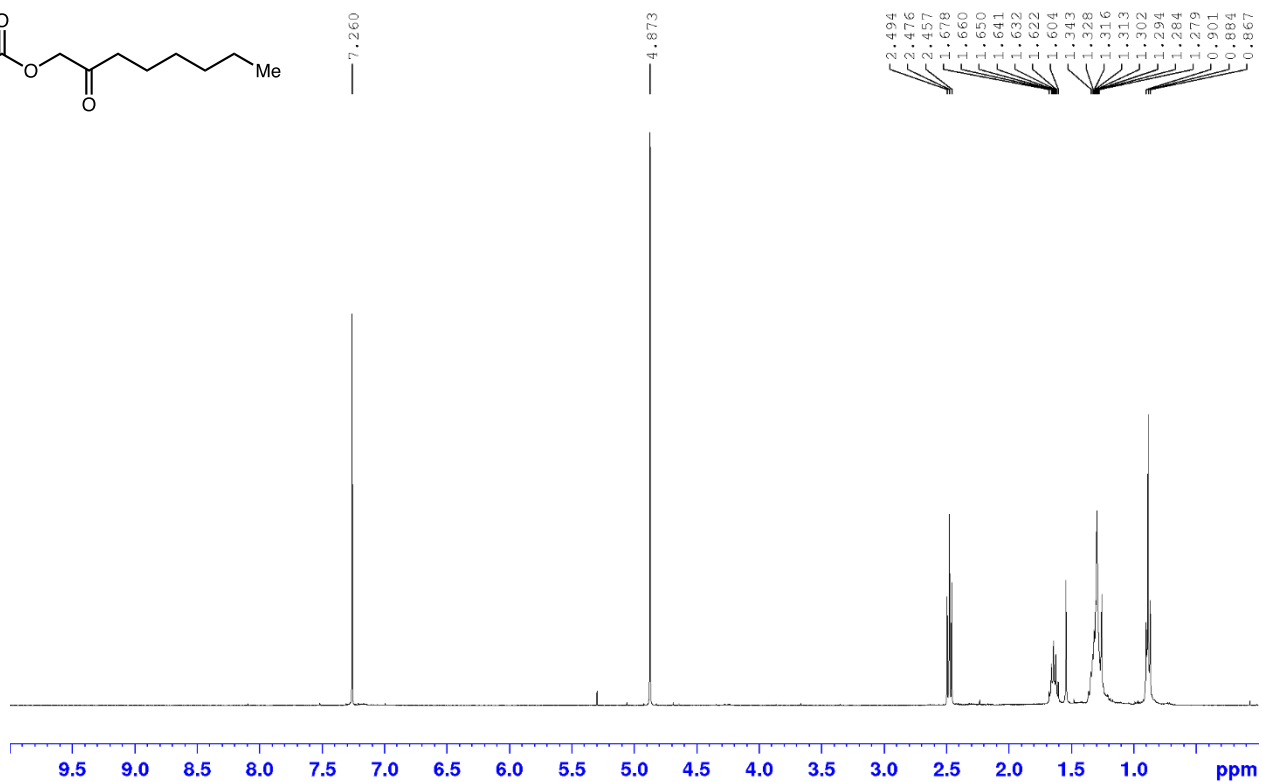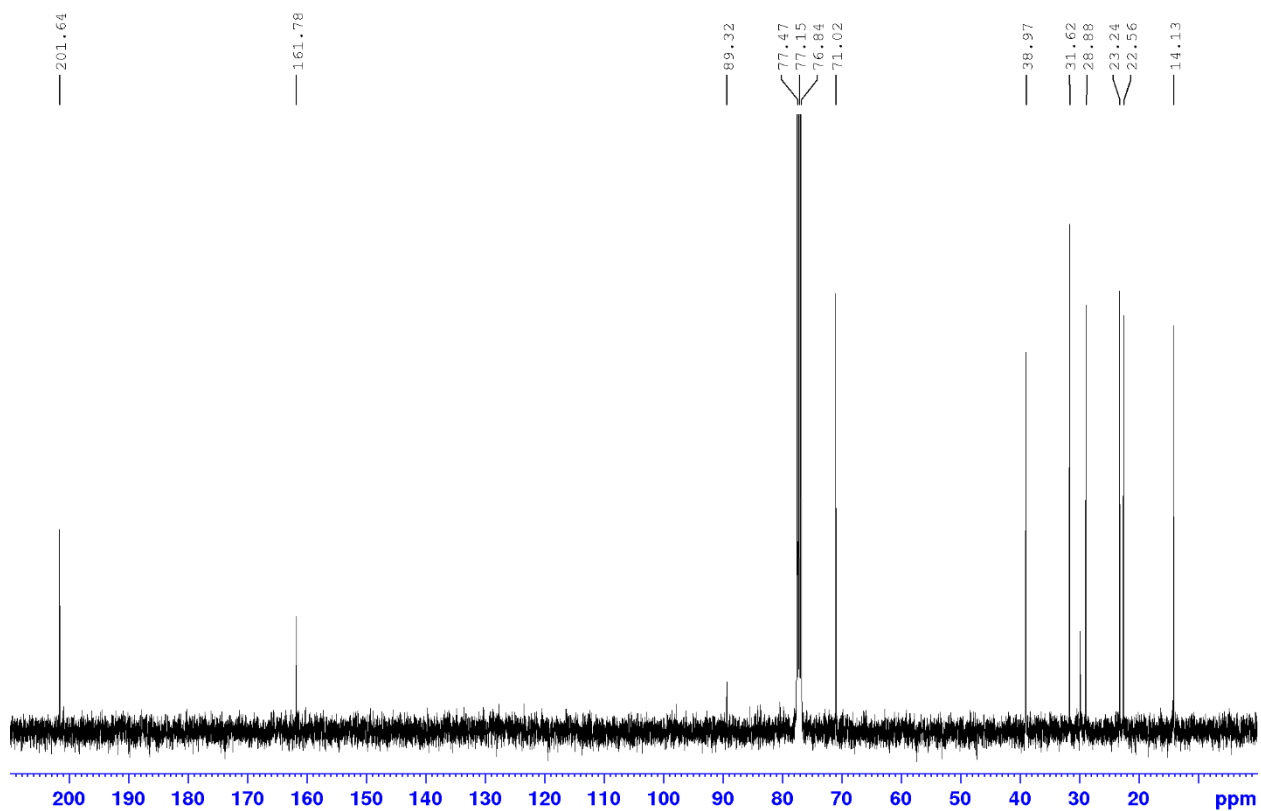

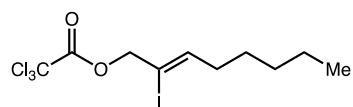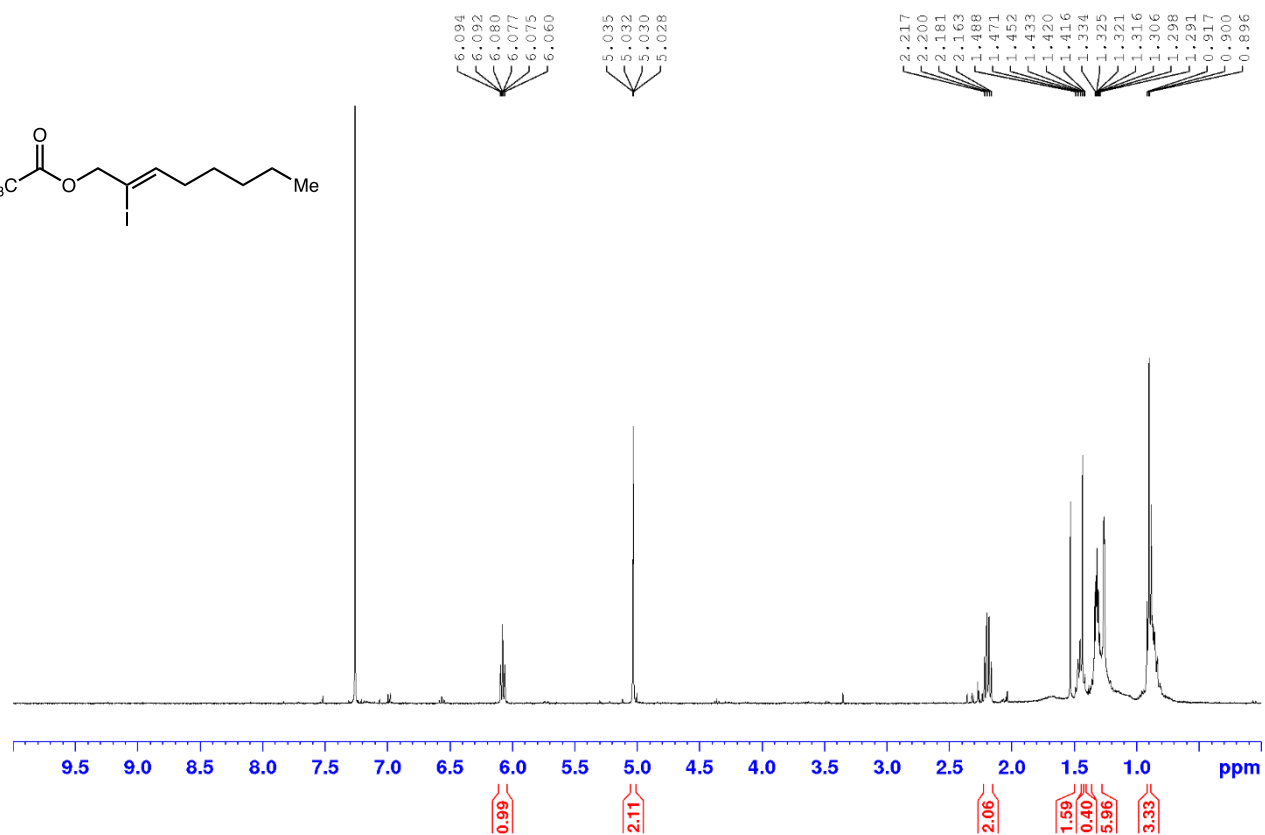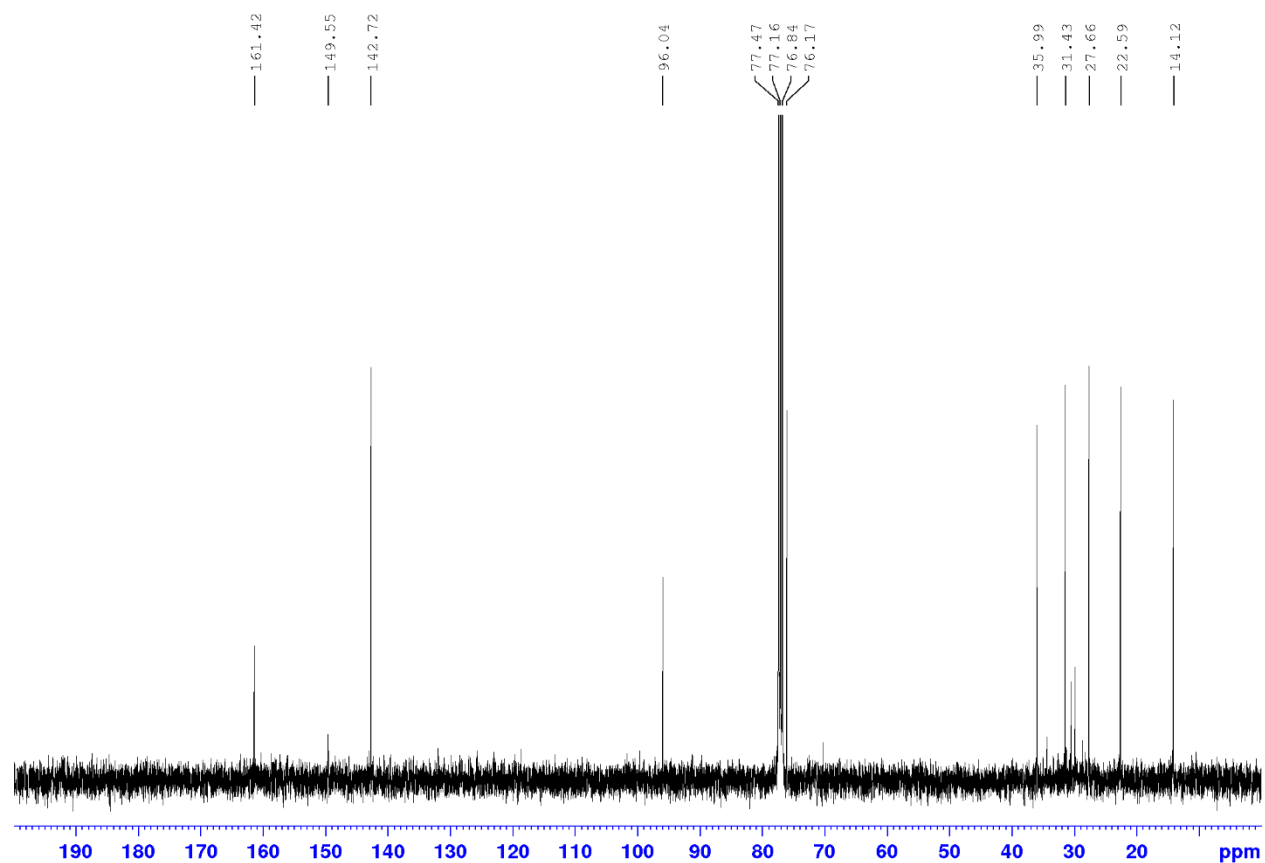

Supplement: Supplementary file 1 [file SC-009-C8SC01214H-s001.pdf]
