# Supplementary material for: β C–H di-halogenation via iterative hydrogen atom transfer
Source: Chem Sci. 2018 Apr 30;9(19):4500–4. doi: 10.1039/c8sc01214h (PMC5958344; doi:10.1039/c8sc01214h)
Supplement: Supplementary file 2 [file SC-009-C8SC01214H-s002.pdf]

Electronic Supplementary Material (ESI) for Chemical Science.
This journal is © The Royal Society of Chemistry 2018

Electronic Supplementary Information for:

β C−H Di-Halogenation via Iterative Hydrogen Atom Transfer

Ethan A. Wappes[†], Avassaya Vanitcha[†], David A. Nagib*

Department of Chemistry and Biochemistry, The Ohio State University, 151 W. Woodruff Ave., Columbus, OH, 43210, United States

Corresponding Author * E-mail: nagib.1@osu.edu

Table of Contents
(with hyperlinks)

I. **General Information**

II. **General Procedure**

III. **Substrate Synthesis**

IV. **Halogenation Optimization**

V. **Di-iodination**

VI. **Di-bromination**

VII. **Mono-chlorination**

VIII. **Post-Synthetic Functionalization**

IX. **Kinetic Experiments**

X. **Intermolecular Halogenation Competition**

XI. **Bond Dissociation Energy Calculations**

XII. **Computation Studies**

   a. **Computational Methods**

   b. **Optimized Cartesian Coordinates**

XIII. **X-ray Crystallographic Data**

XIV. **NMR Spectra**

I. General Information

All chemicals and reagents were purchased from Sigma-Aldrich, Alfa Aesar, Acros, TCI, or ChemImplex. Sodium iodide, sodium bromide and sodium chloride were dried under high vacuum before use. Acetonitrile and triethylamine were distilled over calcium hydride before use. Silicycle F60 (230-400 mesh) silica gel was used or a CombiFlash® Automated Flash Chromatograph for flash column chromatography. Thin layer chromatography (TLC) analyses were performed using Merck silica gel 60 F254 plates and visualized under UV and $KMnO_4$ stain. Melting points were determined using a Thermo Scientific Mel-Temp. ^{1}H and ^{13}C NMR spectra were recorded using a Bruker AVIII 400 or AVIII 600 MHz NMR spectrometer. ^{1}H NMR and ^{13}C NMR chemical shifts are reported in parts per million and referenced with respect to $CDCl_3$ (^{1}H: residual $CHCl_3$ at δ 7.26, ^{13}C: $CDCl_3$ triplet at δ 77.16). ^{1}H NMR and ^{13}C NMR data are reported as chemical shifts (δ ppm), multiplicity (s = singlet, bs = broad singlet, d = doublet, t = triplet, q = quartet, quint = quintet, sex = sextet, m = multiplet, app t = apparent triplet, app q = apparent quartet, app qd = apparent quartet of doublets), coupling constant (Hz), relative integral. High resolution mass spectra were obtained using Bruker MicrOTOF (ESI). IR spectra were recorded using a Thermo Fisher Nicolet iS11 FT-IR and are reported in terms of frequency of absorption (cm^{-1}). Rayonet RPR-100 Photochemical Reactor was used for UV source (300 nm, 16 x RPR-3000A bulbs).

II. General Procedure

Trichloroacetimidate Formation; General Procedure (GP0): To a round-bottom flask containing a stir bar, alcohol (1 equiv.), and CH_2Cl_2 (0.1 M), was added trichloroacetonitrile (1.5 equiv.) and DBU (0.1 equiv.). The solution was stirred and monitored by TLC. Upon completion, the solution was concentrated purified (silica gel, specific eluent conditions noted below).

Di-iodination; General Procedure (GP1): To a 2-dram vial equipped with a PTFE septum cap and magnetic stir bar, was added imidate (1 equiv.), iodobenzene diacetate (3 equiv.) and NaI (3 equiv.). This vial was evacuated and backfilled with N_2 (3x). Dry, degassed dichloromethane and acetonitrile (3:1, 0.2 M) were added to the vial under N_2. The reaction was irradiated with two 26 W compact fluorescent light bulbs and cooled by two fans for 2 hours. Upon completion, the solution was concentrated and purified (silica gel, specific eluent conditions noted below).

Note: Solvent was degassed using a freeze-pump-thaw technique (3x)

Reaction setup and isolation tips

- Sodium iodide must be sufficiently dry to ensure optimum yields in the reaction. Typically, the material was left under high vacuum for at least 24 hours before use.
- Due to product decomposition observed upon prolonged exposure to silica gel, purification requires quick elution of target material with the following techniques.

- Silica gel is loaded with hexanes containing 1% Et_3N to avoid imidate hydrolysis which also results in quicker compound elution. Optimal separation of some di-iodide products requires less Et_3N to ensure slower elution. In this situation, Et_3N should be added only during the loading. This will be noted for specific compounds.
- Most di-iodide products are stable when stored neat at 0 °C unless otherwise noted. Storage at room temperature or in solution causes decomposition over a few days.

Di-bromination; General Procedure (GP2): To a 2-dram vial equipped with a PTFE septum cap and magnetic stir bar, was added imidate (1 equiv.), iodobenzene diacetate (3 equiv.), NaBr (3 equiv.) and Bu_4NBr (1 equiv.). This vial was evacuated and backfilled with N_2 (3x). Dry, degassed hexafluoro-2-propanol and dichloromethane (3:1, 0.2 M) were added to the vial under N_2. The reaction was irradiated with two 26 W compact fluorescent light bulbs and cooled by two fans for 2 hours. Upon completion, the solution was concentrated and purified (silica gel, specific eluent conditions noted below).

Note: Solvent was degassed using a freeze-pump-thaw technique (3x)

Mono-chlorination; General Procedure (GP3): To a 2-dram vial equipped with a PTFE septum cap and magnetic stir bar, was added imidate (1 equiv.), iodobenzene diacetate (3 equiv.), NaCl (3 equiv.) and Bu_4NCl (1 equiv.). This vial was evacuated and backfilled with N_2 (3x). Dry, degassed hexafluoro-2-propanol and dichloromethane (3:1, 0.2 M) were added to the vial under N_2. The reaction was irradiated with ultraviolet light (wavelength 300 nm) with a fan to cool (internal temperature 35 ºC) for 2-24 hours. Upon completion, the solution was concentrated and purified (silica gel, specific eluent conditions noted below).

Note: Solvent was degassed using a freeze-pump-thaw technique (3x)

Setup tip: Reaction seems to be heavily dependent on light setup. With fewer bulbs, poor conversion and mass balance were observed.

III. Substrate Synthesis

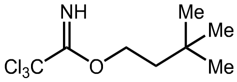

3,3-dimethylbutyl 2,2,2-trichloroacetimidate (S1)

3,3-Dimethyl-1-butanol (1.3 g, 1.5 mL, 12.4 mmol) was subjected to **GP0**. After concentration, the crude mixture was purified (silica gel, 100% hexanes with 1% Et_3N to 10% ethyl acetate/hexanes with 1% Et_3N) to yield imidate **S1** (2.46 g, 80%) as a colorless oil.

R_f: 0.38 (10% ethyl acetate/hexanes)

^{1}H NMR (400 MHz, $CDCl_3$): δ = 8.22 (bs, 1H), 4.34 (t, *J* = 7.1 Hz, 2H), 1.72 (t, *J* = 7.1 Hz, 2H), 0.98 (s, 9H).

^{13}C NMR (150 MHz, $CDCl_3$): δ = 163.3, 91.8, 67.5, 41.6, 29.9, 29.8.

HRMS (ESI-TOF) *m/z*: calc'd for $C_8H_{14}Cl_3NONa$ $[M+Na]^+$ 268.0039 found 268.0070.

IR (film) cm^{-1}: 3346, 2956, 2867, 1661, 1474, 1316, 1292, 1076, 797.

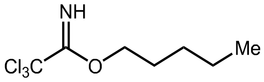

pentyl 2,2,2-trichloroacetimidate (S2)

1-Pentanol (2.0 g, 2.0 mL, 23.7 mmol) was subjected to **GP0**. After concentration, the crude mixture was purified (silica gel, 100% hexanes with 1% Et_3N to 10% ethyl acetate/hexanes with 1% Et_3N) to yield imidate **S2** (3.41g, 62%) as a yellow oil.

R_f: 0.50 (10% ethyl acetate/hexanes)

^{1}H NMR (400 MHz, $CDCl_3$): δ = 8.22 (bs, 1H), 4.29 (t, *J* = 6.6 Hz, 2H), 1.80 – 1.76 (m, 2H), 1.43 – 1.37 (m, 4H), 0.94 – 0.88 (m, 3H).

^{13}C NMR (150 MHz, $CDCl_3$): δ = 163.2, 91.9, 69.8, 28.1 (x2), 22.4, 14.1.

HRMS (ESI-TOF) *m/z*: calc'd for $C_7H_{13}Cl_3NO$ $[M+H]^+$ 232.0063, found 232.0066.

IR (film) cm^{-1}: 3346, 2957, 2932, 2860, 1661, 1467, 1290, 1078, 822.

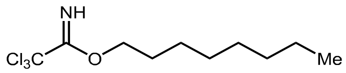

octyl 2,2,2-trichloroacetimidate (S3)

1-Octanol (2.4 g, 3.0 mL, 19.1 mmol) was subjected to **GP0**. After concentration, the crude mixture was purified (silica gel, 100% hexanes with 1% Et_3N to 10% ethyl acetate/hexanes with 1% Et_3N) to yield imidate **S3** (4.60 g, 88%) as a yellow oil.

R_f: 0.50 (10% ethyl acetate/hexanes)

^{1}H NMR (400 MHz, $CDCl_3$): δ = 8.22 (bs, 1H), 4.29 (t, *J* = 6.6 Hz, 2H), 1.78 (q, *J* = 7.0 Hz, 2H), 1.44 (q, *J* = 7.2 Hz, 2H), 1.36 – 1.28 (m, 8H), 0.88 (t, *J* = 6.9 Hz, 3H).

^{13}C NMR (150 MHz, $CDCl_3$): δ = 163.2, 91.9, 69.8, 31.9, 29.3 (x2), 28.4, 26.0, 22.7, 14.2.

HRMS (ESI-TOF) *m/z*: calc'd for $C_{10}H_{18}Cl_3NONa$ $[M+Na]^+$ 296.0352, found 296.0343.

IR (film) cm^{-1}: 3347, 2924, 2855, 1662, 1466, 1305, 1080, 796.

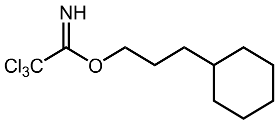

3-cyclohexylpropyl 2,2,2-trichloroacetimidate (S5)

3-Cyclohexyl-1-propanol (0.94 g, 1.0 mL, 6.59 mmol) was subjected to **GP0**. After concentration, the crude mixture was purified (silica gel, 100% hexanes with 1% Et_3N to 10% ethyl acetate/hexanes with 1% Et_3N) to yield imidate **S5** (1.93 g, quant) as a colorless oil.

R_f: 0.50 (20% ethyl acetate/hexanes)

^{1}H NMR (400 MHz, $CDCl_3$): δ = 8.21 (bs, 1H), 4.25 (t, *J* = 6.6 Hz, 2H), 1.80 – 1.61 (m, 7H), 1.32 – 1.10 (m, 6H), 0.92 – 0.82 (m, 2H).

^{13}C NMR (150 MHz, $CDCl_3$): δ = 163.0, 91.9, 70.0, 37.3, 33.5, 33.4 (x2), 26.7, 26.4 (x2), 25.7.

HRMS (ESI-TOF) *m/z*: calc'd for $C_{11}H_{18}Cl_3NONa$ $[M+Na]^+$ 308.0352 found 308.0337.

IR (film) cm^{-1}: 3346, 2920, 2850, 1662, 1448, 1306, 1077, 796.

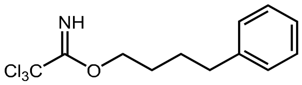

4-phenylbutyl 2,2,2-trichloroacetimidate (S6)

4-Phenylbutan-1-ol (0.80 g, 5.33 mmol) was subjected to **GP0**. After concentration, the crude mixture was purified (silica gel, 100% hexanes with 1% Et_3N to 10% ethyl acetate/hexanes with 1% Et_3N) to yield imidate **S6** (1.38 g, 87%) as a yellow oil.

R_f: 0.47 (10% ethyl acetate/hexanes)

^{1}H NMR (400 MHz, $CDCl_3$): δ = 8.26 (bs, 1H), 7.29 – 7.27 (m, 2H), 7.19 – 7.18 (m, 3H), 4.31 (t, *J* = 6.1 Hz, 2H), 2.69 (t, *J* = 7.3 Hz, 2H), 1.85 – 1.76 (m, 4H).

^{13}C NMR (150 MHz, $CDCl_3$): δ = 163.1, 142.1, 128.49, 128.46, 126.0, 91.8, 69.5, 35.5, 28.0, 27.7.

HRMS (ESI-TOF) *m/z*: calc'd for $C_{12}H_{14}Cl_3NONa$ $[M+Na]^+$ 316.0039 found 316.0039.

IR (film) cm^{-1}: 3341, 3062, 3025, 2928, 2857, 1660, 1495, 1466, 1293, 1077, 795.

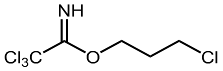

3-chloropropyl 2,2,2-trichloroacetimidate (S7)

3-Chloropropan-1-ol (1.13 g, 1.0 mL, 12.0 mmol) was subjected to **GP0**. After concentration, the crude mixture was purified (silica gel, 100% hexanes with 1% Et_3N to 10% ethyl acetate/hexanes with 1% Et_3N) to yield imidate **S7** (2.16 g, 75%) as a yellow oil.

R_f: 0.58 (10% ethyl acetate/hexanes)

^{1}H NMR (400 MHz, $CDCl_3$): δ = 8.33 (bs, 1H), 4.44 (t, *J* = 5.9 Hz, 2H), 3.68 (t, *J* = 6.4 Hz, 2H), 2.23 (q, *J* = 6.2 Hz, 2H).

^{13}C NMR (150 MHz, $CDCl_3$): δ = 162.8, 91.5, 66.0, 41.2, 31.5.

HRMS (ESI-TOF) *m/z*: calc'd for $C_5H_7Cl_4NONa$ $[M+Na]^+$ 259.9179 found 259.9178.

IR (film) cm^{-1}: 3341, 2966, 1768, 1664, 1288, 1074, 827.

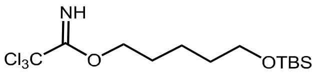

5-((tert-butyldimethylsilyl)oxy)pentyl 2,2,2-trichloroacetimidate (S8)

5-((tert-butyldimethylsilyl)oxy)pentan-1-ol (0.30 g, 1.37 mmol) was subjected to **GP0**. After concentration, the crude mixture was purified (silica gel, 100% hexanes with 1% Et_3N to 10% ethyl acetate/hexanes with 1% Et_3N) to yield imidate **S8** (0.46 g, 93%) as a colorless oil.

R_f: 0.60 (10% ethyl acetate/hexanes)

^{1}H NMR (400 MHz, $CDCl_3$): δ = 8.22 (bs, 1H), 4.29 (t, *J* = 6.5 Hz, 2H), 3.63 (t, *J* = 6.3 Hz, 2H), 1.80 (q, *J* = 7.1 Hz, 2H), 1.60 – 1.55 (m, 2H), 1.52 – 1.46 (m, 2H), 0.89 (s, 9H), 0.04 (s, 6H).

^{13}C NMR (150 MHz, $CDCl_3$): δ = 163.1, 91.8, 69.6, 63.0, 32.5, 28.2, 26.1, 22.4, 18.4, –5.2.

HRMS (ESI-TOF) *m/z*: calc'd for $C_{13}H_{26}Cl_3NO_2SiNa$ $[M+Na]^+$ 384.0696 found 384.0660.

IR (film) cm^{-1}: 3347, 2952, 2928, 2856, 1663, 1471, 1305, 1254, 1082, 832.

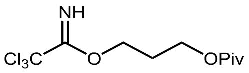

3-(2,2,2-trichloro-1-iminoethoxy)propyl pivalate (S9)

3-Hydroxypropyl pivalate (0.30 g, 1.87 mmol) was subjected to **GP0**. After concentration, the crude mixture was purified (silica gel, 100% hexanes with 1% Et_3N to 10% ethyl acetate/hexanes with 1% Et_3N) to yield imidate **S9** (0.43 g, 75%) as a colorless oil.

R_f: 0.38 (10% ethyl acetate/hexanes)

^{1}H NMR (400 MHz, $CDCl_3$): δ = 8.31 (bs, 1H), 4.39 (t, *J* = 6.2 Hz, 2H), 4.22 (t, *J* = 6.3 Hz, 2H), 2.13 (q, *J* = 6.3 Hz, 2H), 1.22 (s, 3H).

^{13}C NMR (150 MHz, $CDCl_3$): δ = 178.4, 162.8, 91.5, 65.9, 60.7, 38.8, 27.8, 27.3.

HRMS (ESI-TOF) *m/z*: calc'd for $C_{10}H_{16}Cl_3NO_3Na$ $[M+Na]^+$ 326.0093 found 326.0076

IR (film) cm^{-1}: 3344, 2969, 2932, 1726, 1664, 1479, 1282, 1153, 797.

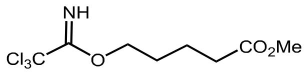

methyl 5-(2,2,2-trichloro-1-iminoethoxy)pentanoate (S10)

Methyl 5-hydroxypentanoate[1] (1.0 g, 7.57 mmol) was subjected to **GP0**. After concentration, the crude mixture was purified (silica gel, 100% hexanes with 1% Et_3N to 10% ethyl acetate/hexanes with 1% Et_3N) to yield imidate **S10** (1.76 g, 84%) as a yellow oil.

R_f: 0.39 (10% ethyl acetate/hexanes)

^{1}H NMR (600 MHz, $CDCl_3$): δ = 8.26 (bs, 1H), 4.30 (t, *J* = 5.9 Hz, 2H), 3.67 (s, 3H), 2.14 (t, *J* = 6.4 Hz, 2H), 1.81 (m, 4H).

^{13}C NMR (150 MHz, $CDCl_3$): δ = 177.8, 163.0, 91.6, 69.1, 51.6, 33.6, 27.8, 21.5.

HRMS (ESI-TOF) *m/z*: calc'd for $C_8H_{12}Cl_3NO_3Na$ $[M+Na]^+$ 297.9780 found 297.9770.

IR (film) cm^{-1}: 3341, 2952, 1734, 1663, 1436, 1294, 1167, 1075, 796.

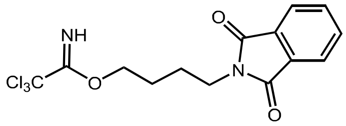

4-(1,3-dioxoisoindolin-2-yl)butyl 2,2,2-trichloroacetimidate (S11)

2-(4-Hydroxybutyl)isoindoline-1,3-dione[2] (0.50 g, 2.28 mmol) was subjected to **GP0**. After concentration, the crude mixture was purified (silica gel, 50% ethyl acetate/hexanes with 1% Et_3N) to yield imidate **S11** (0.83 g, quant) as a white solid

R_f: 0.18 (10% ethyl acetate/hexanes)

^{1}H NMR (600 MHz, $CDCl_3$): δ = 8.25 (bs, 1H), 7.85 – 7.83 (m, 2H), 7.71 – 7.70 (m, 2H), 4.32 (bs, 2H), 3.76 (t, *J* = 6.8 Hz, 2H), 1.85 (t, *J* = 3.2 Hz, 2H).

^{13}C NMR (150 MHz, $CDCl_3$): δ = 169.5, 163.0, 134.1, 132.3, 123.4, 91.7, 68.9, 37.7, 25.9, 25.3.

HRMS (ESI-TOF) *m/z*: calc'd for $C_{14}H_{13}Cl_3N_2O_3Na$ $[M+Na]^+$ 384.9889 found 384.9889.

IR (film) cm^{-1}: 3338, 2949, 1770, 1703, 1663, 1395, 1301, 1075, 1040.

MP: 80 – 82 °C.

[1] Cook, C.; Liron, F.; Guinchard, X.; Roulland, E. *J. Org. Chem.*, **2012**, *77*, 6728.
[2] Wappes, E.A.; Nakafuku, K.M.; Nagib, D.A. *J. Am. Chem. Soc.* **2017**, *139*, 10204.

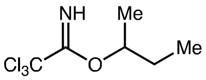

sec-butyl 2,2,2-trichloroacetimidate (S12)

Sec-butanol (0.41 mL, 0.5 mL, 5.5 mmol) was subjected to **GP0**. After concentration, the crude mixture was purified (silica gel, hexanes with 1% Et_3N) to yield imidate **S12** (0.45 g, 38%) as a clear oil.

Note: Imidate is low boiling. Care should be taken while drying to avoid loss of product.

R_f: 0.73 (20% ethyl acetate/hexanes)

^{1}H NMR (400 MHz, $CDCl_3$): δ = 8.20 (bs, 1H), 4.97 (sex, *J* = 6.2 Hz, 1H), 1.80 – 1.62 (m, 2H), 1.33 (d, *J* = 6.3 Hz, 3H), 0.98 (t, *J* = 7.4 Hz, 3H).

^{13}C NMR (100 MHz, $CDCl_3$): δ = 162.6, 92.3, 77.7, 28.7, 18.6, 9.8.

HRMS (ESI-TOF) *m/z*: calc'd for $C_6H_{10}Cl_3NONa$ $[M+Na]^+$ 239.9726, found 239.9736.

IR (film) cm^{-1}: 3345, 2972, 2935, 2879, 1748, 1718, 1659, 1468.

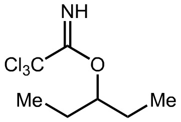

pentan-3-yl 2,2,2-trichloroacetimidate (S13)

3-Pentanol (0.82 g, 1 mL, 9.25 mmol) was subjected to **GP0**. After concentration, the crude mixture was purified (silica gel, 100% hexanes with 1% Et_3N to 10% ethyl acetate/hexanes with 1% Et_3N) to yield imidate **S13** (1.39 g, 65%) as a colorless oil.

R_f: 0.66 (10% ethyl acetate/hexanes)

^{1}H NMR (600 MHz, $CDCl_3$): δ = 8.19 (bs, 1H), 4.92 – 4.90 (m, 1H), 1.73 – 1.71 (m, 4H), 0.98 – 0.96 (m, 6H).

^{13}C NMR (150 MHz, $CDCl_3$): δ = 162.8, 92.4, 82.0, 25.9, 9.5.

HRMS (ESI-TOF) *m/z*: calc'd for $C_7H_{12}Cl_3NONa$ $[M+Na]^+$ 253.9882 found 253.9886.

IR (film) cm^{-1}: 3348, 2969, 2939, 2880, 1658, 1290, 1109, 1075.

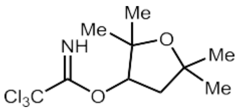

2,2,5,5-tetramethyltetrahydrofuran-3-yl 2,2,2-trichloroacetimidate (S14)

Step 1: Ketone reduction

To solution of 2,2,5,5-tetramethyldihydrofuran-3-one (1.85 g, 2 mL, 13.0 mmol) in methanol was slowly added $NaBH_4$ (0.99 g, 26.0 mmol) at 0 °C. The mixture was warmed to room temperature and stirred for 5 h. Upon completion (monitored by TLC), the reaction was quenched with 1 M HCl (5 mL) slowly at 0 °C and diluted with EtOAc (20 mL) and H_2O (20 mL). The aqueous phase was extracted with EtOAc (3x20 mL). The combined organic layers were washed with brine (2x 20 mL), dried over $MgSO_4$ and concentrated under reduce pressure. The crude mixture of 2,2,5,5-tetramethyltetrahydrofuran-3-ol was used in next step.

Step 2: Trichloroacetimidate formation

2,2,5,5-Tetramethyltetrahydrofuran-3-ol (1.9 g, 13.0 mmol) was subjected to **GP0**. After concentration, the crude mixture was purified (silica gel, 100% hexanes with 1% Et_3N to 10% ethyl acetate/hexanes with 1% Et_3N) to yield imidate **S14** (1.98 g, 52% over two steps) as a colorless oil.

R_f: 0.45 (10% ethyl acetate/hexanes)

^{1}H NMR (400 MHz, $CDCl_3$): δ = 8.29 (bs, 1H), 5.17 (dd, *J* = 5.9, 2.7 Hz, 1H), 2.37 (dd, *J* = 14.1, 5.9 Hz, 1H), 2.09 (dd, *J* = 14.1, 2.7 Hz, 1H), 1.36 (s, 3H), 1.35 (s, 3H), 1.34 (s, 3H), 1.30 (s, 3H).

^{13}C NMR (150 MHz, $CDCl_3$): δ = 162.1, 92.4, 85.9, 81.6, 32.4, 30.0, 29.4, 26.4, 14.6.

HRMS (ESI-TOF) *m/z*: calc'd for $C_{10}H_{16}Cl_3NO_2Na$ $[M+Na]^+$ 310.0144 found 310.0131.

IR (film) cm^{-1}: 3343, 2974, 1661, 1382, 1079, 752.

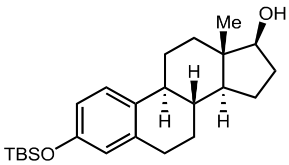

3- (*tert*-butyldimethylsilyl)oxy)-estradiol

Estradiol (1 g, 3.6 mmol) was added to a flask along with NaH (60% in mineral oil, 184 mg, 4.6 mmol) and a magnetic stir bar. The flask was evacuated and backfilled with N_2 diluted with THF (5 mL, 0.7 M) and stirred for 30 minutes. TBSCl (0.6 g, 4.0 mmol) was then added and allowed to stir for 3 hours. Finally, the solution was quenched with H_2O and extracted with CH_2Cl_2. The crude material was carried forward with a slight impurity.

Spectroscopic data is consistent with reported literature data.[3]

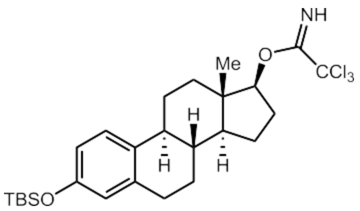

3-((*tert*-butyldimethylsilyl)oxy)-17- trichloroacetimidatyl estradiol (S15)

3-(*Tert*-butyldimethylsilyl)oxy)-esteradiol (0.4 g, 1.03 mmol) was subjected to **GP0**. After concentration, the crude mixture was purified (silica gel, 100% hexanes with 1% Et_3N to 10% ethyl acetate/hexanes with 1% Et_3N) to yield imidate **S15** (0.40 g, 73%) as a white solid.

R_f: 0.47 (10% ethyl acetate/hexanes)

^{1}H NMR (400 MHz, $CDCl_3$): δ = 8.20 (bs, 1H), 7.12 (d, *J* = 8.4 Hz, 1H), 6.61 (dd, *J* = 8.4 Hz, 2.5 Hz, 1H), 6.56 (s, 1H), 4.82 (t, *J* = 8.3 Hz, 1H), 2.82 (d, *J* = 5.0 Hz, 2H), 2.80 – 2.21 (m, 3H), 2.01 – 1.66 (m, 4H), 1.65 – 1.26 (m, 7H), 0.98 (s, 9H), 0.94 (s, 3H), 0.19 (s, 6H).

^{13}C NMR (150 MHz, $CDCl_3$): δ = 163.2, 153.6, 137.9, 133.1, 126.3, 120.1, 117.4, 92.2, 87.7, 49.9, 44.1, 43.7, 38.7, 37.3, 29.8, 27.5, 27.2, 26.4, 25.9, 23.5, 18.4, 12.3, – 4.2.

HRMS (ESI-TOF) *m/z*: calc'd for $C_{26}H_{38}Cl_3NO_2SiNa$ $[M+Na]^+$ 552.1635 found 552.1626.

IR (film) cm^{-1}: 3344, 2930, 2858, 1661, 1496, 1470, 1294, 1252, 878.

MP: 152 – 153 °C.

[3] Top, S.; Jaouen, G.; Vessières, A.; Abjean, J.-P.; Davoust, D.; Rodger, C.A.; Sayer, B.G.; McGlinchey, M.J. *Organometallics* **1985**, *4*, 2130.

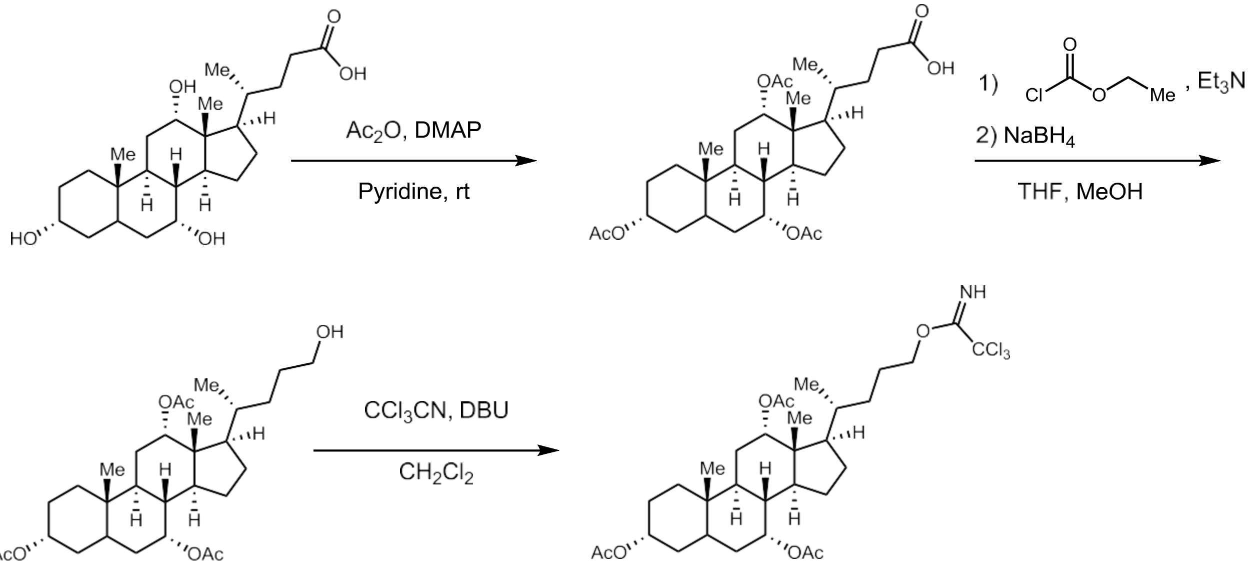

3,7,12-triacetoxy-5-cholanyl-2,2,2-trichloroacetimidate (S16)

Step 1: acylation

To a solution of cholic acid (0.80 g, 2.0 mmol) in pyridine (4 mL) was added DMAP (20 mg, 0.16 mmol) and finally acetic anhydride (4.3 g, 4 mL, 42.4 mmol). The mixture was stirred at room temperature for 18 h. Upon completion (monitored by TLC), saturated solution ammonium chloride (20 mL) and dichloromethane (20 mL) was added to the mixture. The aqueous phase was extracted with dichloromethane (3x20 mL). The combined organic layers were washed with brine (2x 20 mL), dried over $MgSO_4$ and concentrated under reduce pressure. The crude mixture was purified (silica gel, 4:1 to 1:1 hexanes/ethyl acetate) to yield carboxylic acid (0.88 g, 82%) as a white solid.

Spectroscopic data is consistent with reported literature data.[4]

R_f: 0.13 (25% ethyl acetate/hexanes)

^{1}H NMR (400 MHz, $CDCl_3$): δ = 5.09 (S, 1H), 4.91 (d, *J* = 2.6 Hz, 1H), 4.60 – 4.54 (m, 1H), 2.55 – 2.44 (m, 1H), 2.39 – 2.33 (m, 1H), 2.21 (s, 1H), 2.14 (s, 3H), 2.08 (s, 3H), 2.04 (s, 3H), 2.00 – 1.78 (m, 7H), 1.66 – 1.59 (m, 5H), 1.51 – 1.27 (m, 7H), 1.10 – 1.06 (m, 2H), 0.92 (s, 3H), 0.82 (dd, *J* = 6.3, 1.6 Hz, 3H), 0.73 (s, 3H).

^{13}C NMR (150 MHz, $CDCl_3$): δ = 178.5, 170.5, 170.4, 170.3, 75.4, 74.1, 70.7, 60.4, 47.4, 45.1, 43.4, 41.0, 37.8, 34.8, 34.7, 34.5, 34.4, 32.3, 32.2, 31.3, 30.8, 30.6, 30.1, 28.9, 27.2, 26.9, 25.6, 22.8, 22.6, 21.6, 21.4, 21.4, 17.5, 14.2, 12.3.

[4] Zígolo, A. M.; Liñares, G. G.; Baldessari, A. *Steroids*, **2016**, *107*, 10.

Step 2: Reduction of carboxylic acid

To a solution of 3,7,12-triacetoxy-5-cholanic acid (1.23 g, 2.30 mmol) and Et_3N (30 mg, 0.42 mL, 11.04 mmol) in THF (6 mL) was added ethyl chloroformate (33 mg, 0.29 mL, 3.06 mmol) at room temperature. After stirring for 2 h, $NaBH_4$ (0.44 g, 11.63 mmol) and then methanol (1 mL) was added gradually at 0 ^oC until a clear solution was obtained. After stirring at 0 ^oC for 2 h, the reaction mixture was diluted with water (10 mL) and EtOAc (10 mL). The aqueous phase was extracted with EtOAc (2x10 mL). The combined organic layers were washed with brine (2x 20 mL), dried over $MgSO_4$ and concentrated under reduce pressure. The crude mixture was purified (silica gel, 4:1 to 1:1 hexanes/ethyl acetate) to yield 3,7,12-triacetoxy-5-cholan-24-ol (0.82 g, 68%) as a yellow oil.

Spectroscopic data is consistent with reported literature data.[5]

R_f: 0.13 (25% ethyl acetate/hexanes)

^{1}H NMR (400 MHz, $CDCl_3$): δ = 5.09 (bs,1H), 4.91 (d, *J* = 2.7 Hz, 1H), 4.60 – 4.55 (m, 1H), 3.63 – 3.58 (m, 2H), 2.13 (s, 3H), 2.08 (s, 3H), 2.04 (s, 3H), 1.93 – 1.80 (m, 4H),1.76 – 1.54 (m, 7H), 1.52 – 1.38 (m, 5H), 1.28 – 1.25 (m, 3H), 1.12 – 1.06 (m, 2H), 0.92 (s, 3H), 0.83 (d, *J* = 6.5 Hz, 3H), 0.73 (s, 3H).

^{13}C NMR (150 MHz, $CDCl_3$): δ = 170.6, 170.4, 75.5, 74.1, 70.8, 63.3, 47.5, 45.0, 43.4, 41.0, 37.8, 34.8, 34.7, 34.6, 34.3, 31.7, 31.3, 29.1, 28.9, 27.3, 26.9, 25.6, 22.8, 22.6, 21.6, 21.5, 21.4, 17.9, 12.2.

Step 3: Trichloroacetimidate formation

3,7,12-Triacetoxy-5-cholan-24-ol (0.82 g, 1.57 mmol) was subjected to **GP0**. After concentration, the crude mixture was purified (silica gel, 7: 3 to 1:1 ethyl acetate/hexanes with 1% Et_3N) to yield imidate **S16** (0.51 g, 49%) as a white solid.

R_f: 0.20 (25% ethyl acetate/hexanes)

^{1}H NMR (400 MHz, $CDCl_3$): δ = 8.22 (s, 1H), 5.09 (s, 1H), 4.90 (bs, 1H), 4.57 – 4.54 (m, 1H), 4.25 – 4.24 (m, 2H), 2.12 (s, 3H), 2.07 (s, 3H), 2.03 (s, 3H), 1.95 – 1.92 (m, 1H), 1.84 – 1.81 (m, 3H), 1.80 – 1.72 (m, 2H), 1.67 – 1.55 (m, 7H), 1.52 – 1.48 (m, 3H), 1.40 – 1.39 (m, 2H), 1.29 – 1.22 (m, 2H), 1.14 – 1.05 (m, 3H), 0.90 (s, 3H), 0.83 (d, *J* = 6.5 Hz, 3H), 0.72 (s, 3H).

^{13}C NMR (150 MHz, $CDCl_3$): δ = 170.6, 170.6, 170.4, 163.2, 91.8, 75.6, 74.2, 70.9, 70.0, 47.8, 45.2, 43.5, 41.1, 38.0, 34.9, 34.8 (x2), 34.5, 31.9, 31.4, 29.1, 27.4, 27.1, 25.7, 25.0, 23.0, 22.7, 21.7, 21.6, 21.5, 18.0, 12.4.

HRMS (ESI-TOF) *m/z*: calc'd for $C_{32}H_{48}Cl_3NO_7Na$ $[M+Na]^+$ 686.2394, found 686.2386.

[5] Ogawa, S.; Zhou,B., Kimoto, Y.; Omura, K.; Kobayashi, A.; Higashi, T.; Mitmura, K.; Ikegawa, S.; Hagey, L. R.; Hofmann, A. F.; Iida, T. *Steroids*, **2013**, *78*, 927.

IR (film) cm^{-1}: 2945, 2254, 1722, 1663, 1377, 1364, 1247, 1023, 908, 727.

MP: 69 – 70 °C.

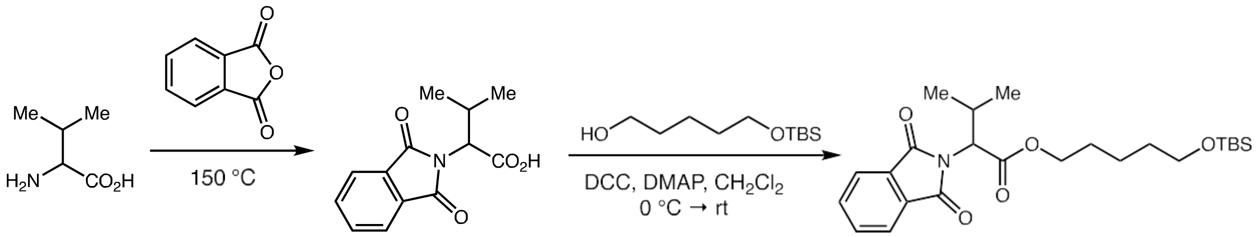

5-((*tert*-butyldimethylsilyl)oxy)pentyl 2-(1,3-dioxoisoindolin-2-yl)-3-methylbutanoate

Step 1: Phthalimide protection

Valine (2 g, 17 mmol) was comined with phthalic anhydride (2.53 g, 17 mmol) in a flask containing a magnetic stir bar. The mixture was stirred at 150 °C for 30 minutes while water was steadily liberated from the flask. Upon completion, the mixture was allowed to cool. The resulting solid was analytically pure and carried forward without further purification.

R_f: 0.17 (5% MeOH/CH_2Cl_2)

^{1}H NMR (600 MHz, $CDCl_3$): 7.87 (dd, *J* = 5.3, 3.1 Hz, 2H), 7.75 (dd, *J* = 5.5, 3.0 Hz, 2H), 4.63 (d, *J* = 8.5 Hz, 1H), 2.80 – 2.73 (m, 1H), 1.17 (d, *J* = 6.6 Hz, 3H), 0.92 (d, *J* = 6.8 Hz, 3H).

^{13}C NMR (150 MHz): 173.0, 167.9, 134.5, 131.8, 123.8, 57.9, 28.6, 21.0, 19.6.

Step 2: Steglich esterification

Phthalimide-protected valine (2.11 g, 8.5 mmol), alcohol (1.86 g, 8.5 mmol), and 4-(Dimethylamino)pyridine (0.1 g, 0.8 mmol) were dissolved in dichloromethane (40 mL) and cooled to 0 °C. Finally, N,N'-dicyclohexylcarbodiimide (DCC) (1.85 g, 9.0 mmol) was added and the reaction was stirred until consumption of alcohol (monitored by TLC). Upon completion, the reaction was concentrated purified (silica gel, 5% ethyl acetate/hexanes). The target material was isolated with a slight impurity and carried forward without further purification.

R_f: 0.51 (20% ethyl acetate/hexanes)

^{1}H NMR (400 MHz, $CDCl_3$): δ = 7.87 (dd, *J* = 5.4, 3.0 Hz, 2H), 7.74 (dd, *J* = 5.5, 3.0 Hz, 2H), 4.56 (d, *J* = 8.2 Hz, 1H), 4.14 – 4.10 (m, 2H), 3.53 – 3.49 (m, 2H), 2.79 – 2.73 (m, 1H), 1.61 – 1.57 (m, 2H), 1.48 – 1.43 (m, 2H), 1.31 – 1.26 (m, 2H), 1.15 (d, *J* = 6.8 Hz, 3H), 0.91 (d, *J* = 6.8 Hz, 3H), 0.86 (s, 9H), 0.1 (s, 6H).

^{13}C NMR (100 MHz, CDCl$_3$): δ = 169.0, 167.9, 134.3, 132.0, 123.6, 65.7, 63.0, 58.0, 32.4, 28.7, 28.4, 26.1, 22.4, 21.1, 19.6, 14.4, –5.2.

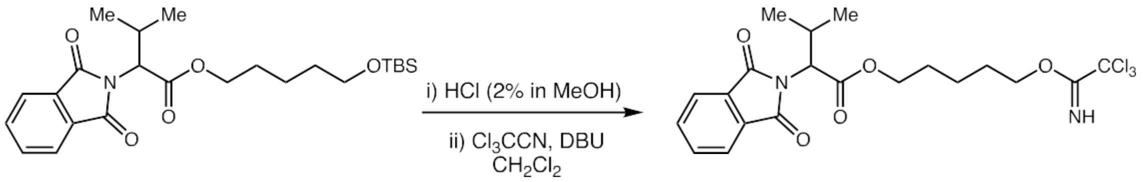

5-(2,2,2-trichloro-1-iminoethoxy)pentyl 2-(1,3-dioxoisoindolin-2-yl)-3-methylbutanoate (S17)

Step 1: Desilylation

The TBS-protected alcohol was stirred in 150 mL of MeOH containing 3 mL of concentrated HCl. Upon consumption of starting material (monitored by TLC), the reaction was partitioned between ethyl acetate and brine and washed with ethyl acetate (2 x 50 mL). The combined organic material was dried over $MgSO_4$ and concentrated then carried forward without further purification.

Step 2: Trichloroacetimidate formation

Crude alcohol was subjected to **GP0**. After concentration, the crude mixture was purified (silica gel, 5% ethyl acetate/hexanes with 1% Et_3N) to yield trichloroacetimidate **S17** (2.3 g, 56% over 3 steps) as a clear oil.

R_f: 0.32 (20% ethyl acetate/hexanes)

^{1}H NMR (400 MHz, CDCl$_3$): δ = 8.22 (bs, 1H), 7.87 (dd, *J* = 5.5, 3.0 Hz, 2H), 7.75 (dd, *J* = 5.5, 3.1 Hz, 2H), 4.56 (d, *J* = 8.3 Hz, 1H), 4.19 (t, *J* = 6.4 Hz, 2H), 4.15 (td, *J* = 6.6, 3.8 Hz, 2H), 2.80 – 2.72 (m, 1H), 1.75 – 1.61 (m, 4H), 1.44 – 1.39 (m, 2H), 1.15 (d, *J* = 6.7 Hz, 3H), 0.91 (d, *J* = 6.8 Hz, 3H).

^{13}C NMR (100 MHz, CDCl$_3$): δ = 168.9, 167.9, 163.0, 134.3, 131.8, 123.6, 91.6, 69.3, 65.3, 57.8, 28.7, 28.1, 27.8, 22.4, 21.1, 19.6.

HRMS (ESI-TOF) *m/z*: calc'd for $C_{20}H_{23}Cl_3N_2O_5Na$ [M+Na]$^+$ 499.0570, found 499.0561.

IR (film) cm^{-1}: 3341, 2962, 2938, 2872, 1712, 1665, 1468.

IV. Halogenation Optimization

Optimization of Di-iodination

Imidate **S3** (0.2 mmol) was subjected to **GP1**. With changes based upon the following tables. Upon completion, the crude mixture was concentrated. A crude yield of di-iodoimidate was determined via ^{1}H NMR (Isopropyl acetate as an internal standard).

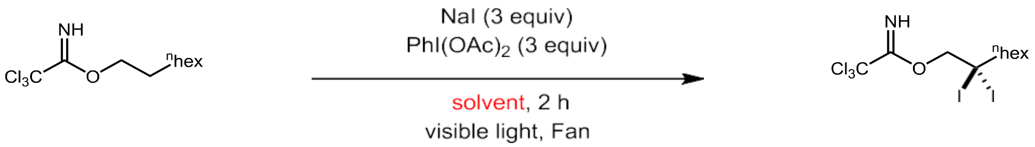

Table S1: Solvent effects.

Entry	Solvent (M)	Dielectric constant[6,7]	Yield
1	MeCN (0.1 M)	36.64	29%
2	CH_2Cl_2 (0.2 M)	9.08	29%
**3**	**MeCN (0.2 M)**	**36.64**	**58%**
4	C_6H_6 (0.2 M)	2.28	56%
5	HFIP (0.2 M)	17.8	0%

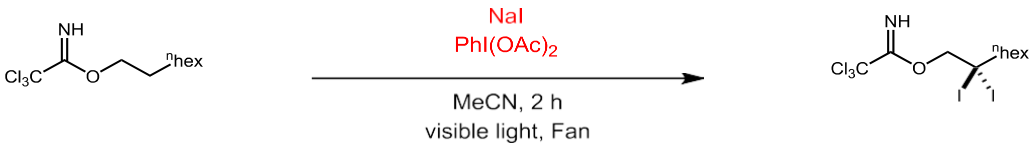

Table S2: Effect of oxidant equivalents and ratio.

Entry	NaI (equiv)	$PhI(OAc)_2$ (equiv)	TM	SM
1	2	2	52%	0%
2	2	3	23%	40%
3	2	4	11%	76%
**4**	**3**	**3**	**55%**	**0%**
5	4	3	10%	0%

[6] Vogel's Practical Organic Chemistry (5th ed.), *J. Phys. Chem. B.* **2001**, *105*, 139.
[7] T = 20 °C

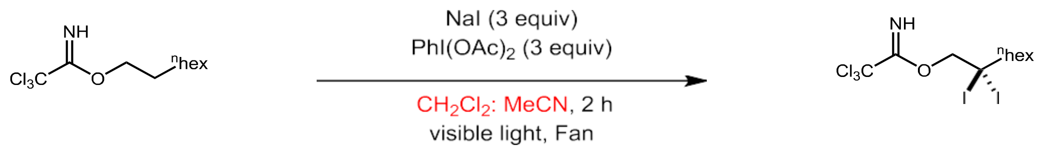

Table S3: Effect of solvent ratio.

Entry	CH_2Cl_2: MeCN	Yield
1	0:1	58%
2	1:1	62%
3	7:3	73%
**4**	**3:1**	**88%**
5	4:1	65%
6	9:1	55%
7	1:0	29%

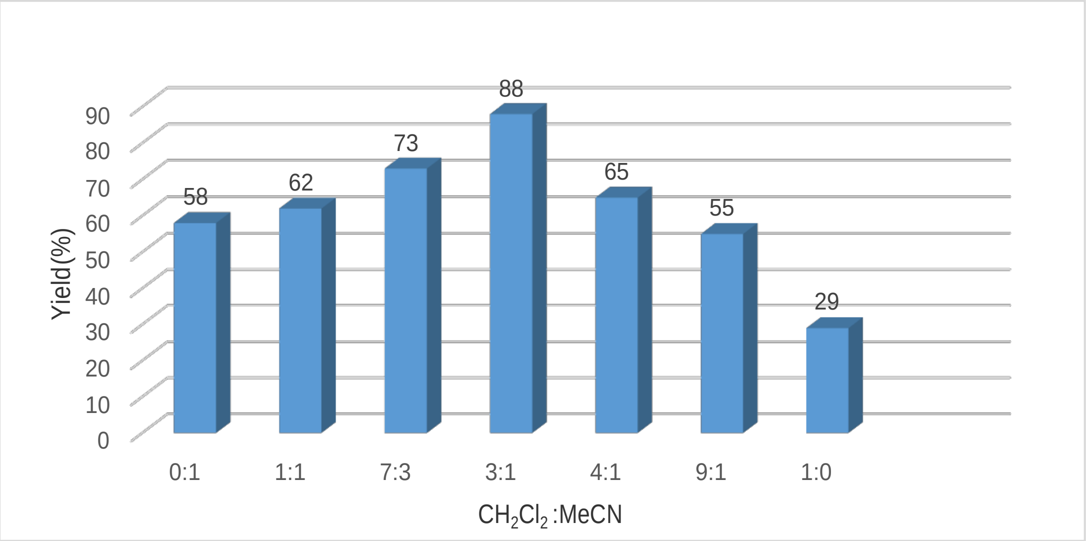

Figure S1: Optimization of solvent mixture.

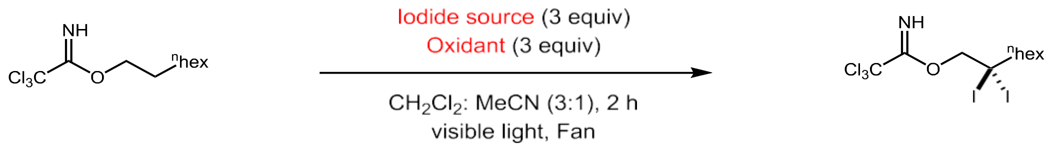

Table S4: Effect of iodide source and oxidant.

Entry	Iodide source (3 equiv)	Oxidant (3 equiv)	Yield
**1**	**NaI**	**$PhI(OAc)_2$**	**88%**
2	CsI	$PhI(OAc)_2$	24%
3	Bu_4NI	$PhI(OAc)_2$	17%
4	NaI + Bu_4NI (1 equiv)	$PhI(OAc)_2$	39%
5[a]	NIS (4 equiv)	–	14%
6	NaI	PhI(*m*CBA)$_2$	69%
7	NaI	$PhI(OPiv)_2$	60%
8	NaI	$PhI(CF_3CO_2)_2$	3%

[a] Irradiated with blue LED (460 nm).

Optimization of Di-bromination

Imidate **S1** (0.2 mmol) was subjected to **GP2**. Upon completion, the crude mixture was concentrated. A crude yield of di-bromoimidate was determined via ^{1}H NMR (Isopropyl acetate as an internal standard).

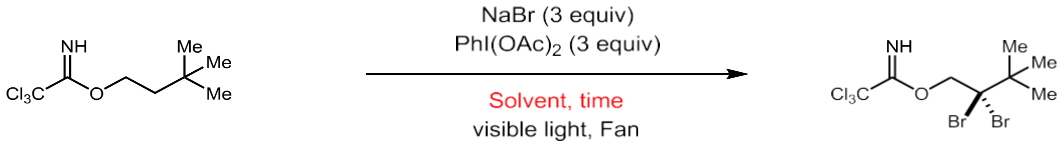

Table S5: Solvent effects.

Entry	Time (h)	Solvent	Yield
1	2	CH_2Cl_2:MeCN (3:1)	34%
2	24	CH_2Cl_2:MeCN (3:1)	47%
3	24	MeCN	23%
4	3	MeCN (50 ^{o}C)	10%
5	24	CH_2Cl_2	0%
6	24	HFIP	10%
**7**	**24**	**CH_2Cl_2:HFIP (1:3)**	**45%**

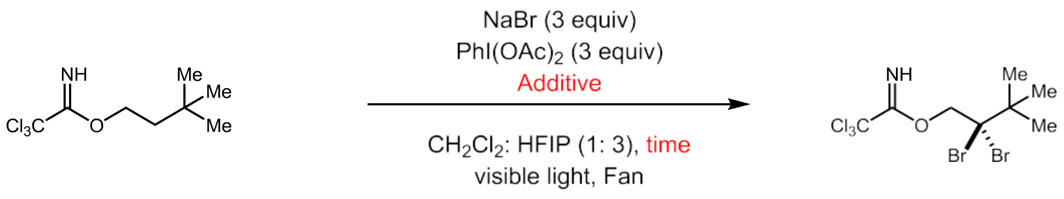

Table S6: Assessing the effect of additives.

Entry	Additive	Time	Yield[a]
1	none	24	45%
2	Bu_4NBr (1 equiv)	24	70% (72%)
**3**	**Bu_4NBr (1 equiv)**	**2**	**quant (92%)**
4	Bu_4NBr (3 equiv) without NaBr	24	13%, (mono-Br 32%)

[a] Isolated yield indicated in parenthesis.

Optimization of Mono-Chlorination

Imidate **S1** (0.2 mmol) was subjected to **GP3**. Upon completion, the crude mixture was concentrated. A crude yield of di-chloroimidate was determined via ^{1}H NMR (Isopropyl acetate as an internal standard).

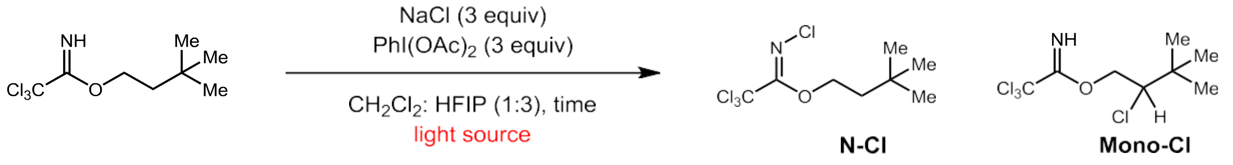

Table S7: Optimization of light source.

Entry	Light	Time (h)	Mono-Cl	N-Cl
1	White CFL 26 W	24	0%	0%
2	White CFL 26 W (0.5 equiv Bu_4NCl)	24	12%	45%
3	Blue LED (460 nm)	2	9%	74%
**4**	**UV (300 nm)**	**2**	**19%**	**0%**

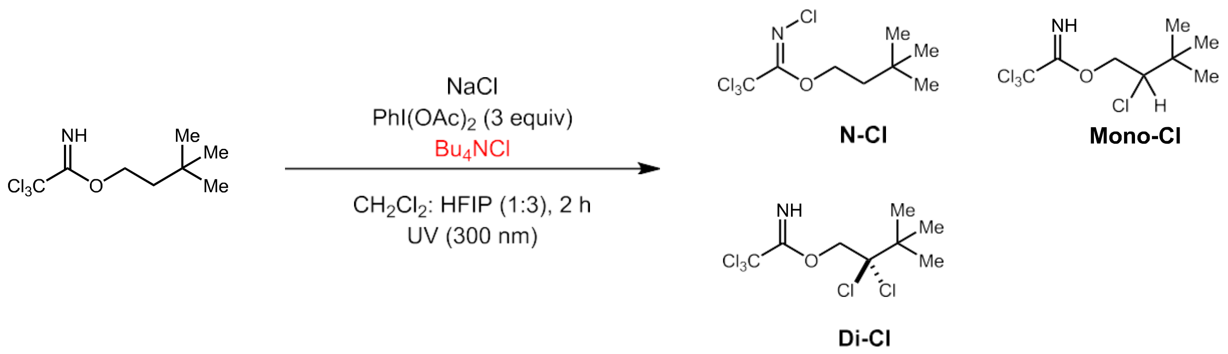

Table S8: Assessing the effect of Bu_4NCl.

Entry	NaCl (equiv)	Bu_4NCl (equiv)	N-Cl	Mono-Cl[a]	Di-Cl	SM
1	3	none	0%	19%	0%	57%
**2**	**3**	**1**	**0%**	**68% (53%)**	**3%**	**22%**
3	2.5	0.5	0%	23%	0%	41%
4	2	1	0%	65%	7%	25%
5	1.5	1.5	0%	59% (47%)	4%	38%
6	1	2	0%	40%	0%	17%
7	0	3	0%	0%	0%	70%

[a] Isolated yield indicated in parenthesis.

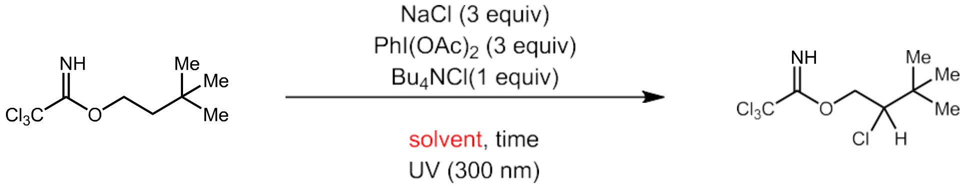

Table S9: Solvent effects.

Entry	Time	Solvent	Yield
**1**	**2**	**CH_2Cl_2:HFIP (1:3)**	**68%**
2	24	CH_2Cl_2:MeCN (3:1)	37%
3	24	CH_2Cl_2:MeCN (1:3)	32%

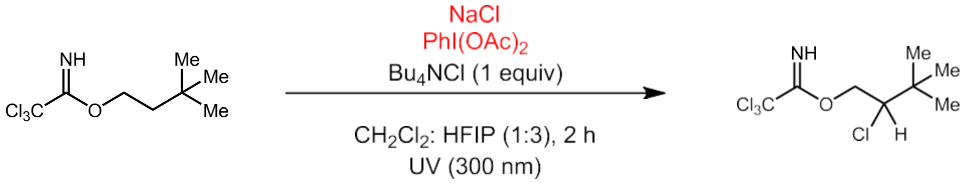

Table S10: Effect of oxidant equivalents and ratios.

Entry	NaCl (equiv)	$PhI(OAc)_2$ (equiv)	Yield
**1**	**3**	**3**	**68%**
2	3	4	43%
3	3	5	7%
4	5	5	60%
5	6	6	10%

V. Di-iodination

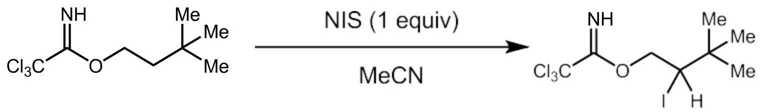

2-iodo-3,3-dimethylbutyl 2,2,2-trichloroacetimidate (1)

To a 2-dram vial equipped with a PTFE septum cap and magnetic stir bar, was added imidate **S1** (49 mg, 0.20 mmol) and *N*-Iodosuccinimide (45 mg, 0.20 mmol). This vial was evacuated and backfilled with N_2 (3x). Dry, degassed acetonitrile (1 mL) was added to the vial under N_2. The reaction was irradiated with two 23 W compact fluorescent light bulbs for 2 hours. Upon completion, the solution was concentrated. A crude yield of mono-iodoimidate **1** (55%) and di-iodoimidate **4** (21%) (mono:di 3:1) was determined via ^{1}H NMR (Isopropyl acetate as an internal standard).

^{1}H NMR (400 MHz, $CDCl_3$): δ = 8.34 (bs, 1H), 4.60 (d, *J* = 6.0 Hz, 2H), 4.35 – 4.33 (m, 1H), 2.0 (s, 9H).

^{13}C NMR (100 MHz, $CDCl_3$): δ = 163.1, 92.0, 72.6, 67.4, 35.0, 28.9.

HRMS (ESI-TOF) *m/z*: calc'd for $C_8H_{13}Cl_3INONa$ $[M+Na]^+$ 393.9005, found 393. 9017.

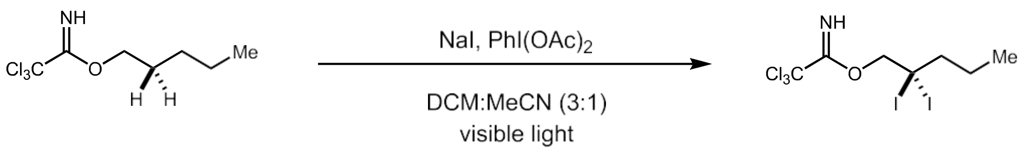

2,2-diiodopentyl 2,2,2-trichloroacetimidate (2)

Imidate **S2** (93 mg, 0.4 mmol) was subjected to **GP1**. Upon completion, the crude mixture was concentrated and purified (silica gel, 1% ethyl acetate/hexanes with 1% Et_3N) to yield di-iodoimidate **2** (0.16 g, 87%) as a yellow oil.

R_f: 0.40 (10% ethyl acetate/hexanes)

^{1}H NMR (400 MHz, $CDCl_3$): δ = 8.48 (bs, 1H), 4. 72 (s, 2H), 2.25 (t, *J* = 8.0 Hz, 2H), 1.65 (sext, *J* = 7.6 Hz, 2H), 1.04 (t, *J* = 7.4 Hz, 3H).

^{13}C NMR (150 MHz, $CDCl_3$): δ = 161.4, 91.0, 80.7, 53.1, 25.1, 12.9, 7.9.

HRMS (ESI-TOF) *m/z*: calc'd for $C_7H_{10}Cl_3I_2NONa$ $[M+Na]^+$ 505.7815, found 505.7809.

IR (film) cm^{-1}: 3338, 2960, 2931, 2873, 1770, 1663, 1284, 1071, 823.

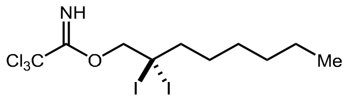

2,2-diiodooctyl 2,2,2-trichloroacetimidate (3)

Imidate **S3** (0.22 g, 0.8 mmol) was subjected to **GP1**. Upon completion, the crude mixture was concentrated. A crude yield of di-iodoimidate (88%) was determined via ^{1}H NMR (Isopropyl acetate as an internal standard). The crude mixture was purified (silica gel, hexanes N) to yield di-iodoimidate **3** (0.35 g, 83%) as a yellow oil.

Note: Silica gel was loaded with 1% Et_3N after which no more Et_3N was used.

R_f: 0.59 (10% ethyl acetate/hexanes)

^{1}H NMR (600 MHz, $CDCl_3$): δ = 8.48 (bs, 1H), 4.72 (s, 2H), 2.29 – 2.26 (m, 2H), 1.63 – 1.59 (m, 2H), 1.43 – 1.38 (m, 2H), 1.33 – 1.31 (m, 4H), 0.90 (t, *J* = 6.7 Hz, 3H).

^{13}C NMR (150 MHz, $CDCl_3$): δ = 161.4, 91.1, 80.7, 51.1, 31.7, 31.5, 28.1, 22.7, 14.1, 8.3.

HRMS (ESI-TOF) *m/z*: calc'd for $C_{10}H_{16}Cl_3I_2NONa$ $[M+Na]^+$ 547.8285, found 547.8259.

IR (film) cm^{-1}: 3342, 2955, 2928, 2856, 1772, 1666, 1448.

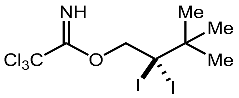

2,2-diiodo-3,3-dimethylbutyl 2,2,2-trichloroacetimidate (4)

Imidate **S1** (93 mg, 0.4 mmol) was subjected to **GP1**. Upon completion, the crude mixture was concentrated. A crude yield of di-iodoimidate (83%) was determined via ^{1}H NMR (Isopropylacetate as an internal standard). The crude mixture was purified (silica gel, hexanes with 1% Et_3N) to yield di-iodoimidate **4** (0.15 g, 73%) as a yellow solid.

R_f: 0.62 (10% ethyl acetate/hexanes)

^{1}H NMR (400 MHz, $CDCl_3$): δ = 8.47 (s, 1H), 4.77 (s, 2H), 1.39 (s, 9H).

^{13}C NMR (100 MHz, $CDCl_3$): δ = 161.8, 91.1, 79.6, 43.8, 35.1 (x3), 9.1.

HRMS (ESI-TOF) *m/z*: calc'd for $C_8H_{12}Cl_3I_2NONa$ $[M+Na]^+$ 519.7972, found 519.7963.

IR (film) cm^{-1}: 3346, 2970, 2932, 2884, 1467.

MP: 81-83 °C.

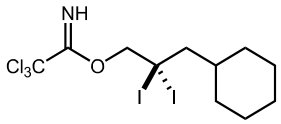

3-cyclohexyl-2,2-diiodopropyl 2,2,2-trichloroacetimidate (5)

Imidate **S5** (0.11 g, 0.4 mmol) was subjected to **GP1**. Upon completion, the crude mixture was concentrated and purified (silica gel, 1% ethyl acetate/hexanes with 1% Et_3N) to yield di-iodoimidate **5** (0.10 g, 63%) as a yellow oil.

R_f: 0.64 (10% ethyl acetate/hexanes).

^{1}H NMR (600 MHz, $CDCl_3$): δ = 8.47 (bs, 1H), 4.65 (s, 2H), 2.41 (d, *J* = 4.3 Hz, 2H), 1.91 – 1.90 (m, 2H), 1.69 – 1.62 (m, 3H), 1.38 – 1.30 (m, 3H), 1.20 – 1.13 (m, 3H).

^{13}C NMR (150 MHz, $CDCl_3$): δ = 161.4, 91.1, 80.5, 57.4, 40.7, 34.5 (x2), 26.3 (x2), 26.1, 6.8.

HRMS (ESI-TOF) *m/z*: calc'd for $C_{11}H_{16}Cl_3I_2NONa$ $[M+Na]^+$ 559.8285 found 559.8261.

IR (film) cm^{-1}: 3336, 2921, 2849, 1769, 1664, 1293, 1073, 824.

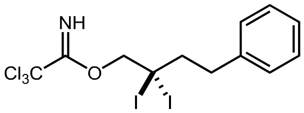

2,2-diiodo-4-phenylbutyl 2,2,2-trichloroacetimidate (6)

Imidate **S6** (0.12 g, 0.4 mmol) was subjected to **GP1**. Upon completion, the crude mixture was concentrated. A crude yield of di-iodoimidate (65%) was determined via ^{1}H NMR (Isopropyl acetate as an internal standard). The crude mixture was purified (silica gel, 1% ethyl acetate/hexanes with 1% Et_3N) to yield di-iodoimidate **6** (0.11 g, 51%) as a yellow oil.

R_f: 0.43 (10% ethyl acetate/hexanes)

^{1}H NMR (400 MHz, $CDCl_3$): δ = 8.52 (bs, 1H), 7.30 – 7.26 (m, 2H), 4.79 (s, 2H), 2.94 (t, *J* = 8.1 Hz, 2H), 2.59 (t, *J* = 8.2 Hz, 2H).

^{13}C NMR (150 MHz, $CDCl_3$): δ = 161.4, 139.5, 128.8, 126.6, 91.1, 80.5, 52.8, 38.0, 6.3.

HRMS (ESI-TOF) *m/z*: calc'd for $C_{12}H_{12}Cl_3I_2NONa$ $[M+Na]^+$ 567.7972, found 567.7944.

IR (film) cm^{-1}: 3335, 3025, 2925, 1766, 1664, 1285, 1073, 1001, 825.

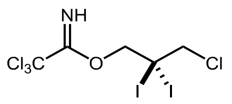

3-chloro-2,2-diiodopropyl 2,2,2-trichloroacetimidate (7)

Imidate **S7** (96 mg, 0.4 mmol) was subjected to **GP1**. Upon completion, the crude mixture was concentrated. A crude yield of di-iodoimidate (88%) was determined via ^{1}H NMR (Isopropyl acetate as an internal standard). The crude mixture was purified (silica gel, 1% ethyl acetate/hexanes with 1% Et_3N) to yield di-iodoimidate **7** (0.13 g, 66%) as a yellow oil.

R_f: 0.61 (10% ethyl acetate/hexanes)

^{1}H NMR (400 MHz, $CDCl_3$): δ = 8.53 (bs, 1H), 4.69 (s, 2H), 4.26 (s, 2H).

^{13}C NMR (150 MHz, $CDCl_3$): δ = 160.9, 90.9, 76.5, 56.6, – 0.52.

HRMS (ESI-TOF) *m/z*: calc'd for $C_5H_5Cl_4I_2NONa$ $[M+Na]^+$ 511.7112, found 511.7118.

IR (film) cm^{-1}: 3337, 1772, 1665, 1295, 1074, 824.

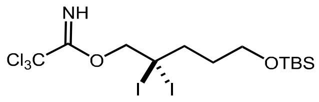

5-((tert-butyldimethylsilyl)oxy)-2,2-diiodopentyl 2,2,2-trichloroacetimidate (8)

Imidate **S8** (0.15 g, 0.4 mmol) was subjected to **GP1**. Upon completion, the crude mixture was concentrated and purified (silica gel, 3% ethyl acetate/hexanes with 1% Et_3N) to yield di-iodoimidate **8** (0.18 g, 74%) as a yellow oil.

R_f: 0.61 (10% ethyl acetate/hexanes)

^{1}H NMR (400 MHz, $CDCl_3$): δ = 8.48 (bs, 1H), 4.75 (s, 2H), 3.72 (t, *J* = 6.0 Hz, 2H), 2.35 (t, *J* = 7.8 Hz, 2H), 1.88 – 1.84 (m, 2H), 0.89 (s, 9H), 0.06 (s, 6H).

^{13}C NMR (150 MHz, $CDCl_3$): δ = 161.4, 91.1, 81.0, 61.5, 48.0, 35.2, 26.1, 18.4, 8.2, – 5.1.

HRMS (ESI-TOF) *m/z*: calc'd for $C_{13}H_{24}Cl_3I_2NO_2SiNa$ $[M+Na]^+$ 635.8629, found 635.8639.

IR (film) cm^{-1}: 3343, 2952, 2927, 2855, 1665, 1286, 1254, 1074, 832.

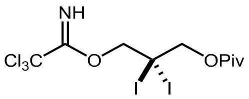

2,2-diiodo-3-(2,2,2-trichloro-1-iminoethoxy)propyl pivalate (9)

Imidate **S9** (0.12 g, 0.4 mmol) was subjected to **GP1**. Upon completion, the crude mixture was concentrated and purified (silica gel, 3% ethyl acetate/hexanes with 1% Et_3N) to yield di-iodoimidate **9** (0.11 g, 48%) as a yellow solid.

R_f: 0.38 (10% ethyl acetate/hexanes)

^{1}H NMR (400 MHz, $CDCl_3$): δ = 8.51 (bs, 1H), 4.71 (s, 2H), 4.54 (s, 2H), 1.26 (s, 3H).

^{13}C NMR (150 MHz, $CDCl_3$): δ = 176.5, 161.2, 90.9, 72.8, 39.2, 27.4, – 3.1.

HRMS (ESI-TOF) *m/z*: calc'd for $C_{10}H_{14}Cl_3I_2NO_3Na$ $[M+Na]^+$ 577.8026 found 577.7991.

IR (film) cm^{-1}: 3301, 2975, 2931, 1764, 1725, 1664, 1279, 1148, 1088.

MP: 108 – 110 °C.

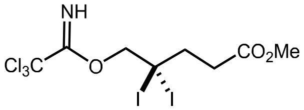

methyl 4,4-diiodo-5-(2,2,2-trichloro-1-iminoethoxy)pentanoate (10)

Imidate **S10** (0.11 g, 0.4 mmol) was subjected to GP1. Upon completion, the crude mixture was concentrated and purified (1% ethyl acetate/hexanes with 1% Et_3N) to yield di-iodoimidate **10** (0.10 g, 49%) as a yellow oil.

R_f: 0.38 (10% ethyl acetate/hexanes)

^{1}H NMR (400 MHz, $CDCl_3$): δ = 8.52 (bs, 1H), 4.76 (s, 2H), 3.72 (s, 3H), 2.77 – 2.74 (m, 2H), 2.61 – 2.58 (m, 2H).

^{13}C NMR (150 MHz, $CDCl_3$): δ = 171.9, 161.3, 90.9, 80.9, 52.1, 45.7, 36.9, 4.6.

HRMS (ESI-TOF) *m/z*: calc'd for $C_8H_{10}Cl_3I_2NO_3Na$ $[M+Na]^+$ 549.7713 found 549.7703.

IR (film) cm^{-1}: 2950, 1713, 1655, 1617, 1436, 1271, 1204, 1172, 1108, 827.

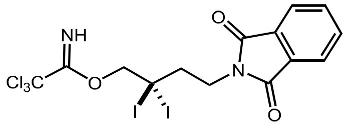

4-(1,3-dioxoisoindolin-2-yl)-2,2-diiodobutyl 2,2,2-trichloroacetimidate (11)

Imidate **S11** (68 mg, 0.19 mmol) were subjected to **GP1**. Upon completion, the crude mixture was concentrated and purified (silica gel, 50% ethyl acetate/hexanes with 1% Et_3N) to yield di-iodoimidate **11** (81 mg, 70%) as a white solid.

R_f: 0.13 (10% ethyl acetate/hexanes)

^{1}H NMR (600 MHz, $CDCl_3$): δ = 8.51 (bs, 1H), 7.85 – 7.83 (m, 2H), 7.72 – 7.71 (m, 2H), 4.78 (s, 2H), 4.02 (t, *J* = 7.5 Hz, 2H), 2.70 (t, *J* = 7.5 Hz, 2H)

^{13}C NMR (150 MHz, $CDCl_3$): δ = 169.0, 161.2, 134.2, 132.2, 123.5, 90.9, 80.6, 48.1, 40.5, – 1.5.

HRMS (ESI-TOF) *m/z*: calc'd for $C_{14}H_{11}Cl_3I_2N_2O_3Na$ $[M+Na]^+$ 636.7822 found 636.7794.

IR (film) cm^{-1}: 3057, 1771, 1706, 1439, 1396, 1368, 824.

MP: 152 – 153 °C.

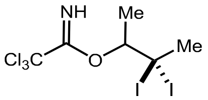

3,3-diiodobutan-2-yl 2,2,2-trichloroacetimidate (12)

Imidate **S12** (87 mg, 0.4 mmol) was subjected to **GP1**. Upon completion, the crude mixture was concentrated. A crude yield of di-iodoimidate (87%) was determined via ^{1}H NMR (Isopropyl acetate as an internal standard). The crude mixture was purified (silica gel, 1% ethyl acetate/hexanes with 1% Et_3N) to yield di-iodoimidate **12** (0.13 g, 70%) as a yellow oil.

R_f: 0.63 (10% ethyl acetate/hexanes)

^{1}H NMR (400 MHz, $CDCl_3$): δ = 8.50 (bs, 1H), 4.63 (q, *J* = 6.0 Hz, 1H), 3.02 (s, 3H), 1.59 (d, *J* = 5.9 Hz, 3H).

^{13}C NMR (150 MHz, $CDCl_3$): δ = 161.0, 91.2, 84.2, 43.6, 19.2, 5.4.

HRMS (ESI-TOF) *m/z*: calc'd for $C_6H_8Cl_3I_2NONa$ $[M+Na]^+$ 491.7659, found 491.7685.

IR (film) cm^{-1}: 3339, 2927, 1664, 1342, 1280, 1072, 1055, 815.

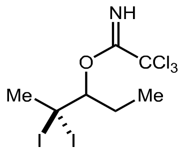

2,2-diiodopentan-3-yl 2,2,2-trichloroacetimidate (13)

Imidate **S13** (93 mg, 0.4 mmol) was subjected to **GP1** with the following changes: iodobenzene diacetate (0.26 g, 0.8 mmol) and NaI (0.12 g, 0.8 mmol). Upon completion, the crude mixture was concentrated and purified (silica gel, 3% ethyl acetate/hexanes with 1% Et_3N) to yield di-iodoimidate **13** (0.13 g, 68%) as a yellow oil.

Note: The isolated sample contains 10% of the 2,4 distal di-iodide product as a mixture of diastereomers.

R_f: 0.62 (10% ethyl acetate/hexanes)

^{1}H NMR (400 MHz, $CDCl_3$): δ = 8.53 (s, 1H), 4.73 (dd, *J* = 9.7 Hz, 2.2 Hz, 1H), 2.02 – 1.91 (m, 2H), 1.10 (t, *J* = 7.4 Hz, 3H).

^{13}C NMR (150 MHz, $CDCl_3$): δ = 162.3, 91.4, 87.6, 44.0, 29.5, 10.3, 5.7.

HRMS (ESI-TOF) *m/z*: calc'd for $C_7H_{10}Cl_3I_2NONa$ $[M+Na]^+$ 505.7815, found 505.7819.

IR (film) cm^{-1}: 3343, 2970, 2935, 1763, 1710, 1663, 1340, 1278, 1054, 977.

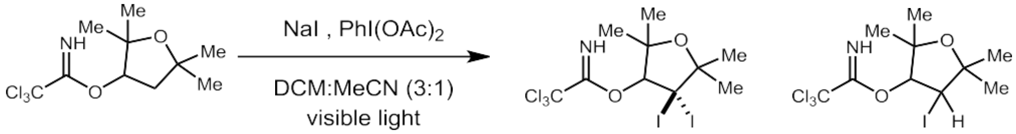

Imidate **S14** (0.12 g, 0.4 mmol) was subjected to **GP1**. Upon completion, the crude mixture was concentrated and purified (silica gel, 1% ethyl acetate/hexanes with 1% Et_3N) to yield di-iodoimidate **14** (0.10 g, 48%) as a yellow oil and mono-iodoimidate **14'** (0.04 g, 24%) as a colorless oil.

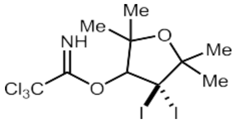

4,4-diiodo-2,2,5,5-tetramethyltetrahydrofuran-3-yl 2,2,2-trichloroacetimidate (14)

R_f: 0.60 (10% ethyl acetate/hexanes)

^{1}H NMR (400 MHz, $CDCl_3$): δ = 8.66 (bs, 1H), 5.78 (s, 1H), 1.75 (s, 3H), 1.72 (s, 3H), 1.61 (s, 3H), 1.39 (s, 3H).

^{13}C NMR (150 MHz, CDCl$_3$): δ = 162.1, 92.4, 85.9, 81.6, 32.4, 30.0, 29.4, 26.4, 14.6.

HRMS (ESI-TOF) *m/z*: calc'd for $C_{10}H_{14}Cl_3I_2NO_2Na$ [M+Na]$^+$ 561.8077, found 561.8098.

IR (film) cm^{-1}: 2978, 1766, 1726, 1616, 1371, 1215, 986, 751.

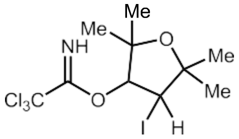

4-iodo-2,2,5,5-tetramethyltetrahydrofuran-3-yl 2,2,2-trichloroacetimidate (14')

R$_f$: 0.52 (10% ethyl acetate/hexanes)

^{1}H NMR (400 MHz, CDCl$_3$): δ = 8.47 (bs, 1H), 5.38 (d, J = 5.2 Hz, 1H), 4.41 (d, J = 5.2 Hz, 1H), 1.53 (s, 3H), 1.39 (s, 3H), 1.37 (s, 3H), 1.34 (s, 3H).

^{13}C NMR (150 MHz, CDCl$_3$): δ = 162.3, 91.6, 85.0, 83.2, 82.8, 32.4, 30.1, 28.4, 24.8.

HRMS (ESI-TOF) *m/z*: calc'd for $C_{10}H_{15}Cl_3INO_2Na$ [M+Na]$^+$ 435.9111, found 435.9092.

IR (film) cm^{-1}: 3345, 2973, 2930, 1662, 1463, 1380, 1303, 1109, 1079.

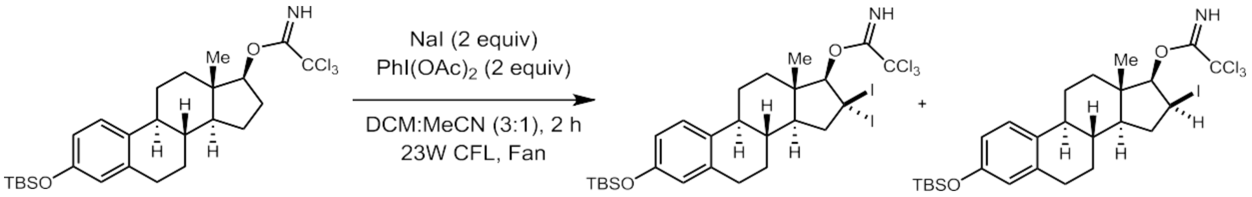

Imidate **S15** (53 mg, 0.1 mmol) were subjected to **GP1** with the following changes: concentration was reduced to 0.1 M. Upon completion, the crude mixture was concentrated. A crude yield of di-iodoimidate **15** (48%) and mono-iodoimidate **15'** (46%) was determined via ^{1}H NMR (Isopropyl acetate as an internal standard). The crude mixture was concentrated and purified (silica gel, 3% ethyl acetate/hexanes with 1% Et_3N).

Note: Di-iodoimidate is prone to decomposition even during storage at 0 °C for an extended time.

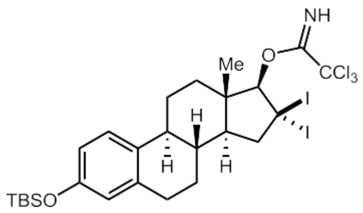

3-((*tert*-butyldimethylsilyl)oxy)-16,16-diiodo-17-trichloroacetimidatyl estradiol (15)

R_f: 0.58 (10% ethyl acetate/hexanes)

^{1}H NMR (400 MHz, $CDCl_3$): δ = 8.67 (bs, 1H), 7.09 (d, *J* = 8.4 Hz, 1H), 6.62 (dd, *J* = 8.5, 2.6 Hz, 1H), 6.56 (d, *J* = 2.6 Hz, 1H), 5. 49 (s, 1H), 3.48 (dd, *J* = 14.6, 5.8 Hz, 1H), 3.37 (t, *J* = 14.0 Hz, 1H), 2.83 – 2.80 (m, 2H), 2.33 – 2.24 (m, 3H), 1.95 – 1.93 (m, 1H), 1.82 – 1.77 (m, 2H), 1.53 (s, 3H), 1.48 – 1.43 (m, 2H), 1.07 (s, 3H), 0.98 (s, 9H), 0.19 (s, 6H).

^{13}C NMR (150 MHz, $CDCl_3$): δ = 164.1, 153.9, 137.7, 132.5, 126.1, 120.2, 117.6, 107.2, 100.0, 87.7, 48.8, 46.8, 43.8, 41.0, 38.1, 37.9, 29.6, 28.1, 25.9, 23.6, 18.4, 11.5, – 4.2.

HRMS (ESI-TOF) *m/z*: calc'd for $C_{26}H_{37}Cl_3I_2NO_2Si$ $[M+H]^+$ 781.9749, found 781.9722.

IR (film) cm^{-1}: 2928, 2857, 1757, 1708, 1655, 1606, 1495, 1471, 1288, 1251, 955, 837.

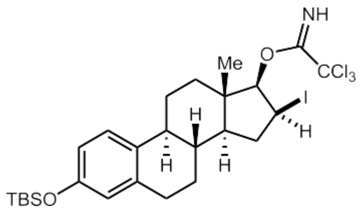

3-((*tert*-butyldimethylsilyl)oxy)-16-iodo-17-trichloroacetimidatyl estradiol (15')

R_f: 0.50 (10% ethyl acetate/hexanes)

^{1}H NMR (400 MHz, $CDCl_3$): δ = 8.36 (bs, 1H), 7.10 (d, *J* = 8.4 Hz, 1H), 6.60 (d, *J* = 8.4 Hz, 1H), 6.55 (d, *J* = 2.6 Hz, 1H), 4.81 (q, *J* = 8.1 Hz, 1H), 4.24 (d, *J* = 8.4 Hz, 1H), 2.81 – 2.80 (m, 2H), 2.71 – 2.64 (m, 1H), 2.26 – 2.15 (m, 3H), 2.01 – 1.99 (m, 1H), 1.89 – 1.83 (m, 1H), 1.59 – 1.26 (m, 6H), 1.18 (s, 3H), 0.97 (s, 9H), 0.18 (s, 6H).

^{13}C NMR (150 MHz, $CDCl_3$): δ = 162.6, 153.7, 137.7, 132.5, 126.2, 120.2, 117.5, 91.8, 85.3, 50.6, 44.2, 44.1, 39.5, 38.2, 37.8, 29.6, 27.4, 25.9, 25.7, 22.5, 18.3, 13.7, – 4.2.

HRMS (ESI-TOF) *m/z*: calc'd for $C_{26}H_{37}Cl_3INO_2SiNa$ $[M+Na]^+$ 678.0602, found 678.0614.

IR (film) cm^{-1}: 3336, 2927, 2854, 2360, 2160, 1660, 1607, 1495, 1471, 1462, 1305, 1254, 1131, 1115, 1084, 955, 837, 797.

MP: 140 °C (decomposed).

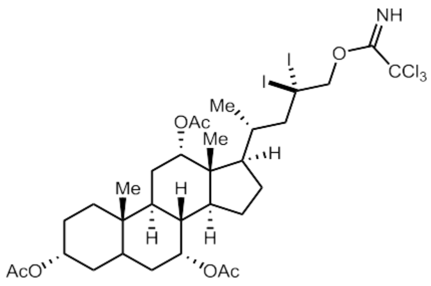

3,7,12-triacetoxy-4,4-diiodo-5-cholanyl-2,2,2-trichloroacetimidate (16)

Imidate **S16** (66.5 mg, 0.1 mmol) was subjected to **GP1** with the following changes: concentration was reduced to 0.1 M. Upon completion, the crude reaction was concentrated and purified (silica gel, 25% ethyl acetate/hexanes with 1% Et_3N) to yield di-iodoimidate **16** (64.9 mg, 71%) as a yellow solid.

R_f: 0.20 (25% ethyl acetate/hexanes)

^{1}H NMR (400 MHz, $CDCl_3$): δ = 8.48 (bs, 1H), 5.07 (s, 1H), 4.89 (s, 2H), 4.69 (d, *J* = 12.1 Hz, 1H), 4.55 – 4.45 (m, 3H), 2.68 (d, *J* = 15.4 Hz, 1H), 2.19 – 2.18 (m, 2H), 2.14 – 2.10 (m, 8H), 2.07 – 2.06 (m, 4H), 2.03 (s, 3H), 1.78 – 1.73 (m, 6H), 1.66 – 1.57 (m, 9H), 1.51 – 1.47 (m, 5H), 1.07 – 1.06 (d, *J* = 6.4 Hz, 3H), 0.90 (s, 3H), 0.79 (s, 3H).

^{13}C NMR (150 MHz, $CDCl_3$): δ = 170.6, 170.4, 170.4, 161.1, 91.0, 80.3, 75.5, 74.2, 70.8, 56.1, 48.3, 45.4, 43.7, 41.1, 38.5, 38.0, 34.9, 34.8, 34.5, 34.5, 31.4, 28.9, 27.2, 27.0, 25.6, 22.8, 22.7, 21.7, 21.6, 21.5, 12.4, 6.7.

HRMS (ESI-TOF) *m/z*: calc'd for $C_{32}H_{46}Cl_3I_2NO_7Na$ $[M+Na]^+$ 938.0327, found 938.0384.

IR (film) cm^{-1}: 2940, 2870, 1720, 1377, 1364, 1233, 728.

MP: 89 – 90 °C.

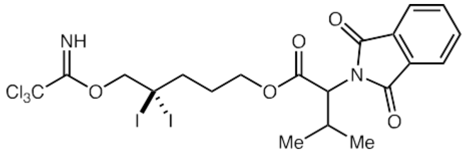

4,4-diiodo-5-(2,2,2-trichloro-1-iminoethoxy)pentyl 2-(1,3-dioxoisoindolin-2-yl)-3-methyl butanoate (17)

Imidate **S17** (95.2 mg, 0.2 mmol) was subjected to **GP1**. Upon completion, the crude reaction was concentrated and purified (silica gel, 5% ethyl acetate/hexanes with 1% Et_3N) to yield di-iodoimidate **17** (104 mg, 72%) as a yellow solid.

R_f: 0.31 (20% ethyl acetate/hexanes)

^{1}H NMR (400 MHz, $CDCl_3$): δ = 8.48 (bs, 1H), 7.88 (dd, *J* = 5.4, 3.0 Hz, 2H), 7.74 (dd, *J* = 5.4, 3.0 Hz, 2H), 4.56 (d, *J* = 11.9 Hz, 1H), 4.60 (d, *J* = 11.9 Hz, 1H), 4.58 (d, *J* = 8.2

Hz, 1H), 4.27 – 4.23 (m, 1H), 4.21 – 4.18 (m, 1H), 2.78 – 2.73 (m, 1H), 2.28 – 2.23 (m, 1H), 2.21 – 2.17 (m, 1H), 1.97 –1.92 (m, 2H), 1.16 (d, *J* = 6.6 Hz, 3H), 0.92 (d, *J* = 6.8 Hz, 3H).

^{13}C NMR (150 MHz, $CDCl_3$): δ = 168.9, 167.9, 161.3, 134.4, 131.9, 123.8, 90.9, 80.8, 63.8, 57.7, 47.5, 31.1, 28.8, 21.2, 19.6, 5.7.

HRMS (ESI-TOF) *m/z*: calc'd for $C_{20}H_{21}Cl_3I_2N_2O_5Na$ $[M+Na]^+$ 750.8503, found 750.8513.

IR (film) cm^{-1}: 3337, 2966, 2924, 2896, 2872, 1738, 1711, 1665, 1466, 1441.

MP: 106 – 108 °C.

VI. Di-bromination

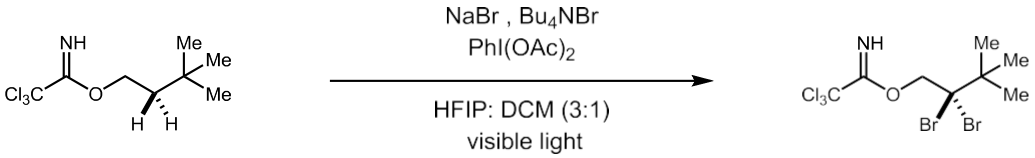

2,2-dibromo-3,3-dimethylbutyl 2,2,2-trichloroacetimidate (18)

Imidate **S1** (49 mg, 0.2 mmol) was subjected to **GP2**. The mixture was concentrated and purified (silica gel, 1% ethyl acetate in hexanes with 1% Et_3N) to yield di-bromoimidate **18** (74 mg, 92%) as a colorless solid. **18** was recrystallized from a mixture of chloroform and hexanes (10:1) to obtain a crystal of X-ray quality.

R_f: 0.50 (10% ethyl acetate/hexanes)

^{1}H NMR (400 MHz, $CDCl_3$): δ = 8.47 (bs, 1H), 4.87 (s, 2H), 1.37 (s, 9H).

^{13}C NMR (150 MHz, $CDCl_3$): δ = 162.2, 91.1, 83.5, 75.6, 44.1, 28.0.

HRMS (ESI-TOF) *m/z*: calc'd for $C_8H_{12}Br_2Cl_3NONa$ $[M+Na]^+$ 423.8249, found 423.8233.

IR (film) cm^{-1}: 3341, 2977, 1665, 1367, 1299, 1070, 829.

MP: 61 – 63 °C.

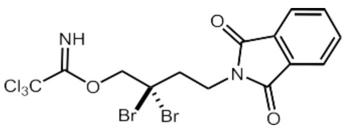

2,2-dibromo-4-(1,3-dioxoisoindolin-2-yl)butyl 2,2,2-trichloroacetimidate (20)

Imidate **S11** (70 mg, 0.2 mmol) was subjected to **GP2**. The mixture was concentrated and purified (silica gel, 20% ethyl acetate in hexanes with 1% Et_3N) to yield di-bromoimidate **20** (71 mg, 68%) as a white solid.

R_f: 0.33 (25% ethyl acetate/hexanes)

^{1}H NMR (400 MHz, $CDCl_3$): δ = 8.52 (s, 1H), 7.86 – 7.85 (m, 2H), 7.73 – 7.72 (m, 2H), 4.86 (s, 2H), 4.12 (t, *J* = 7.4 Hz, 2H), 2.84 (t, *J* = 7.4 Hz, 2H).

^{13}C NMR (150 MHz, $CDCl_3$): δ = 168.0, 161.6, 134.2, 132.2, 123.5, 90.8, 76.6, 62.7, 44.3, 36.5.

HRMS (ESI-TOF) *m/z*: calc'd for $C_{14}H_{11}Br_2Cl_3N_2O_3Na$ $[M+Na]^+$ 540.8100, found 540.8080.

IR (film) cm^{-1}: 3339, 2939, 1773, 1709, 1668, 1397, 1372, 1290.

MP: 84 – 86 °C.

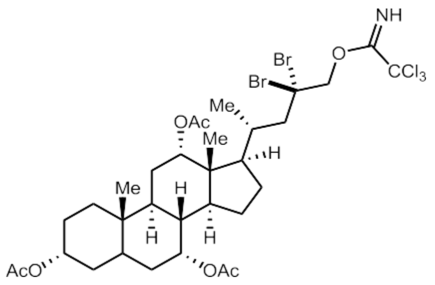

3,7,12-triacetoxy-4,4-dibromo-5-cholanyl-2,2,2-trichloroacetimidate (22)

Imidate **S16** (66.5 mg, 0.1 mmol) was subjected to **GP2.** The mixture was concentrated and purified (silica gel, 25% ethyl acetate in hexanes with 1% Et_3N) to yield di-bromoimidate **22** (74.6 mg, 91%) as a white solid.

R_f: 0.19 (25% ethyl acetate/hexanes)

^{1}H NMR (400 MHz, $CDCl_3$): δ = 8.49 (s, 1H), 5.08 (s, 1H), 4.88 (s, 2H), 4.77 (d, *J* = 12.0 Hz), 4.68 (d, *J* = 11.9 Hz, 1 H), 4.55 (bs, 2H), 2.67 (d, *J* = 15.4 Hz, 1 H), 2.12 (s, 3H), 2.06 (s, 5H), 2.01 (s, 6H), 1.76 – 1.74 (m, 4H), 1.64 – 1.57 (m, 7H), 1.48 – 1.46 (m, 4H), 1.06 – 1.05 (m, 3H), 0.90 (s, 3H), 0.77 (s, 3H).

^{13}C NMR (150 MHz, $CDCl_3$): δ = 170.6, 170.5, 170.4, 161.6, 90.9, 76.6, 75.5, 74.2, 70.8, 67.3, 51.9, 48.4, 45.4, 43.6, 41.0, 37.9, 35.3, 34.8, 34.7, 34.4, 31.4, 29.8, 28.9, 27.3, 21.0, 25.6, 22.8, 22.7, 21.6, 21.5, 20.4, 12.3.

HRMS (ESI-TOF) *m/z*: calc'd for $C_{32}H_{46}Cl_3Br_2NO_7Na$ $[M+Na]^+$ 842.0604, found 842.0631.

IR (film) cm^{-1}: 2941, 2254, 1721, 1668, 1377, 1248, 1023, 906, 726.

MP: 77 – 79 °C.

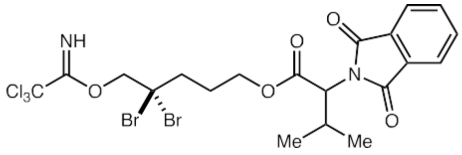

4,4-dibromo-5-(2,2,2-trichloro-1-iminoethoxy)pentyl 2-(1,3-dioxoisoindolin-2-yl)-3-methylbutanoate

Imidate **S17** was subjected to **GP2**. After the reaction, the crude reaction was purified (silica gel, 5% ethyl acetate/hexanes with 1% Et_3N) to yield dibromoimidate (92 mg, 73%) as a clear oil.

R_f: 0.30 (20% ethyl acetate/hexanes)

^{1}H NMR (600 MHz, $CDCl_3$): δ = 8.49 (bs, 1H), 7.88 (dd, *J* = 5.4, 3.1 Hz, 2H), 7.75 (dd, *J* = 5.4, 3.0 Hz, 2H), 4.71 (d, *J* = 11.9 Hz, 1H), 4.68 (d, *J* = 12.0 Hz, 1H), 4.57 (d, *J* = 8.2

Hz, 1H), 4.26 – 4.17 (m, 2H), 2.41 – 2.31 (m, 2H), 2.03 – 1.99 (m, 2H), 1.16 (d, *J* = 6.8 Hz, 3H), 0.92 (d, *J* = 6.8 Hz, 3H).

^{13}C NMR (150 MHz, $CDCl_3$): δ = 168.9, 167.9, 161.7, 134.4, 131.8, 123.8, 90.8, 76.8, 66.4, 64.2, 57.8, 43.4, 28.7, 26.8, 21.1, 19.6.

HRMS (ESI-TOF) *m/z*: calc'd for $C_{20}H_{21}Br_2Cl_3N_2O_5Na$ $[M+Na]^+$ 654.8780, found 654.8758.

IR (film) cm^{-1}: 3336, 2964, 2928, 2874, 2851, 1744, 1713, 1668, 1467.

VII. Mono-chlorination

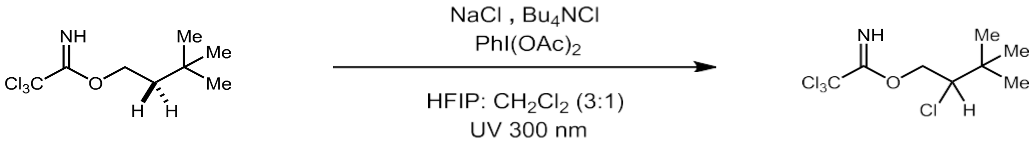

2-chloro-3,3-dimethylbutyl 2,2,2-trichloroacetimidate (19)

Imidate **S1** (49 mg, 0.2 mmol) was subjected to **GP3**. The mixture was concentrated and purified (silica gel, 1% ethyl acetate in hexanes with 1% Et_3N) to yield mono-chloroimidate **19** (30 mg, 53%) as a yellow solid.

R_f: 0.51 (10% ethyl acetate/hexanes)

^{1}H NMR (400 MHz, $CDCl_3$): δ = 8.35 (bs, 1H), 4.67 (dd, *J* = 11.7, 3.4 Hz, 1H), 4.40 (dd, *J* = 11.7, 8.3 Hz, 1H), 4.06 (dd, *J* = 8.3, 3.4 Hz, 1H), 1.11 (S, 9H).

^{13}C NMR (150 MHz, $CDCl_3$): δ = 162.8, 91.3, 70.8, 69.1, 35.4, 27.1.

HRMS (ESI-TOF) *m/z*: calc'd for $C_8H_{13}Cl_4NONa$ [M+Na]$^+$ 301.9649 found 301.9669.

IR (film) cm^{-1}: 3345, 2965, 1769, 1666, 1478, 1315, 1077, 829, 797

MP: 156 – 157 °C.

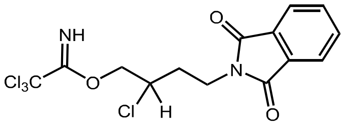

2-chloro-4-(1,3-dioxoisoindolin-2-yl)butyl 2,2,2-trichloroacetimidate (21)

Imidate **S11** (70 mg, 0.2 mmol) was subjected to **GP3**. The mixture was concentrated and purified (silica gel, 10% to 30% ethyl acetate in hexanes with 1% Et_3N) to yield mono-chloroimidate **21** (70 mg, 88%) as a colorless oil.

R_f: 0.38 (25% ethyl acetate/hexanes)

^{1}H NMR (600 MHz, $CDCl_3$): δ = 8.39 (bs, 1H), 7.86 – 7.85 (m, 2H), 7.73 – 7.72 (m, 2H), 4.53 (dd, *J* = 11.4, 5.9 Hz, 1H), 4.43 (dd, *J* = 11.4, 6.0 Hz, 1H), 4.27 – 4.26 (m, 1H), 3.99 – 3.89 (m, 2H), 2.38 – 2.33 (m, 1H), 2.18 – 2.11 (m, 1H).

^{13}C NMR (150 MHz, $CDCl_3$): δ = 168.3, 162.4, 134.2, 123.5, 91.1, 71.5, 55.5, 35.3, 33.6.

HRMS (ESI-TOF) *m/z*: calc'd for $C_{14}H_{13}Cl_4N_2O_3$ [M+H]$^+$ 396.9680, found 396.9667.

IR (film) cm^{-1}: 2939, 2185, 1712, 1670, 1604, 1459, 1397, 1240.

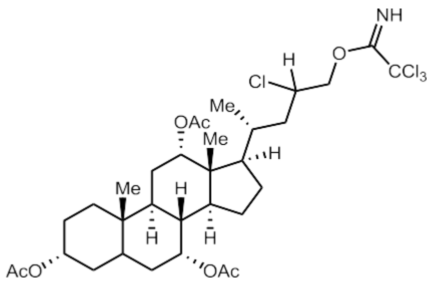

3,7,12-triacetoxy-4-chloro-5-cholanyl-2,2,2-trichloroacetimidate (23)

Imidate **S16** (66 mg, 0.1 mmol) was subjected to **GP3.** The mixture was concentrated and purified (silica gel, 10% to 25% ethyl acetate in hexanes with 1% Et_3N) to yield mono-chloroimidate **22** (64 mg, 90%, as a mixture of diastereomers dr = 3:1) as a white solid.

R_f: 0.24 (25% ethyl acetate/hexanes)

^{1}H NMR (600 MHz, $CDCl_3$): δ = 8.37 (bs, 1H, major diastereomer), 8.20 (bs, 1H, minor diastereomer), 5.12 (bs, 1H, minor diastereomer), 5.09 (bs, 1H, major diastereomer), 4.90 (s, 2H), 4.57 (m, 2H), 4.44 – 4.43 (m, 2H), 4.26 – 4.24 (m, 1H), 2.14 – 2.12 (m, 5H), 2.08 – 2.07 (m, 4H), 2.04 (s, 5H), 1.85 – 1.84 (m, 4H), 1.75 – 1.40 (m, 24H), 1.24 (m, 3H), 1.08 – 1.06 (m, 3H), 0.91 (m, 3H), 0.85 – 0.83 (m, 4H), 0.77 (s, 1H), 0.72 (s, 3H).

^{13}C NMR (150 MHz, $CDCl_3$): δ = 170.8 (major diastereomer), 170.6 (minor diastereomer), 163.3, 162.6, 91.2, 56.5, 56.3, 48.4, 47.8, 45.4, 45.3, 43.6, 43.5, 41.1, 38.0, 34.9, 34.5, 33.9, 32.4, 31.9, 31.4, 29.0, 27.4, 27.0, 25.7, 25.0, 23.0, 22.7, 21.7, 21.6, 21.5, 18.5, 18.0, 17.6, 12.4.

HRMS (ESI-TOF) *m/z*: calc'd for $C_{32}H_{48}Cl_4NO_7$ $[M+H]^+$ 698.2185 found 698.2157.

IR (film) cm^{-1}: 2941, 2871, 2254, 1727, 1667, 1377, 1233, 1023.

MP: 76 – 77 °C.

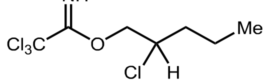

2-chloropentyl 2,2,2-trichloroacetimidate (26)

Imidate **S2** (47 mg, 0.2 mmol) was subjected to **GP3**. The mixture was concentrated. A crude yield of mono-chloroimidate (72%) was determined via ^{1}H NMR (Isopropyl acetate as an internal standard). The crude mixture was purified (silica gel, 1% ethyl acetate in hexanes with 1% Et_3N) to yield mono-chloroimidate **26** (27 mg, 50%) as a colorless oil.

R_f: 0.45 (10% ethyl acetate/hexanes)

^{1}**H NMR (600 MHz, CDCl$_3$):** δ = 8.37 (bs, 1H), 4.49 – 4.39 (m, 2H), 4.25 – 4.21 (m, 1H), 1.88 – 1.83 (m, 1H), 1.77 – 1.74 (m, 1H), 1.63 – 1.60 (m, 1H), 1.50 – 1.47 (m, 1H), 0.95 (t, J = 7.4 Hz, 3H).

^{13}**C NMR (100 MHz, CDCl$_3$):** δ = 162.6, 91.2, 72.1, 58.1, 36.7, 19.3, 13.6.

HRMS (ESI-TOF) *m/z*: calc'd for $C_7H_{12}Cl_4NO$ $[M+H]^+$ 265.9673 found 265.9667.

IR (film) cm^{-1}: 3345, 2965, 2875, 1667, 1465, 1382, 1303, 1085, 1003, 828, 797.

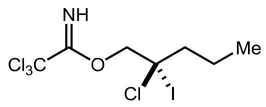

2-chloro-2-iodopentyl 2,2,2-trichloroacetimidate (± 27)

Chloroimidate **26** (50 mg, 0.2 mmol) was subjected to **GP1** with the following changes: to the reaction was added iodobenzene diacetate (64 mg, 0.2 mmol) and NaI (30 mg, 0.2 mmol). Upon completion, the crude mixture was concentrated and purified (silica gel, 100% hexanes with 1% Et_3N) to yield iodoimidate ±**27** (54 mg, 71%) as a colorless oil.

R$_f$: 0.13 (100% hexanes)

^{1}**H NMR (600 MHz, CDCl$_3$):** δ = 8.48 (bs, 1H), 4.74 – 4.68 (m, 2H), 2.34 – 2.29 (m, 1H), 2.15 – 2.10 (m, 1H), 1.71 – 1.64 (m, 2H), 1.02 (t, J = 7.4 Hz, 3H).

^{13}**C NMR (150 MHz, CDCl$_3$):** δ = 161.7, 91.0, 78.5, 54.3, 50.2, 21.9, 13.4.

HRMS (ESI-TOF) *m/z*: calc'd for $C_7H_{10}Cl_4INONa$ $[M+Na]^+$ 413.8459 found 413.8432.

IR (film) cm^{-1}: 3342, 2963, 2874, 1773, 1667, 1299, 1077, 828, 795.

VIII. Post-synthetic functionalization

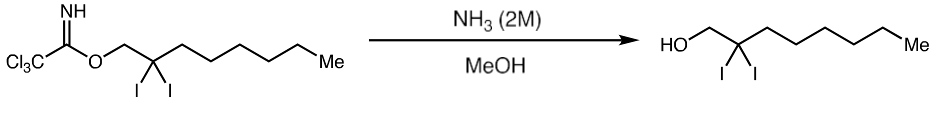

2,2-diiodooctan-1-ol (28)

Di-iodoimidate **3** (51.6 mg, 0.1 mmol) was dissolved in 2M NH_3 (3 mL in MeOH, 6 mmol) and stirred at room temperature for 23 hours. Upon completion (monitored by TLC), the reaction was concentrated and purified (silica gel, 5% ethyl acetate/hexanes) to yield alcohol **28** (25 mg, 66%) as a yellow oil.

R$_f$: 0.55 (20% ethyl acetate/hexanes)

^{1}H NMR (400 MHz, CDCl$_3$): δ = 3.86 (d, *J* = 6.6 Hz, 2H), 2.60 (t, *J* = 7.1 Hz, 1H), 2.21 – 2.17 (m, 2H), 1.65 – 1.58 (m, 2H), 1.43 – 1.36 (m, 2H), 1.34 – 1.30 (m, 4H), 0.90 (t, *J* = 6.9 Hz, 3H).

^{13}C NMR (100 MHz, CDCl$_3$): δ = 77.9, 50.2, 31.74, 31.71, 28.2, 27.6, 22.7, 14.2.

HRMS (ESI-TOF) *m/z*: calc'd for $C_8H_{16}I_2ONa$ $[M+Na]^+$ 404.9188 found 404.9158.

IR (film) cm^{-1}: 3387, 2954, 2925, 2853, 1773, 1456.

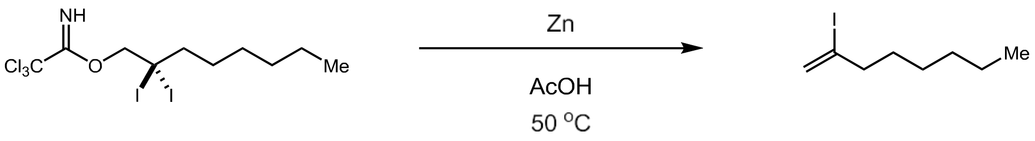

2-iodooct-1-ene (29)

A mixture of di-iodoimidate **3** (0.10 g, 0.19 mmol) and zinc (62 mg, 0.95 mmol) in acetic acid (1 mL, 0.2 M) was stirred at 50 °C for 17 h. Upon completion, the solution was concentrated and purified (silica gel, 100% hexanes) to yield vinyliodide **29** (32 mg, 71%).

^{1}H NMR (600 MHz, CDCl$_3$): δ = 6.01 – 6.00 (m, 1H), 5.68 (m, 1H), 2.40 – 2.36 (m, 2H), 1.53 – 1.48 (m, 2H), 1.29 (m, 6H), 0.89 (t, *J* = 6.8 Hz, 3H).

^{13}C NMR (150 MHz, CDCl$_3$): δ = 125.2, 112.9, 45.5, 31.7, 29.2, 28.0, 22.7, 14.2.

Spectroscopic data is consistent with reported literature data[8]

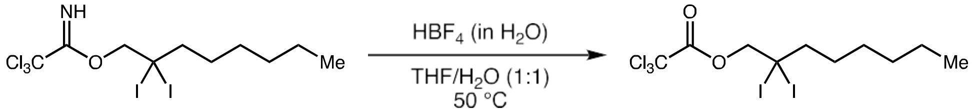

2,2-diiodooctyl 2,2,2-trichloroacetate (30)

Di-iodoimidate **3** (52.6 mg, 0.1 mmol) was dissolved in a 1:1 mixture of THF and H_2O (2 mL, 0.5 M). To this stirring solution was added HBF_4 (48% in water) (0.56 mg, 40 μL, 0.3 mmol) and the reaction was heated to 50 °C for 45 minutes. Upon completion, the reaction was diluted with ethyl acetate (2 mL) and quenched with sat'd $NaHCO_3$ (1 mL). The organic layer was separated and the aqueous phase was extracted with ethyl acetate (2 x 2 mL). The combined organic solution was dried over $MgSO_4$, concentrated, and purified by flash column chromatography (silica gel, hexanes) to yield the target ester **30** (42 mg, 81%) as a yellow oil.

R$_f$: 0.18 (hexanes)

[8] (a) Kamiya, N.; Chikami, Y.; Ishii, Y. *Synlett.* **1990**, 675. (b) Cheung, L.L.W.; Yudin, A.K. *Org. Lett.* **2009**, *11*, 1281.

^{1}H NMR (600 MHz, CDCl$_3$): δ = 4.80 (s, 2H), 2.22 – 2.20 (m, 2H), 1.64 – 1.59 (m, 2H), 1.43 – 1.40 (m, 2H), 1.34 – 1.31 (m, 4H), 0.90 (t, *J* = 6.8 Hz, 3H).

^{13}C NMR (150 MHz, CDCl$_3$): δ = 160.5, 89.6, 79.9, 50.9, 31.7, 31.6, 28.1, 22.7, 14.1, 5.7.

HRMS (ESI-TOF) *m/z*: calc'd for $C_{10}H_{15}Cl_3I_2O_2Na$ [M+Na]$^+$ 548.8125 found 548.8097.

IR (film) cm^{-1}: 2980, 2856, 2929, 2857, 1770, 1446.

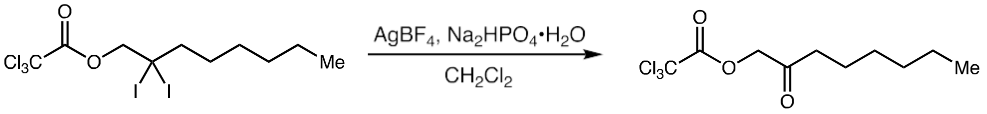

2-oxooctyl 2,2,2-trichloroacetate (31)

A vial of ester **30** (52.5 mg, 0.1 mmol) and $Na_2HPO_4 \cdot H_2O$ (36 mg, 0.3 mmol) was brought into an N_2 filled glove box to which $AgBF_4$ (58.4 mg, 0.3 mmol) was added. The vial was removed from the glove box, CH_2Cl_2 (3 mL, 0.33 M) was added, and the reaction was stirred for 2 hours. The reaction was concentrated and purified (silica gel, hexanes to 2% ethyl acetate/hexanes) to yield the target ketone **31** (19 mg, 66%) as a yellow oil.

R$_f$: 0.42 (10% ethyl acetate/hexanes)

^{1}H NMR (600 MHz, CDCl$_3$): δ = 4.87 (s, 2H), 2.48 (t, *J* = 7.4 Hz, 2H), 1.68 – 1.60 (m, 2H), 1.34 – 1.28 (m, 6H), 0.88 (t, *J* = 6.9 Hz, 3H).

^{13}C NMR (100 MHz, CDCl$_3$): δ = 201.6, 161.8, 89.3, 71.0, 39.0, 31.6, 28.9, 23.2, 22.6, 14.1.

HRMS (ESI-TOF) *m/z*: calc'd for $C_{10}H_{15}Cl_3O_3Na$ [M+Na]$^+$ 310.9984 found 310.9959.

IR (film) cm^{-1}: 2981, 2971, 2931, 2891, 1775, 1736.

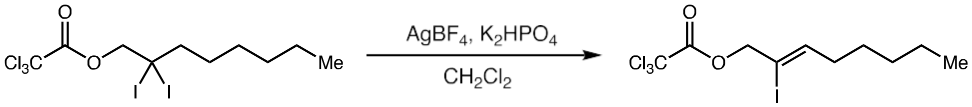

(Z)-2-iodooct-2-en-1-yl 2,2,2-trichloroacetate (32)

Di-iodoester **30** (52.7 mg, 0.1 mmol) was added to a 2-dram vial equipped with a PTFE septum which was then brought into an N_2 filled glove box. To this vial, was added AgOTf (79.1 mg, 0.3 mmol) and flame-dried K_2HPO_4 (40.1 mg, 0.3 mmol). The vial was removed from the glove box and CH_2Cl_2 (3 mL, 0.33 M) was added under N_2. The reaction was stirred for 1.5 hours and concentrated in vacuo. The crude mixture purified (silica gel, pentanes) to yield vinyl iodide **32** (25 mg, 62%) as a clear oil.

R_f: 0.70 (10% ethyl acetate/hexanes)

^{1}H NMR (400 MHz, $CDCl_3$): δ = 6.08 (tt, *J* = 6.6, 1.1 Hz, 1H), 5.03 (q, *J* = 0.9 Hz, 2H), 2.19 (q, *J* = 7.2 Hz, 2H), 1.49 – 1.42 (m, 2H), 1.33 – 1.29 (m, 6H), 0.90 (t, *J* = 7.2 Hz, 3H).

^{13}C NMR (100 MHz, $CDCl_3$): δ = 161.4, 149.6, 142.7, 96.0, 76.2, 36.0, 31.4, 27.7, 22.6, 14.1.

HRMS (ESI-TOF) *m/z*: calc'd for $C_{10}H_{14}Cl_3IO_2Na$ $[M+Na]^+$ 420.9002 found 420.9001.

IR (film) cm^{-1}: 2967, 2930, 2859, 1768, 1216.

IX. Kinetic Experiments

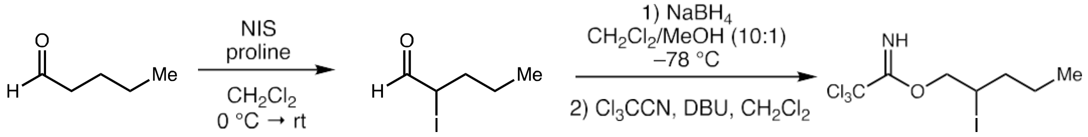

2-iodopentyl 2,2,2-trichloroacetimidate (24)

Step 1: iodination

A flask was charged with pentanal (1.62 g, 2 mL, 18.8 mmol), CH_2Cl_2 (40 mL, 0.5 M), and cooled to 0 °C. N-iodosuccinimide (6.34 g, 28 mmol) and proline (0.44 g, 3.8 mmol) were added after which the reaction was warmed to room temperature and allowed to stir for 8 hours until starting aldehyde was consumed (monitored by ^{1}H NMR). Upon completion, the reaction was diluted with pentanes and filtered. The crude filtrate was concentrated carefully and carried forward without further purification.

R_f: 0.48 (10% ethyl acetate/hexanes)

^{1}H NMR (400 MHz, $CDCl_3$): δ = 9.26 (d, *J* = 3.2 Hz, 1H), 4.47 (td, *J* = 7.4, 3.2 Hz, 1H), 1.96 – 1.90 (m, 2H), 1.57 – 1.49 (m, 1H), 1.44 – 1.35 (m, 1H), 0.96 (t, *J* = 7.4 Hz, 3H).

^{13}C NMR (100 MHz, $CDCl_3$): δ = 191.9, 36.6, 34.3, 22.8, 13.4.

Step 2: Reduction of aldehyde

The crude aldehyde (1 g, 4.7 mmol) was dissolved in CH_2Cl_2 (400 mL, ~0.01 M) and cooled to –78 °C. $NaBH_4$ (178 mg, 4.7 mmol) was added portionwise to the vigorously stirred reaction. Finally, MeOH (40 mL) was added dropwise over ten minutes. Once addition was complete, the crude mixture was checked by TLC to ensure consumption of starting aldehyde (10% ethyl acetate/hexanes) and was quenched at –78 °C with H_2O (20 mL) and brine (20 mL). This solution was allowed to warm to room temperature while stirring and then transferred to a separatory funnel where the organic layer was isolated, dried over $MgSO_4$, and concentrated carefully. This crude oil was carried forward without further purification.

R_f: 0.24 (10% ethyl acetate/hexanes)

^{1}H NMR (600 MHz, $CDCl_3$): δ = 4.26 – 4.22 (m, 1H), 3.77 – 3.68 (m, 2H), 1.94 (dd, *J* = 7.2, 6.4 Hz, 1H), 1.90 – 1.84 (m, 1H), 1.76 – 1.69 (m, 1H), 1.61 – 1.54 (m, 1H), 1.46 – 1.39 (m, 1H), 0.94 (t, *J* = 7.4 Hz, 3H).

^{13}C NMR (100 MHz, $CDCl_3$): δ = 68.8, 41.8, 38.4, 22.8, 13.4.

Step 3: Trichloroacetimidate formation

Crude alcohol was subjected to **GP0**. After concentration, the crude mixture was purified (silica gel, hexanes with 1% Et_3N) to yield trichloroacetimidate **24** (0.9 g, 53% over 3 steps) as a clear oil.

R_f: 0.53 (10% ethyl acetate/hexanes)

^{1}H NMR (400 MHz, $CDCl_3$): δ = 8.73 (bs, 1H), 4.61 (dd, *J* = 11.3, 5.8 Hz, 1H), 4.46 (dd, *J* = 11.3, 7.4, 1H), 4.39 – 4.32 (m, 1H), 1.88 – 1.80 (m, 2H), 1.66 – 1.55 (m, 1H), 1.49 – 1.40 (m, 1H), 0.95 (t, *J* = 7.4 Hz, 3H).

^{13}C NMR (100 MHz, $CDCl_3$): δ = 162.2, 91.2, 73.8, 38.4, 29.4, 22.5, 13.4.

HRMS (ESI-TOF) *m/z*: calc'd for $C_7H_{11}Cl_3INONa$ $[M+Na]^+$ 379.8849, found 379.8846.

IR (film) cm^{-1}: 3343, 2959, 2931, 2873, 1664, 1457.

Initial rate for 1st Iodination

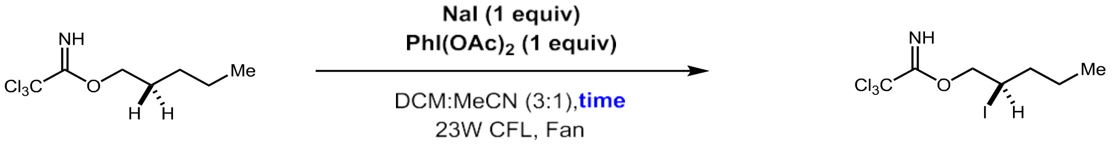

To a 2-dram vial equipped with PTFE septa cap and magnetic stir bar, was added imidate **S2** (47.0 mg, 0.20 mmol), iodobenzene diacetate (64.0 mg, 0.20 mmol), and NaI (30.0 mg, 0.20 mmol). This vial was evacuated and backfilled with N_2 (3x). Dry dichloromethane and acetonitrile (3:1, 0.2 M) were degassed using a freeze-pump-thaw technique (3x), then added to the vial under N_2. The reaction was stirred at 600 rpm, irradiated with two 23 W compact fluorescent light bulbs (~ 2 cm), and cooled by two fans. After the allotted time (i.e. 0, 5, 7, 10 min), the mixture was passed through basic Al_2O_3 (2 cm in monster pipet) and washed with dichloromethane (2 x 5 mL). The solution was concentrated under reduced pressure. The ^{1}H NMR of crude material was analyzed for mono-iodoimidate **24** with isopropyl acetate (23 µL, 0.20 mmol) as an internal standard.

Note: Before 10 min, the ^{1}H NMR of crude material showed a mixture of starting imidate and mono-iodoimidate **24**. After 10 min, the ^{1}H NMR of crude material showed the formation of di-iodoimidate **2** in the mixture. Time points before 10 min were chosen to avoid any convolution from the di-iodoimidate **2**.

Analysis: The NMR yields of mono-iodoimidate **24** were plotted against time in minutes. The slope through four points was utilized to calculate the initial rate of the reaction. The procedure was repeated and the average of three runs was taken. Calculated values are tabulated below.

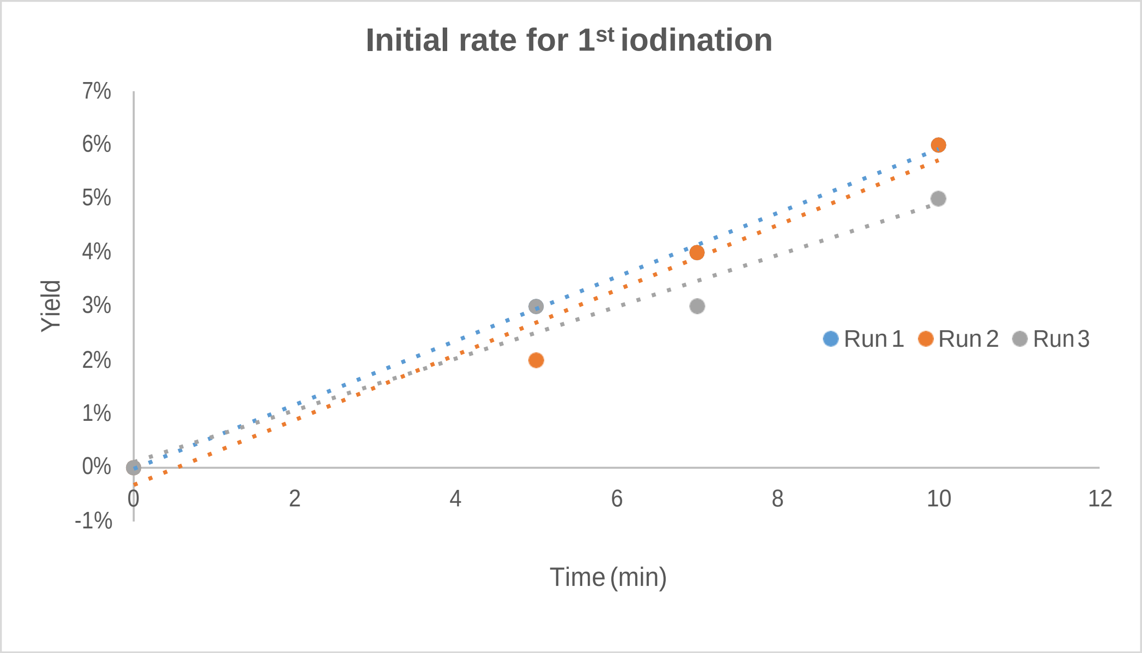

Figure S2: Initial rates for 1st iodination.

Table S11: Initial rate for 1st iodination.

Run	Initial rate (min^{-1})	R^2
1	0.0059	0.9985
2	0.0060	0.9660
3	0.0048	0.9623
**Avg.**	**0.0056**	0.9756

Initial Rate for 2nd Iodination

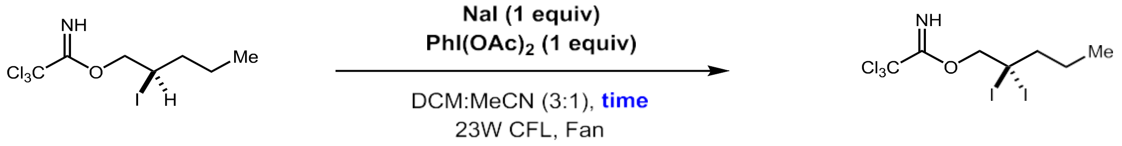

To a 2-dram vial equipped with PTFE septa cap and magnetic stir bar, was added mono-iodoimidate **24** (72.0 mg, 0.20 mmol), iodobenzene diacetate (64.0 mg, 0.20 mmol), and NaI (30.0 mg, 0.20 mmol). This vial was evacuated and backfilled with N_2 (3x). Dry dichloromethane and acetonitrile (3:1, 0.2 M) were degassed using a freeze-pump-thaw technique (3x), then added to the vial under N_2. The reaction was stirred at 600 rpm, irradiated with two 23 W compact fluorescent light bulbs (~ 2 cm), and cooled by two fans. After the allotted time (i.e. 25, 30, 35 and 40 min), the mixture was passed through basic Al_2O_3 (2 cm in monster pipet) and washed with dichloromethane (2x5 mL). The solution was concentrated under reduced pressure. The ^{1}H NMR of crude material was analyzed for di-iodoimidate **2** with isopropyl acetate (23 µL, 0.20 mmol) as an internal standard.

Note: Before 25 mins, the ^{1}H NMR of crude material showed di-iodoimidate **2** in 1–3 % yield. To ensure reproducible kinetic data, time points were chosen at 25 min and beyond.

Analysis: The NMR yields of di-iodoimidate **2** were plotted against time in minutes. The slope through four points was utilized to calculate the initial rate of the reaction. The procedure was repeated and the average of three runs was taken. Calculated values are tabulated below.

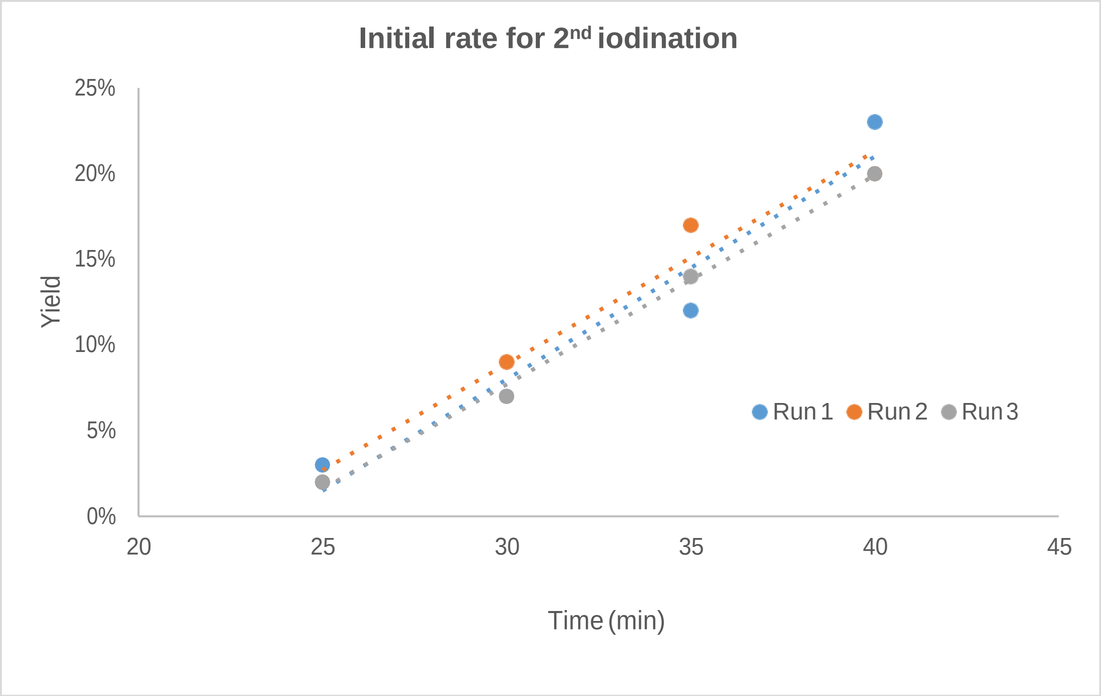

Figure S3: Initial rate for 2nd iodination.

Table S12: Initial rate for 2nd iodination

Run	Initial Rate (min^{-1})	R^2
1	0.0130	0.9399
2	0.0124	0.9707
3	0.0122	0.9963
**Avg.**	**0.0125**	0.9690

According to table S1 and S2, the average rate for the second iodination (12.5 x 10^3 min^{-1}) is 2.2 x as fast as the first iodination (5.6 x 10^3 min^{-1}).

Reaction Rate Profile for Di-iodination

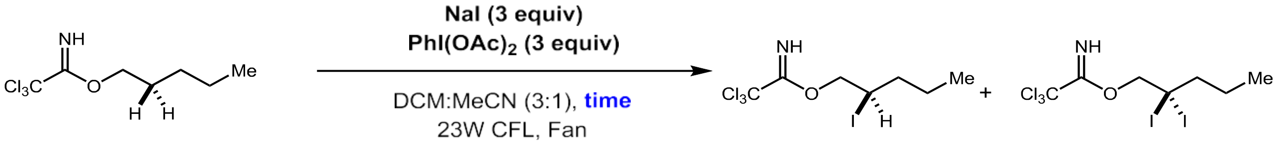

To a 2-dram vial equipped with PTFE septa cap and magnetic stir bar, was added imidate **S2** (47.0 mg, 0.20 mmol), iodobenzene diacetate (0.19 g, 0.60 mmol), and NaI (90.0 mg, 0.60 mmol). This vial was evacuated and backfilled with N_2 (3x). Dry dichloromethane and acetonitrile (3:1, 0.2 M) were degassed using a freeze-pump-thaw technique (3x), then added to the vial under N_2. The reaction was stirred at 600 rpm, irradiated with two 23 W compact fluorescent light bulbs (~ 2 cm), and cooled by two fans. After the allotted time, the mixture was passed through basic Al_2O_3 (2 cm in monster pipet) and washed with dichloromethane (2x5 mL). The solution was concentrated under reduced pressure. The ^{1}H NMR of crude material was analyzed with isopropyl acetate (23 µL, 0.20 mmol) as an internal standard.

The NMR yields of the product were plotted against time in minutes. The procedure was repeated and the average of three runs was taken.

Table S13: Iodination reaction at different time points.

**Time (min)**	**Di-iodoimidate (2)**	**Mono-iodoimidate (24)**	**Starting imidate (S2)**
5	0%	7%	90%
10	1%	8%	84%
13	7%	28%	55%
15	14%	29%	35%
20	62%	16%	7%
30	70%	19%	0%
32	71%	10%	0%
120	85%	0%	0%

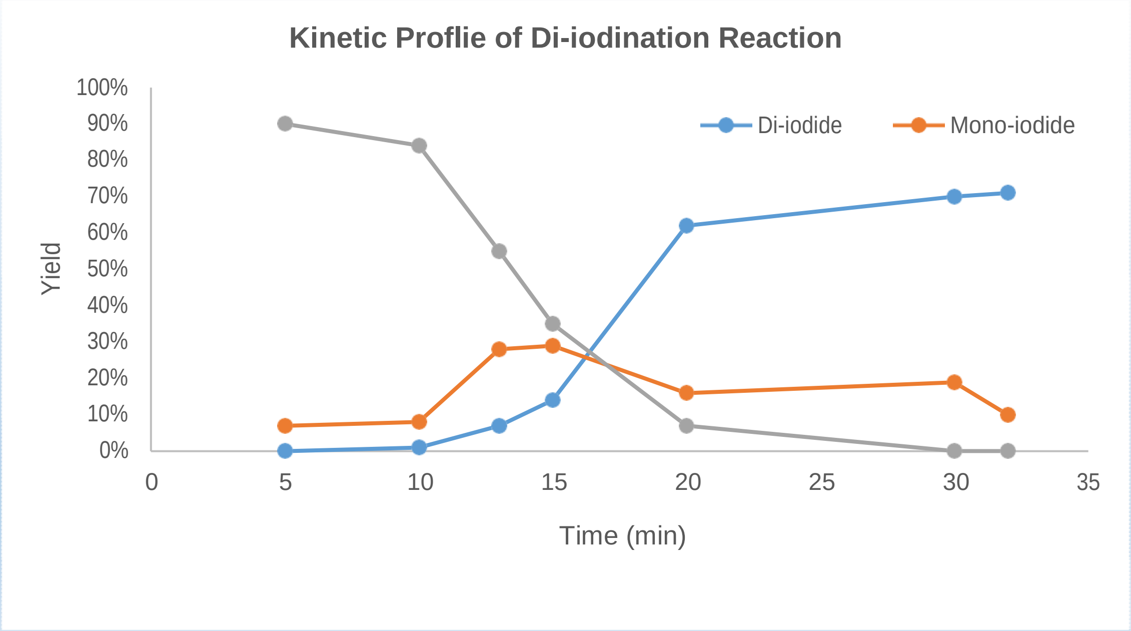

Figure S4: Kinetic profile of di-iodination reaction.

X. Intermolecular Halogenation Competition

I vs Cl

Imidate **S1** (49 mg, 0.2 mmol), NaI (45 mg, 0.3 mmol), NaCl (18 mg, 0.3 mmol) and Bu_4NCl (28 mg, 0.1 mmol) were subjected to **GP1** for entry 1 and **GP3** for entry 2. Upon completion, the solution was concentrated. All crude yields were determined via ^{1}H NMR (Isopropyl acetate as an internal standard).

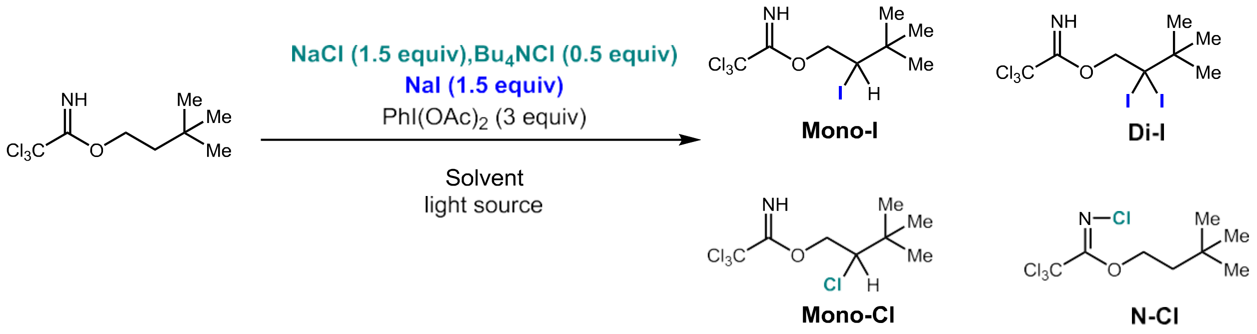

Table S14: Solvent and light source effect on iodination vs chlorination competition.

Entry	Solvent	Light Source	time	Mono-I	Di-I	Mono-Cl	N-Cl	SM
1	CH_2Cl_2:MeCN (3:1)	23 W	2 h	40%	47%	0%	0%	0%
2	HFIP:CH_2Cl_2 (3:1)	UV (300 nm)	26 h	0%	0%	20%	26%	53%

Crude NMR of entry 1

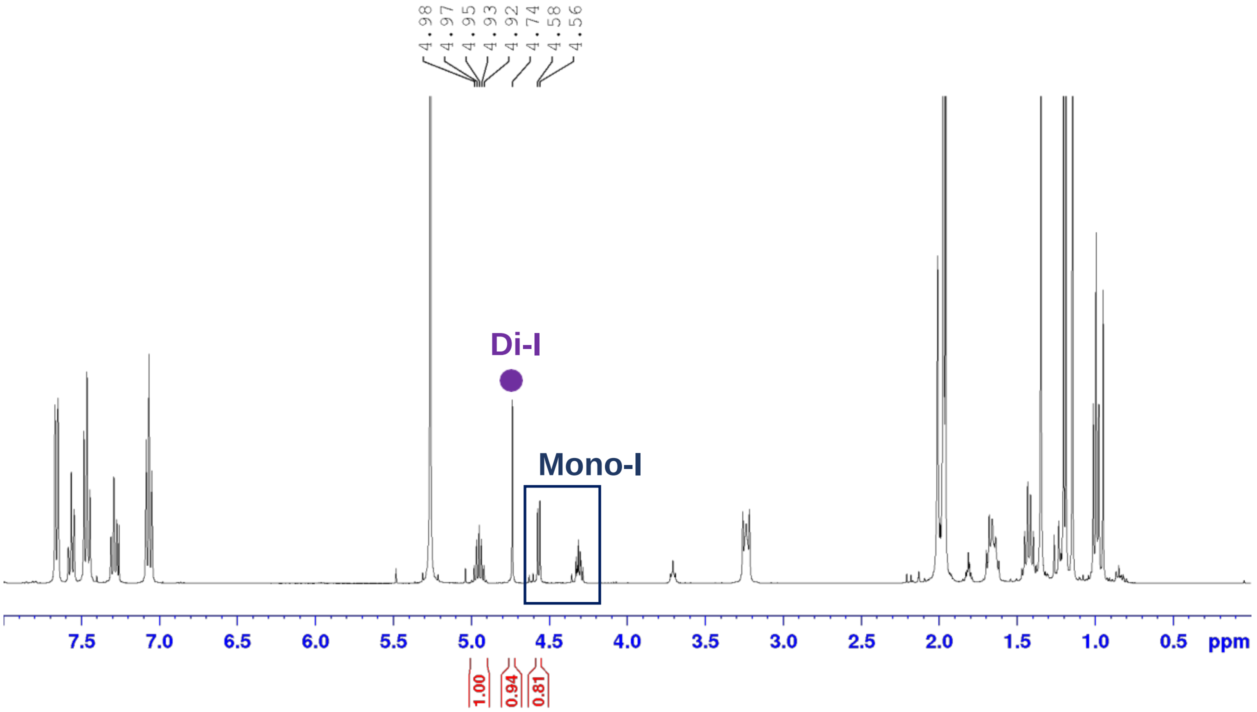

Crude NMR of entry 2

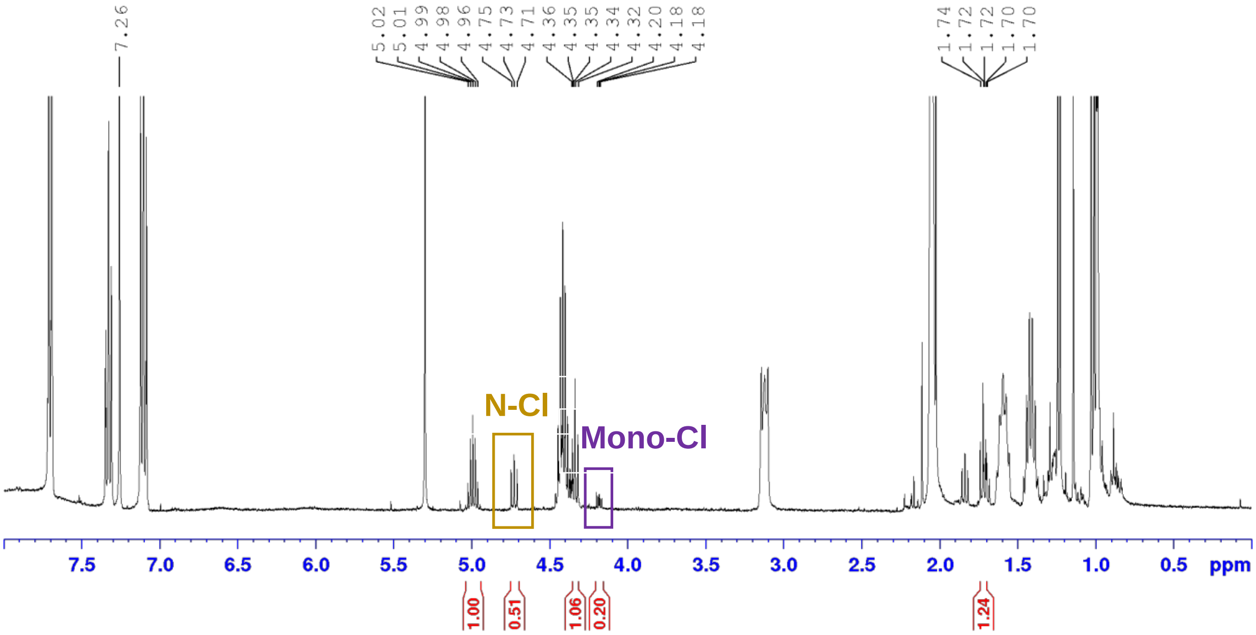

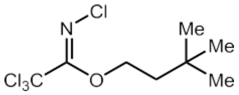

3,3-dimethylbutyl-2,2,2-tetrachloroacetimidate (20')

Colorless oil.

R_f: 0.65 (10% ethyl acetate/hexanes)

^{1}H NMR (400 MHz, $CDCl_3$): δ = 4.71 (t, J = 7.4 Hz, 2H), 1.82 (t, J = 7.5 Hz, 2 H), 0.98 (s, 9H).

^{13}C NMR (150 MHz, $CDCl_3$): δ = 164.7, 91.9, 74.7, 42.8, 29.9, 29.8.

HRMS (ESI-TOF) *m/z*: calc'd for $C_8H_{13}Cl_4NONa$ $[M+Na]^+$ 301.9649 found 301.9650.

IR (film) cm^{-1}: 2958, 2868, 1623, 1469, 1367, 1214, 1033, 999.

I vs Br

Imidate **S1** (49 mg, 0.2 mmol), NaI (45 mg, 0.3 mmol) and NaBr (31 mg, 0.3 mmol) were subjected to **GP1**. After 0.5 to 2 h, the solution was concentrated. All crude yields were determined via ^{1}H NMR (Isopropyl acetate as an internal standard).

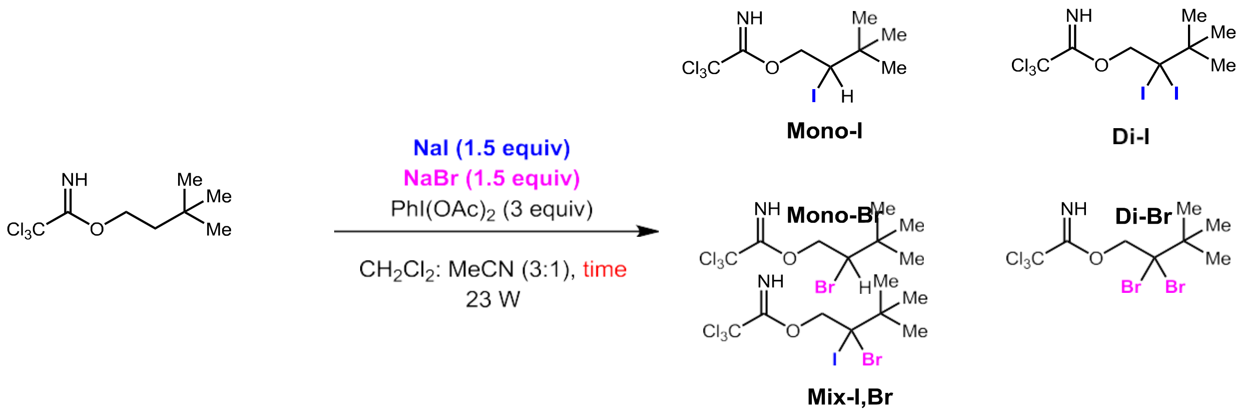

Table S15: Time study iodination vs bromination competition.

Entry	Time (h)	Mono-I	Di-I	Mono-Br	Di-Br	Mix-I,Br	SM
1	0.5	5%	2%	10%	1%	2%	21%
2	1	5%	15%	21%	18%	32%	7%
3	1.5	6%	15%	15%	19%	33%	4%
4	2	5%	19%	9%	16%	33%	4%

Crude NMR of entry 4

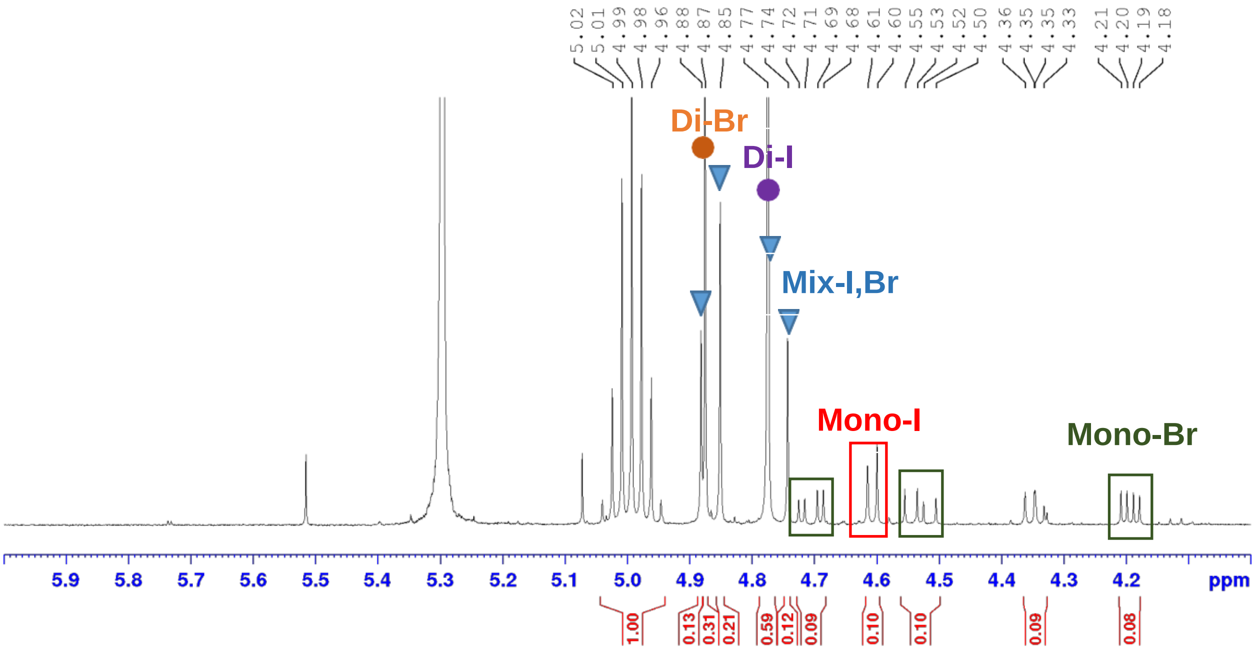

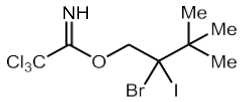

2-bromo-2-iodo-3,3-dimethylbutyl 2,2,2-trichloroacetimidate

^{1}H NMR (400 MHz, $CDCl_3$): δ = 8.47 (bs, 1H), 4.87 (d, *J* = 12.3 Hz, 1H), 4.76 (d, *J* = 12.9 Hz, 1H), 1.39 (s, 9H).

HRMS (ESI-TOF) *m/z*: calc'd for $C_8H_{12}BrCl_3INONa$ $[M+Na]^+$ 449.8246, found 449.8291.

Imidate **S1** (49 mg, 0.2 mmol), NaI (45 mg, 0.3 mmol), NaBr (31 mg, 0.3 mmol) were subjected to **GP1** for entry 1 and 2, and **GP2** for entry 3 and 4. For entry 2 and 4, Bu_4NBr (32 mg, 0.1 mmol) was added to the reaction. After 2 h, the solution was concentrated. All crude yields were determined via ^{1}H NMR (Isopropyl acetate as an internal standard).

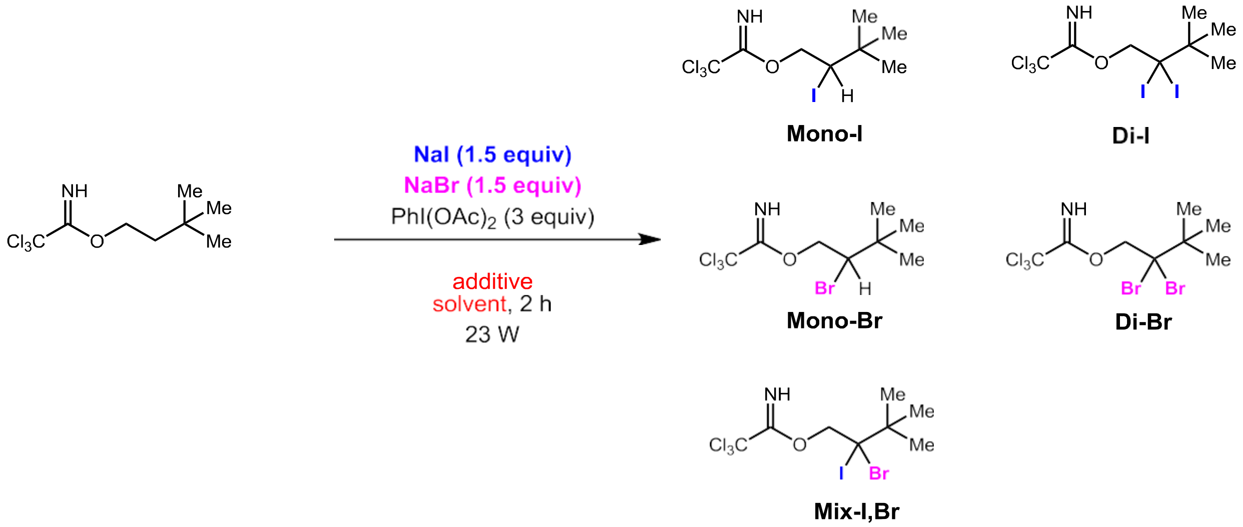

Table S16: Solvent effect on iodination vs bromination competition.

Entry	Solvent (3:1)	Additive	Mono-I	Di-I	Mono-Br	Di-Br	Mix- I,Br	SM
1	CH_2Cl_2:MeCN	–	5%	19%	9%	16%	33%	4%
2	CH_2Cl_2:MeCN	Bu_4NBr (1 equiv)	5%	10%	28%	24%	24%	11%
3	HFIP:CH_2Cl_2	–	0%	0%	17%	4%	0%	56%
4	HFIP:CH_2Cl_2	Bu_4NBr (1 equiv)	0%	0%	11%	2%	0%	67%

Br vs Cl

Imidate **S1** (49 mg, 0.2 mmol), NaBr (31 mg, 0.3 mmol), NaCl (17 mg, 0.3 mmol), Bu_4NBr (32 mg, 0.1 mmol) and Bu_4NCl (27 mg, 0.1 mmol) were subjected to **GP1** for entry 1, **GP2** for entry 2 and **GP3** for entry 3. After 2 h, the solution was concentrated. All crude yields were determined via ^{1}H NMR (Isopropyl acetate as an internal standard).

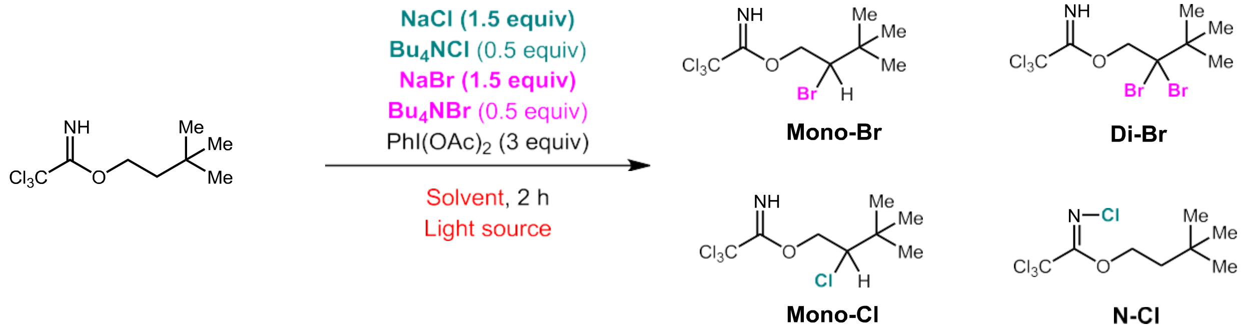

Table S17: Solvent effect on iodination vs bromination competition.

Entry	Solvent	Light Source	Mono-Br	Di-Br	Mono-Cl	N-Cl	SM
1	CH_2Cl_2:MeCN (3:1)	23 W	42%	26%	0%	0%	16%
2	HFIP:CH_2Cl_2(3:1)	23 W	27%	10%	0%	33%	0%
3	HFIP:CH_2Cl_2 (3:1)	UV (300 nm)	26%	27%	0%	17%	0%

Crude NMR of entry 3

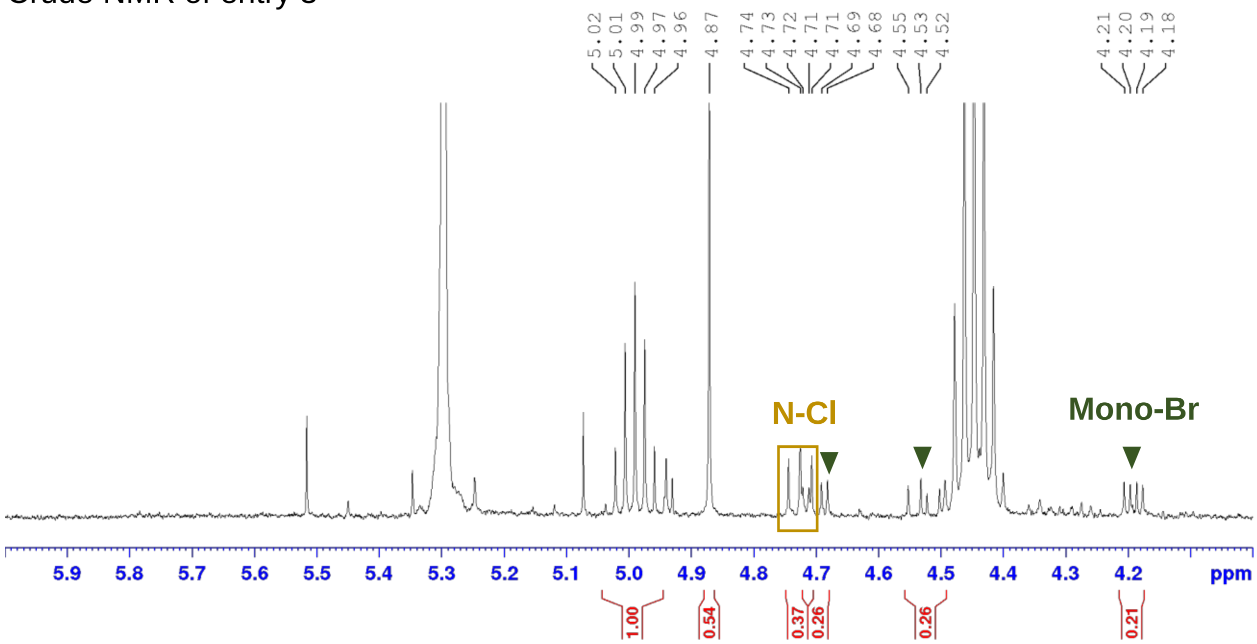

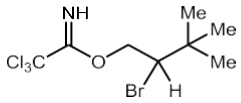

2-bromo-3,3-dimethylbutyl 2,2,2-trichloroacetimidate

Using conditions similar to the mono-iodination, mono-bromination can be achieved.

To a 2-dram vial equipped with a PTFE septum cap and magnetic stir bar, was added imidate **S1** (0.20 mmol, 49 mg) and *N*-bromosuccinimide (0.20 mmol, 36 mg). This vial was evacuated and backfilled with N_2 (3x). Dry, degassed acetonitrile (1 mL) was added to the vial under N_2. The reaction was irradiated with two 23 W compact fluorescent light bulbs for 2 hours. Upon completion, the solution was concentrated. A crude yield of mono-bromoimidate (53%) and di-bromoimidate **18** (13%) (mono:di 4:1) were determined via ^{1}H NMR (Isopropyl acetate as an internal standard).

^{1}H NMR (400 MHz, $CDCl_3$): δ = 8.32 (bs, 1H), 4.64 – 4.60 (m, 1H), 4.45 – 4.40 (m, 1H), 4.13 – 4.10 (m, 1H), 1.92 (s, 9H).

^{13}C NMR (100 MHz, $CDCl_3$): δ = 162.6, 91.2, 71.1, 63.2, 35.2, 27.9 (x3).

HRMS (ESI-TOF) *m/z*: calc'd for $C_8H_{13}Cl_3BrNONa$ $[M+Na]^+$ 345.9144, found 345.9122.

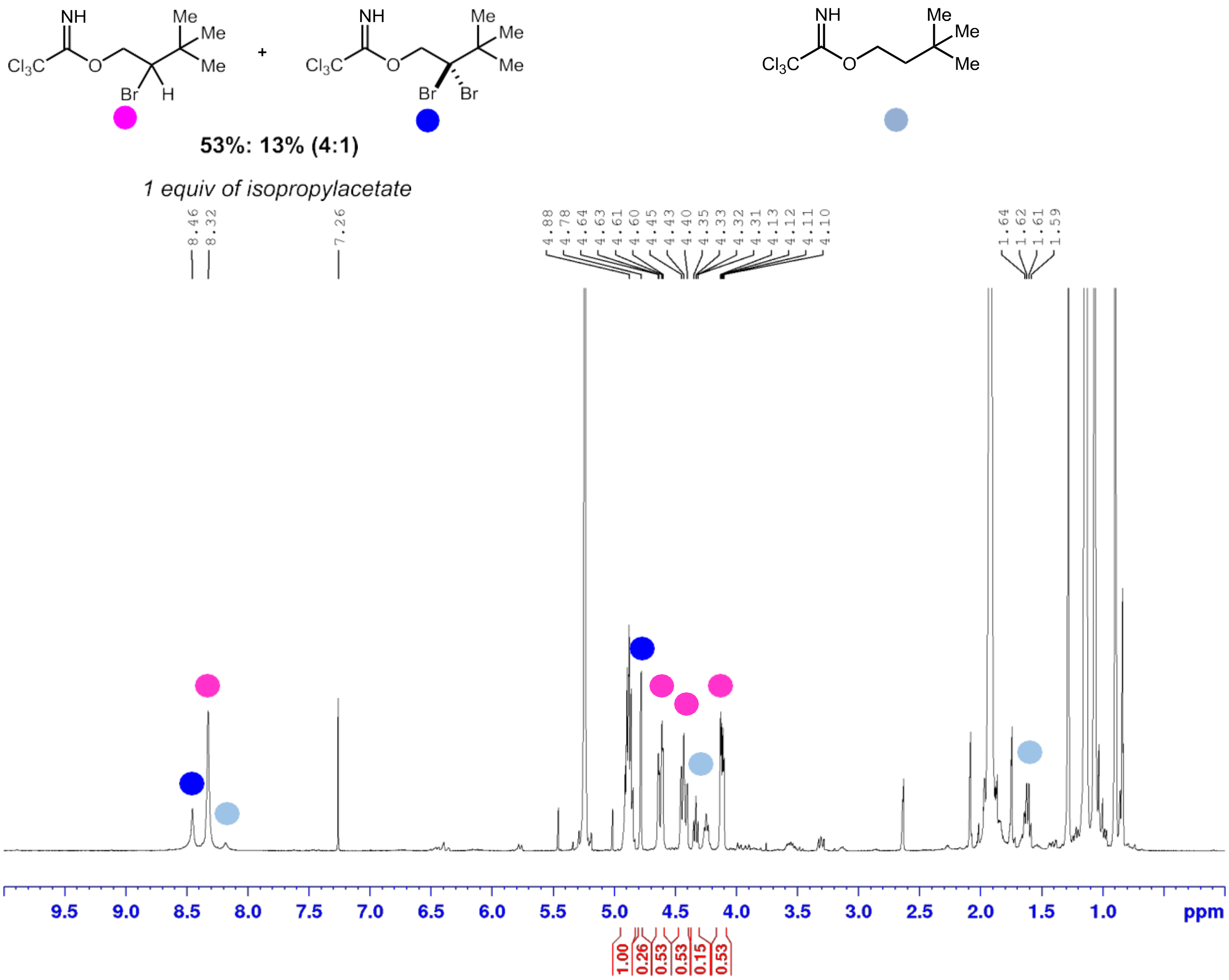
NH
Me
Me
Cl3C
O
Me
Br
H
+
NH
Me
Me
Cl3C
O
Me
Br
Br
NH
Me
Me
Cl3C
O
Me
53%: 13% (4:1)
1 equiv of isopropylacetate
8.46
8.32
7.26
4.88
4.78
4.64
4.63
4.61
4.60
4.45
4.43
4.40
4.35
4.33
4.32
4.31
4.13
4.12
4.11
4.10
1.64
1.62
1.61
1.59
9.5
9.0
8.5
8.0
7.5
7.0
6.5
6.0
5.5
5.0
4.5
4.0
3.5
3.0
2.5
2.0
1.5
1.0
ppm
1.00
0.26
0.53
0.53
0.15
0.53

XI. Bond Dissociation Enthalpy (BDE) Calculations

The structures of various C-centered radicals were optimized using Spartan ’16 (see Section **XII** for details). We have performed Density functional theory (DFT) studies to determine the homolytic C-H BDEs at β position of halo-imidates.

Structure optimization: ωB97X-D/6-31G(D)

Fragmentation Reaction	BDE (kcal/mol)
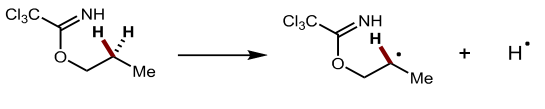	107.0
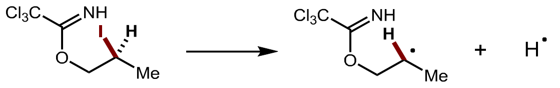	103.9
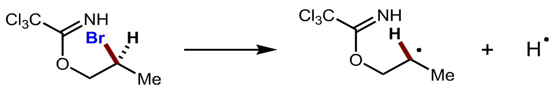	102.2
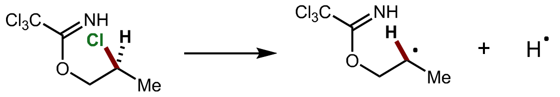	101.8

XII. Computational Studies

a. Computational Methods

Density functional theory (DFT) calculations were performed using Spartan ‘16[9]. All geometries were optimized at the ωB97X-D/6-31G(D)[10] level of theory and used for the determination of homolytic bond dissociation enthalpies (BDE).

C-Centered Radical	Total Energy (hartrees)

[9] Shao, Y; Molnar, L. F.; Jung, Y.; Kussmann, J.; Ochsenfeld, C.; Brown, S. T.; Gilbert, A. T. B.; Slipchenko, L. V.; Levchenko, S. V.; O’Neill, D. P.; DiStasio Jr., R. A.; Lochan, R. C.; Wang, T.; Beran, G. J. O.; Besley, N. A.; Herbert, J. M.; Lin, C. Y.; Van Voorhis, T.; Chien, S. H.; Sodt, A.; Steele, R. P.; Rassolov, V. A.; Maslen, P. E.; Korambath, P. P.; Adamson, R. D.; Austin, B.; Baker, J.; Byrd, E. F. C.; Dachsel, H.; Doerksen, R. J.; Dreuw, A.; Dunietz, B. D.; Dutoi, A. D.; Furlani, T. R.; Gwaltney, S. R.; Heyden, A.; Hirata, S.; Hsu, C.-P.; Kedziora, G.; Khalliulin, R. Z.; Klunzinger, P.; Lee, A. M.; Lee, M. S.; Liang, W. Z.; Lotan, I.; Nair, N; Peters, B.; Proynov, E. I.; Pieniazek, P. A.; Rhee, Y. M.; Ritchie, J.; Rosta, E.; Sherrill, C. D.; Simmonett, A. C.; Subotnik, J. E.; Woodcock III, H. L.; Zhang, W.; Bell, A. T.; Chakraborty, A. K.; Chipman, D. M.; Keil, F. J.; Warshel, A.; Hehre, W. J.; Schaefer, H. F.; Kong, J.; Krylov, A. I.; Gill, P. M. W.; Head-Gordon, M. *Phys. Chem. Chem. Phys.* **2006**, 8, 3172.

[10] Chai, J.-D.; Head-Gordon, M. Long-range Corrected Hybrid Density Functionals with Damped Atom−atom Dispersion Corrections. Phys. Chem. Chem. Phys. 2008, 10, 6615.

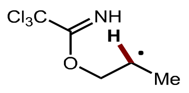	–1705.06291
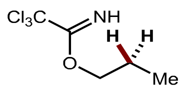	–1705.73408
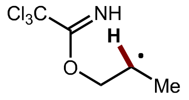	–1715.861902
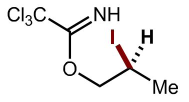	–1716.52805
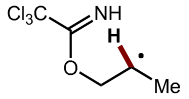	–4278.387273
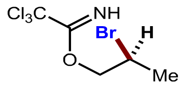	–4279.050742
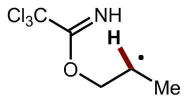	–2164.652056
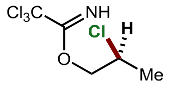	–2165.314876

b. Optimized Cartesian Coordinates

N-Centered Radical	Cartesian Coordinates
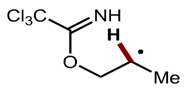	N N1 -1.1210306 0.9855448 -1.7968687<br>C C1 -0.4466201 1.4213194 -0.8233762<br>C C2 -0.1460772 2.8957208 -0.4536662<br>O O1 0.1317653 0.6374360 0.0797117<br>C C3 -0.0280928 -0.7763366 -0.1079635<br>C C4 0.7243961 -1.4702201 0.9649135<br>C C5 0.4516421 -2.9017752 1.2683367<br>H H1 1.6298047 -0.9954892 1.3308143<br>Cl Cl1 1.6205138 3.1459976 -0.4276270<br>Cl Cl2 -0.8689195 4.0135279 -1.6415486<br>Cl Cl3 -0.8320864 3.2401752 1.1575011<br>H H2 -1.4848201 1.7338365 -2.3807797<br>H H3 0.3306914 -1.0277063 -1.1194520<br>H H4 -1.0978001 -1.0235921 -0.0893613<br>H H5 0.8994029 -3.5785622 0.5211638<br>H H6 -0.6252106 -3.1134325 1.2765901<br>H H7 0.8624410 -3.1864438 2.2416120

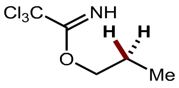	N N1 -1.8752624 -0.0110733 1.5267824<br>C C1 -0.6239034 -0.0028277 1.6884543<br>C C2 0.1685999 0.0050699 3.0205374<br>Cl Cl1 1.1910367 1.4668709 3.0819093<br>Cl Cl2 -0.9342084 0.0115863 4.4225594<br>Cl Cl3 1.1929189 -1.4548621 3.0970059<br>O O1 0.2480491 0.0023601 0.6868271<br>C C3 -0.2928416 -0.0046763 -0.6407695<br>C C4 0.8796424 0.0059707 -1.6027676<br>C C5 0.4090498 -0.0007644 -3.0563457<br>H H1 1.4916054 0.8930124 -1.4052799<br>H H2 1.5095740 -0.8678641 -1.4028976<br>H H3 -2.3790944 -0.0126122 2.4097173<br>H H4 -0.9361466 0.8731306 -0.7654691<br>H H5 -0.9180190 -0.8958308 -0.7631706<br>H H6 1.2630055 0.0065602 -3.7396443<br>H H7 -0.1883395 -0.8923307 -3.2774207<br>H H8 -0.2056666 0.8782806 -3.2800283
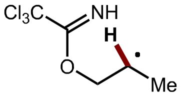	C C1 -1.0043551 -0.8432660 -2.5725332<br>C C2 -0.3480233 -1.1465454 -1.2032725<br>N N1 -0.0228458 -2.2679862 -0.7254692<br>O O1 -0.1635631 0.0038634 -0.5616087<br>Cl Cl1 -1.3039463 -2.3497852 -3.4807983<br>Cl Cl2 0.0847405 0.2012781 -3.5210732<br>Cl Cl3 -2.5612428 -0.0144679 -2.3019986<br>C C3 0.4887825 -0.0883755 0.7149377<br>C C4 0.7100064 1.2840774 1.2419923<br>C C5 0.3601363 1.6781237 2.6363981<br>I I1 1.9276181 2.5526756 0.1279320<br>H H1 -0.2352705 -3.0345488 -1.3583905<br>H H2 1.4203286 -0.6535962 0.5819246<br>H H3 -0.1498510 -0.6593905 1.3982117<br>H H4 1.2062893 1.5463519 3.3274181<br>H H5 -0.4687461 1.0621618 3.0029960<br>H H6 0.0599421 2.7294297 2.6933337
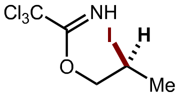	C C1 -0.8855414 -1.8542382 -2.2167451<br>C C2 -0.1119916 -1.5481215 -0.9115925<br>O O1 -0.3705701 -0.2917872 -0.5539004<br>N N1 0.6490228 -2.3088919 -0.2545407<br>Cl Cl1 -2.6315696 -1.6202834 -1.9279465<br>Cl Cl2 -0.6066101 -3.5377880 -2.7372645<br>Cl Cl3 -0.3252980 -0.7472558 -3.4946319<br>C C3 0.3014055 0.1825719 0.6072263<br>C C4 -0.0512557 1.6424915 0.8045456<br>I I1 0.6887651 2.8338058 -0.8634719<br>C C5 0.5002028 2.1730740 2.1199934<br>H H1 0.7355440 -3.2297453 -0.6760471<br>H H2 1.3798321 0.0283718 0.5035974<br>H H3 -0.0400689 -0.3949510 1.4759208<br>H H4 -1.1336354 1.7692586 0.7504476<br>H H5 1.5893435 2.0772505 2.1628623<br>H H6 0.0698797 1.6014011 2.9516816<br>H H7 0.2425452 3.2248372 2.2598655
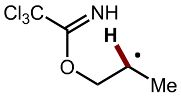	C C1 -0.2754530 -1.2694735 0.9072024<br>N N1 -0.7298145 -1.0348116 2.0602157<br>C C2 0.2545849 -2.6097750 0.3394666<br>Cl Cl1 0.1303715 -3.9103441 1.5539325<br>Cl Cl2 1.9689201 -2.4053995 -0.1174157<br>Cl Cl3 -0.7061230 -3.0600213 -1.0928637<br>O O1 -0.1748177 -0.3418152 -0.0412949<br>C C3 -0.6351547 0.9820626 0.3198397<br>C C4 -0.4274926 1.8740165 -0.8471889<br>Br Br1 -1.7825383 1.8908574 -2.1549763<br>C C5 0.9370373 2.2980733 -1.2674257<br>H H1 -0.7337123 -1.8601530 2.6531620<br>H H2 -0.0426193 1.3260961 1.1750976<br>H H3 -1.6812122 0.9183688 0.6279254<br>H H4 1.4293366 1.5139105 -1.8613809<br>H H5 0.9115044 3.2092919 -1.8717241

	H H6 1.5571826 2.4791161 -0.3825717
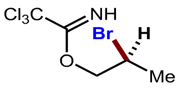	C C1 -0.0462444 -1.6743784 0.6536642<br>N N1 0.3745879 -1.5180746 1.8319531<br>C C2 -0.4469389 -2.9889115 -0.0583976<br>Cl Cl1 -0.2841591 -4.3806855 1.0463092<br>Cl Cl2 0.6147920 -3.2336312 -1.4668585<br>Cl Cl3 -2.1477189 -2.8703263 -0.5884426<br>O O1 -0.2119632 -0.6661818 -0.2000596<br>C C3 0.1549849 0.6248062 0.2727632<br>C C4 -0.0469680 1.6193944 -0.8515974<br>Br Br1 1.1932288 1.2235753 -2.3283989<br>C C5 0.1521825 3.0483110 -0.3712466<br>H H1 0.4426617 -2.3964439 2.3390592<br>H H2 1.1910011 0.6121551 0.6247362<br>H H3 -0.4887476 0.8912352 1.1201174<br>H H4 -1.0393010 1.4845529 -1.2852409<br>H H5 1.1542688 3.1884024 0.0452707<br>H H6 0.0183447 3.7567933 -1.1912064<br>H H7 -0.5840113 3.2794072 0.4075755
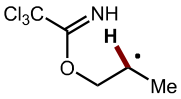	C C1 0.0184445 -1.5288457 0.3923586<br>N N1 0.4913328 -1.8096739 1.5275107<br>C C2 -0.7849118 -2.4443238 -0.5647439<br>Cl Cl1 -0.9884782 -4.0734409 0.1325699<br>Cl Cl2 0.0852164 -2.5792594 -2.1145281<br>Cl Cl3 -2.3975156 -1.7248904 -0.8329199<br>O O1 0.1414958 -0.3322783 -0.1758371<br>C C3 0.8429729 0.6682881 0.6015420<br>C C4 0.8157789 1.9388713 -0.1619118<br>Cl Cl4 2.0869082 2.1873129 -1.3129208<br>C C5 -0.4465996 2.6994788 -0.3717377<br>H H1 0.3002224 -2.7695588 1.8016440<br>H H2 1.8576711 0.3149196 0.7983552<br>H H3 0.3220402 0.7811675 1.5584154<br>H H4 -0.2488712 3.7539938 -0.5858955<br>H H5 -1.0205837 2.2862524 -1.2140878<br>H H6 -1.0751231 2.6319870 0.5221868
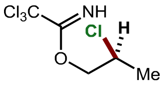	N N1 0.1742894 1.5513626 -1.8352055<br>C C1 -0.0617935 1.6919056 -0.6045498<br>C C2 -0.3361105 2.9979885 0.1794809<br>Cl Cl1 -0.3099891 4.4102607 -0.9102916<br>Cl Cl2 -1.9462552 2.8880333 0.9436769<br>Cl Cl3 0.9181480 3.2020089 1.4278653<br>O O1 -0.1150476 0.6714055 0.2484735<br>C C3 0.1289218 -0.6202421 -0.2978158<br>C C4 0.0322718 -1.6313457 0.8266162<br>C C5 0.0869557 -3.0562957 0.2978374<br>Cl Cl4 1.3712443 -1.3626277 2.0144383<br>H H1 0.1770310 2.4374863 -2.3332854<br>H H2 1.1114995 -0.6424149 -0.7788361<br>H H3 -0.6303062 -0.8369275 -1.0583930<br>H H4 -0.8886665 -1.4587615 1.3875785<br>H H5 1.0117425 -3.2314455 -0.2602436<br>H H6 -0.7639983 -3.2376600 -0.3680309<br>H H7 0.0400630 -3.7727307 1.1206846

XIII. X-ray Crystallographic Data

Table 1. Crystallographic details for 2,2-dibromo-3,3-dimethylbutyl 2,2,2-trichloro acetimidate **(19)**

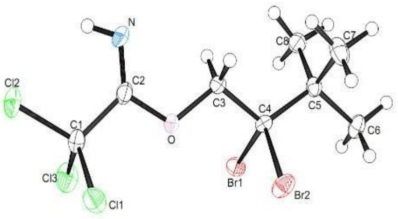

Empirical formula	C8 H12 Br2 Cl3 N O
Formula weight	404.36
Temperature	150(2) K
Wavelength	0.71073 Å
Crystal system	Monoclinic
Space group	C 2/c
Unit cell dimensions	a = 18.3209(5) Å
	b = 12.3996(3) Å
	c = 12.1068(3) Å
	β= 98.766(1)°
Volume	2718.20(12) $Å^3$
Z	8
Density (calculated)	1.976 Mg/m^3
Absorption coefficient	6.530 mm^{-1}
F(000)	1568
Crystal size	0.04 x 0.04 x 0.23 mm^3
Theta range for data collection	3.286 to 27.887°
Index ranges	-24<=h<=24, -16<=k<=16, -15<=l<=15
Reflections collected	36801
Independent reflections	3252 [R(int) = 0.0283]
Completeness to theta = 25.242°	99.8 %
Refinement method	Full-matrix least-squares on F^2
Data / restraints / parameters	3252 / 0 / 141
Goodness-of-fit on F^2	1.074
Final R indices [I>2sigma(I)]	R1 = 0.0177, wR2 = 0.0392
R indices (all data)	R1 = 0.0214, wR2 = 0.0404
Largest diff. peak and hole	0.527 and -0.351 e/$Å^3$

Table 2. Atomic coordinates (x 10^4) and equivalent isotropic displacement parameters ($Å^2$x 10^3) for 2,2-dibromo-3,3-dimethylbutyl 2,2,2-trichloro acetimidate **(19)**. U(eq) is defined as one third of the trace of the orthogonalized U^{ij} tensor.

	x	y	z	U(eq)
C(1)	5142(1)	7445(1)	4827(2)	23(1)
C(2)	4520(1)	6831(1)	5281(1)	22(1)
C(3)	3381(1)	5987(1)	4737(1)	20(1)
C(4)	2866(1)	5671(1)	3685(1)	16(1)
C(5)	2187(1)	5010(1)	3927(1)	18(1)
C(6)	1687(1)	4692(2)	2845(2)	25(1)
C(7)	1725(1)	5679(2)	4637(2)	31(1)
C(8)	2450(1)	3982(2)	4579(2)	25(1)
N	4502(1)	6578(2)	6252(1)	44(1)
O	3987(1)	6604(1)	4435(1)	24(1)
Cl(1)	4779(1)	8575(1)	4037(1)	31(1)
Cl(2)	5806(1)	7888(1)	5946(1)	36(1)
Cl(3)	5568(1)	6578(1)	3955(1)	35(1)
Br(1)	3453(1)	4851(1)	2737(1)	23(1)
Br(2)	2549(1)	7005(1)	2870(1)	27(1)

Table 3. Bond lengths [Å] and angles [°]

C(1)-C(2)	1.541(2)
C(1)-Cl(2)	1.7649(17)
C(1)-Cl(1)	1.7680(18)
C(1)-Cl(3)	1.7694(18)
C(2)-N	1.222(2)
C(2)-O	1.3327(19)
C(3)-O	1.4408(19)
C(3)-C(4)	1.516(2)
C(3)-H(3A)	0.9900
C(3)-H(3B)	0.9900
C(4)-C(5)	1.554(2)
C(4)-Br(2)	1.9691(16)
C(4)-Br(1)	1.9707(16)
C(5)-C(6)	1.531(2)
C(5)-C(7)	1.538(2)
C(5)-C(8)	1.539(2)
C(6)-H(6A)	0.9800
C(6)-H(6B)	0.9800
C(6)-H(6C)	0.9800
C(7)-H(7A)	0.9800
C(7)-H(7B)	0.9800
C(7)-H(7C)	0.9800
C(8)-H(8A)	0.9800
C(8)-H(8B)	0.9800
C(8)-H(8C)	0.9800
N-H(1N)	0.816
N-H(2N)	0.662
C(2)-C(1)-Cl(2)	109.96(12)
C(2)-C(1)-Cl(1)	110.28(12)
Cl(2)-C(1)-Cl(1)	109.12(10)
C(2)-C(1)-Cl(3)	109.27(12)
Cl(2)-C(1)-Cl(3)	109.36(9)
Cl(1)-C(1)-Cl(3)	108.83(10)

N-C(2)-O	124.17(16)
N-C(2)-C(1)	126.81(16)
O-C(2)-C(1)	109.01(14)
O-C(3)-C(4)	109.21(13)
O-C(3)-H(3A)	109.8
C(4)-C(3)-H(3A)	109.8
O-C(3)-H(3B)	109.8
C(4)-C(3)-H(3B)	109.8
H(3A)-C(3)-H(3B)	108.3
C(3)-C(4)-C(5)	113.05(13)
C(3)-C(4)-Br(2)	107.58(11)
C(5)-C(4)-Br(2)	110.70(10)
C(3)-C(4)-Br(1)	107.33(11)
C(5)-C(4)-Br(1)	111.21(10)
Br(2)-C(4)-Br(1)	106.69(7)
C(6)-C(5)-C(7)	107.99(14)
C(6)-C(5)-C(8)	109.01(14)
C(7)-C(5)-C(8)	108.40(15)
C(6)-C(5)-C(4)	111.48(14)
C(7)-C(5)-C(4)	110.27(13)
C(8)-C(5)-C(4)	109.62(13)
C(5)-C(6)-H(6A)	109.5
C(5)-C(6)-H(6B)	109.5
H(6A)-C(6)-H(6B)	109.5
C(5)-C(6)-H(6C)	109.5
H(6A)-C(6)-H(6C)	109.5
H(6B)-C(6)-H(6C)	109.5
C(5)-C(7)-H(7A)	109.5
C(5)-C(7)-H(7B)	109.5
H(7A)-C(7)-H(7B)	109.5
C(5)-C(7)-H(7C)	109.5
H(7A)-C(7)-H(7C)	109.5
H(7B)-C(7)-H(7C)	109.5
C(5)-C(8)-H(8A)	109.5
C(5)-C(8)-H(8B)	109.5
H(8A)-C(8)-H(8B)	109.5

C(5)-C(8)-H(8C)	109.5
H(8A)-C(8)-H(8C)	109.5
H(8B)-C(8)-H(8C)	109.5
C(2)-N-H(1N)	110.8
C(2)-N-H(2N)	121.7
H(1N)-N-H(2N)	127.1
C(2)-O-C(3)	114.84(13)

Table 4. Anisotropic displacement parameters ($Å^2 x 10^3$). The anisotropic displacement factor exponent takes the form: $-2\pi^2[h^2a^{*2}U^{11} + ... + 2 h k a^* b^* U^{12}]$

	U^{11}	U^{22}	U^{33}	U^{23}	U^{13}	U^{12}
C(1)	16(1)	25(1)	26(1)	-1(1)	-1(1)	0(1)
C(2)	18(1)	23(1)	22(1)	0(1)	-3(1)	-3(1)
C(3)	19(1)	24(1)	15(1)	1(1)	1(1)	-5(1)
C(4)	19(1)	16(1)	14(1)	0(1)	1(1)	1(1)
C(5)	17(1)	18(1)	20(1)	-2(1)	2(1)	-2(1)
C(6)	21(1)	23(1)	26(1)	-3(1)	-5(1)	-2(1)
C(7)	23(1)	36(1)	37(1)	-12(1)	11(1)	-3(1)
C(8)	24(1)	25(1)	25(1)	6(1)	1(1)	-6(1)
N	33(1)	76(1)	20(1)	10(1)	-9(1)	-30(1)
O	19(1)	33(1)	17(1)	2(1)	-2(1)	-11(1)
Cl(1)	24(1)	28(1)	40(1)	11(1)	8(1)	3(1)
Cl(2)	25(1)	43(1)	37(1)	-4(1)	-7(1)	-13(1)
Cl(3)	25(1)	40(1)	42(1)	-7(1)	5(1)	9(1)
Br(1)	24(1)	27(1)	20(1)	-2(1)	7(1)	1(1)
Br(2)	34(1)	18(1)	27(1)	6(1)	-6(1)	0(1)

Table 5. Hydrogen coordinates (x 10^4) and isotropic displacement parameters ($Å^2$x 10^3)

	x	y	z	U(eq)
H(3A)	3109	6422	5227	24
H(3B)	3571	5332	5152	24
H(6A)	1968	4253	2383	37
H(6B)	1508	5344	2433	37
H(6C)	1266	4276	3025	37
H(7A)	1560	6345	4239	47
H(7B)	2026	5858	5353	47
H(7C)	1293	5259	4771	47
H(8A)	2024	3522	4646	38
H(8B)	2699	4179	5327	38
H(8C)	2795	3590	4181	38
H(1N)	4910	6639	6627	23(10)*
H(2N)	4188	6467	6434	19(12)*

*Fixed at position in electron density map and U(iso) refined.

Table 6. Torsion angles [°]

Cl(2)-C(1)-C(2)-N	-6.8(3)
Cl(1)-C(1)-C(2)-N	-127.1(2)
Cl(3)-C(1)-C(2)-N	113.3(2)
Cl(2)-C(1)-C(2)-O	173.49(12)
Cl(1)-C(1)-C(2)-O	53.11(17)
Cl(3)-C(1)-C(2)-O	-66.48(16)
O-C(3)-C(4)-C(5)	-179.32(13)
O-C(3)-C(4)-Br(2)	-56.79(15)
O-C(3)-C(4)-Br(1)	57.69(15)
C(3)-C(4)-C(5)-C(6)	-179.15(14)
Br(2)-C(4)-C(5)-C(6)	60.08(15)
Br(1)-C(4)-C(5)-C(6)	-58.33(15)
C(3)-C(4)-C(5)-C(7)	60.91(18)

Br(2)-C(4)-C(5)-C(7)	-59.86(16)
Br(1)-C(4)-C(5)-C(7)	-178.27(12)
C(3)-C(4)-C(5)-C(8)	-58.36(18)
Br(2)-C(4)-C(5)-C(8)	-179.12(11)
Br(1)-C(4)-C(5)-C(8)	62.46(15)
N-C(2)-O-C(3)	-2.3(3)
C(1)-C(2)-O-C(3)	177.49(14)
C(4)-C(3)-O-C(2)	-173.10(14)

Table 7. Hydrogen bonds [Å and °].

D-H...A	d(D-H)	d(H...A)	d(D...A)	<(DHA)
C(3)-H(3B)...Cl(3)#1	0.99	2.96	3.9244(18)	165.8
N-H(1N)...N#2	0.82	2.63	3.282(3)	138.1
N-H(1N)...Cl(2)	0.82	2.49	2.9587(18)	117.9
N-H(2N)...Br(1)#3	0.66	2.76	3.3345(18)	147.0

Symmetry transformations used to generate equivalent atoms:

#1 -x+1,-y+1,-z+1 #2 -x+1,y,-z+3/2 #3 x,-y+1,z+1/2
